# Dynamotypes for Dummies: A Toolbox, Atlas, and Tutorial for Simulating a Comprehensive Range of Realistic Synthetic Seizures

**DOI:** 10.1523/ENEURO.0200-25.2025

**Published:** 2025-10-15

**Authors:** Christina Sheckler, Kathleen Kish, Zion Walker, Grant Barkelew, Dakota N. Crisp, Matt P. Szuromi, Maria Luisa Saggio, William C. Stacey

**Affiliations:** ^1^Department of Neurology, University of Michigan, Ann Arbor, Michigan 48109; ^2^Department of Biomedical Engineering, Biointerfaces Institute, University of Michigan, Ann Arbor, Michigan 48109; ^3^Department of Mathematics and Statistics, Graduate Program for Neurosciences, Boston University, Boston, Massachusetts 02215; ^4^Aix Marseille Univ, INSERM, INS, Marseille 13284, France; ^5^VA Ann Arbor Healthcare System, Ann Arbor, Michigan 48105

**Keywords:** bifurcation, computational model, dynamics, epilepsy, tutorial

## Abstract

Epileptic seizures involve the brain transitioning from a resting state to an abnormal state of synchronized bursting, akin to a bifurcation in dynamical systems where a parameter shift triggers a qualitative change in behavior. A comprehensive model was previously developed that used dynamical equations capable of simulating 16 “dynamotypes” of seizures that span the full range of theoretical first-order dynamics. The current work is a tool to understand and implement this model with the goal of generating a wide range of synthetic seizures. We present a dynamical atlas of all 16 possible onset–offset bifurcation combinations, each characterized by distinct features in simulated EEG-like recordings. We include a tutorial and graphical user interphase that generates diverse simulated seizures. In addition, we include methods to add realistic noise and filtering effects to enhance their resemblance to human EEG data. This toolbox has two purposes: it is a practical, educational demonstration of the dynamical principles underlying seizure bifurcations, and it provides the algorithms necessary to produce large numbers of realistic, diverse seizure patterns that have similar noise and filtering characteristics as human EEG. This generative model can aid in training seizure detection algorithms, understanding brain dynamical behavior for clinicians, and exploring the impact of noise on EEG recordings and detection algorithms.

## Significance Statement

This work contains a tutorial, atlas, and generative model for a comprehensive, realistic seizure model based on dynamical theory. This user-friendly tool is designed to teach the theoretical principles underlying the model, as well as implement it in order to generate a wide range of simulated seizures that have the same appearance as human EEG recordings. This work is thus broadly applicable to clinicians, students, and researchers.

## Introduction

Epilepsy is one of the most common and debilitating neurological diseases and available treatments are inadequate for a large percentage of patients. Technological advances provide hope for additional treatment options, such as recording devices and data tools that can generate and analyze long-term electroencephalography (EEG) data. However, one of the primary challenges in epilepsy is that seizure recordings can differ greatly from one patient to the next. This variability has led clinicians to develop an empirical approach to identifying seizures based on rules that allow a great deal of flexibility, i.e., the clinical teaching of identifying “rhythmic patterns that differ from background and evolve over time and space.” Given such broad guidelines, the heterogeneity of patients, and the inherently noisy environment of an EEG, it is perhaps not surprising that agreement between EEG experts is limited (Benbadis et al., 2009; Abend et al., 2011). Without clear definitions of seizures, most attempts to develop classification systems are based on observational descriptions (Perucca et al., 2014; Lagarde et al., 2019), which have not yet shown a clear relationship between the specific seizure types and treatment choices or underlying mechanisms. Classifying seizures remains a major challenge: there are a wide range of potential patterns, data are difficult to acquire and analyze, it is difficult to build and annotate multicenter datasets, and it is still unclear which aspects of the seizures are clinically relevant.

Modern computational tools now allow for data-driven classification; however, such tools require many thousands of examples for training. This strategy can be robust when analyzing human EEG data (Kaestner and Stacey, 2023; Jing et al., 2023a,b). However, building a training dataset for human EEG is challenging because it requires expert labeling, which is impractical with large numbers of seizures and somewhat unreliable given the limited agreement between reviewers. Additionally, the “gold standard” of seizure classification is highly subjective.

One option to help overcome these challenges is to generate a large, labeled seizure dataset with a gold standard computational model in order to train detection algorithms. Such a model has several requirements in order to be a valid, realistic training set: (1) it should generate a wide range of seizure patterns similar to those found in humans; (2) seizures should have similar amplitude and scale as human EEG; (3) noise should be included in the background activity as well as in the dynamics of the seizure itself; and (4) acquisition parameters (e.g., filtering and sampling rate) should be similar to those of EEG amplifiers. Recent work (Wang et al., 2024) has begun to generate synthetic, spatiotemporal seizure patterns at the whole brain scale, but even those large-scale projects are limited to one single type of dynamical pattern (Jirsa et al., 2014) and are not designed to have the signal properties of human seizures. To our knowledge, no prior computational model has been developed that satisfies all of these requirements and produces a variety of seizure morphologies. The goal of this work is to present a comprehensive computational model that can generate realistic synthetic seizures.

The first step in the process is choosing a seizure model. There is a long history of modeling seizures, primarily concentrated on exploring the effects of specific pathologies within models (Lytton, 2008; Wendling et al., 2016). While that approach can inform about mechanisms, it is less likely to generate a broad range of seizures. In the current work, we use the Saggio–Jirsa model that encompasses a wide range of basic onset and offset dynamics (Saggio et al., 2017) and is able to create a diversity of bursting patterns that closely resemble human EEG data. This model focuses on invariant properties of the seizure dynamics, rather than specific biophysical mechanisms. It is based on first principles of bifurcation theory: the goal is to produce a signal that has the same key dynamical characteristics, agnostic to specific physiological mechanisms. Being independent from mechanisms is a crucial feature of this approach, because a wide range of different pathologies can produce seizures with similar dynamical characteristics (Jirsa et al., 2014; Saggio et al., 2020), a phenomenon known as degeneracy (Albantakis et al., 2024). In essence, this model produces seizures that “look real” without being specific to one type of epilepsy, which is exactly what is necessary to build a large database of synthetic seizures. Note however that this model is focused just on the onset and offset of seizures; it does not describe interictal activity such as spikes nor more complicated ictal discharges such as spike–wave complexes.

The current work uses that model as a basis for several components, all of which are available for download and are designed for general use. The following components are available at https://github.com/Dynamotypes-for-Dummies:

1. “Dynamotypes for Dummies tutorial” is the eponymous tutorial written as a MATLAB Live Editor. It describes the basic approach for generating seizures with the Saggio–Jirsa model and how it can be used to span a wide range of dynamical properties. It also contains an active code that generates each output as a practical demonstration. Changing parameters (e.g., noise, recording conditions, amplitude, frequency) changes the seizure output directly. This file is saved as Extended Data 1 as Word and PDF documents, and the MATLAB Live Script can be downloaded at this link: https://github.com/Dynamotypes-for-Dummies/dynamotypes-for-dummies-tutorial.
2. Algorithms for realistic noise and filtering. Within the tutorial, we include detailed descriptions of how to generate realistic noise and filtering characteristics and show how these changes produce realistic seizure outputs.
3. Interactive GUI: We include a separate graphical user interface (GUI) in MATLAB, which allows real-time manipulation of the different parameters as sliders, showing how the model produces the voltage signal. It includes graphics that show how these parameters affect the dynamical properties and the output voltage signal. These files are on the GitHub repository. It also includes a video and tutorial for how to adjust this GUI to other parameters and dynamotypes.
4. Dynamotype Atlas: We present an atlas showing multiple examples of each of the 16 dynamotypes, along with a generative MATLAB code to replicate all atlas figures. These files are on the GitHub repository, and a copy of the Atlas is saved as Extended Data 2.
5. Database of Atlas Seizures. The 330 seizures from the Atlas are saved in a file “seizure_atlas.mat” on the GitHub repository.
6. Python code to generate seizures for each dynamotype is also available on the GitHub repository.
7. MATLAB script “create_database.mlx” to generate a wide array of simulated seizures for each class, which can be adapted to produce larger datasets. This file is saved as Extended Data 3 as Word and PDF documents and is also on the GitHub repository as a MATLAB Live Script format.

10.1523/ENEURO.0200-25.2025.d1 Data 1 Microsoft Word and PDF versions of the Matlab Live “Dynamotypes for Dummies Tutorial.” Download Data 1, ZIP file.

10.1523/ENEURO.0200-25.2025.d2 Data 2 PDF version of the atlas of all 16 dynamotypes. Download Data 2, ZIP file.

10.1523/ENEURO.0200-25.2025.d3 Data 3 Microsoft Word and PDF versions of the Matlab Live script “create_database,” which generates large numbers of seizures of different dynamotypes. Download Data 3, ZIP file.

These tools have several purposes. One major goal is to simulate realistic human EEG recordings. Simulated EEG data could be used for training seizure detection or classification algorithms. A major advantage of this model is that it has specific labels that define exactly when seizures start and what type of underlying onset dynamics are present, removing the need for expert labeling and providing a ground truth. Another major goal is to provide teaching about this approach of generating seizures, practical training on how to implement it, and provide code for other researchers to customize for their own purposes. The visualizations and user-friendly parameter adjustment also provide tremendous flexibility for a wide range of applications.

## Materials and Methods

### Introduction to dynamical theory

Dynamical systems are systems whose state changes over time according to a defined rule. In the context of biological modeling, dynamical systems are most often realized in two forms: discrete maps and differential equations. This model is restricted to ordinary differential equations (ODEs).

First-order ODEs consist of equations that relate the time derivative of an independent state variable (which may be a vector) to the current state of the system and possibly external time-dependent forcing. Our core model contains no external forcing. The general form is as follows:

$$\frac{dx}{dt}=f(x).$$

Given starting values for the components of the state variable, known as the initial conditions, the solutions of the ODEs prescribe a unique trajectory that describes the evolution of the state variables, beginning at the initial condition. Explicit solutions to ODEs are highly desirable as they tell us the exact value of the state variable at every time point. However, ODEs can be complex, and their solutions are highly nontrivial, which motivates the so-called qualitative study of ODEs (in “Dynamotypes for dummies tutorial” section, Brief Introduction to Dynamical Systems).

The qualitative theory of ODEs is concerned with describing the long-term behavior of the dynamical system. Does the system “settle down” at a certain value? Does it persistently oscillate? Does it exhibit chaos? These questions motivate the first term of interest, a stable attractor. Attractors are most generally understood as a subset of possible values of the state variable toward which the system evolves. The attractor is stable in the sense that, given a trajectory initialized on the attractor, a sufficiently small, arbitrary perturbation away from the attractor results in the system returning to the attractor. Conversely, a set of states may be unstable, in which case small perturbations result in trajectories which flow away rather than returning to the attractor, in which case the location can be called a repeller.

The two types of attractors pertinent to this work are stable fixed points and stable limit cycles. So-called chaotic or strange attractors are explicitly disallowed in our model, as they only exist in systems of three or more dimensions, while our fast subsystem is only two-dimensional. Furthermore, degenerate solutions like line attractors or centers are not relevant to the present work.

First, fixed points are single states at which the system remains for all time when initialized there. Mathematically, these are disconnected points at which *d***x**/*dt* = 0. Thus, fixed points are solutions of f(x)=0. Writing this in components $f_{i}(x)=0$, for all components 1≤i≤n. For a single fixed *i*, the set of states that solves $f_{i}(x)=0$ is called a nullcline. Thus, fixed points are the intersections of the *n* nullclines. Fixed points can be either stable, unstable, or saddles. Stability of a fixed point is assessed by evaluating the Jacobian matrix at the fixed point. The Jacobian is given as follows:

$$J=\left[\begin{array}{ccc} \frac{\partial f_{1}}{\partial x_{1}} & \cdots & \frac{\partial f_{1}}{\partial x_{n}}\\ \vdots & \ddots & \vdots \\ \frac{\partial f_{n}}{\partial x_{1}} & \cdots & \frac{\partial f_{n}}{\partial x_{n}} \end{array}\right].$$

The Jacobian evaluated at the fixed point describes the growth or decay of small perturbation. A fixed point is stable when the real parts of the eigenvalues of the Jacobian are all negative. A fixed point is unstable if the real parts of all eigenvalues are positive. A fixed point with a mix of eigenvalues with positive and negative real parts is a saddle. If the eigenvalues are all real, the fixed point is known as a node. If eigenvalues are complex, then we call the fixed point a spiral.

Limit cycles are periodic solutions of ODEs, *x*(*t*), such that x(t)=x(t+T) for some finite period *T*. Limit cycles can also be stable or unstable. The stability of limit cycles is assessed via Floquet theory (Encyclopedia of Mathematics, 2025), which is beyond the scope of this tutorial. Table 1 gives a concise description of attractors and repellers (from “Dynamotypes for dummies tutorial” section: Brief Introduction to Dynamical Systems; subsection: Long-Term Behavior, Attractors, and Stability). In a dynamical system, parameters are fixed values that influence the system’s behavior but do not evolve with time. For example, in the one-dimensional equation, dx/dt=f(x)=ax, a (a real number) is the sole parameter. Clearly, choosing different parameters will produce distinct dynamics, changing the state trajectories from a given initial condition. Thus, varying parameters can shift the location of attractors in the state space. Furthermore, the qualitative dynamics may be altered. Loosely, a qualitative change in dynamics as parameters are varied is known as a bifurcation, and the parameters that induced the bifurcation are known as bifurcation parameters. In the context of this work, bifurcations can be understood as (1) the creation/annihilation of attractors/repellers or (2) the change in stability of an attractor/repeller (further elaborated in “Dynamotypes for dummies tutorial” section, Brief Introduction to Dynamical Systems; subsection, Bifurcations).

**Table 1.** Description of attractors and repellers

Attractor/repellor	Description	Intuition
Stable fixed point	A point in a dynamical system where, if the system is slightly perturbed in any direction, it will return to that point	Like a marble at the bottom of a bowl—no matter where you nudge it, it will roll back to the bottom. Stable because it always returns to the same spot
Unstable fixed point	An unstable fixed point is a point in a system that repels nearby trajectories. If the system is at that point and is perturbed, it will not return to that point	Like a marble balanced on top of a dome—any tiny push will cause it to roll away. Unstable because even the smallest push will move it away
Saddle fixed point	A point where the system may be attracted in some directions but repelled in others	Like a marble on a horse saddle—it stays put if moved front-to-back but rolls off side-to-side. Saddle because stable in one direction but unstable in another
Stable limit cycle	A stable limit cycle is a repeating pattern or cycle in a system that attracts nearby trajectories. If the system is slightly perturbed, it returns to this cycle	Like a ceiling fan spinning at steady speed—even if slowed slightly, it returns to original rhythm. Stable because it settles into repeating motion
Unstable limit cycle	An unstable limit cycle is a repeating pattern or cycle in a system that repels nearby trajectories	If you spin a coin perfectly, slight wobble eventually causes it to fall. Unstable because the spinning motion is hard to maintain

### Bifurcation theory as a model of seizures

There are several approaches to model seizures (Wendling et al., 2016), ranging from highly physiological models that include structure and specific channels (Traub et al., 2005) to mean field approximations that generalize the bulk effects of excitation and inhibition (Demont-Guignard et al., 2012) to dynamical descriptions that focus on how certain behaviors can be produced, agnostic to specific mechanisms (Saggio and Jirsa, 2023). Our work uses the latter approach, as introduced in Saggio et al. (2017). This model is phenomenological: it aims to use the simplest mathematical expression able to reproduce some relevant dynamics hypothesized to underlie seizures. It is important to stress that variables and parameters in phenomenological models are abstract and do not have an obvious biophysical correlate other than one of the variables reproducing the voltage timeseries. This type of phenomenological approach has been often been used to build large-scale brain models due to their lower computational burden (Hutchings et al., 2015; Sinha et al., 2017; Lopes et al., 2020; Hashemi et al., 2021).

As described extensively in prior work (Jirsa et al., 2014; Saggio et al., 2017; Saggio et al., 2020), the underlying phenomenon guiding this model is that seizures are defined by abrupt transitions in dynamics. In the field of dynamical systems, this type of transition between two states requires proximity to a bifurcation (Izhikevich, 2010). The Saggio–Jirsa model (Saggio et al., 2017) assumes that a seizure’s onset and offset are due to the system going through such bifurcations [see the Supplementary material of Saggio et al. (2020) for how to use the model for input-induced transitions, another proposed mechanism for transitions]. A feature of this approach is that it is independent of specific mechanism—the bifurcations are invariant and preserved across mechanisms, species, and etiologies (Jirsa et al., 2014).

**Figure 1.**
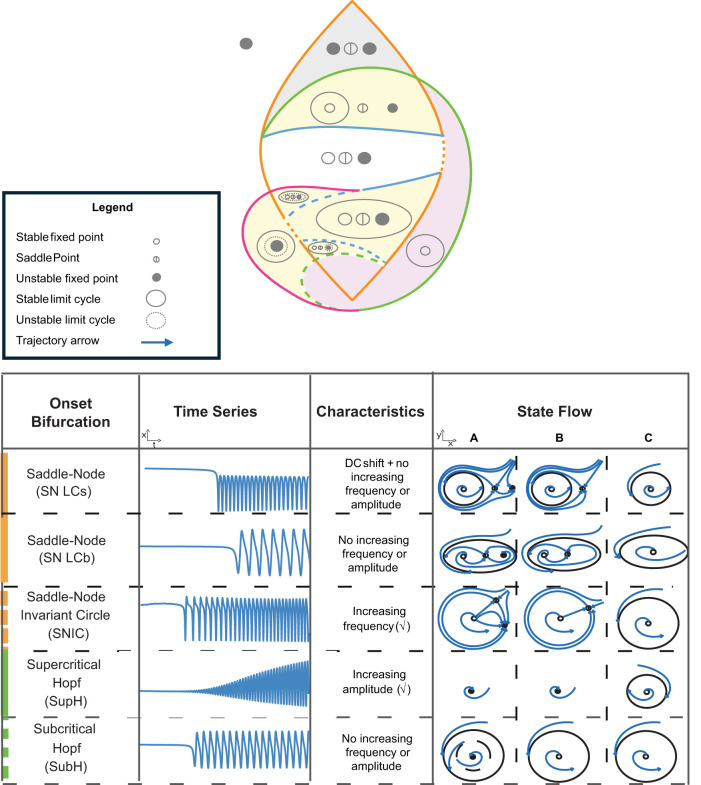
Onset bifurcations. The top portion of this figure shows a bifurcation diagram of the Saggio–Jirsa model. The lower portion shows visualization of its onset dynamics. SN onsets can arise with or without direct current (DC) shifts, which were distinguished by the presence of big (LCb) or small (LCs) limit cycles in state space (Saggio et al., 2017). Bifurcations scale from zero based on square root scaling laws as indicated. The lower right portion of the figure illustrates the state flow diagrams for key bifurcations, showing how system trajectories evolve through state space during seizure onset. Column A shows the state space before the bifurcation has occurred, where the system is at rest. Column B shows the state space at the bifurcation. Column C shows the state space after the bifurcation where the system is in the limit cycle.

**Figure 2.**
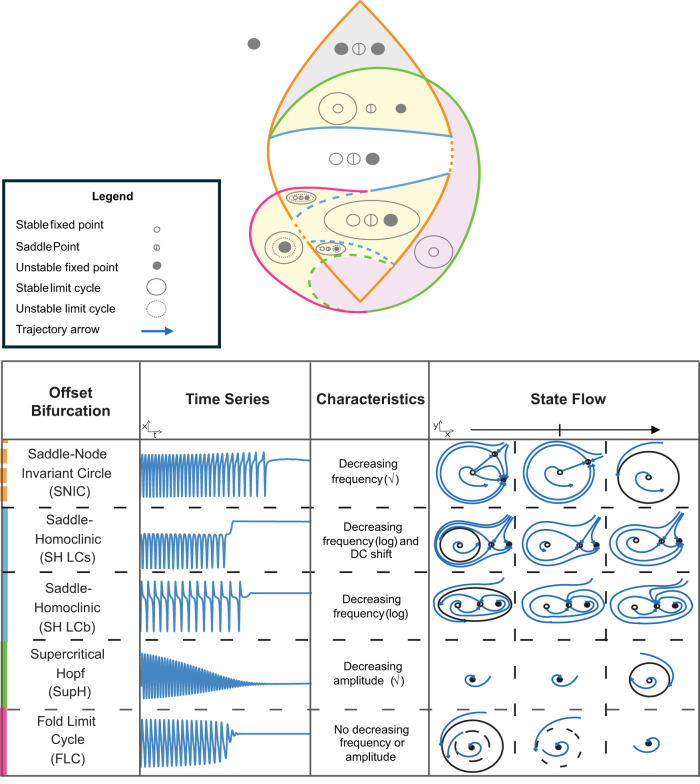
Offset bifurcations. The top portion of this figure shows a bifurcation diagram of the Saggio–Jirsa model. The lower portion shows visualization of its offset dynamics. SH offsets can arise with or without direct current (DC) shifts, which were distinguished by the presence of big (LCb) or small (LCs) limit cycles in state space (Saggio et al., 2017). Bifurcations scale to zero based on the either square root or logarithmic scaling laws as indicated. The lower right portion of the figure illustrates the state flow diagrams for key bifurcations, showing how system trajectories evolve through state space during seizure offset. Column A shows the state space before the bifurcation has occurred, where the system is in the limit cycle. Column B shows the state space at the bifurcation. Column C shows the state space after the bifurcation where the system is at rest.

The Saggio–Jirsa model is based on four different onset [saddle node (SN), SN invariant circle (SNIC), supercritical Hopf (SupH), subcritical Hopf (SubH)] and offset [saddle homoclinic (SH), SNIC, SupH, and fold limit cycle (FLC)] bifurcations. Each of these has distinct, well described dynamical properties. Figures 1 and 2 show an illustration of onset and offset dynamics.

**Figure 3.**
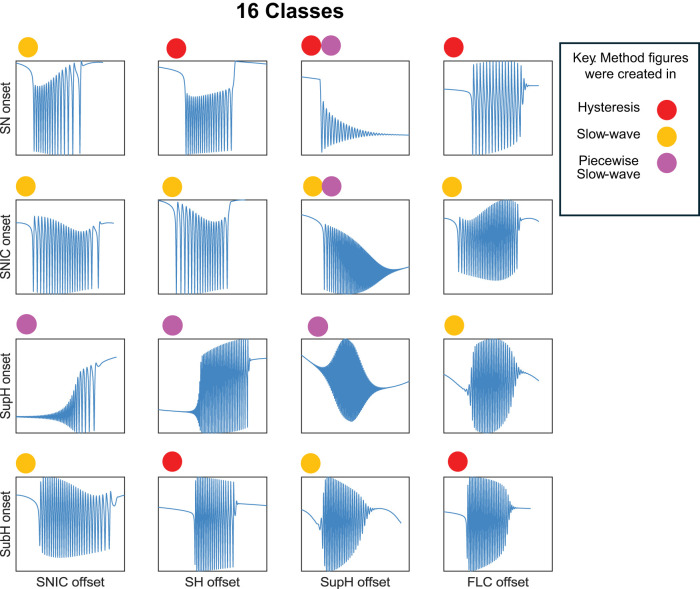
Sixteen seizure dynamotypes, distinguished by their combination of onset and offset dynamics. Examples herein are generated using the different techniques described in this work.

The Saggio–Jirsa model results in a taxonomy covering the 16 possible combinations of onset/offset bifurcations (Izhikevich, 2000), which are called dynamotypes (Saggio et al., 2020). Figure 3 shows a characteristic example of each dynamotype, generated using the methods presented here. Note that the SN (LCb) and SubH onsets, as well as the SN (LCb) and FLC offsets, all have arbitrary scaling and thus cannot be distinguished from each other in the time series data.

One key aspect of the original model is fast-slow bursting, which explains both the fast rhythms of oscillations within the bursting state (i.e., the spiking rate within a seizure) and the slow rhythm of transitions between the bursting and resting state (i.e., the transition between seizure and rest; Saggio et al., 2017). That model described oscillations in the bursting state by the time evolution of a fast subsystem (fast variables, *x*, *y*). Plotting the *x* variable over time resembles the voltage tracing of an EEG, specifically an intracranial electrode that samples from a small region of the brain, such as a stereo-EEG (SEEG). The transition between resting and bursting states is dictated by the time evolution of a slow subsystem (slow variable, *z*). The slow subsystem aids in steering the fast subsystem in parameter space so that bursting can occur. These variables form a system of equations that creates the model (Eq. 1):

$$\begin{array}{rl} & \dot{x}=-y\\ & \dot{y}=x^{3}-\mu_{2}(z)\;*\;x-\mu_{1}(z)-y(\nu (z)+x+x^{2})\\ & \dot{z}=\mathrm{Slow}\_\mathrm{Variable}. \end{array}$$

**Figure 4.**
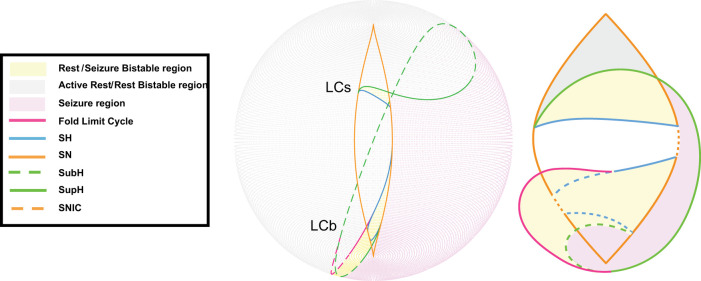
Left: Spherical projection of the map, partially transparent. Colored lines represent bifurcations, and shaded areas identify regions where specific brain states are possible (i.e., seizure and rest). Right, Stylized two-dimensional projection of the same map.

The two fast variables *x* and *y* produce either rest, oscillations, or both, depending on the values of the three parameters (*µ*_1_, *µ*_2_, and *ν*) in Equation 1. The slow variable *z* affects how the model moves around parameter space and can induce bursting in the fast variables. We simulate *z* using multiple methods described below. Instead of looking at the behaviors of the fast variables for all possible combinations of the three parameters, for this system, we can, without loss of generality (Dumortier et al., 1991), restrict the description to those combinations lying on a spherical surface with small enough radius. This two-dimensional spherical surface creates a “map” with bifurcation curves that partition the surface into separated “regions” (Fig. 4). Within one region, the fast subsystem exhibits similar behaviors even though quantitative features may change. For example, a region may be characterized by oscillations, but the specific frequency or amplitude may change within it. This parameter-space map contains regions that correspond to brain states of either seizure, rest, or bistability. One can model movement within the parameter space by varying the input coordinates *µ*_1_, *µ*_2_, and *ν*, which will correspond to navigating around the map. The model’s (i.e., simulated brain’s) activity will change depending on the specific bifurcations it crosses during this navigation and the region it enters. For instance, if the parameters move the system from “rest” to “seizure” state by crossing a bifurcation, a seizure will begin. In a bistable region where rest and seizure states coexist, the system does not necessarily need to leave the region for a seizure to begin. If, initially in the rest state, the system can transition into a seizure if the appropriate bifurcation curve is met, then the system reverses direction and remains in the bistable region. Bistable regions can also generate seizures if input to the system such as noise or stimulation pushes it across the threshold that separates the rest state from the seizure state, without the need for movement on the map. Overall, this model provides a rational explanation for why some brains are more likely to seize at certain times if they are closer to an onset bifurcation in parameter space.

One clarification is necessary regarding terminology and labeling. There are bursting paths in which the system first crosses one bifurcation (e.g. SN), which does not lead directly to oscillations until after another bifurcation is crossed (e.g., SupH). By the classical definition (Izhikevich, 2000), seizure would be labeled a SupH [Saggio et al. (2017), their Fig. 8]. However, that distinction is ambiguous in vivo, where the presence of a DC shift is the sine qua none of a SN bifurcation, and it may not be possible to ascertain which bifurcation caused the bursting. Therefore, following a recent labeling convention [Saggio et al. (2020), their Fig. 4*C*], showing the same pattern from Saggio et al. (2017; labeled as a SN), we classify a seizure with a DC shift at onset as a SN (an example is seen in the bottom left of Fig. 4). That is, under conditions where there are two bifurcations necessary for onset—one that destabilizes the resting state and a second that causes oscillations—we define the seizure by the first bifurcation. Because of this convention, some of the dynamotypes proposed in Saggio et al. (2017) had not been previously generated, which necessitated developing additional methods.

### Generating seizures

**Figure 5.**
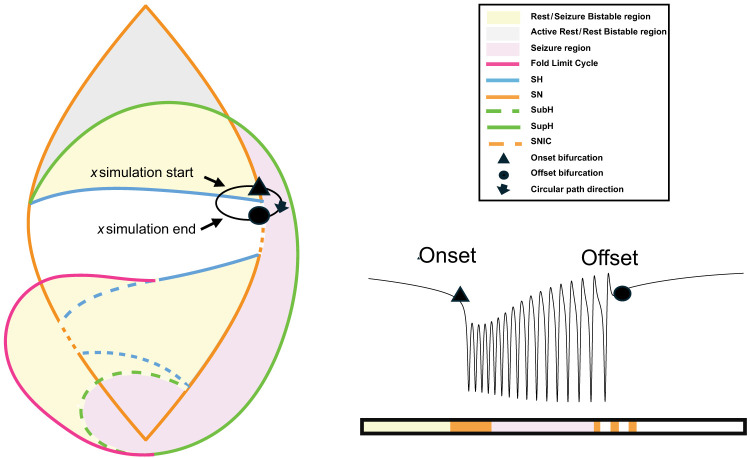
Parameter-space path (left) shown with corresponding simulated seizure trace of the variable *x* (right).

To generate a simulated seizure using the Saggio–Jirsa model, we must identify a path through parameter space that “connects” onset and offset bifurcation curves. We call this path the bursting path; it is the set of parameter values along which the system varies in order to exhibit the proper sequence of bifurcations that yield a burster. To create a burster of a particular class, we must find a path that appropriately connects the correct onset and offset bifurcations. Movement of the system along this path is accomplished by parameterizing the bursting path in terms of the slow variables, *z*. A sample bursting path is shown in Figure 5.

We traversed the parameter-space map using three methods to change the slow variable in Equation 1. Each method functions by changing the parameters *µ*_1_, *µ*_2_, and *ν* to define the position of the system on the spherical surface. The first two methods, hysteresis-loop bursting, slow-wave bursting using circular paths (Izhikevich, 2000; Golubitsky et al., 2001), were previously implemented by the Saggio–Jirsa model (Saggio et al., 2017). Here, we present a third method (slow-wave piecewise bursting) to simulate noncircular pathways that provide more customizable pathways. Note that because the parameter-space map is a spherical surface, paths in all three methods are made of arcs and circles.

#### Hysteresis-loop bursting

**Figure 6.**
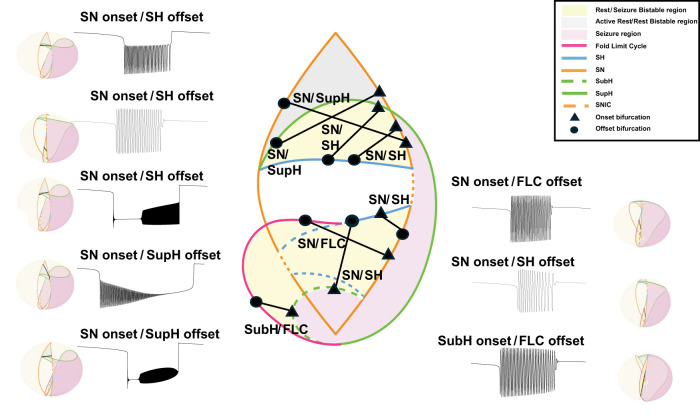
Sample hysteresis-loop bursting paths for each dynamotype on the 2D projection of the map (center) as well as the 3D image of the map and a corresponding seizure generated from the highlighted paths. The transparent sphere has been rotated in each case to highlight the bursting path in black.

In their simplest implementation, the hysteresis-loop bursts follow arc-shaped paths between an onset bifurcation curve and an offset bifurcation curve, through a bistable region. The hysteresis is produced by the slow variable receiving feedback from the fast subsystem to drive the transition between states to create a coiled spring-like path that “pushes or pulls” the system back to resting state as in Saggio et al. (2017; Eq. 2). We used this method to produce five dynamotypes, summarized in Figure 6. We generated different seizures for each dynamotype by choosing several different locations on the onset and offset bifurcation arcs:

$$\begin{array}{rl} & \dot{x}=-y\\ & \dot{y}=x^{3}-\mu_{2}(z)\;*\;x-\mu_{1}(z)-y(\nu (z)+x+x^{2})\\ & \dot{z}=-c\left(\sqrt{{\left(x-x_{s}\right)}^{2}-{\left(y-y_{s}\right)}^{2}-\;d^{*}}\right), \end{array}$$

where d* is a parameter that defines how close the system is to a threshold of excitability for bursting and $x_{s},y_{s}$ are the stable fixed points of each fast variable when the system is in the resting state and *c* is the velocity of the slow variable (Saggio et al., 2017).

#### Slow-wave bursting

A simple implementation of slow-wave bursting follows a circular path created by using one point on the onset curve, one point on the offset curve, and a third point on the sphere. There is no feedback from the fast subsystem; rather, the slow subsystem autonomously travels along the closed path, here at a constant speed as in Equation 3 (Saggio et al., 2017):

$$\begin{array}{rl} & \dot{x}=-y\\ & \dot{y}=x^{3}-\mu_{2}(z)\;*\;x-\mu_{1}(z)-y(\nu (z)+x+x^{2})\\ & \dot{z}=k. \end{array}$$

**Figure 7.**
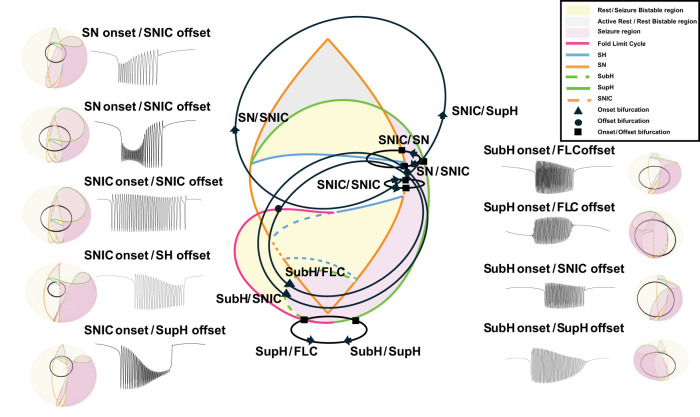
Sample slow-wave bursting paths for each class on the 2D projection of the map (center) as well as the 3D image of the map and a corresponding seizure generated from the highlighted paths.

As before, we generated several examples for each dynamotype by simulating paths that intersect with several points along each onset and offset curve (see atlas). We produced seven dynamotypes with this method, which are summarized in Figure 7. All dynamotypes can be produced using this method, but some must cross additional bifurcations to complete the circle.

#### Piecewise bursting

**Figure 8.**
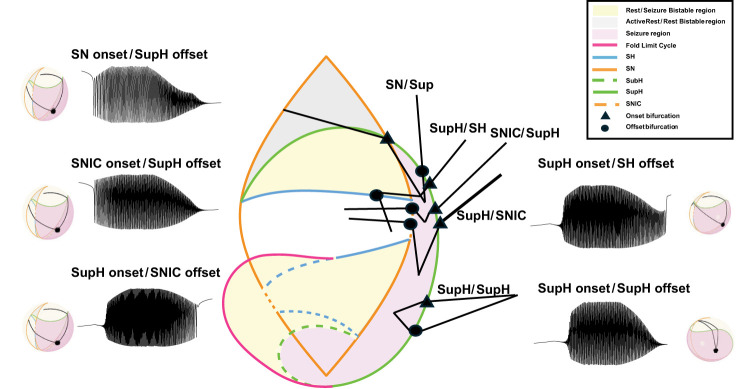
Sample piecewise bursting paths for each class on the 2D projection of the map (center) as well as the 3D projection of the map and a corresponding seizure generated from the highlighted paths.

The third method for traversing the map is another implementation of slow-wave bursting. The piecewise method allows the user to customize paths across specific bifurcations and starting/ending points. This method also uses Equation 3, although the *k*(*t*) term is defined differently. To elicit piecewise bursting, we used direct pathways between four defined points to move through the parameter space. The first point is a point in the rest region. The second point is on the onset bifurcation curve. The third point is a point in the limit cycle (seizure) region. The fourth point is on the offset bifurcation curve. Four arcs are created to generate a continuous path between the points. We included delays at the turning point of some simulations to represent spending longer periods in the bursting state. Note that this method has a specified beginning and endpoint, which is a different implementation than the prior two and allows the brain to traverse to new conditions after the seizure, suggesting the plausible situation that the seizure had modified the values of the parameters. Also note that it is possible to start/stop the other methods in different locations as well if desired, as shown previously (Saggio et al., 2020; Saggio and Jirsa, 2024). We produced five dynamotypes with this method, which are summarized in Figure 8. One advantage of this approach is to represent specific dynamotypes without also crossing additional bifurcations. While one could also use a truncated version of slow-wave bursting for this same effect, this piecewise bursting is more versatile because the pathways are not constrained to circles.

### Adding noise to the simulation

Model-generated seizures may have realistic dynamics, but they lack a key feature of experimental/clinical seizure recordings: noise. Real EEG always contains some level of noise, because the brain itself generates background activity that has a 1/*f* or “pink” power spectrum (Barry and De Blasio, 2021). There are two primary types of noise present in real EEG recordings: dynamical noise (i.e., noisiness in the brain behavior itself) and background noise (i.e., noise produced by background brain activity and from the electronics of the acquisition system).

#### Dynamical noise

We added dynamical noise, or parametric noise, to the model equations in the Euler–Maruyama update (Eq. 4). This noise represents random voltage fluctuations in the brain that perturb the system, sometimes pushing the system into or out of the seizure state (Lopes da Silva et al., 2003; Jirsa et al., 2014; Maturana et al., 2020). It is added to the fast variable *x* of Equations 1–3, which represents the voltage of the system:

$$\left[\begin{array}{c} x_{n+1}\\ y_{n+1}\\ z_{n+1} \end{array}\right]=\left[\begin{array}{c} x_{n}\\ y_{n}\\ z_{n} \end{array}\right]+\;t\mathrm{step}\;\;\;\left[\begin{array}{c} \dot{x}\\ \dot{y}\\ \dot{z} \end{array}\right]\;\;+\;\sqrt{t\mathrm{step}\;}\;\left[\begin{array}{c} \mathrm{pinknoise}(n)\\ 0\\ 0 \end{array}\right].$$

The effect of dynamical noise depends on the specific path through parameter space. This is because (1) integration method scales differently for different paths and (2) specific values for noise have produced different effects in different dynamical regimes. Therefore, for each dynamotype, we created an empirical model for dynamical noise amplitude. We manually adjusted the noise amplitude for a subset of paths and found the lowest amplitude to create a 5–10% deviation in seizure length across 10 simulated seizures. We fit a surface to the tested points (“linearinterp” in MATLAB). Using these surfaces, we assigned an appropriate noise amplitude to every simulated path.

#### Background noise

We also added background/acquisition noise to our simulated seizure data by adding noise directly to the generated simulated seizure data. This background activity can be modeled as pink noise, which we generate using 1/*f* noise with a normal error distribution (Lennon, 2000) and which is simply added to the prior outputs, which are the *x* values in the prior equations (Eq. 5):

noisySim=x+pinkNoise*α*max(x).

**Figure 9.**
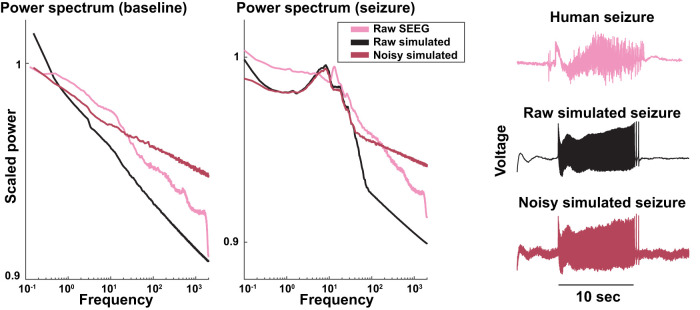
Left, Power spectral density during interictal periods for raw simulated data, postprocessed simulated data, and human SEEG data. Adding noise to the simulation adds higher frequencies similar to the human data. Middle, Power spectral density during a seizure for raw simulated data, postprocessed simulated data, and human SEEG. Note the baseline trace (left) clearly demonstrates a 1/*f* decay consistent with pink noise, confirming that the implementation aligns with theoretical expectations in the absence of seizure dynamics. In contrast, during seizure periods (middle), the dynamics of the seizure dominate the spectrum, naturally overriding the expected 1*/f* decay. This is to be expected, as seizure activity introduces structured rhythmicity and amplitude modulation that diverge from purely stochastic noise. Right, A human seizure recorded with SEEG (top) compared with simulations without (middle) and with (bottom) noise. The addition of pink noise to the simulated data makes it look realistic in the time series.

We added a range of background noise amplitudes that were scaled to the amplitude of the bursting: *α* = 0% (no noise), 20% (moderate noise), and 40% (high noise). We tested the effect of the added background noise by comparing the power spectra of simulated seizures with human data (Fig. 9). Without background noise, spectral power dropped very quickly at frequencies >30 Hz. Adding the background noise caused power to mirror true physiological recordings by increasing the spectral power making a slope of 1/*f*^ a^ (Barry and De Blasio, 2021). Visual observation of the raw signals corroborated those results: adding noise makes the simulation look closer to realistic EEG. Background noise can be added using the toolbox function *add_pink_noise*() to match physiological data.

### Signal processing to simulate physiological recordings

We performed four postprocessing steps to increase the resemblance between model data and human SEEG recordings.

First, we normalized the amplitude so that the maximum value was 1 and the minimum value was 0 because the amplitude produced by the dynamical model is arbitrary.

Second, we scaled the simulated seizures so that the spike frequency was consistent with clinical guidelines. The original model in Saggio et al. (2017) has arbitrary time; thus for each seizure, we defined the effective sampling frequency for the tracing to make the spikes during a seizure be between 1 and 30 Hz, as is typical for human seizures.

**Figure 10.**
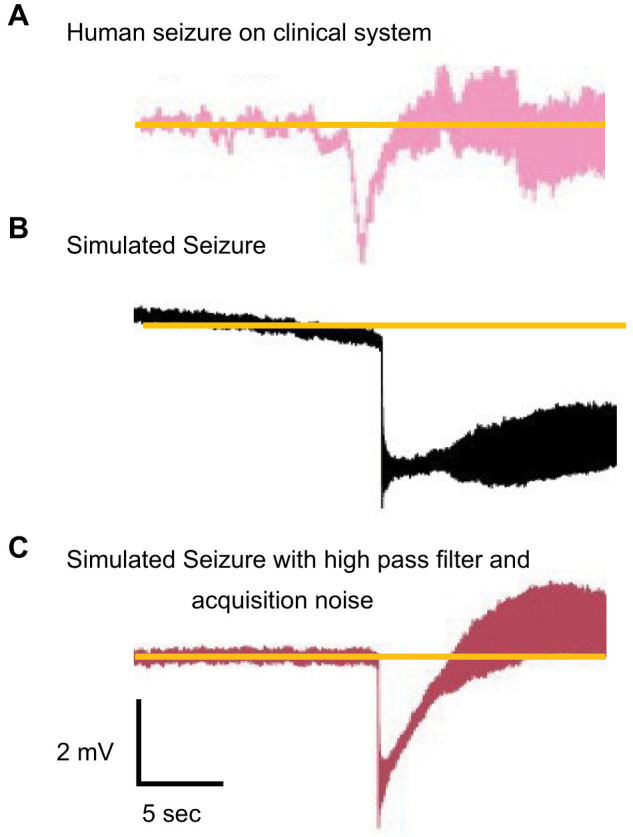
Top. Human seizure data showing a likely DC shift at the start of a seizure are attenuated in the EEG recording system (data are from a standard clinical SEEG, a platinum-iridium SEEG electrode recorded with Natus Quantum amplifier). Middle, The appearance of a DC shift in simulated data. Bottom, Postprocessing of simulated data using a high-pass filter to model electrode drift effects. Simulated data were timescaled and filtered to match human data using the algorithms above.

Third, we filter the time series to simulate the effect of clinical EEG recording systems, based on that sampling frequency. Clinical EEG electrodes are not optimized for accurate DC recordings (Merrill et al., 2005), and their filtering properties can distort the lower frequencies of EEG signals (Stacey et al., 2013). In addition, most commercial systems have a high-pass filter 0.1 or higher. This filter removes electrode drift but also distorts DC shifts that can occur during seizures. To emulate these filtering properties, we applied second-order digital high-pass filters with cutoff frequencies between 0.1 and 1 Hz (Fig. 10).

Fourth, we doubled the dataset by duplicating each simulation with the amplitudes vertically flipped. This removes the effect of polarity of the spiking, which is arbitrary in SEEG because the dipole of the spiking region can have any orientation.

**Figure 11.**
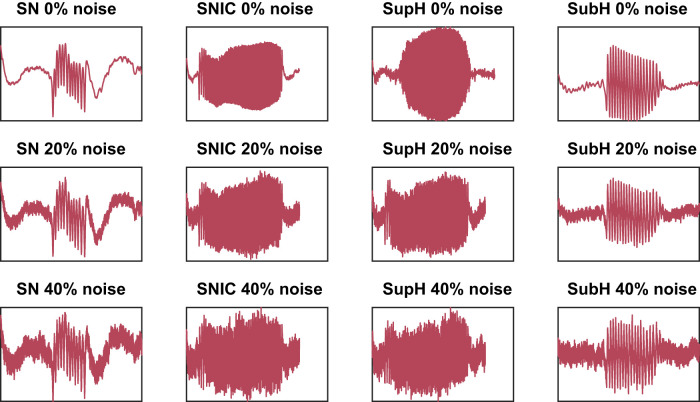
Four sample simulated seizures with postprocessing adding three levels of acquisition noise and then electrode filtering.

Examples of this process are demonstrated in Figures 10 and 11.

### Scaling output to physiological data

The simulated seizures generated by our model are not intended to be exact replicas of all human seizure activity but rather to capture key biological features that are most relevant for seizure onset detection and onset/offset bifurcation classification. The model's flexible parameterization enables users to generate a wide range of seizure morphologies observed in clinical EEG. While one method to treat such data is to use normalized scales and treat the dynamics independent of scale, it is also desirable to be able to match the synthetic seizures with physiological data. This model allows for arbitrary scaling that can be matched to data of interest. Amplitude can be modified by a parameter alpha in the model, and furthermore, it is fully user-defined, acknowledging the variability introduced by differences in hardware, electrode placement, and normalization practices. Duration is controllable via the *k* and k_fast parameters and choice of dynamical path. The signal-to-noise ratio (relative amplitude between the bursting and the noise signal) is a parameter that can be set to any arbitrary level. Moreover, the model’s bifurcation-based structure imposes biologically meaningful temporal patterns at onset and offset, which resemble the electrophysiological transitions seen in real seizures. This framework also allows for systematic generation of underrepresented seizure types, including slow-amplitude growth (e.g., SupH onset), DC shifts with varying degrees of recovery to the baseline (via postprocessing high-pass filtering), and SNIC bifurcation onsets that are rare in focal epilepsy (Saggio et al., 2020). These edge cases are commonly missed by traditional machine learning (ML) models due to limited labeled data. Our simulation platform overcomes this limitation by enabling large-scale, parameter-swept generation of diverse synthetic seizures, thus mitigating overfitting and improving generalization across patient populations. While exhaustive biological replication is not the goal, the model’s design prioritizes flexibility, interpretability, and practical utility in addressing gaps in seizure detection and classification.

### Code availability

All of the following files are available for download on GitHub at https://github.com/Dynamotypes-for-Dummies.

**Figure 12.**
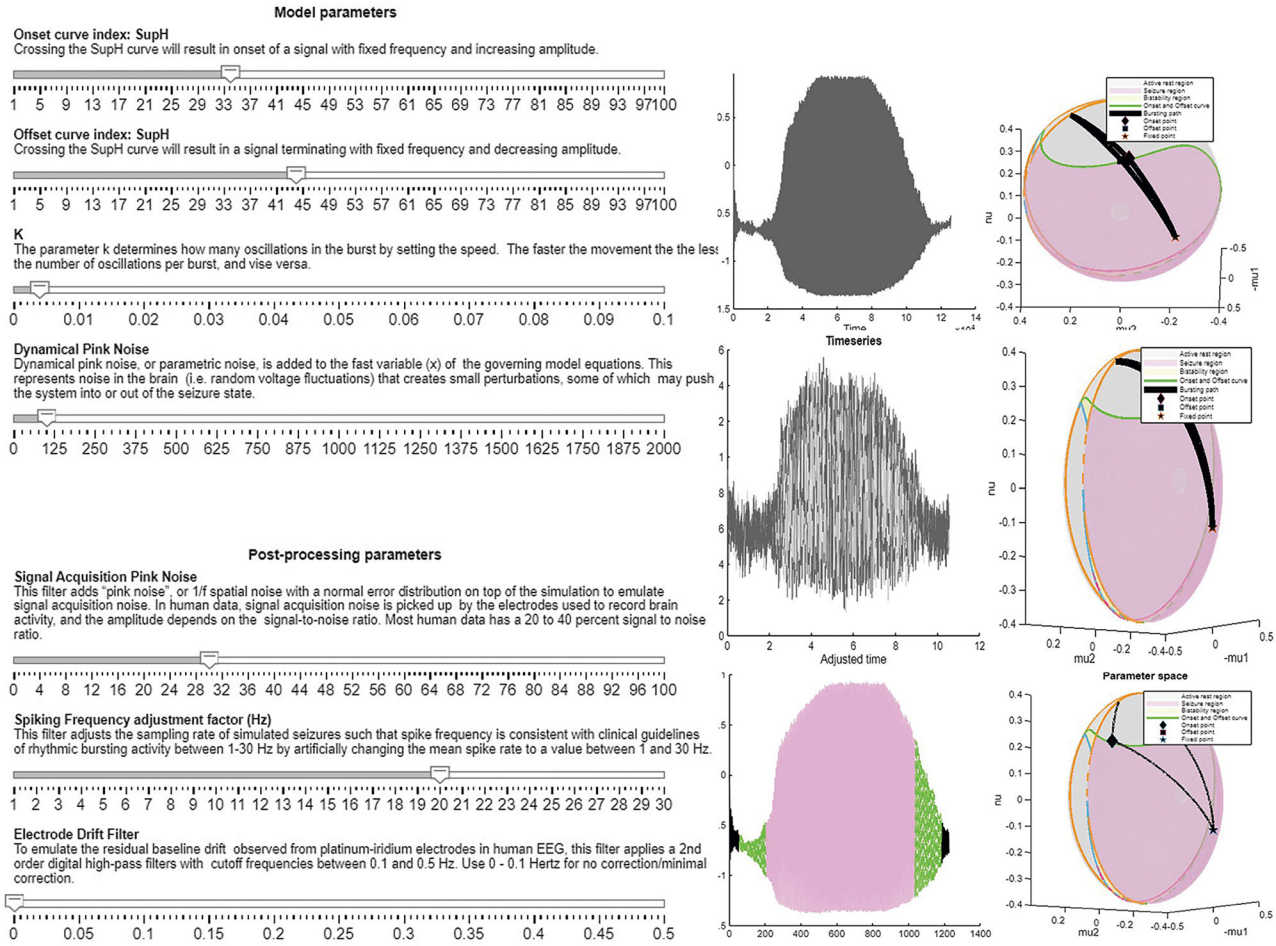
This figure shows the interface of the GUI. On the left are the parameters for the piecewise GUIs. The right shows the output of pressing the three buttons Run Simulation, Run Simulation with Postprocessing, and Run Video Simulation, respectively. There is also a button to save the timeseries as a .mat file.

1. “Dynamotypes for Dummies Tutorial”: This is a MATLAB Live Script that explains the background of the model. It explains the mathematical framework behind the model based on several prior works (Bertram et al., 1995; Izhikevich, 2000; Golubitsky et al., 2001; Jirsa et al., 2014; Strogatz, 2015; Saggio et al., 2017; Crisp et al., 2020; Saggio et al., 2020; Saggio and Jirsa, 2023; Depannemaecker et al., 2022; Szuromi et al., 2023) and offers a step-by-step guide to the code, including features like choosing onset and offset points, adding dynamical noise, and conducting postprocessing. Together, these resources allow researchers to create custom datasets for training models, further aiding in seizure detection and classification tasks. For users without access to MATLAB, Extended Data 1 has a Word and PDF version of this document; it allows review of the principles taught but will not run the simulations.
2. GUI: Interactive GUIs are included to allow users to generate and visualize seizures across three dynamotypes. These GUIs feature sliders for parameter adjustment and buttons for generating seizures, performing postprocessing, and simulating the dynamics of the seizure as a video. The synchronized video simulation highlights the seizure creation process, model dynamics, and postprocessing results, making the tool intuitive and user-friendly. An example snapshot is shown in Figure 12.
3. Python model code: For readers without MATLAB, a Python code to generate all 16 dynamotypes is provided in Python. Additionally, three Python scripts—*Slowwave.py*, *Hysteresis.py*, and *Piecewise.py*—are available. These scripts can independently generate seizures, showcasing specific dynamical behaviors associated with the model. In order to account for different versions of Python, a video walkthrough is provided to install and run on the online platform colab.research.google.com.
4. Atlas: The atlas shows several trajectories for all 16 dynamotypes. It includes a Word document *Atlas* describing all 16 classes in detail, with figures illustrating five representative paths and seizures for each class, with no noise, low noise, and high noise. The code *make_atlas.m* is provided to generate the atlas, which produces 330 total sample seizures in the file *seizure_atlas.mat*, including these representative trajectories. A PDF version of the printed atlas is present in Extended Data 2. The MATLAB data file of the 330 seizures is present in the GitHub.
5. Generate database: This includes the script *create_database.mlx* to generate a dataset containing an array of seizures for each class, with the ability to customize the simulation parameters used by the model. A PDF and Word version of this MATLAB Live Script is provided in Extended Data 3 for users without access to MATLAB.

## Results

The provided GUI, tutorial, atlas, and code can generate numerous pathways from all 16 dynamotypes, with a wide range of noise levels. Producing all 16 dynamotypes required three methods of producing bursting by traversing the dynamical map, which are each presented below. In the figures, the parameter-space diagrams have a boxed area to compare between the stylized 2D representation and the true mathematical projection. Each figure has six points: three for onset (orange) and three for offset (blue). Gray paths represent the bursting paths between five pairs of onset/offset points. The five paths in state space generate five seizures, which have the corresponding EEG time series shown. There are three levels (0, low, high) of noise added to each of the five seizures.

### Hysteresis-loop example: SN/SH dynamotype

**Figure 13.**
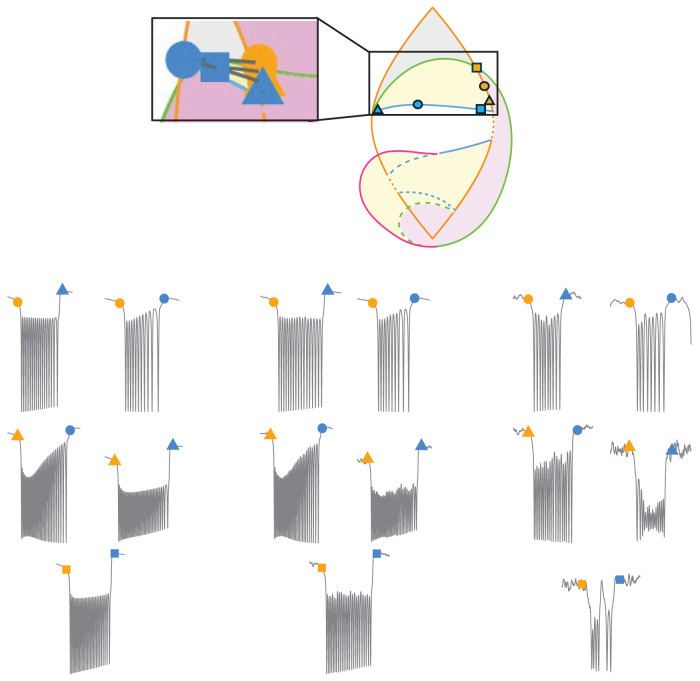
SN/SH dynamotype trajectories formed with hysteresis bursting. Low noise (left group), medium noise (middle group), and high noise (right group) show the effects of dynamical noise. High noise in this dynamotype can push the system into/out of the bistable state independently, which makes some of the seizures shorter in this case. It can also induce seizures spontaneously (data not shown).

The SN/SH dynamotype has a SN onset bifurcation and a SH offset bifurcation arise in the top portion of the parameter-space map. This class, which is very common in human intracranial EEG recordings and conserved across multiple species, was previously modeled as the *Epileptor* (Jirsa et al., 2014). In the *x* time series, this type of seizure creates a DC shift that begins when the seizure starts and ends when the seizure stops (Fig. 13). Spiking frequency slows down logarithmically at seizure offset.

### Slow-wave example: SNIC/SH dynamotype

**Figure 14.**
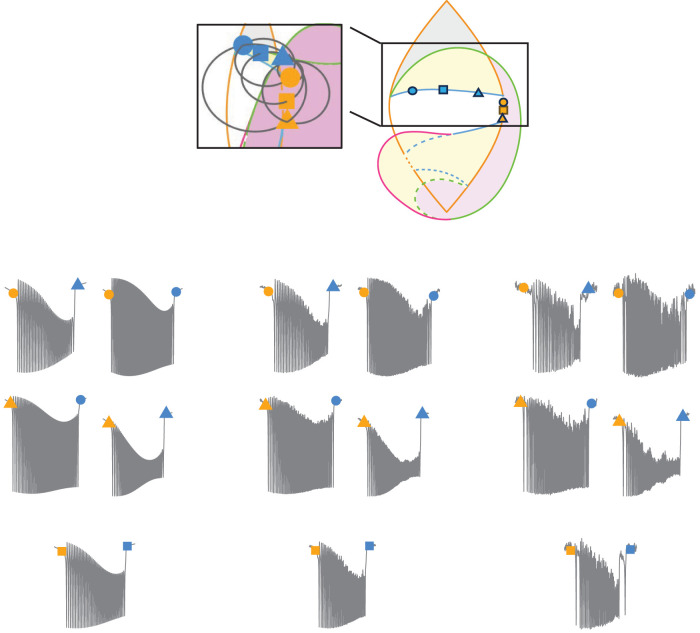
SNIC/SH dynamotype trajectories. In this dynamotype, most of the bursting does not occur in a bistable state (pink, obligatory bursting region), and thus even high noise (right group) does not affect the bursting characteristics significantly.

This dynamotype has a SNIC onset and a SH offset, characterized by square root scaling of the frequency at onset and logarithmic frequency scaling at offset, along with a possible DC shift (the chosen trajectories all have a DC shift; Fig. 14).

### Piecewise example: SupH/SH dynamotype

**Figure 15.**
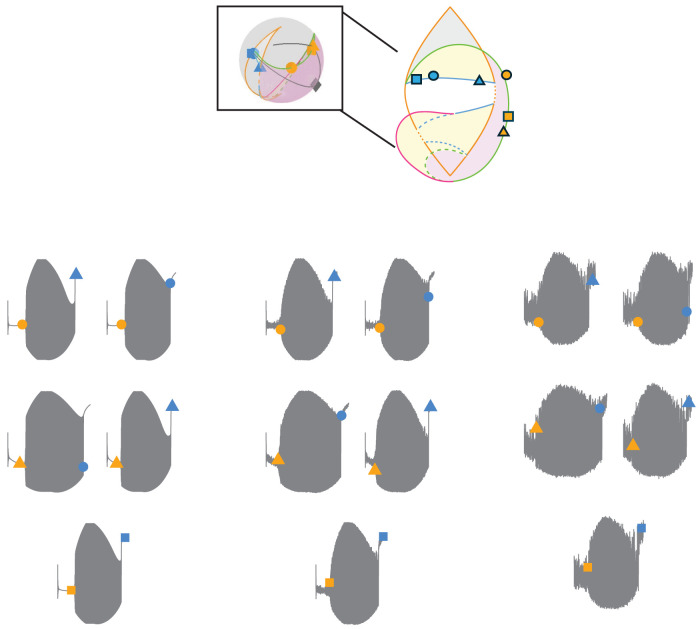
SupH/SH dynamotype trajectories. Similar to Figure 12, the bursting duration is not affected strongly by noise because it occurs in the pink region.

This dynamotype has a SupH onset bifurcation and a SH offset bifurcation, which creates increasing amplitude scaling at onset without a DC shift, then logarithmic frequency scaling at offset that may have a DC shift. The chosen trajectories all have a DC shift at offset (Fig. 15).

### Creating realistic synthetic seizures

**Figure 16.**
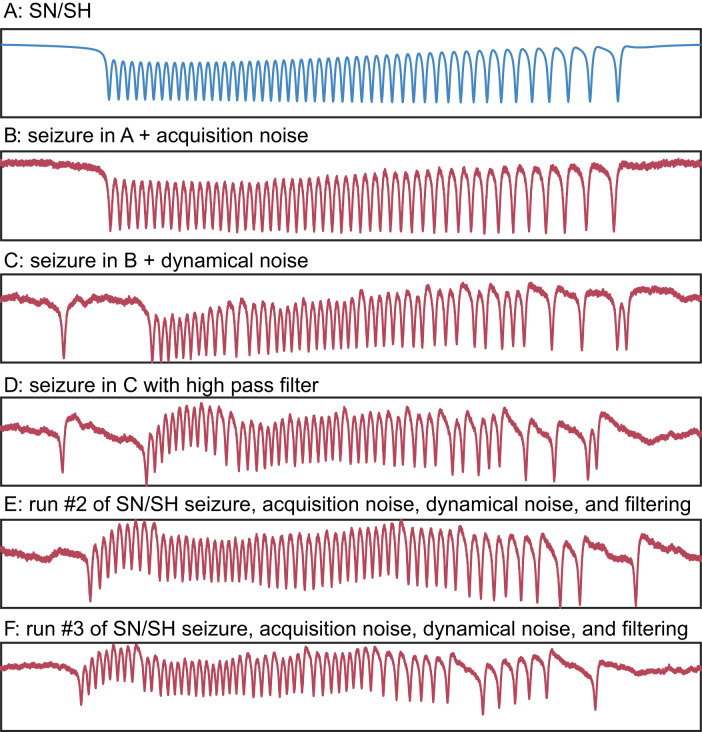
SN/SH dynamotype (***a***) generated with varying levels of acquisition noise (***b***), dynamical noise (***c***), and electrode filtering (***d***). Multiple runs with the same parameters (***d–f***) yield unique synthetic seizures, which have similar signal quality to clinically recorded human seizures. Axes are arbitrary units.

The methods above are capable of producing all 16 dynamotypes along several different pathways. This allows simulation of an arbitrarily large number of potential dynamical phenomena. However, the raw output of these models is not realistic—a trained electrophysiologist can easily spot that these simulated seizures are “too perfect” (Fig. 14*A*) and do not adequately portray seizures found in vivo. Three additional tools were necessary to transform these simulations into realistic seizures. The first step was adding pink noise to the signal, which would be expected in brain recordings due to the background neural activity. As seen in Figure 14*B*, pink noise adds background activity similar to standard EEG signals. However, the bursting activity of the seizure itself is still unreasonably ideal—it is very rare to see a seizure with such mathematical precision. We then add noise to the state variable *x* (Fig. 14*C*), which causes irregular stuttering in the spiking that is common in vivo. Finally, while DC shifts can be seen in some clinical systems, they are always filtered to some degree, typically lasting less than a few seconds (Ikeda et al., 1999; Saggio et al., 2020). Adding a high-pass filter similar to clinical systems reduces the length of the DC shift, causing the bursting to return to zero mean within the first eight spikes (Fig. 14*D*). As a demonstration of the utility of this tool, we also performed two more iterations with the same parameters and pathway (Fig. 14*E*,*F*), showing how it is straightforward to generate a library of seizures with similar dynamics. The result is a simulated seizure that has an appearance similar to human intracranial EEG. This final result can then serve as a template for one pathway of this particular dynamotype. Of note, all simulations in Figure 16 were generated and saved using in the provided GUI.

The create_database.mlx script is designed to generate a large and diverse dataset of simulated seizures by sweeping across onset and offset bifurcation curves for various dynamotype classes. It currently performs this sweep using .fit bifurcation paths for the hysteresis and slow-wave classes while holding key model parameters—alpha, k, k_fast, and dstar—constant. The script does not currently support sweeping over these parameters directly. For each point on the bifurcation curve, the simulation incorporates a low level of dynamical noise, with its value selected from prefitted .fit models based on the point's position along the bifurcation path.

There are several challenges and limitations to be aware of when considering future extensions that include parameter sweeps over k, k_fast, alpha, sigma, and dstar. First, the relationship between noise, seizure length, and path length is nontrivial: noise is typically proportional to seizure duration and trajectory length, meaning that higher noise levels can degrade the clarity and consistency of the simulated signal. In the hysteresis model, high noise levels in the bistable regime can introduce “stuttering” in both the seizure waveform and the *x*_3_ variable, which complicates detection and classification.

Additionally, varying the *k* parameter has a strong effect on seizure duration. Lower *k* values often result in prolonged seizures, which may exceed the default simulation time window—necessitating longer simulation durations to fully capture seizure termination. Finally, although the parameter values used are intentionally chosen, they are not random nor universally applicable. Many combinations of k, k_fast, alpha, and dstar will fail to generate valid seizures, so parameter tuning remains essential to ensure realistic and physiologically meaningful results. Sweeping over additional parameters such as k, k_fast, alpha, sigma, and dstar is not currently implemented in the script and is left to the user to design and implement, with the understanding that doing so will require careful parameter tuning and extended simulation durations to ensure valid and meaningful seizure dynamics.

## Discussion

This sample dataset of simulated seizures offers significant value across multiple domains, including ML, diagnostic tool development, and education. Large datasets are indispensable for training ML algorithms, and this work represents a toolbox to generate the first large-scale dataset of seizures labeled by onset and offset bifurcation. Generating simulated data provides a time- and cost-efficient solution to address the scarcity of bifurcation-labeled data. Simulated datasets can encompass extensive variability without the logistical and ethical challenges associated with clinical data collection.

Synthetic data have important strengths and weaknesses when used to train detection algorithms. One benefit is that the onset time is mathematically defined (in the hysteresis model when the *z* parameter is greater than zero, in the slow-wave and slow-wave piecewise models when the system has crossed an onset bifurcation), removing the uncertainties that exist in biological data and providing a gold standard when developing the algorithm. By incorporating noise and recording parameters, our model allows objective measurement of how those recording conditions affect the accuracy of the detector. The ability to produce large numbers of seizures provides important rigor for ML models, with the added benefit in this model that it can span multiple dynamotypes (most prior work has focused on a single model, which was a single dynamotype), multiple parameters, and over an arbitrary range of noise and recording conditions. This model thus provides the most comprehensive range of synthetic seizures yet published.

On the other hand, synthetic seizures have known weaknesses: human data are notoriously heterogeneous and may not be represented by the model. Amplitude, duration, and frequencies of the modeled data are arbitrary, so training algorithms must ignore specific values. This model does not include interictal patterns like spikes, high-frequency oscillations, and slowing. Finally, as with any model there is risk that they will misrepresent true physiology, leading to potential false positives or false negatives when the trained algorithms are applied to human data.

ML algorithms trained on a synthetic seizure dataset could detect and predict onset and offset bifurcations, enhancing the precision and reliability of diagnostic tools. Prior methods for classifying specific dynamotypes were based on manually selected features (Saggio et al., 2020), but physiological EEG is often very noisy and such classification is difficult. Modern ML tools are very powerful but require vast datasets, with realistic noise, to train. A major goal of this work is to provide a toolbox to create a dataset that can identify seizure onset time and dynamotype efficiently. With a large enough appropriate dataset, tools such as neural networks and reinforcement learning can learn which parts of the signals to ignore, a key aspect of interpreting EEG accurately. Another possible avenue to handle the noise in EEG is to train a model on simulated data and then fine-tune the model with a smaller labeled dataset of human EEG data. Improved diagnostic accuracy enables earlier interventions, which are critical for effective treatment. Additionally, simulated datasets are invaluable for medical education, providing students and professionals with exposure to diverse bifurcation dynamics, fostering enhanced diagnostic acumen and better preparation for clinical scenarios. Overall, this dataset advances the understanding, diagnosis, and treatment of epilepsy and related conditions.

Further variability in the dataset can be achieved by altering model parameters. For instance, in the hysteresis model, the parameter *d*∗ plays a pivotal role in determining system behavior. When *d*∗ ≤ 0, the system remains in a fixed point, precluding bursting activity. For small positive *d*∗, bursting emerges; with an increasing *d*∗, the ratio between burst and rest durations grows, transitioning to purely oscillatory behavior for sufficiently large *d*∗. Similarly, in the hysteresis and slow-wave models, parameters *k*, *d*∗, and *c* can be modified to influence oscillatory patterns. For instance, *c*, which controls path traversal speed, affects the number of oscillations per burst, with faster movements producing fewer oscillations. Path variability in the hysteresis and slow-wave implementations also hold potential for dataset expansion.

This model is adaptable to explore additional phenomena, such as the effects of stimulation on bifurcations, proximity to bifurcations, and seizure propagation dynamics.

Prior studies indicate that carefully timed stimulation effectively terminates seizures with a DC shift (SN bifurcation; Szuromi et al., 2023). However, further investigation could reveal whether specific types of stimulation are more effective for other onset bifurcations. For example, SubH onset bifurcations, which act as resonators, might require resonant frequency stimulation for termination.

One important limitation to this model is that it only simulates ictal activity. It does not include interictal spikes. It also does not simulate higher-order behavior such as sequences of oscillations during the ictal state, mixed mode oscillations, or bifurcations requiring more than two fast variables. Future work may incorporate such details, but the present work is primary focused on realistic first-order ictal data.

Perturbation analysis, as demonstrated in previous studies on electrical systems (Chow et al., 1990) and reservoirs (Heppell et al., 2000), could also be employed to probe the proximity of nearby bifurcations. Such approaches could elucidate the dynamics underlying seizure transitions. Furthermore, the type of bifurcation has been shown to influence seizure spread (Wang et al., 2011; Reimbayev and Belykh, 2014; Belykh et al., 2015). Exploring the relationship between the bifurcation type, spatiotemporal seizure organization, and brain network models (e.g., virtual epileptic patient; Jirsa et al., 2017; Proix et al., 2017) may yield insights with clinical translation potential. Understanding how dynamotype relates to seizure propagation and patient-specific modeling could provide a foundation for personalized therapeutic strategies.

## Synthesis

Reviewing Editor: Catherine Schevon, Columbia University Decisions are customarily a result of the Reviewing Editor and the peer reviewers coming together and discussing their recommendations until a consensus is reached. When revisions are invited, a fact-based synthesis statement explaining their decision and outlining what is needed to prepare a revision will be listed below. The following reviewer(s) agreed to reveal their identity: Theoden Netoff.

Both reviewers were in agreement regarding the strong contribution of the work, including the tutorial illustrating dynamical principles of seizure bifurcations based on prior work describing up to 16 "dynamotypes" to represent electroclinical seizures, the value of simulated seizures in fields such as seizure detection, and the GUI/simulation toolkit provided. They also agreed on several points that the authors need to address to strengthen the manuscript, particularly in the areas of biological realism, machine learning utility, and practical issues with the toolbox and associated database.

Major Comments:

1. The title "Dynamotypes for Dummies" is informal and somewhat misleading. The paper does not provide an in-depth exploration of the origins of dynamotypes. For instance, it does not delve into nullcline analysis, which is crucial for explaining the mechanisms of the bifurcations. A more accurate title would reflect the toolkit/tutorial focus rather than imply a comprehensive theoretical primer.

2. The manuscript lacks sufficient quantitative validation of the realism of simulated seizures. While power spectral density comparisons are presented, frequency content alone does not establish biological plausibility. Further analyses comparing real and synthetic seizures across broader statistical features (e.g., duration distributions, amplitude variance, temporal structure) would significantly strengthen the work.

3. The discussion refers to a large labeled dataset for machine learning purposes; however, no such dataset is actually provided. The code and tutorial allow the user to generate synthetic datasets, but the paper does not describe any dataset or validate machine learning applications trained on them. Claims regarding ML algorithm training and prediction enhancement are speculative and should be revised or supported by a concrete example.

4. It seems that the primary goal of this toolbox is to establish a platform for generating a variety of well-controlled, but realistic-looking EEG for training seizure detection algorithms. Synthetic data such as this has a well-defined seizure onset time (presumably when crossing bifurcation, though it would be helpful to spell this out in the intro). It would be helpful to elaborate some on the advantages of using synthetic data for training such algorithms (e.g. it is possible to determine how recording conditions such as noise and bandwidth may or may not affect detection accuracy). It would also be useful to highlight potential problems with using synthetic data to train algorithms (e.g. without including non-seizure activity like sharp waves, interictal spikes, etc, algorithms trained on synthetic data may be prone to false positives).

5. The discussion implies that knowing the onset bifurcation type might help in the selection of treatment (e.g. for a stimulation paradigm). How does this toolbox help identify bifurcation type from patient data? Can it be used to train an algorithm that would infer bifurcation type from recorded EEG?

6. This tutorial is intended to be a "for dummies" primer on dynamotypes. The text throughout is generally very clearly written in a digestable manner. However, there are some places wherein the manuscript could be more user-friendly by starting from the basics. For example, somewhere early in the Methods section, it may be helpful to visually take the reader through a simplified example of seizure onset and/or offset (possibly in 2D state space?) before diving into a spherical projection of the map with 6 different types of bifurcation shown.

7. Related to above, it would be useful to include a bit more text describing that the models presented here are not biophysical models per se, but rather they are descriptive/phenomenological models. An epilepsy researcher that is relatively new to dynamical models may be inclined to attach the variables u and v directly to some physical process.

8. The tutorial scripts were seemingly useful, but had some limitations that precluded me from using them fully. The Matlab Live Script version, which appeared to be the most instructive format, required the Symbolic Math Toolbox, which I do not have and thus could not run. The python version ran, but was significantly less interactive/instructive.

9. The GUI tools were very useful for generating data under different recording conditions. 3 examples are provided. How would these be modified for additional dynamotypes?

10. The "save simulation" button in the GUI produces invalid .mat filenames that appear to include multiple strings. Below is one example. Perhaps, instead of saving all of the parameters in the filename, they could be saved as a variable in the .mat file?

"SN_SH_timeseries_Offset_index_1_Onset_index_4_dstar_0.50_k_0.00_dynamical_noise_0.00_tMax_500.00_adjusted_frequency_30.00_electrode_drift_filter_20.00.matSN_SH_timeseries_Offset_index_0_Onset_index_"

Minor Comments:

In Figure 1, the table is helpful but it is unclear how the Subcritical Hopf and Saddle Node LCb are differentiated. Clearer labeling of LCs and LCb in both onset and offset would aid interpretation.

Figures 3, 4, and to some extent 2 and 7 are of low resolution and not publication-quality. Figure 4 would benefit from higher contrast to highlight bursting paths.

The noise implementation is a welcome feature, but the statistical properties of the noise (e.g., interval distributions) are not validated. Figure 7's "Scaled Power" axis is unclear-if representing pink noise, the slope should follow a 1/f decay. Instead, the power only minimally decays from 0.1 to 100 Hz, which is inconsistent.

In Figure 7, the legend incorrectly labels black as noisy and red as raw; this should be reversed.

Figure 8 should include axis labels to make comparisons of DC shift attenuation quantifiable. The same applies to Figures 14C and D, which are stated to differ in high-pass filtering but appear nearly identical visually.

The extended data was attached in a variety of formats, some of which did not appear correctly. In the docx version of the "tutorial", tables were cutoff and unreadable.

## Author Response

Both reviewers were in agreement regarding the strong contribution of the work, including the tutorial illustrating dynamical principles of seizure bifurcations based on prior work describing up to 16 "dynamotypes" to represent electroclinical seizures, the value of simulated seizures in fields such as seizure detection, and the GUI/simulation toolkit provided. They also agreed on several points that the authors need to address to strengthen the manuscript, particularly in the areas of biological realism, machine learning utility, and practical issues with the toolbox and associated database.

Major Comments:

1. The title "Dynamotypes for Dummies" is informal and somewhat misleading. The paper does not provide an in-depth exploration of the origins of dynamotypes. For instance, it does not delve into nullcline analysis, which is crucial for explaining the mechanisms of the bifurcations. A more accurate title would reflect the toolkit/tutorial focus rather than imply a comprehensive theoretical primer.

We agree that the main paper did not have a detailed description of dynamical theory, which makes the title misleading. Indeed, our goal was to provide a theoretical primer, albeit not a comprehensive one (which is infeasible). However, we do feel there is a need for a 'Dynamotypes for Dummies' manuscript, and so have made many changes to justify this title and improve the paper. We have several responses to this.

A: It was the Matlab Live script that contained the tutorial and in-depth explanation of the origins of Dynamotypes, not the main paper. Although admittedly confusing, that script was the "Dynamotypes for Dummies", and the main text merely introduces it. The underlying theory is extensive, starting from Izhikevich to Golubitsky to Saggio (J Math Neurosci) to Saggio (eLife). In total, the citations comprise literally hundreds of pages of background theory. The tutorial guides the reader through these and other selections "if the reader would like to understand the theory behind the construction of the model". We do not think it is feasible to explain all of this background in a single article. In addition, because this study of dynamics is best understood by seeing the dynamics, we wrote it into a Live editor, so changes can be visualized within Matlab instantaneously.

B: However, we agree that even the Matlab script was not extensive. For a full "for Dummies" tutorial, it is certainly helpful to have a better description of the basics, including ODEs and nullclines. We have added this to the main paper and the Matlab Script. These are in Section A and B of the main paper.

C: We believe that with these changes, our tutorial now does present enough information to deserve the "for Dummies" title. Short of writing another book like Izhikevich, the most feasible method of teaching these concepts is to provide a "garden path" through past literature, as we do. We also point out that the genesis of this paper is literally to accomplish precisely what we claim: members of our lab wrote and refined this tutorial as a way of teaching incoming students how to implement those models. It has been successful multiple times, albeit requiring the students to do extra reading. This success led us to desire to share it with others.

2. The manuscript lacks sufficient quantitative validation of the realism of simulated seizures. While power spectral density comparisons are presented, frequency content alone does not establish biological plausibility. Further analyses comparing real and synthetic seizures across broader statistical features (e.g., duration distributions, amplitude variance, temporal structure) would significantly strengthen the work.

Thank you for the thoughtful feedback. The question of realism is indeed a major goal of this work. However, "quantitative validation" of realism is not straightforward. In fact, due to the essentially infinite variability of real seizures, we respectfully argue that the key aspect of a realistic seizure model is not that it "match" a list of collected seizures (which can never comprise the full range of biological plausibility), but rather that it have the capacity to produce a comprehensive, broad range of parameters that can be manipulated and scaled to match any arbitrary seizure. Our approach satisfies this latter method.

There are two primary methods for comparing realism. The first is matching the objective parameters: amplitude, duration, peak frequency, spectrograms, etc. The second has traditionally been to match the visual appearance. Past attempts at this second method were often completely subjective, and it was not until the work of Perucca et al 2014 and Lagarde et al. 2019 that more systematic methods were proposed. In effect, this second method is focused on capturing (objectively and visually) the dynamical signature of the seizure, but there was no ground truth to guide those methods. Saggio et al. 2020 was the first work to perform this task from a scientific approach rather than a purely visual one. The idea of dynamotypes is so appealing because it reconciles the visual approaches while also providing dynamical insight. That description was explored in great detail in Saggio 2020, and was a motivation for our current work.

As for the question of matching objective parameters with real seizures, we must clarify a critical aspect of our model. All parameters (amplitude, frequency, duration) are simply scaling parameters in the model, and can assume any arbitrary value. The model can easily be set to a specific amplitude or frequency. The duration can also be controlled by the speed at which the system traverses the seizure regime using the slow variable z, which is also a parameter. Amplitude itself is somewhat arbitrary in EEG due to its dependence on 1/r^2 from Coulomb's law, and the signal to noise ratio is a parameter that we implicitly vary (i.e. the low, medium, and high levels of noise in the Atlas). In other words, our method implicitly allows the user to match the amplitude, duration, and frequency of our model with real seizures. This idea of arbitrary scaling is paramount, because it means we do not have to compare scale directly and can just focus on the appearance when using the model output.

However, we did not explain this key aspect. We apologize for not making this clear in the paper.

In addition, we also point out that, to our knowledge, ours is the first model to incorporate noise and filtering effects to the output. These effects are absolutely critical to making the seizures look 'real', but are also difficult to quantify except with power spectra and visually. In our clinical opinion (epilepsy clinicians are on our team), this model successfully captures both effects to the point that we are unable to distinguish model from reality in the noisy simulations-many of these simulations look like seizures in a way that we have not previously seen in simulations. Although we did not specifically state so, our intention was that biological plausibility would be self-evident by observing the noisy simulations in the Atlas. Provided we explain that scaling is arbitrary and can be adjusted to specific data (which we did not adequately do), we believe these patterns not only span that range of plausibility, we also have never previously seen seizure simulations that look so similar to reality (but only after adding noise and filtering effects).

Moreover, by enabling extensive parameter sweeps, the model mitigates the risk of machine learning models overfitting to specific patients or limited training distributions. Rather than over-relying on sparse or biased clinical datasets, researchers can use this tool to create a broad, balanced training corpora that spans a wide space of seizure morphologies and dynamics. For these reasons, we believe the current level of validation is appropriate given the intended use of the model, and that its flexibility and scalability provide a practical, efficient, and biologically informed solution to current gaps in seizure detection and classification.

We have made the following changes in the paper to address this concern:

1. We have added two parameters to the model, k_fast, a parameter that controls the number of oscillations in a burst (which directly affects the duration), and alpha, a parameter that controls the amplitude of the seizure. Modifying these parameters, the simulation can be flexibly modified to produce any amplitude or duration. This demonstrates how the results can be scaled to arbitrary levels and allows users to replicate a wide range of human EEG patterns that resemble the diversity seen in clinical data.

2. Based on later concerns, we have made several updates to describe how to customize the simulations better. A direct consequence of this is that the output can be arbitrarily scaled for amplitude, frequency, and duration. We also describe how SNR is an adjustable parameter.

3. We have added a new section F to the Methods:

F: Scaling output to physiological data The simulated seizures generated by our model are not intended to be exact replicas of all human seizure activity, but rather to capture key biological features that are most relevant for seizure onset detection and onset/offset bifurcation classification. The model's flexible parameterization enables users to generate a wide range of seizure morphologies observed in clinical EEG. While one method to treat such data is to use normalized scales and treat the dynamics independent of scale, it is also desirable to be able to match the synthetic seizures with physiological data. This model allows for arbitrary scaling that can be matched to data of interest. Amplitude can be modified by a parameter alpha in the model, and furthermore, it is fully user-defined, acknowledging the variability introduced by differences in hardware, electrode placement, and normalization practices. Duration is controllable via the k and k_fast parameters and choice of dynamical path. The signal to noise ratio (relative amplitude between the bursting and the noise signal) is a parameter that can be set to any arbitrary level. Moreover, the model's bifurcation-based structure imposes biologically meaningful temporal patterns at onset and offset, which resemble the electrophysiological transitions seen in real seizures. This framework also allows for systematic generation of underrepresented seizure types, including slow-amplitude growth (e.g., supercritical Hopf onset), DC shifts with varying degrees of recovery to baseline (via post processing high-pass filtering), and SNIC bifurcation onsets that are rare in focal epilepsy {Saggio, 2020 #1821}. These edge cases are commonly missed by traditional machine learning models due to limited labeled data. Our simulation platform overcomes this limitation by enabling large-scale, parameter-swept generation of diverse synthetic seizures, thus mitigating overfitting and improving generalization across patient populations. While exhaustive biological replication is not the goal, the model's design prioritizes flexibility, interpretability, and practical utility in addressing gaps in seizure detection and classification.

3. The discussion refers to a large labeled dataset for machine learning purposes; however, no such dataset is actually provided. The code and tutorial allow the user to generate synthetic datasets, but the paper does not describe any dataset or validate machine learning applications trained on them. Claims regarding ML algorithm training and prediction enhancement are speculative and should be revised or supported by a concrete example.

We agree with this comment. There are two aspects of this concern we need to address.

First, we did not make it clear in the paper that the github contained the seizure_atlas.mat file for the 330 seizures in the Atlas. We have corrected this in the paper:

4. Dynamotype Atlas: We present an atlas showing multiple examples of each of the 16 dynamotypes, along with generative MATLAB code to replicate all atlas figures. These files are on the Github repository, and a copy of the Atlas is saved as Extended Data 2.

5. Database of Atlas Seizures. The 330 seizures from the Atlas are saved in a file 'seizure_atlas.mat' on the Github repository.

Second, we agree that 330 example seizures does not constitute a 'large labeled dataset' in the traditional machine learning sense. We have amended the language in the paper to reflect this. Rather than providing a static dataset, our goal is to offer a flexible generative framework. To this end, we have now added a script-create_database.mlx-to the GitHub repository. This script shows users how to generate large and diverse synthetic seizure datasets by sweeping across bifurcation paths. Through this script, users can modify model parameters to produce a wide variety of synthetic seizures, including examples with increasing amplitude, variable DC baseline shifts, and low-voltage fast activity. While we believe this simulation tool could help address known shortcomings in current seizure datasets and facilitate machine learning applications, we acknowledge that we have not yet conducted formal validation or training experiments, and therefore refrain from making definitive claims about model performance. Our next work using this method will be to generated many thousands of seizures for each dynamotype for this purpose. The discussion now reads:

Machine learning algorithms trained on a synthetic seizure dataset could detect and predict onset and offset bifurcations, enhancing the precision and reliability of diagnostic tools. Prior methods for classifying specific dynamotypes were based on manually-selected features (Saggio et al., 2020), but physiological EEG is often very noisy and such classification is difficult. Modern machine learning tools are very powerful but require vast datasets, with realistic noise, to train. A major goal of this work is to provide a toolbox to create a dataset that can identify seizure onset time and dynamotype efficiently. With a large enough appropriate dataset, tools such as neural networks and reinforcement learning can learn which parts of the signals to ignore, a key aspect of interpreting EEG accurately.

We also added a new section to the github with this script, and point to it in the beginning of the paper:

7. Matlab script 'create_database.mlx' to generate a wide array of simulated seizures for each class, which can be adapted to produce larger datasets.

4. It seems that the primary goal of this toolbox is to establish a platform for generating a variety of well-controlled, but realistic-looking EEG for training seizure detection algorithms. Synthetic data such as this has a well-defined seizure onset time (presumably when crossing bifurcation, though it would be helpful to spell this out in the intro). It would be helpful to elaborate some on the advantages of using synthetic data for training such algorithms (e.g. it is possible to determine how recording conditions such as noise and bandwidth may or may not affect detection accuracy). It would also be useful to highlight potential problems with using synthetic data to train algorithms (e.g. without including non-seizure activity like sharp waves, interictal spikes, etc, algorithms trained on synthetic data may be prone to false positives).

We have added this excellent suggestion to the results:

Synthetic data has important strengths and weaknesses when used to train detection algorithms. One benefit is that the onset time is mathematically defined (in the hysteresis model when the z parameter is greater than zero, in the slow-wave and slow-wave piecewise models when the system has crossed an onset bifurcation), removing the uncertainties that exist in biological data and providing a gold standard when developing the algorithm. By incorporating noise and recording parameters, our model allows objective measurement of how those recording conditions affect the accuracy of the detector. The ability to produce large numbers of seizures provides important rigor for machine learning models, with the added benefit in this model that it can span multiple dynamotypes (most prior work has focused on a single model, which was a single dynamotype), multiple parameters, and over an arbitrary range of noise and recording conditions. This model thus provides the most comprehensive range of synthetic seizures yet published.

On the other hand, synthetic seizures have known weaknesses: human data are notoriously heterogeneous and may not be represented by the model. Amplitude, duration, and frequencies of the modeled data are arbitrary, so training algorithms must ignore specific values. This model does not include interictal patterns like spikes, high frequency oscillations, and slowing. Finally, as with any model there is risk that they will misrepresent true physiology, leading to potential false positives or false negatives when the trained algorithms are applied to human data.

5. The discussion implies that knowing the onset bifurcation type might help in the selection of treatment (e.g. for a stimulation paradigm). How does this toolbox help identify bifurcation type from patient data? Can it be used to train an algorithm that would infer bifurcation type from recorded EEG? The reviewer is correct that identifying the bifurcation type from recorded EEG is a potential goal of using these data. We are currently developing methods to do so but they are beyond the scope of this work. But this is not a new idea, as at least three labs have already done this. In short, the most straightforward method is using supervised features focusing on DC shift (Saddle Node), change in amplitude (Supercritical Hopf), and change in frequency (SNIC). That method has been used in three previous works identifying bifurcation type (e.g. Saggio et al eLife 2020, https://www.biorxiv.org/content/10.1101/2023.04.02.535246v1 https://www.biorxiv.org/content/10.1101/2025.02.12.637999v1). All three of those studies have already developed early detection algorithms that are applied to 'real' EEG, but are limited because the number of seizures was small and they had to resort to supervised features. One goal of our paper is to introduce a model that can move the field forward by providing hundreds of thousands of synthetic seizures to train machine learning algorithms. With such a rich dataset, there are several modern methods (e.g. neural networks, reinforcement learning) that can learn how to infer bifurcation type and how to 'see through' noise. That field is moving forward quickly in other disciplines, and we need a model to apply those tools to epilepsy. However, it is critical that those tools see "noisy, realistic" data, which is a primary outcome of this work.

In response to this, we have added the following to the Discussion.

Machine learning algorithms trained on a synthetic seizure dataset could detect and predict onset and offset bifurcations, enhancing the precision and reliability of diagnostic tools. Prior methods for classifying specific dynamotypes were based on manually-selected features (Saggio et al., 2020), but physiological EEG is often very noisy and such classification is difficult. Modern machine learning tools are very powerful but require vast datasets, with realistic noise, to train. A major goal of this work is to provide a toolbox to create a dataset that can identify seizure onset time and dynamotype efficiently. With a large enough appropriate dataset, tools such as neural networks and reinforcement learning can learn which parts of the signals to ignore, a key aspect of interpreting EEG accurately. Another possible avenue to handle the noise in EEG is to train a model on simulated data, then fine tune the model with a smaller labeled dataset of human EEG data. Improved diagnostic accuracy enables earlier interventions, which are critical for effective treatment. Additionally, simulated datasets are invaluable for medical education, providing students and professionals with exposure to diverse bifurcation dynamics, fostering enhanced diagnostic acumen and better preparation for clinical scenarios. Overall, this dataset advances the understanding, diagnosis, and treatment of epilepsy and related conditions.

6. This tutorial is intended to be a "for dummies" primer on dynamotypes. The text throughout is generally very clearly written in a digestible manner. However, there are some places wherein the manuscript could be more user-friendly by starting from the basics. For example, somewhere early in the Methods section, it may be helpful to visually take the reader through a simplified example of seizure onset and/or offset (possibly in 2D state space?) before diving into a spherical projection of the map with 6 different types of bifurcation shown.

Thank you for this suggestion, we agree this would be very helpful in clarifying how to use the model. Along with item #1, we have made extensive changes to the Methods section to make this a more instructive "for dummies" primer. Directly related to this question, we have added Figure 5, a figure that shows a simplified example of a seizure onset and offset alongside a path in 2D space. It is a great segue to Fig. 6 that shows several additional dynamotype paths.

7. Related to above, it would be useful to include a bit more text describing that the models presented here are not biophysical models per se, but rather they are descriptive/phenomenological models. An epilepsy researcher that is relatively new to dynamical models may be inclined to attach the variables u and v directly to some physical process.

We have amended section II.A. in Methods to introduce more about phenomenological models and these parameters:

There are several approaches to model seizures (Wendling et al., 2015), ranging from highly physiological models that include structure and specific channels (Traub et al., 2005), to mean field approximations that generalize the bulk effects of excitation and inhibition (Demont-Guignard et al., 2012), to dynamical descriptions that focus on how certain behaviors can be produced, agnostic to specific mechanisms (Saggio and Jirsa, 2023). Our work uses the latter approach, as introduced in (Saggio et al., 2017). This model is phenomenological: it aims to use the simplest mathematical expression able to reproduce some relevant dynamics hypothesized to underlie seizures. It is important to stress that variables and parameters in phenomenological models are abstract and do not have an obvious biophysical correlate other than one of the variables reproducing the voltage timeseries. This type of phenomenological approach has been often been used to build large-scale brain models due to their lower computational burden (Hutchings et al., 2015; Sinha et al., 2017; Lopes et al., 2020; Hashemi et al., 2021).

As described extensively in prior work (Jirsa et al., 2014; Saggio et al., 2017; Saggio et al., 2020), the underlying phenomenon guiding this model is that seizures are defined by abrupt transitions in dynamics. In the field of dynamical systems, this type of transition between two states requires proximity to a bifurcation (Izhikevich, 2010). The Saggio-Jirsa model (Saggio et al., 2017) assumes that a seizure's onset and offset are due to the system going through such bifurcations (see the Supplementary material of (Saggio et al., 2020) for how to use the model for input-induced transitions, another proposed mechanism for transitions). A feature of this approach is that it is independent of specific mechanism-the bifurcations are invariant and preserved across mechanisms, species, etiologies (Jirsa et al., 2014).

8. The tutorial scripts were seemingly useful, but had some limitations that precluded me from using them fully. The Matlab Live Script version, which appeared to be the most instructive format, required the Symbolic Math Toolbox, which I do not have and thus could not run. The python version ran, but was significantly less interactive/instructive.

We sincerely apologize-we caught this dependency error in the GUIs (we hope-it appears they were not a problem) but missed it in the Matlab Live. We have corrected this. This is especially important because, as we describe in #1, that script is the primary tutorial and it needs to be usable.

9. The GUI tools were very useful for generating data under different recording conditions. 3 examples are provided. How would these be modified for additional dynamotypes? We appreciate the feedback. In response, we have added both a tutorial video and a written document that walk through the specific steps required to modify the GUI script in order to implement an additional dynamotype. These resources are designed to clearly outline which parameters and functions need to be adjusted, making it easier for users to extend the tool to support new dynamotypes.

10. The "save simulation" button in the GUI produces invalid .mat filenames that appear to include multiple strings. Below is one example. Perhaps, instead of saving all of the parameters in the filename, they could be saved as a variable in the .mat file? "SN_SH_timeseries_Offset_index_1_Onset_index_4_dstar_0.50_k_0.00_dynamical_noise_0.00_tMax_500.00_adjusted_frequency_30.00_electrode_drift_filter_20.00.matSN_SH_timeseries_Offset_index_0_Onset_index_" We sincerely apologize- we have streamlined the filename structure greatly by removing the names of the parameters. The simulation now saves the class title and the numerical values of the parameters, "Class_xx_xx_xx_xx_xx_xx_xx.mat" and saves a string of all the parameter names in the .mat file. It was necessary to have a unique file name so that subsequent saves would not overwrite the previous one.

Minor Comments:

11. In Figure 1, the table is helpful but it is unclear how the Subcritical Hopf and Saddle Node LCb are differentiated. Clearer labeling of LCs and LCb in both onset and offset would aid interpretation.

We have changed the labels in the figures (Now Figure 1 and Figure 2) to make this more clear. We have also updated the text to point out that SubH and SN LCb are not mathematically distinguishable (both have arbitrary scaling). The same is true for SN LCb and FLC offsets. That aspect of classification was previously discussed in Saggio et al 2020.

II. A.

Note that the SN (LCb) and SubH onsets, as well as the SN (LCb) and FLC offsets, all have arbitrary scaling and thus cannot be distinguished from each other in the time series data.

12. Figures 3, 4, and to some extent 2 and 7 are of low resolution and not publication-quality. Figure 4 would benefit from higher contrast to highlight bursting paths.

We have submitted all figures as vector format. The Word document cannot handle the higher resolution so we inserted lower resolution as placeholder.

13. The noise implementation is a welcome feature, but the statistical properties of the noise (e.g., interval distributions) are not validated. Figure 7's "Scaled Power" axis is unclear-if representing pink noise, the slope should follow a 1/f decay. Instead, the power only minimally decays from 0.1 to 100 Hz, which is inconsistent.

While we appreciate the reviewer's attention to the spectral characteristics of the noise, we note that the baseline trace (Figure 9, which was previously Fig. 7, left) clearly demonstrates a 1/f decay consistent with pink noise, confirming that the implementation aligns with theoretical expectations in the absence of seizure dynamics. In contrast, during seizure periods (Figure 9 (previously 7), right), the dynamics of the seizure dominate the spectrum, naturally overriding the expected 1/f decay within the 1-30 Hz band. This is to be expected, as seizure activity introduces structured rhythmicity and amplitude modulation that diverge from purely stochastic noise. Note also that since the raw signal already has some power at higher frequencies, the introduction of pink noise increases the amount of spectral power at the higher frequencies, so the slope of 1/f^b is less steep when noise is present. That effect is clearly shown in Fig. 9. We have now clarified this distinction.

As for validating the interval distributions, the specific properties of the noise, like amplitude, frequency, and duration, are tunable parameters of our model and can be scaled arbitrarily as described in response #2. We now state that the noise "Background noise can be added using the toolbox function add_pink_noise() to match physiological data". Note that this function in the toolbox explains how to change the parameters for magnitude and spectral characteristics (the 'b' in the 1/f^b) in the code.

14. In Figure 7, the legend incorrectly labels black as noisy and red as raw; this should be reversed.

This has been corrected, thank you. This figure is now Fig. 9.

15. Figure 8 should include axis labels to make comparisons of DC shift attenuation quantifiable. The same applies to Figures 14C and D, which are stated to differ in high-pass filtering but appear nearly identical visually.

These concerns relate back to the issue of arbitrary scaling in the model, discussed in response #2. We adjusted noise values to be a percentage of the amplitude and frequency of the spike discharges, which resulted in noise that is very similar to clinical systems. We have added axis labels to Fig. 8 (now Fig. 10), although the precise scale of the model data is arbitrary and can be adjusted to fit the human data-this is a strength of our model.

Regarding Fig 14 (now Fig. 16), we did not add labels since the amplitude and duration of both signal and noise are purely arbitrary units in this simulation-only figure, which is meant to be purely qualitative. We state in the caption now that "axes are arbitrary units." The difference between C and D is subtle but very important: this is a SN onset with a DC shift, and the shift is maintained at seizure onset in C. However, it detrends in D under the word "pass" in the figure, abolishing the DC shift. Why this is so critical is that DC shift is very important clinically as well as dynamically, but EEG systems filter it out. The point of this figure is to show that simple HP filtering, as is expected in EEG systems, can make DC shifts look shorter. We have seen innumerable seizure onsets in human data that look exactly like Figure D, and an important feature of our method is that we can explain why it happens purely using technical parameters. To point out this effect, we have altered the text to say "Adding a high pass filter similar to clinical systems reduces the length of the DC shift, causing the bursting to return to zero mean within the first 8 spikes (Fig. 16D)." 16.The extended data was attached in a variety of formats, some of which did not appear correctly. In the docx version of the "tutorial", tables were cutoff and unreadable.

We have corrected this. The challenge was that we wanted to provide a copy of the tutorial as a Word document and of the Atlas as a PDF. Unfortunately eNeuro will not allow submission of PDF files, so it had to be saved as a zip file. Note that these files are also available in the github, but we felt it was reader-friendly to allow direct download without going to github of these two critical documents.
