# Supplementary material for: Dynamotypes for Dummies: A Toolbox, Atlas, and Tutorial for Simulating a Comprehensive Range of Realistic Synthetic Seizures
Source: eNeuro. 2025 Oct 15;12(10):ENEURO.0200-25.2025. doi: 10.1523/ENEURO.0200-25.2025 (PMC12549069; doi:10.1523/ENEURO.0200-25.2025)
Supplement: Data 1 — Microsoft Word and PDF versions of the Matlab Live “Dynamotypes for Dummies Tutorial.” Download Data 1, ZIP file. [file eneuro-12-ENEURO.0200-25.2025-s002.zip › Dynamotypes_for_dummies_tutorial.docx]

Dynamotypes for Dummies Model Walkthrough

Matlab Live Editor for **Dynamotypes for Dummies: A toolbox, atlas, and tutorial for simulating a comprehensive range of realistic synthetic seizures**

Christina Sheckler, Kathleen Kish, Zion Walker, Grant Barkelew, Matt Szuromi, Marisa Saggio, Dakota Crisp, William Stacey

Contact info: William Stacey ([William.stacey@umich.edu](mailto:William.stacey@umich.edu)), Christina Sheckler (ninashec@umich.edu), Kathleen Kish (katfin@umich.edu)

clear all

addpath('Walkthrough helper files');

# *Literature*

Before we give a brief walkthrough of the model, we would like to list out resources that may be useful in learning the ins and outs of the model. We understand that people interested in the model may have varying degrees of mathematical proficiency. While it is not necessary to have a deep rigorous understanding of all the math involved in the construction and employment of this model, we do think that having some experience with differential equations and linear algebra will greatly improve the reader's ability to understand and utilize the model.

Here is the list of useful materials:

Using unfoldings of high codimension singularities to model fast-slow bursters (i.e. the approach used in this model):

- Bertram, Richard, et al. "Topological and phenomenological classification of bursting oscillations." *Bulletin of mathematical biology* 57.3 (1995): 413-439. **Uses these unfoldings for bursting**
- Izhikevich, Eugene. "Neural Excitability, Spiking, and Bursting." 2000. **A very accessible introduction to bursting, uses some unfoldings; establishes the taxonomy of fast-slow bursters**
- Golubitsky, Martin, Kresimir Josic, and Tasso J. Kaper. "An unfolding theory approach to bursting in fast–slow systems." *Global analysis of dynamical systems*. CRC Press, 2001. 282-313. **Formalizes and extends the approach of Bertram et al**
- Saggio, Maria Luisa et al. "Fast-Slow Bursting in the Unfolding of a High Codimension Singularity and the Ultra-Slow Transitions of Classes." 2017. **Systematically extends the work of Golubitsky et al to codimension 3 and proposes models for all classes of Izhikevich’s taxonomy (planar only)**

Using fast-slow bursters to classify and model seizures

- Jirsa, Viktor et al. "On the Nature of Seizure Dynamics." 2014. **Extends Izhikevich taxonomy to seizures and proposes one type of burster to model the most common seizure type**
- Saggio, Maria Luisa et al. "A Taxonomy of Seizure Dynamotypes." 2020. **Extends the approach of Jirsa et al to the full (planar) taxonomy, using the model in the Saggio et al 2017**
- Crisp, Dakota et al. "Quantifying Epileptogenesis in Rats with Spontaneous and Responsive Brain State Dynamics." 2020. **Application of the taxonomy**
- Saggio, Maria Luisa & Jirsa, Viktor. "Phenomenological Mesoscopic Models for Seizure Activity." 2022. **A review**
- Depannemaecker, Damien et al. "A unified physiological framework of transitions between seizures, sustained ictal activity and depolarization block at the single neuron level." 2022. **It also maps a new neural model to the unfolding used in Saggio et al 2017**
- Szuromi, Matthew et. al. "Optimization of Ictal Aborting Stimulation Using the Dynamotype Taxonomy." *2023.* **Application of the model to stimulation**

If the reader is new to nonlinear dynamics, we highly suggest they start by reading Strogatz, Steven. *Nonlinear Dynamics and Chaos.*. Particularly, Parts I & II of the book. Part III is on chaos and does not apply. If one is in a rush, they could probably skip all of Chapter 4 and Sections 3.6-3.7, 6.5-6.8, 7.2-7.6, and 8.6-8.7.

Next, we suggest reading Izhikevich (2000), Jirsa (2014), Saggio (2017), and Saggio (2020) in chronological order. You could skip Izhikevich, but we think even a cursory reading helps set the stage well for the following papers. It also gives great visualizations.

The other papers are supplementary and discuss a range of uses and applications of the model. These may be interesting depending on what the reader intends to use the model for.

Finally, if the reader would like to understand the theory behind the construction of the model in Saggio (2017), they should read Bertram and Golubitsky.

# *Brief introduction to dynamical theory*

Dynamical systems are systems whose state changes over time according to a defined rule. In the context of biological modeling, dynamical systems are most often realized in two forms: discrete maps and differential equations. Differential equations can also be ordinary or partial. For this work, we utilize ordinary differential equations (ODEs), to which we will restrict this introduction.

First-order ODEs consist of equations which relate the time derivative of an indpendent state variable (which may be vector valued) to the current state of the system and possibly external time-dependent forcing. Our core model contains no external forcing. Then general form is then:


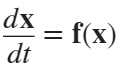


Given starting values for the components of the state variable, known as the initial conditions, the solutions of the ODEs prescribe a unique trajectory that describes the evolution of the state variables, beginning at the initial condition. Explicit solutions to ODEs are highly desirable as they tell us the exact value of the state variable at every time point. However, ODEs can be complex and their solutions highly nontrivial. This motivates the so-called qualitative study of ODEs.

### Long-Term Behavior, Attractors, and Stability

The qualitative theory of ODEs is concerned with describing the long-term behavior of the dynamical system. Does the system "settle down" at a certain value? Does it persistently oscillate? Does it exhibit chaos? These questions motivate the first term of interest, a **stable** **attractor.** Attractors are most generally understood as a subset of possible values of the state variable towards which the system evolves. The attractor is stable in the since that, given a trajectory initialized on the attractor, a sufficently small, arbitrary perturbation away from the attractor results in the system returning to the attractor. Conversely, a set of states may be **unstable**, in which case small perturbations result in trajectories which flow away from that set. In this case, we may also call this a **repeller.**

The two types of attractors pertinent to this work are **stable fixed points** and **stable limit cycles**. So-called chaotic or strange attractors are explicitly dissallowed in our model, as they only exist in systems of 3 or more dimensions, while our fast-subsystem is only 2D. Further, degenerate solutions like line attractors or centers are not relevent to the present work.

First, fixed points are single states at which the system remains for all time when intitialized there. Mathematically, these are disconnected points at which
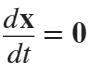
. Thus, fixed points are solutions of
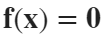
. Writing this in components
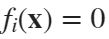
, for all components
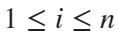
. For a single fixed
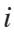
, the set of states that solves
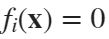
 is called a **nullcline.** Thus, fixed points are the intersections of the
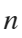
 nullclines. Fixed points can be either stable, unstable or **saddles**. Stability of a fixed point is assessed by evaluating the **Jacobian matrix** at the fixed point. The Jacobian is given by


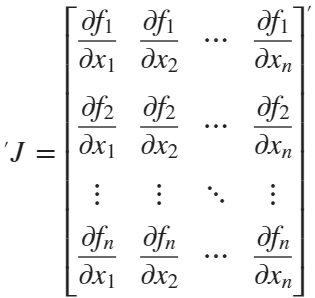


The Jacobian evaluated at the fixed point describes the growth or decay of small perturbation. A fixed point is stable when the real parts of the **eigenvalues** of the Jacobian are all negative. A fixed point is unstable if the real parts of all eigenvalues are positive. A fixed point with a mix of eigenvalues with positive and negative real parts is a saddle. If the eigenvalues are all real, the fixed point is known as **nodes**. If eigenvalues are complex, then we call the fixed point a **spiral**.

Limit cycles are periodic solutions of ODEs,
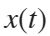
, such that
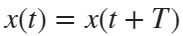
 for some finite period
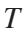
. Limit cycles can also be stable or unstable. The stability of limit cycles is assessed via **Floquet Theory**, but its development is involved, and we refrain from doing so in this work.

| Stable Fixed point | A point in a dynamical system where,  if the system is slightly perturbed in  any direction, it will return to that point. | Like a marble at the bottom of a  bowl—no matter where you nudge it,  it will roll back to the bottom. Stable  because it always returns to the same  spot. |
| --- | --- | --- |
| Unstable fixed point | An unstable fixed point is a point in  a system that repels nearby  trajectories. If the system is at that  point and is perturbed, it will not  return to that point. | Like a marble balanced on top of a  dome—any tiny push will cause it to roll  away. Unstable because even the  smallest push will move it away. |
| Saddle fixed point | A point where the system may be  attracted in some directions but  repelled in others. | Like a marble on a horse saddle—it  stays put if moved front-to-back but  rolls off side-to-side. Saddle because  stable in one direction but unstable  in another. |
| Stable limit cycle | A stable limit cycle is a repeating  pattern or cycle in a system that  attracts nearby trajectories. If the  system is slightly perturbed, it  returns to this cycle. | Like a ceiling fan spinning at steady  speed—even if slowed slightly, it  returns to original rhythm. Stable  because it settles into repeating  motion. |
| Unstable limit cycle | An unstable limit cycle is a repeating  pattern or cycle in a system that  repels nearby trajectories. | If you spin a coin perfectly, slight  wobble eventually causes it to fall.  Unstable because the spinning  motion is hard to maintain. |

### Bifurcations

In a dynamical system, **parameters** are fixed values that influence the systems behavior but do not evolve with time. For example, in the 1D equation,
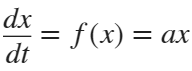
,
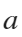
 (a real number) is the sole parameter. Clearly, choosing different parameters will produce distinct dynamics, changing the state trajectories from a given initial condition. Thus, varying parameters can shift the location of attractors in the **state space**. Furthermore, the qualitative dynamics may be altered. Loosely, a qualitative change in dynamics as parameters are varied is known as a **bifurcation** and the parameters that induced the bifurcation are known as **bifurcation parameters**. In the context of this work, bifurcations can be understood as (1) the creation/annhiliation of attractors/repellers, or (2) the change in stability of an attractor/repeller. We now give heuristic descriptions for the distinct bifurcations utilized in this work.

**Saddle-Node (SN):** We first presume that a stable fixed point and a saddle coexist a certain value of the bifurcation parameter. As the bifurcation parameter varies, these fixed point move towards one another. At a critical value, they collide and are both annhilated. Mathematically, this is realized as two distinct fixed point solutions becoming a double root (collision) and then complex conjugates. Varying the bifurcation parameter in reverse, the fixed points reappear at the point of collision and move away from one another.

**Saddle-Node on an Invariant Circle (SNIC):** We begin by assuming a stable fixed point and a saddle coexist on a closed trajectory. As we vary the bifurcation parameter, these fixed points move along the circle and gradually approach one another. At a critical value, they collide and annihilate each other, just like in a standard saddle-node bifurcation. However, this collision occurs on a periodic orbit, and their annhilation produces limit cycle. In reverse, the fixed points appear on the limit cycle, destroying the periodic solution.

**Supercritical Hopf (supH):** We start with a stable spiral that becomes increasingly oscillatory as the bifurcation parameter is varied. At the critical value, this fixed point loses stability and a stable limit cycle is born with zero amplitude — it grows continuously from the fixed point. Nearby trajectories that once spiraled into the fixed point now spiral outward and settle on the new periodic orbit. In reverse, the limit cycle shrinks and merges into the stable fixed point, restoring its stability.

**Subcritical Hopf (subH):** Here, a stable fixed point coexists with an unstable limit cycle that encircles it. As the bifurcation parameter changes, the fixed point becomes unstable, as the unstable limit cycle shrinks and merges with the fixed point, making the fixed point unstable. Reversing the bifurcation, the unstable limit cycle reappears and the fixed point becomes stable.

**Saddle Homoclinic (SH):** We consider a saddle fixed point with a trajectory that leaves and returns to the saddle along its own unstable and stable directions. As the bifurcation parameter varies, a nearby limit cycle expands and approaches this trajectory. At the bifurcation, the limit cycle collides with the saddle, causing its oscillations to become arbitrarily slow near the saddle. After the collision, the cycle disappears. In reverse, the limit cycle is born suddenly from the saddle’s orbit.

**Fold Limit Cycle (FLC):** Here, two limit cycles — one stable, one unstable — coexist. As the bifurcation parameter is varied, they approach one another in amplitude and shape. At the bifurcation point, they collide and annihilate, just like fixed points in a saddle-node bifurcation. Reversing the parameter change, the two cycles are born simultaneously.

# *Framework*

*(Much of the following is adapted or pulled directly from Saggio and Szuromi Papers.)*

The model uses fast-slow bursting to simulate seizures. A simple fast-slow burster is characterized by two rhythms: the fast rhythms of oscillations in the active or bursting state and the slow rhythm of transitions between the active and resting state.


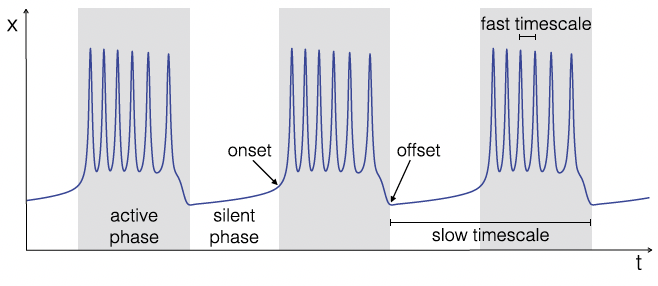


The oscillations in the bursting state are described by the time evolution of a fast subsystem (fast variables). In contrast, the transition between resting and bursting states is dictated by the time evolution of a slow subsystem (slow variables), which produces a bifurcation in the fast subsystem. Bifurcations are qualitative changes in the behavior of a dynamical system as specific parameters, known as the bifurcation parameters, are varied smoothly. For these transitions (bifurcations) to be determined by the slow subsystem, the bifurcation parameters of the fast subsystem must depend on the slow variables.

The general mathematical framework used to produce this bursting is:


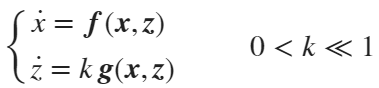


Where
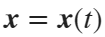
 is the 𝑛-dimensional state vector of the fast variables, and
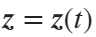
 is the 𝑚-dimensional vector of slow variables. The dots represent derivatives with respect to time. 𝒇 and 𝒈 are functions.
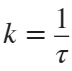
, where 𝜏 is the characteristic time constant of the separation of the fast and slow rhythms. We require 𝑘 to be significantly less than one to ensure a quasi-static variation of parameters.

Resting states are represented as stable fixed-point solutions, and oscillatory states are represented as stable limit cycles in the fast subsystem (Izhikevich, 2010). When no applied stimuli are present, to transition from a resting state to an oscillatory state, a bifurcation must occur that alters the existence or stability of the fixed point, and there must be a stable limit cycle after the bifurcation occurs. To transition back to rest from oscillations, the converse must occur.

Applied stimuli cause state switches by forcing a transition between coexisting attractors. In this case, the system must be bistable, and the forcing must move the system into the alternative attractor's basin.

# *Bursting Classes & The Unfolding*

In planar systems, there are six codim-1 (i.e. requiring the change of only one parameter value) bifurcations that can act as either the onset or offset for the bursting state. Four act as onset bifurcations: Saddle-Node (SN), Saddle-Node on an Invariant Circle (SNIC), Supercritical Hopf (SupH), and Subcritical Hopf (SubH). Four act as offset bifurcations: SNIC, SupH, Saddle-Homoclinic (SH), and Fold Limit Cycle (FLC). This yields sixteen unique pairs of planar onset and offset bifurcations, shown in Table 1, which we call the bursting class (Izhikevich 2000, Saggio et al., 2017).


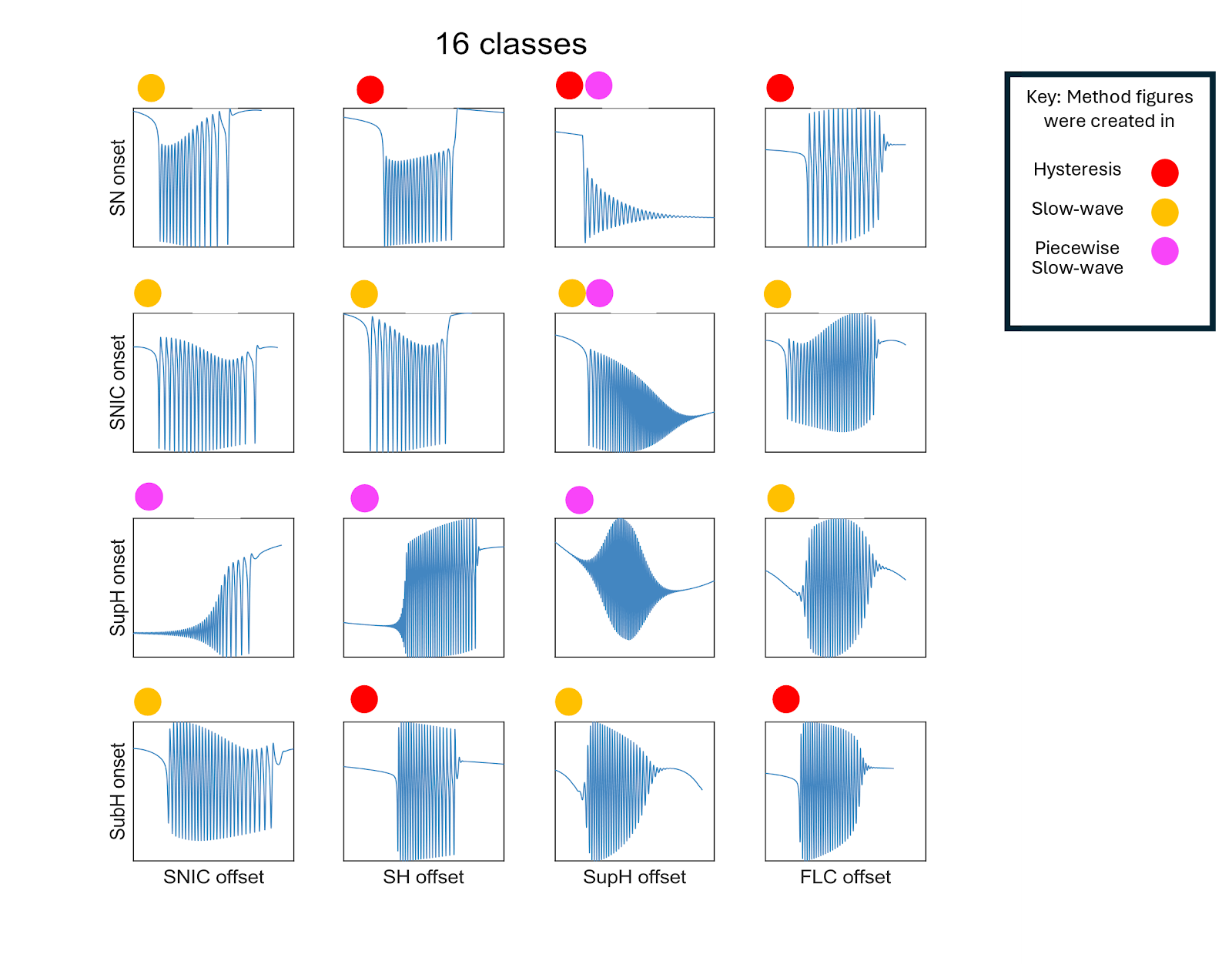


Table 1

A fast subsystem which allows for construction of all 16 bursting classes is created through "unfolding" a singularity of high codimension. The resultant equations are:


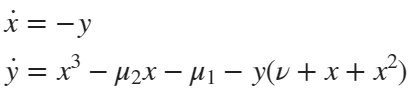


Here,
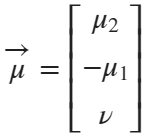
 is our parameter vector, which defines the parameter space. We often examine spherical "slices" of this parameter space, as the topological structure for large 3D regions can be inferred from the segregated regions on the sphere. Bifurcation manifolds in parameter space intersect the sphere, leaving curves which segregate the surface. In our diagrams below, we flatten this sphere for better visualization. Below is the 2d flattened diagram and the corresponding 3d projection.


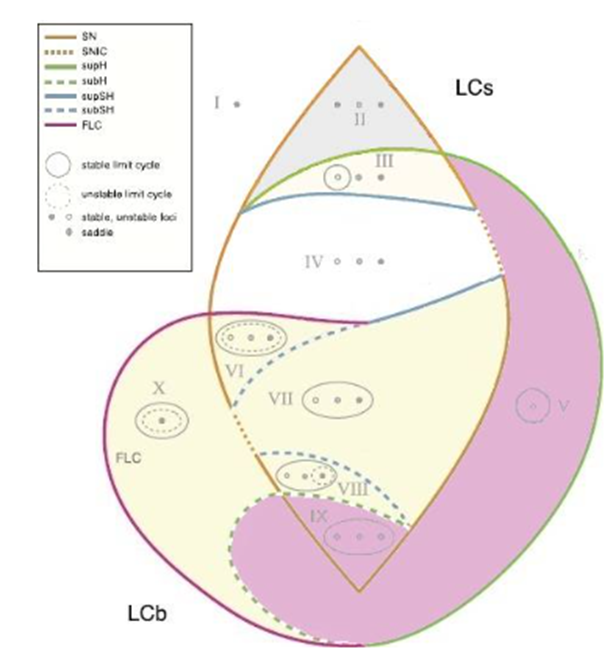


In this framework, a seizure corresponds to a stable limit cycle for sustained oscillations, while the resting state is modeled as a stable fixed point. There is also another fixed point solution, different from the resting state, which we named ‘active rest’ and consider as part of the ictal regime. Which, among these behaviors, are possible in the model depends on the values of its parameters. The input parameters m*u2, mu1,* and *nu* of the differential equations and algorithm that govern the Saggio-Jirsa fast subsystem rely on a three dimensional spherical map. At each point of the map, that is, for any precise choice of the three parameters values, the system exhibits specific behaviors. This map includes a rest and active rest region (grey), a rest/seizure or bistable region (yellow), and a seizure region (purple) that govern the behavior of the seizure. It also includes several bifurcation curves that enclose these regions and correspond to transitions among those behaviors. To manipulate the behavior of the model to create seizures, one would vary the input coordinates m*u2, mu1,* and *nu* to create a specific path within the map. Now understanding the behavior of these dynamical systems, you can begin to understand the behavior of the map in each region.

| Region Number | Topology | Description of behavior |
| --- | --- | --- |
| I | Single stable fixed point | Behavior in this region will be attraction to the stable fixed point, will see time series at a steady amplitude, |
| II | Two stable fixed points, one saddle point | Behavior in this region will be attraction to the two stable fixed points and repulsion from the saddle point. System may transition between two fixed points. Will see time series at a steady amplitude |
| III | One stable fixed point, one saddle point, one stable limit cycle with unstable point inside | Behavior in this region will be attraction to both the stable limit cycle and stable point, thus making it bistable, will see timeseries oscillating in limit cycle or at a steady amplitude at the fixed point |
| IV | One stable fixed point, one saddle point, one unstable point | Behavior in this region will be attraction to the stable point and repulsion from the saddle and unstable point, timeseries will show rest at a steady value |
| V | Stable limit cycle with unstable point inside | Behavior in this region will be attraction to the limit cycle, timeseries will show oscillations |
| VI | Stable limit cycle with unstable limit cycle inside with stable, saddle, and unstable fixed points inside | Behavior in this region will be attraction to both the stable limit cycle and stable point, thus making it bistable, will see timeseries oscillating in limit cycle or at a steady amplitude at the fixed point |
| VII | Stable limit cycle with stable, saddle, and unstable fixed points inside | Behavior in this region will be attraction to both the stable limit cycle and stable point, thus making it bistable, will see timeseries oscillating in limit cycle or at a steady amplitude at the fixed point |
| VIII | Stable limit cycle with unstable point, saddle point and unstable limit cycle with stable fixed point inside | Behavior in this region will be attraction to both the stable limit cycle and stable point, thus making it bistable, will see timeseries oscillating in limit cycle or at a steady amplitude at the fixed point |
| IX | Stable limit cycle with two unstable points and one saddle point inside | Behavior in this region will be attraction to the limit cycle, timeseries will show oscillations |
| X | Stable limit cycle with unstable limit cycle and stable point inside | Behavior in this region will be attraction to both the stable limit cycle and stable point, thus making it bistable, will see timeseries oscillating in limit cycle or at a steady amplitude at the fixed point |

These segregated regions define different dynamical regimes (shown in the next section), of which there are four predominant types: *monostable rest*, *monostable active*, and *bistable (rest/seizure) and bistable (rest/active rest)*. The bistable regions can be categorized further:

- *Limit Cycle big (LCb)* - A stable fixed point and a stable limit cycle exist. The fixed point lies on the interior of the limit cycle.
- *Limit Cycle small (LCs)* - A stable fixed point and a stable limit cycle exist. The fixed point lies on the exterior of the limit cycle.
- *Active Rest* - Two stable fixed points exist.

In the diagrams below, the topological structure of phase space (the fixed points and limit cycles) are sketched in gray in the corresponding regions of parameter space.

***Note:* when in an LCs or Active Rest bistable region, a jump between attractors results in a DC shift in the timeseries.**

figure;

get_plot()

axis off;

xlabel('\mu_2')

ylabel('-\mu_1')

zlabel('\nu')


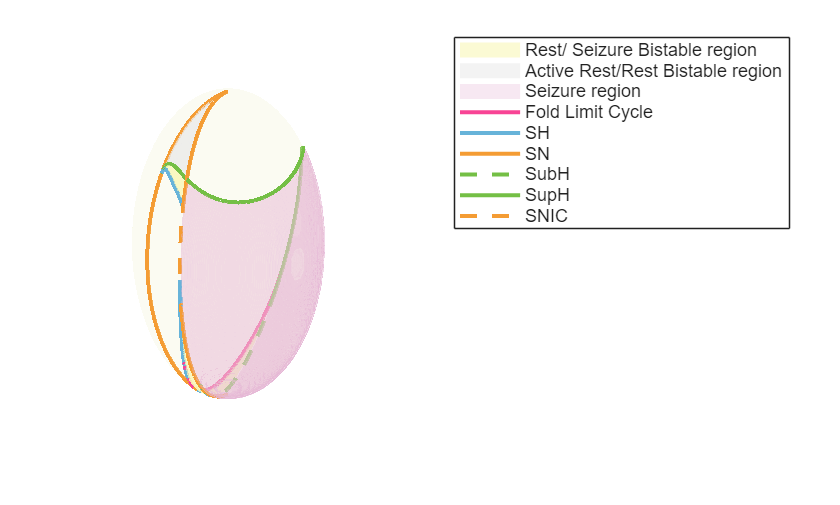


The Saggio-Jirsa model is based upon four different onset (Saddle Node (SN), Saddle Node Invariant Circle (SNIC), Supercritical Hopf (SupH), Subcritical Hopf (SubH)) and offset (Saddle Homoclinic (SH), SNIC, SupH, and Fold Limit Cycle (FLC)) bifurcations.

Each of these has distinct, well described dynamical properties.


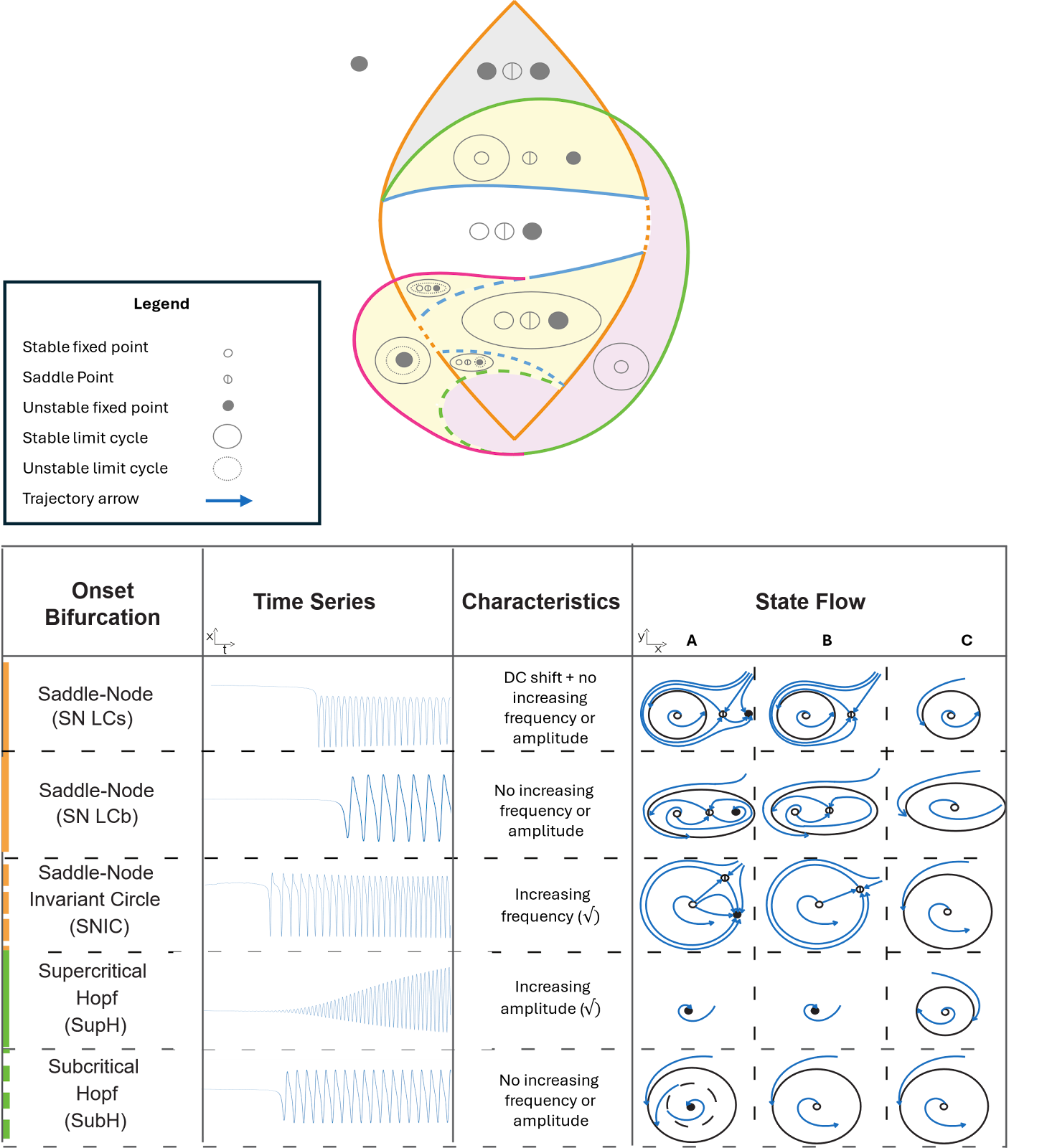


The top portion of this figure shows a bifurcation diagram of the Saggio-Jirsa model. The lower portion shows visualization of onset dynamics in the Saggio-Jirsa bursting model. Saddle Node onsets and Saddle Homoclinic offsets can both arise with or without direct current (DC) shifts, which were distinguished by the presence of big (LCb) or small (LCs) limit cycles in state space (Saggio et al., 2017). Bifurcations scale from zero based on the either square root or logarithmic scaling laws as indicated. The lower right portion of the figure illustrates the state flow diagrams for key bifurcations, showing how system trajectories evolve through state space during seizure onset.

Below, we detail the flow behavior and corresponding signal effect for each bifurcation.

1. Saddle-Node (SN) → DC Shift.

Flow Behavior: Pre-bifurcation: The system resides at a stable equilibrium point (node). A nearby unstable equilibrium (saddle point) exists but has no effect on the system's current state. At bifurcation: As a control parameter crosses a critical threshold, the stable node and saddle point collide and annihilate. Post-bifurcation: With no remaining equilibria, trajectories are forced to escape to the limit cycle.

Signal Effect: A sudden DC shift (voltage jump) occurs as the system transitions from rest to limit cycle.

2. Saddle-Node (SN) → no DC Shift.

Flow behavior: Pre-bifurcation: The system rests at a stable equilibrium (node), with a nearby unstable equilibrium (saddle). At bifurcation: The stable node and saddle collide and annihilate (fold bifurcation), but unlike the standard SN case, the points are enclosed in the limit cycle the system jumps to. Post-bifurcation: The system escapes to the limit cycle.

Signal Effect: No abrupt voltage shift (unlike standard SN) because system jumps to nearby limit cycle. Immediate onset of oscillations (frequency may depend on system parameters).

3. Saddle-Node on Invariant Cycle (SNIC) → Increasing Frequency.

Flow Behavior: Pre-bifurcation: Two equilibria (a stable node and a saddle) exist on a closed orbit (invariant cycle). The system can rest at the stable node. At bifurcation: The equilibria collide and vanish, leaving the invariant cycle intact but now devoid of fixed points. Post-bifurcation: Trajectories must flow continuously around the cycle, forming a limit cycle. Near the bifurcation, the period of oscillation is infinite. As the parameter moves further, the period decreases.

Signal Effect: Oscillations emerge with initially low frequency (long intervals between spikes). Frequency ramps up as the limit cycle tightens (period shortens).

4. Supercritical Hopf (SupH) → Increasing Amplitude.

Flow Behavior: Pre-bifurcation: The system is at a stable equilibrium (spiral sink). Small perturbations decay back to rest. At bifurcation: The equilibrium loses stability. A stable limit cycle emerges smoothly from the fixed point (radius grows from zero). Post-bifurcation: The limit cycle’s amplitude grows as the parameter moves away from the bifurcation point.

Signal Effect: Smooth onset of small oscillations that grow in amplitude.

5. Subcritical Hopf (SubH) → Arbitrary Jumps.

Flow Behavior: Pre-bifurcation: The equilibrium is stable, but an unstable limit cycle surrounds it. At bifurcation: The unstable cycle shrinks and merges with the equilibrium, destabilizing it. Trajectories now escape to a large-amplitude limit cycle. Post-bifurcation: The system exhibits large, sudden oscillations with hysteresis.

Signal Effect: Abrupt onset of high-amplitude oscillations (no smooth transition).


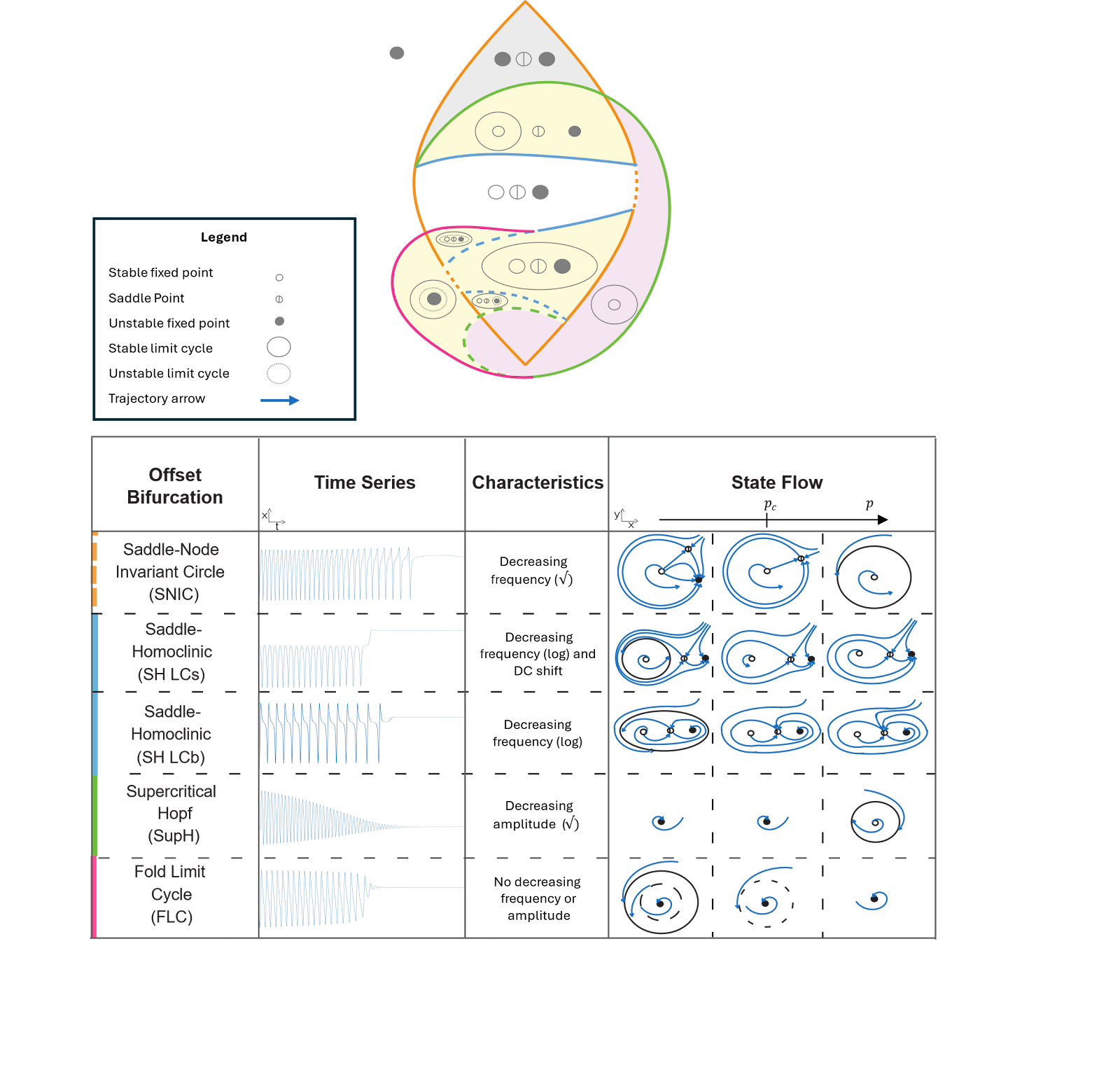


The top portion of this figure shows a bifurcation diagram of the Saggio-Jirsa model. The lower portion shows visualization of onset dynamics in the Saggio-Jirsa bursting model. Saddle Node onsets and Saddle Homoclinic offsets can both arise with or without direct current (DC) shifts, which were distinguished by the presence of big (LCb) or small (LCs) limit cycles in state space (Saggio et al., 2017). Bifurcations scale to zero based on the either square root or logarithmic scaling laws as indicated. The lower right portion of the figure illustrates the state flow diagrams for key bifurcations, showing how system trajectories evolve through state space during seizure offset. Each diagram highlights the pre- and post-bifurcation behavior, including fixed points, limit cycles, and trajectory directionality.

Below, we detail the flow behavior and corresponding signal effect for each bifurcation:

1.Saddle-Node on Invariant Circle (SNIC) → Decreasing Frequency.

Flow Behavior: Pre-termination: Oscillations follow a limit cycle with a "ghost" of the vanished saddle-node pair, causing trajectories to slow near the collision point. At bifurcation: Infinite-period orbit (trajectories take infinitely long to complete a cycle). Post-termination: System collapses to fixed point.

Signal Effect: Exponentially increasing inter-spike intervals (frequency ↘) as trajectories slow near the ghost saddle-node, then abrupt silence when the limit cycle collides.

2. Saddle-Homoclinic (SH) → Decreasing Frequency and DC shift.

Flow Behavior: Pre-termination: Limit cycle approaches a homoclinic orbit, causing trajectories to spend more time near the saddle. At bifurcation: Infinite-period loop forms, then breaks. Post-termination: Oscillations vanish; system returns to equilibrium.

Signal Effect: Spike-wave bursts with logarithmically slowing frequency as phase flow stretches near the saddle’s unstable manifold, then voltage snaps to baseline when the homoclinic loop breaks.

3. Saddle-Homoclinic (SH LCb) → Decreasing Frequency.

Flow Behavior: Pre-termination: Limit cycle approaches a homoclinic orbit, causing trajectories to spend more time near the saddle. At bifurcation: Infinite-period loop forms, then breaks. Post-termination: Oscillations vanish; system returns to equilibrium.

Signal Effect: Decreasing frequency (logarithmic slowing) and No DC shift (returns to original baseline).

4. Supercritical Hopf (SupH) → Decreasing Amplitude.

Flow Behavior: Pre-termination: Stable limit cycle shrinks smoothly toward the now-stable equilibrium. At bifurcation: Cycle radius reaches zero; equilibrium regains stability. Post-termination: System rests at the fixed point.

Signal Effect: Smooth amplitude decay as the limit cycle shrinks radially to the equilibrium, no frequency change, then silence at bifurcation.

5. Fold Limit Cycle (FLC) → Sudden shift to zero.

Flow Behavior: Pre-termination: Stable and unstable limit cycles coexist (stable cycle is observable). At bifurcation: Cycles collide and annihilate. Post-termination: Oscillations abruptly cease; trajectories fall to equilibrium.

Signal Effect: Oscillations vanish mid-cycle (no slowing) when stable/unstable cycles collide, causing instant voltage return to baseline.

# *Bursting Paths & Burster Types*

To create a burster, we must identify a path through parameter space that “connects” onset and offset bifurcation curves. We call this path the bursting path; it is the set of parameter values along which the system varies in order to exhibit the proper sequence of bifurcations that yield a burster. To create a burster of a particular class, we must find a path that appropriately connects the correct onset and offset bifurcations. Movement of the system along this path is accomplished by parameterizing the bursting path in terms of the slow variables,
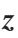
.


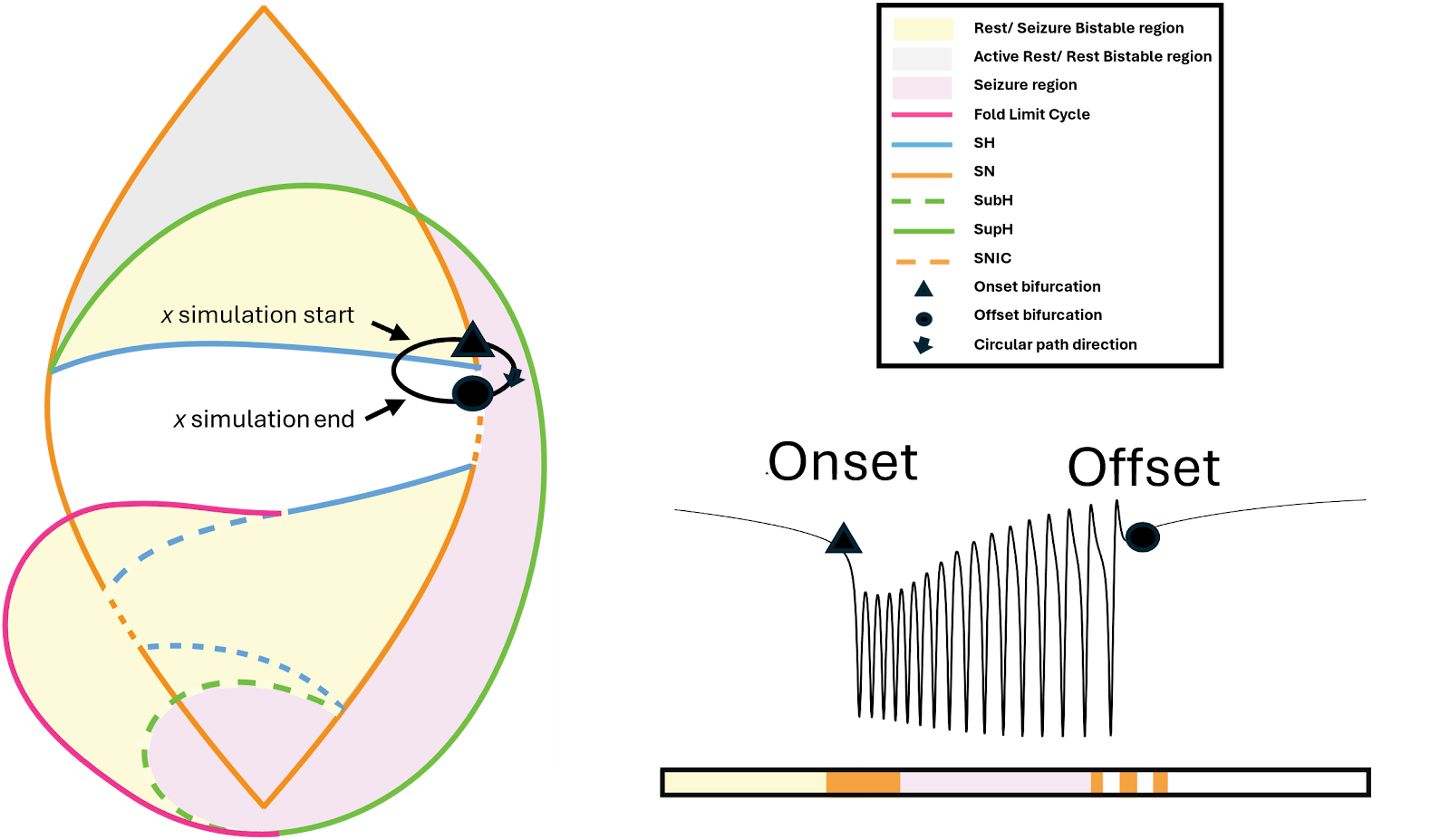


The traversal of this path is driven by the slow oscillation of the system. The slow oscillation can occur through two mechanisms:

- *Slow-wave burster -* The slow subsystem is a self-sustained oscillator, thus feedback from the fast to the slow subsystem is not required. In this case, the slow subsystem must be at least two-dimensional,
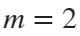
.
- *Hysteresis-loop burster -* The slow subsystem oscillates due to feedback from the fast subsystem. This can occur if the fast subsystem shows hysteresis between the silent and active states, which can be used to inform the slow subsystem about the state of the fast subsystem (e.g., by baseline). In this case, one slow variable is enough,
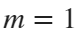
.

# Adding noise into the equation of the simulation

Seizures generated using Saggio et al.’s dynamical model of fast-slow bursting are mostly realistic, but they lack certain features of human sEEG recordings, such as noise. We added dynamical pink noise to our simulated seizures. Pink noise was chosen because it closely resembles noise in the brain (i.e. 1/f noise).

Dynamical noise, or parametric noise, was added to the fast variable (*x*) of the model equations (see Saggio 2017). This represents noise in the brain (i.e. random voltage fluctuations) that creates small perturbations, some of which may push the system into or out of the seizure state (da Silva 2003, Maturana 2020). In the hysteresis model, additional noise stops the hysteresis effect, and the system undergoes noise induced transitions as opposed to bifurcation induced transitions.

In the slow wave model, individual bifurcations responded differently to additional dynamical noise. The SubH and SupH bifurcations produce seizures that were largely unaffected by noise because most of their path was in the obligatory seizure region. Note that the SubH theoretically might be more susceptible to noise when it is in the the fixed point/limit cycle bistability region, which we did not test. The SN and SNIC bifurcations do not clearly start the seizure or have the visual characteristics we typically associate with the bifurcation at high levels of noise. Note this may be a byproduct of the proximity to the bistability region. This may also explain why SNIC bifurcations are so hard to spot in human data and account for 3% of all human data seizures.

# *Hysteresis-Loop Bursters*

## Slow Dynamics

For hysteresis-loop bursters, the simplest construction of a path is an arc (a segment of a great circle) on the sphere. This arc is drawn from a point on the offset curve through a bistable region to a point on the onset curve. We design the slow dynamics so that the traversal of this path works as follows. When the system is at rest, the parameters should change so that the system moves towards the onset bifurcation. When the system is in an active state, the system should move towards offset. Using a one dimensional slow-subsystem, this means we want
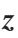
 to increase if the system is at rest, and decrease if the system is seizing. The following formulation accomplishes this:


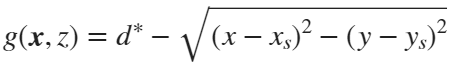


Here,
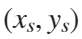
 are the coordinates of the resting state. We note that
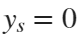
 always. We also note that
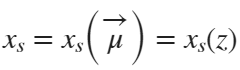
 since
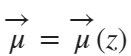
.

This formulation essentially measures the distance between the current location of the system and the resting state. If this distance is below a threshold,
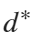
,
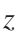
 increases, and the parameters approach critical onset values. When above the threshold,
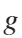
 is negative, and we approach offset.

## Parametrization

For the slow dynamics to generate a hysteresis-loop burster, we need an appropriate parametrization of
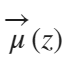
 (bursting path). As we said, the simplest way is to make an arc connecting points on onset and offset curves using a segment of a great circle.

First, we choose points in parameter space,
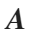
 and
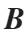
. Where
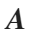
 lies on the offset curve, and
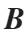
 lies on the onset. Given these choices the parametric equation is then


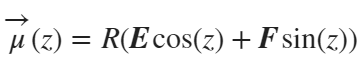


where


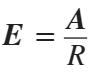
 and
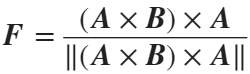


Given that the system begins at rest with
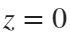
, and that the arc path connecting
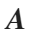
 to
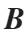
 solely passes through bistable regions, this parametrization (coupled with the slow dynamics above) will produce a hysteresis-loop burster.

## Paths

5 of the 16 burster types can be created using hysteresis-loop bursters


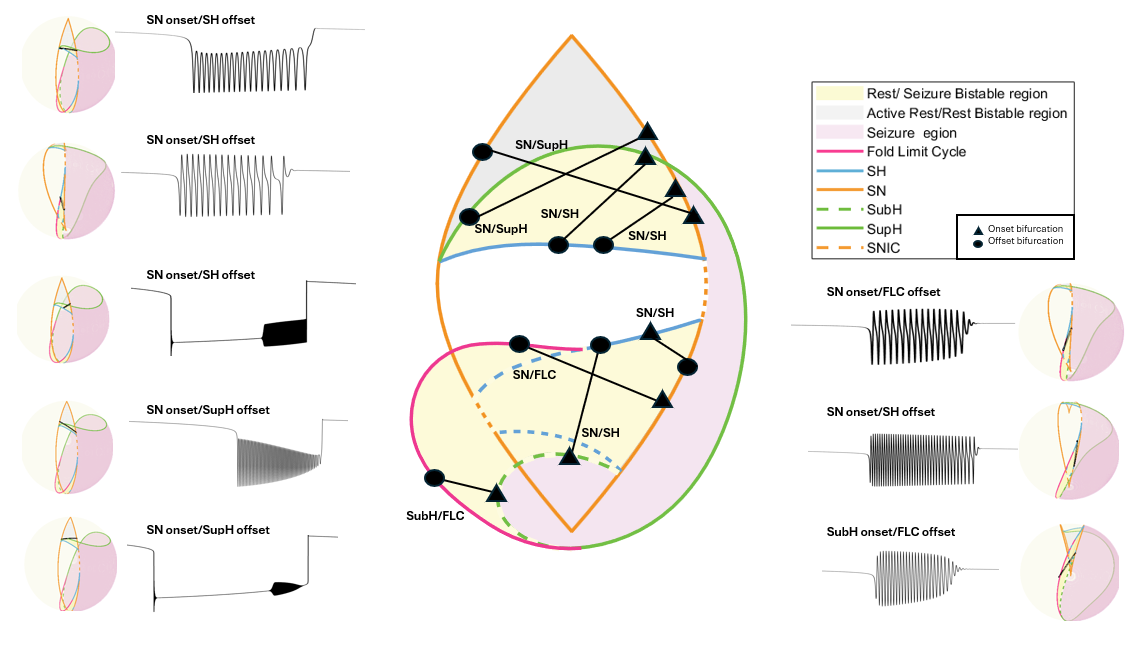


## CODE

### Settings

clear all

% Settings - Integration

x0=[0;0;0]; % initial conditions (must be a column)

% Settings - Model

% focus

b = 1.0;

% radius of the sphere, do not change

R = 0.4;

% The dstar parameter is an excitability parameter that controls the ratio between duration of seizure and duration of rest. When dstar is smaller or equal to zero, no seizure activity is possible, and the system will always stay at rest. For small';

% positive values of dstar, seizure and rest occur. For sufficiently big values of dstar, only seizure activity is possible. '

dstar = 0.3;

%%'The parameter k determines how fast the slow variable traverses through

%%parameter space. A smaller k yields a slower traversal of k.

k=0.00015;

%the parameter k_fast determines how many oscillations are in the burst. A

%higher k is yields more bursts, a lower k yields less oscillations

k_fast = 1;

%the parameter alpha controls the amplitude of the burst. a lower alpha

%yields a smaller amplitude, a higher alpha yields a higher amplitude

alpha = 0.2;

% The N parameter controls solution of resting state. Upper Branch (Case 1): Smoother transitions, reduced hysteresis. Lower Branches (Cases 2 and 3): Potential for hysteresis, with complex, path-dependent responses and multiple equilibria. The systems

% state may not revert immediately when external conditions are reversed, creating the characteristic hysteresis loop.

N = 1;

%length of time the simulation will run for

tmax = 15000;

%Integration step/Sampling rate of the simulation, assume it is represented in miliseconds

tstep = 0.01;

%%class wanted to run, input '2s', '2b', '3s','4b','10', '11' '14', '16'

%this shows what labels correspond to what class : '2s - SN/SH', '2b-SN/SH', '3s- SN/SUP','4b - SN/FLC ','10-SN/SH', '11-SN/Sup' '14-SN/Sub', '16-SN/Sup'

class = '2s';

%% function takes in class, and gets a randomized point on each

%% bifurcation curve

[onset_curve,offset_curve]=hysteresis_random_path(class);

onset_curve_length=length(onset_curve);

offset_curve_length=length(offset_curve);

%%choose specific points. These points will be the index of the total curve

%%length found in the previous two lines. The number chosen must be less

%%than the total length of the particular curve

onset_index = 11; %start at first point on the onset curve

offset_index = 3; %end at 50th point on the offset curve

A = offset_curve(:,offset_index);

B = onset_curve(:,onset_index);

% uncomment this code to do random path

% % One random path - select random point on onset curve and offset curve

% random_onset_index=randsample(onset_curve_length,1);

% random_offset_index=randsample(offset_curve_length,1);

% A = offset_curve(:,random_offset_index);

% B = onset_curve(:,random_onset_index);

tspan = 0:tstep:tmax;

% Create arc path

[E, F] = Parametrization_2PointsArc(A,B,R);

N_t = length(tspan);

X = zeros(3,N_t);

xx = x0;

%Dynamical pink noise, or parametric noise, is added to the fast variable (x) of'the governing model equations. This represents noise in the brain

%i.e. random voltage fluctuations) that creates small perturbations, some of which'may push the system into or out of the seizure state.

sigma = 00;

Rn = [pinknoise([1,N_t],-1, sigma);pinknoise([1,N_t],-1, 00);pinknoise([1,N_t],-1, 00)];

%integration to generate signal

for n = 1:N_t

% Euler-Meruyama method

Fxx = HysteresisLoop_Model(tspan(n),xx,b,k,k_fast, alpha, R,dstar,E,F,N);

xx = xx + tstep*Fxx + sqrt(tstep)*Rn(:,n);

X(:,n) = xx;

end

x = X';

t = tspan;

% Onset happens when you leave the resting state. In the absence of noise you can use peaks in the z variable.

% With noise, you need to explicitly track the distance between the resting state and the current state of the system.

% When it exceeds dstar, that should be good. Vice versa for offset. In the model function you will see another function

% used called resting state. This function calculates the x coordinate for the fixed points, x_rs (y coordinate is always 0).

% Then just take the distance between (x_rs, 0) and (x, y), the current position of the system.

% If you look at the equations for zdot you will see this is how z “knows how to turn around

z = x(:,3);

% Calculate Bursting Path

mu2 = R.*(E(1).*cos(z)+F(1).*sin(z));

mu1 = -R.*(E(2).*cos(z)+F(2).*sin(z));

nu = R.*(E(3).*cos(z)+F(3).*sin(z));

figure;

grid off;

set(gca, 'XColor', 'none');

plot(t,x(:,1),'LineWidth',1,'DisplayName','x', 'Color','k');

%xticks([])

hold on

plot(t,x(:,3),'r','LineWidth',2,'DisplayName','z')

hold off

%xlabel('t')

title('Timeseries')

legend('Location','northwest','FontSize',18)


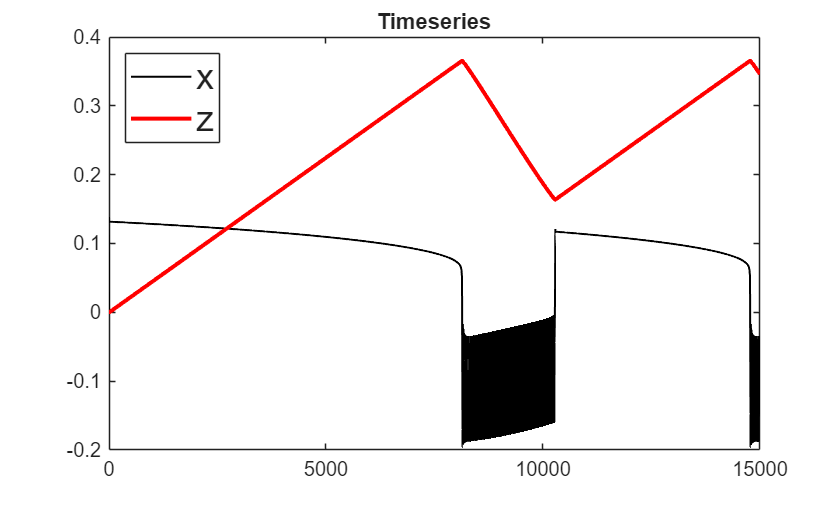


figure;

get_plot();

plot3(mu2,-mu1,nu,'k', 'DisplayName', 'Bursting Path', 'LineWidth',3);

xlabel('\mu_2')

ylabel('-\mu_1')

zlabel('\nu')


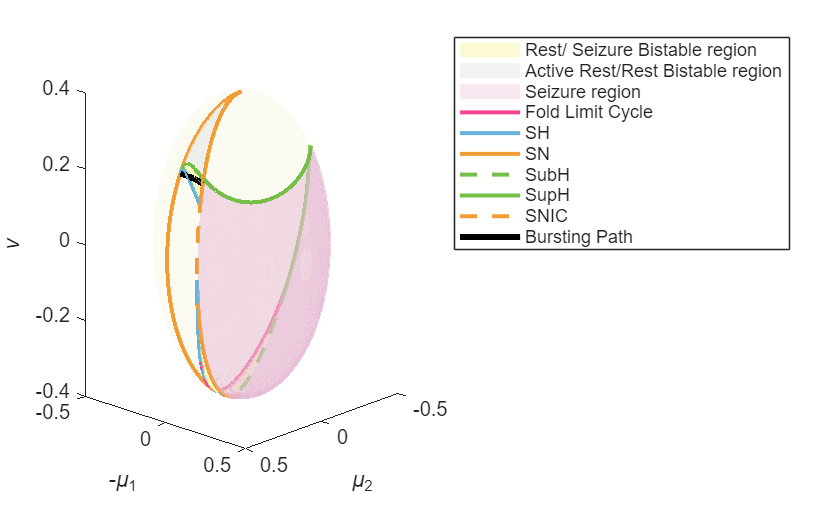


# SLOW-WAVE BURSTERS - CIRCULAR

## Parametrization

Slow wave bursters must be self-sustaining oscillations. In other words, the direction in which the bursting path is traversed will not change due to feedback from the fast subsystem (i.e. whether the system is active or resting). Thus, the simplest way to construct a slow wave path is to draw a closed loop (circle) which is traversed in a single direction, driven by a dummy variable at constant velocity:


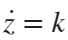


We need 3 initial points in parameter space to define the circle on the sphere. Call them
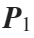
,
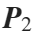
,
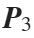
. Here, the traversal of the path is intended to pass through these points in sequential order. The unique circle which passes through these points can be described as the intersection of the sphere and the plane which passes through these 3 points. First, we define the plane. We start with the normal vector:


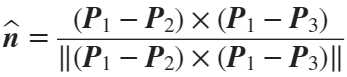


Letting
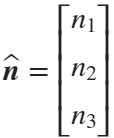
, the equation of the plane is the standard formulation:


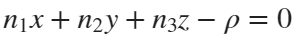


where
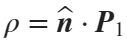
is the signed distance from the origin to the plane. Note
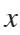
,
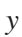
,
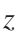
 refer to coordinates in parameter space here.

Using the pythagorean identity, we find that the radius of the smaller circle is
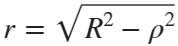
.


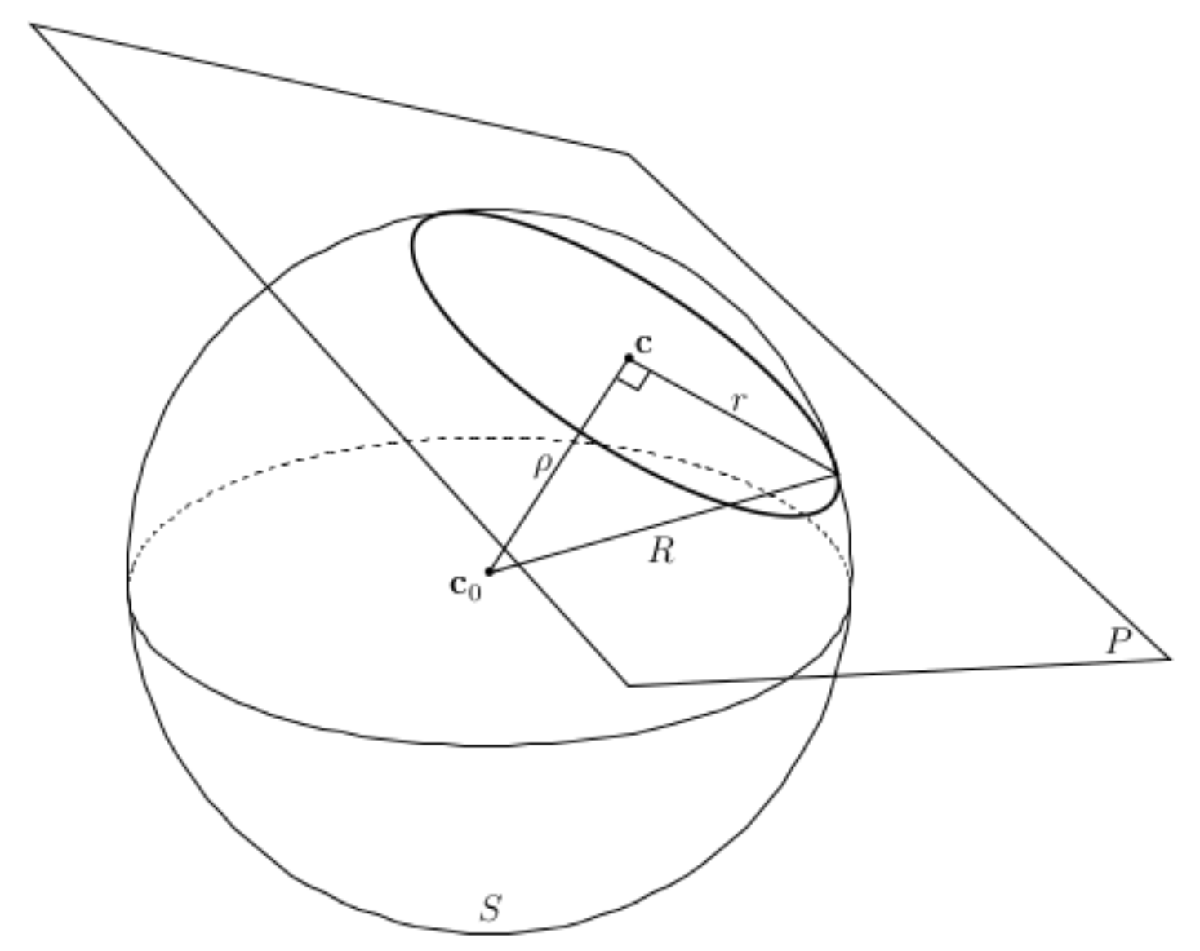


The center of the smaller circle is
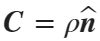
. This allows us to construct the following parametrization:


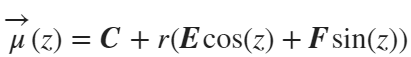


where


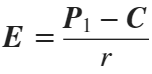
 and
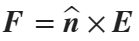
.

This step is similar to the hysteresis-loop path because it is constructed by making a great circle on the smaller sphere centered at
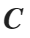
 with radius
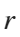
.

## Paths

8 of the 16 burster types are created using slow-wave bursters


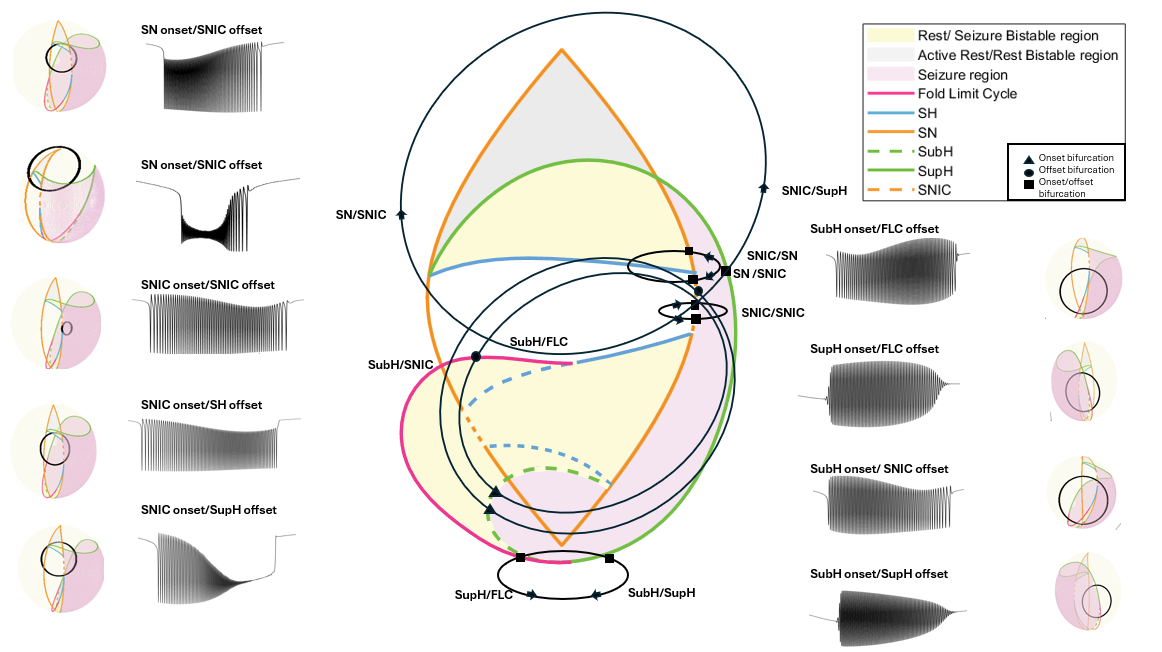


## CODE

### Settings

clear all

% SETTINGS - INTEGRATION

x0=[0;0;0]; % initial conditions (must be a column)

% Settings - Model

% focus

b = 1.0;

% radius of the sphere, do not change

R = 0.4;

%%'The parameter k determines how fast the slow variable traverses through

%%parameter space. A smaller k yields a slower traversal of k.

k=0.00035;

%the parameter k_fast determines how many oscillations are in the burst. A

%higher k is yields more bursts, a lower k yields less oscillations

k_fast = 1;

%the parameter alpha controls the amplitude of the burst. a lower alpha

%yields a smaller amplitude, a higher alpha yields a higher amplitude

alpha = 1;

%length of time the seizure will run for

tmax = 24000;

%Integration step/Sampling rate of the simulation

tstep = 0.01;

%%class wanted to run, input 1,5, 6,7,8,9,12,13,15

%this shows what labels correspond to what class : '1 - SN/SNIC', '5

%-SNIC/SNIC', '6 - SNIC/SH','7 - SNIC/SUP','8-Sub/FLC', '9-Sup/SNIC'

%'12-Sup/FLC', '13-Sub/SNIC', '15-Sub/Sup',

class = 13;

%% function takes in class 2,4,14,16, and gets a randomized point on each bifurcation curve

[onset_curve,offset_curve,offset_curve2, flag] = slow_wave_circular_random_path(class);

onset_curve_length=length(onset_curve);

offset_curve_length=length(offset_curve);

%%choose specific points

onset_index = 1;

offset_index = 1;

if flag == 2 || flag == 3

p1 = onset_curve(:,onset_index);

p2 = offset_curve(:,offset_index);

p3 = offset_curve2;

else

p1 = onset_curve(:,onset_index);

p2 = offset_curve2(:,offset_index);

p3 = offset_curve;

end

% uncomment this code to do random path

% % One random path - select random point on onset curve and offset curve

% onset_curve_length=length(onset_curve);

% offset_curve_length=length(offset_curve);

% random_onset_index=randsample(onset_curve_length,1);

% random_offset_index=randsample(offset_curve_length,1);

% if flag == 2 || flag == 3

%

% p1 = onset_curve(:,random_onset_index);

% p2 = offset_curve(:,random_offset_index);

% p3 = offset_curve2;

% else

% p1 = onset_curve(:,random_onset_index);

% p2 = offset_curve2(:,random_offset_index);

% p3 = offset_curve;

% end

tspan = 0:tstep:tmax;

% Create circular path based 3 defining points

[E, F, C, r] = Parametrization_3PointsCircle(p1',p2',p3');

N_t = length(tspan);

X = zeros(3,N_t);

xx = x0;

sigma = 40;

Rn = [pinknoise([1,N_t],-1, sigma);pinknoise([1,N_t],-1, 00);pinknoise([1,N_t],-1, 00)];

mu2_big = zeros(1, length(N_t));

mu1_big = zeros(1, length(N_t));

nu_big = zeros(1, length(N_t));

%integration to generate signal

for n = 1:N_t

%Euler-Meruyama method

[Fxx, mu2, mu1,nu] = SlowWave_Model(tspan(n),xx,b,k,k_fast, alpha, E,F,C,r);

xx = xx + tstep*Fxx + sqrt(tstep)*Rn(:,n);

X(:,n) = xx;

mu2_big(n) = mu2;

mu1_big(n) = mu1;

nu_big(n) = nu;

end

% Ensure p1, p2, p3 are column vectors (if not already)

p1 = p1(:);

p2 = p2(:);

p3 = p3(:);

mu1_big = -mu1_big;

tol = 1e-6;

if class == 5 || class == 1 || class == 6 || class == 8 || class == 13

% Find onset_idx (match p1 in mu2_big, mu1_big, nu_big)

onset_idx = find( ...

abs(mu2_big(:) - p2(1)) < tol & ...

abs(mu1_big(:) - p2(2)) < tol & ...

abs(nu_big(:) - p2(3)) < tol ...

);

% Find offset_idx (match p2 in mu2_big, mu1_big, nu_big)

offset_idx = find( ...

abs(mu2_big(:) - p1(1)) < tol & ...

abs(mu1_big(:) - p1(2)) < tol & ...

abs(nu_big(:) - p1(3)) < tol ...

);

else

% Find onset_idx (match p1 in mu2_big, mu1_big, nu_big)

onset_idx = find( ...

abs(mu2_big(:) - p1(1)) < tol & ...

abs(mu1_big(:) - p1(2)) < tol & ...

abs(nu_big(:) - p1(3)) < tol ...

);

% Find offset_idx (match p3 in mu2_big, mu1_big, nu_big)

offset_idx = find( ...

abs(mu2_big(:) - p2(1)) < tol & ...

abs(mu1_big(:) - p2(2)) < tol & ...

abs(nu_big(:) - p2(3)) < tol ...

);

end

onset_idx = unique(onset_idx);

offset_idx = unique(offset_idx);

onset_idx_ = onset_idx(1);

offset_idx_ = offset_idx(2);

x = X';

t = tspan;

% Calculate Bursting Path

z=0:0.01:2*pi;

mu2=C(1)+r*(E(1)*cos(z)+F(1)*sin(z));

mu1=-(C(2)+r*(E(2)*cos(z)+F(2)*sin(z)));

nu=C(3)+r*(E(3)*cos(z)+F(3)*sin(z));

figure;

plot(t,x(:,1),'LineWidth',1, 'Color','k')

hold on

if(length(x) >= offset_idx)

scatter(t(onset_idx), 1, 'g');

scatter(t(offset_idx),1, 'r')

end

xlabel('t')

ylabel('x')

title('Timeseries')


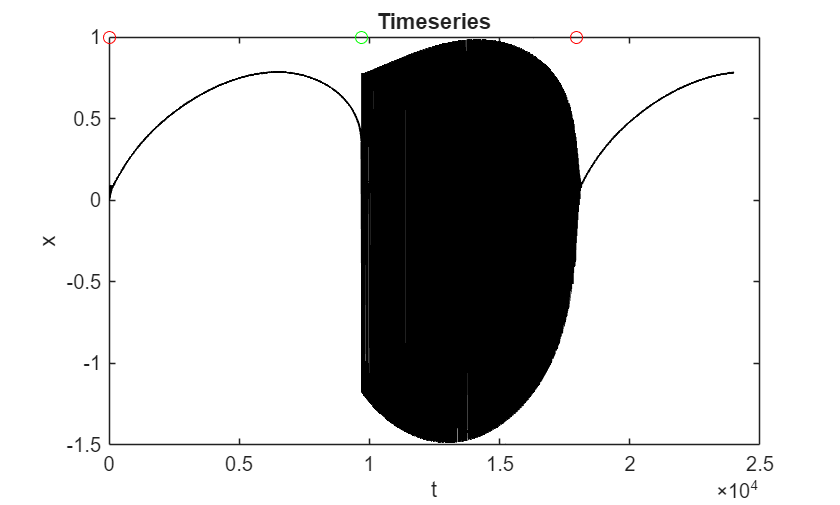


figure;

get_plot();

plot3(mu2, -mu1, nu, 'k', 'DisplayName', 'Bursting Path', 'LineWidth',3);

xlabel('\mu_2')

ylabel('-\mu_1')

zlabel('\nu')


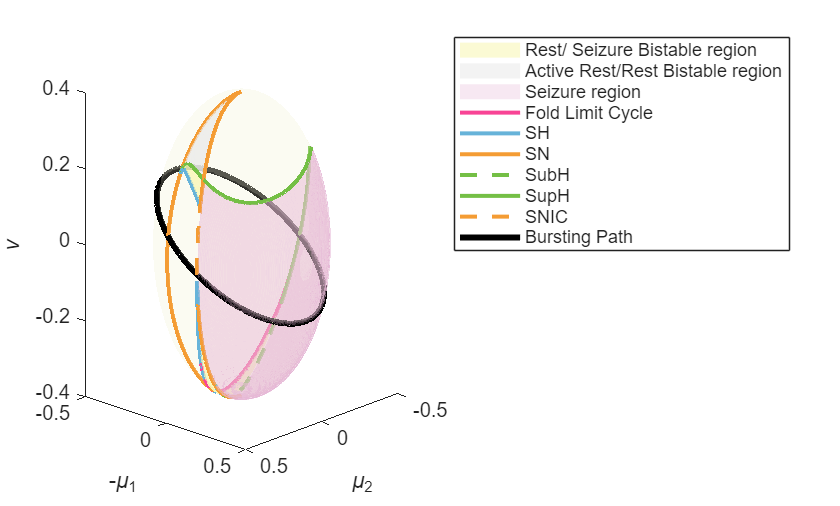


# SLOW-WAVE BURSTERS - PIECEWISE

## Parametrization

Slow wave bursters must be self-sustaining oscillations. In other words, the direction in which the bursting path is traversed will not change due to feedback from the fast subsystem (i.e., whether the system is active or resting. In certain classes, paths cross the saddle node bifurcation and exhibit dc shifts. If these secondary dynamics are undesirable, one can manipulate the path to not cross the saddle-node bifurcation. While slow wave circles are theoretically plausible to achieve this goal, they are practically difficult because some onset-offset pairs are challenging to connect with continuous pathways without crossing other bifurcations. To address this, we can construct piecewise paths using the slow wave method. In addition, this approach allows a bursting pathway to begin and end in different locations in the state space, rather than returning to the original starting point.

To do this, direct pathways between defined points are used to move through specific locations. Four arcs are created on the surface of the sphere to make a piecewise arc path using 4 points. The first point is a fixed point in the rest region. The second point is a point on the onset bifurcation curve. The third point is a randomized point in the limit cycle region. The fourth point is a point in the offset bifurcation curve. The arc paths are created from the rest point to first onset bifurcation point, first bifurcation point to limit cycle point, limit cycle point to second bifurcation point, and offset bifurcation point to the rest point, to create a continuous path. Next, to calculate the total time the path traversed, the path was scaled by the k variable and t step variable. Note that unlike the previous two methods, this method only traverses the path one time during the simulation. We produced 5 classes with this method

## Paths

5 of the 16 burster types are created using slow-wave piecewise bursters


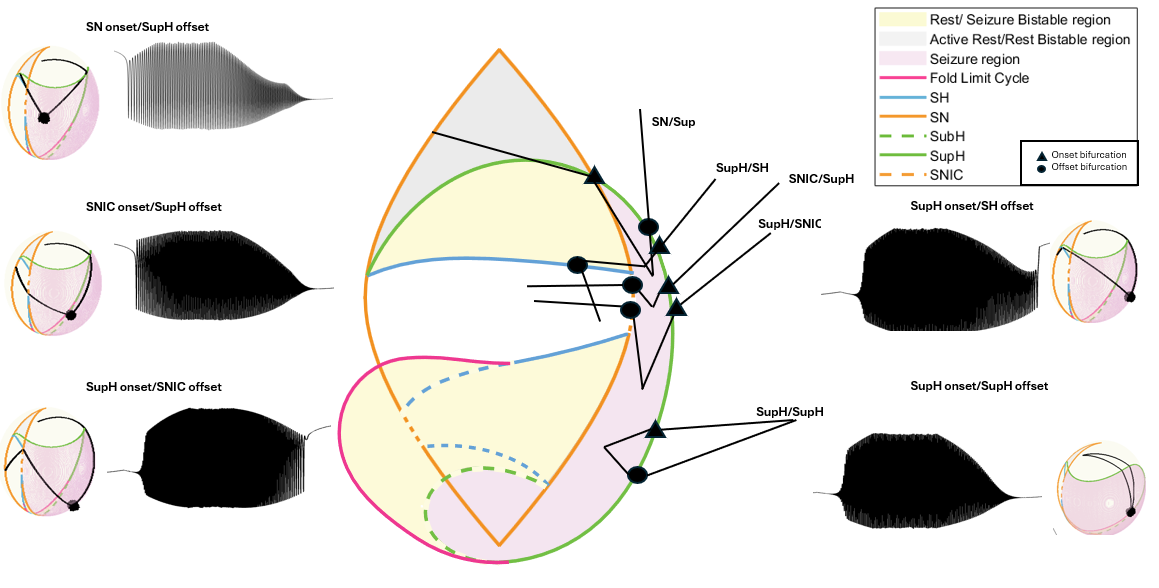


# CODE

### Settings

clear all

% SETTINGS - INTEGRATION

x0=[0;0;0]; % initial conditions (must be a column)

% Settings - Model

% focus

b = 1.0;

% radius of the sphere, do not change

R = 0.4;

%%'The parameter k determines how fast the slow variable traverses through

%%parameter space. A smaller k yields a slower traversal of k.

k=0.00015;

%the parameter k_fast determines how many oscillations are in the burst. A

%higher k is yields more bursts, a lower k yields less oscillations

k_fast = 0.05;

%the parameter alpha controls the amplitude of the burst. a lower alpha

%yields a smaller amplitude, a higher alpha yields a higher amplitude

alpha = 0.2;

%Integration step/Sampling rate of the simulation

tstep = 0.01;

%%class wanted to run, input 3,7,9,10,11

%this corresponds to '3 - SN/Sup', '7 - SNIC/Sup', '9- Sup/SNIC', '10 -Sup/SH',

% '11 - Sup/Sup'

class = 11;

[p0,onset_curve,p1_5,offset_curve,p3]=piecewise_random_path(class);

onset_curve_length=length(onset_curve);

offset_curve_length=length(offset_curve);

%%choose specific points

onset_index = 1;

offset_index = 44;

p1 = onset_curve(onset_index,:);

p2 = offset_curve(offset_index,:);

% uncomment this code to do random path

% % One random path - select random point on onset curve and offset curve

% random_onset_index=randsample(onset_curve_length,1);

% random_offset_index=randsample(offset_curve_length,1);

% p1 = onset_curve(:,random_onset_index);

% p2 = offset_curve(:,random_offset_index);

stall_val = 30000;

[mu2_straight_path0,mu1_straight_path0,nu_straight_path0,rad1] = sphereArcPath(k,tstep,p0,p1);

[mu2_straight_path0_5,mu1_straight_path0_5,nu_straight_path0_5,rad2] = sphereArcPath(k,tstep,p1,p1_5);

points = repmat(p1_5, stall_val, 1)';

%path noise sigma

sigma = 100;

Rn = [pinknoise([1,length(points)],-1, sigma);pinknoise([1,length(points)],-1, sigma);pinknoise([1,length(points)],-1, sigma)];

points = points + Rn;

[mu2_straight_path,mu1_straight_path,nu_straight_path,rad3] = sphereArcPath(k,tstep,p1_5,p2);

[mu2_straight_path1,mu1_straight_path1,nu_straight_path1,rad4] = sphereArcPath(k,tstep,p2,p3);

mu2_all = [mu2_straight_path0, mu2_straight_path0_5, points(1, :), mu2_straight_path, mu2_straight_path1];

mu1_all = [mu1_straight_path0, mu1_straight_path0_5, points(2, :), mu1_straight_path, mu1_straight_path1];

mu1_all = -mu1_all;

nu_all = [nu_straight_path0, nu_straight_path0_5, points(3,:), nu_straight_path, nu_straight_path1];

N_t = length(mu2_all);

X = zeros(3,N_t);

xx = x0;

%signal pink noise sigma

sigma = 100;

Rn = [pinknoise([1,N_t],-1, sigma);pinknoise([1,N_t],-1, 00);pinknoise([1,N_t],-1, 00)];

mu2_big = zeros(1, length(N_t));

mu1_big = zeros(1, length(N_t));

nu_big = zeros(1, length(N_t));

%%get onset index by finding Radians to bifurcation, and getting index

%%through k and tstep parameters

onset_index = floor((rad1/k)/tstep);

offset_index = floor(((rad1+rad2+rad3)/k)/tstep) + stall_val;

%integration to generate signal

for n = 1:N_t

%%Euler-Meruyama method

[Fxx,mu2,mu1,nu] = SlowWave_Model_piecewise(0,xx,b,k,k_fast, alpha, mu2_all(n), mu1_all(n),nu_all(n));

xx = xx + tstep*Fxx + sqrt(tstep)*Rn(:,n);

X(:,n) = xx;

mu2_big(n) = mu2;

mu1_big(n) = mu1;

nu_big(n) = nu;

end

x = X';

seizure = x(:,1);

figure;

plot(seizure,'k');

hold on;

scatter(onset_index,1, 'g');

scatter(offset_index,1, 'r');


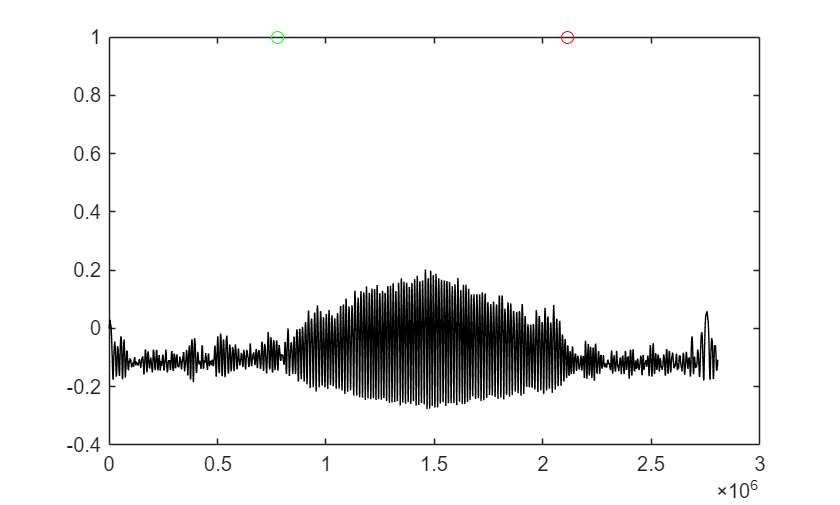


figure;

hold on;

get_plot();

hold on;

plot3(mu2_all, -mu1_all, nu_all, 'k', 'LineWidth',2, 'DisplayName', 'Bursting Path');


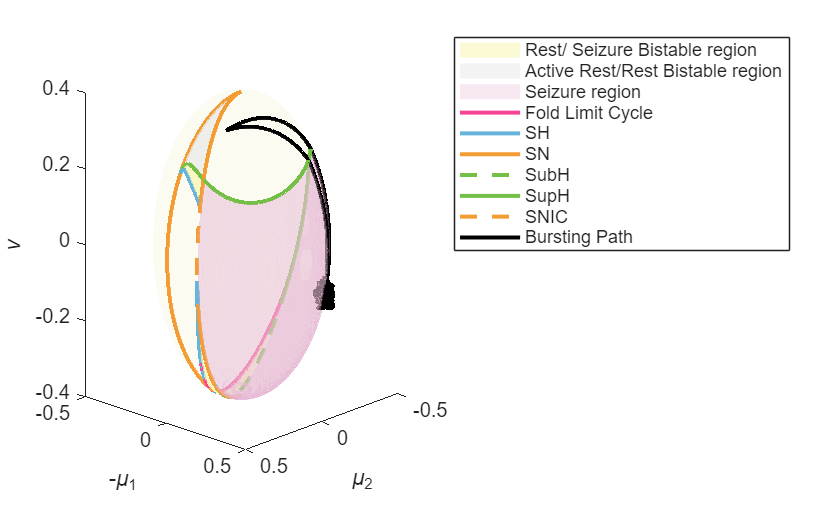


# Postprocessing

Seizures generated using Saggio et al.’s dynamical model of fast-slow bursting have similar bursting patterns as human seizures, but they lack certain features of human sEEG recordings, such as noise. We added dynamical pink noise to our simulated seizures. Pink noise was chosen because it closely resembles noise in the brain. Dynamical noise, or parametric noise, was added to the fast variable (*x*) of the model equations (see Saggio 2017) to represent noise in the brain (i.e. random voltage fluctuations) that creates small perturbations, some of which may push the system into or out of the seizure state (da Silva 2003, Maturana 2020). In the hysteresis model, additional noise **stops** the hysteresis loop bursting, and the system undergoes noise induced transitions as opposed to bifurcation induced transitions.


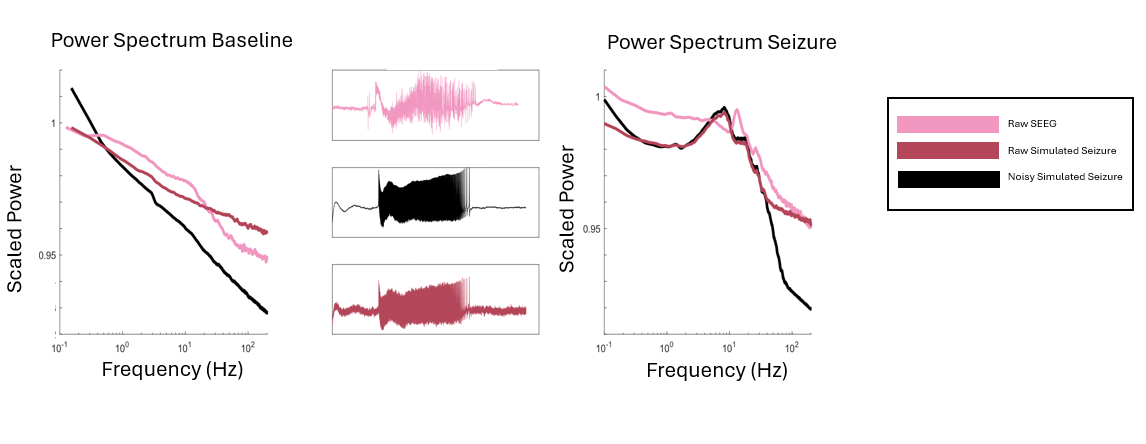


%%using previous signal

data = x(:,1);

[pks,locs] = findpeaks(data, 'MinPeakProminence', 0.10);

fs = 1/(0.01*tstep);

t = (0:length(data)-1) / fs;

figure;

plot(data, 'Color',[180/255, 70/255, 90/255]);

hold on;

plot(locs, pks, 'ro'); % Plot peaks with red circles

title(['Number of Peaks: ', num2str(numel(locs))]);

hold off;


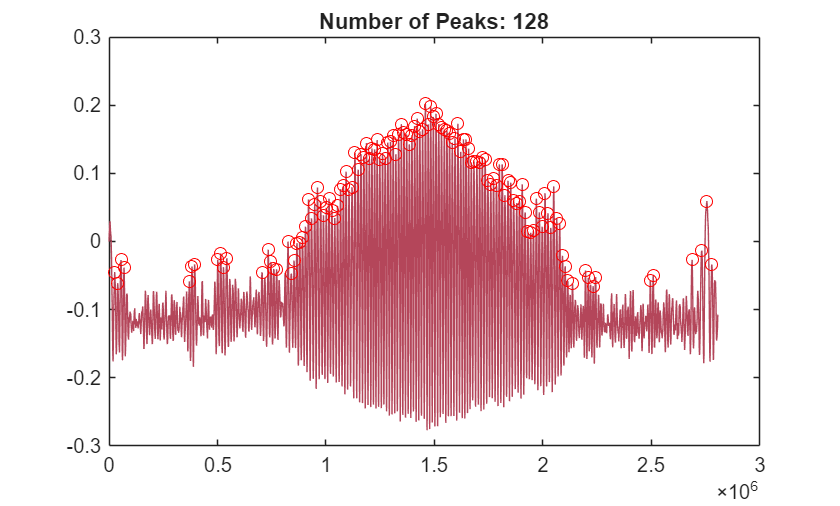


%%Getting average spike rate

time_in_seconds = locs / fs; % Convert peak indices to seconds

spike_rates = diff(time_in_seconds);

average_frequency = mean(spike_rates);

fprintf('The average frequency of the signal is %.2f Hz\n', average_frequency);

The average frequency of the signal is 2.17 Hz

We adjust the sampling rate of simulated seizures such that spike frequency was consistent with clinical guidelines. According to AES, a seizure consists of rhythmic bursting activity between 1-30 Hz

if average_frequency < 1 || average_frequency > 30

% Calculate spike rates

spike_rates = diff(time_in_seconds);

% Adjust spike rates to achieve a mean average spiking rate of 5 Hz

target_avg_spike_rate = (1/10); % Hz

% Calculate the current average spiking rate

current_avg_spike_rate = mean(spike_rates);

% Calculate the adjustment factor

adjustment_factor = target_avg_spike_rate / current_avg_spike_rate;

% Adjust spike rates

adjusted_spike_rates = spike_rates * adjustment_factor;

% Calculate the mean average spiking rate after adjustment

mean_avg_spike_rate = mean(adjusted_spike_rates);

% Calculate the new sampling frequency

new_sampling_frequency = fs / adjustment_factor;

disp('New sampling frequency (Hz):');

disp(new_sampling_frequency);

disp('New average frequency of the signal (Hz):');

disp(1/target_avg_spike_rate)

end

%add pink noise over

min_val = min(highpass(data(:), 5, fs));

max_val = max(highpass(data(:), 5, fs));

Spontaneous DC shifts reflect alterations in the excitation level of neuronal membrane potentials. The rapid charge accumulation at the electrode-electrolyte interface causes electrode polarization which induces a baseline drift that is distinguished by an electrode’s material properties. Modern EEG recording equipment uses alternating current (AC) amplifiers with low-frequency high-pass filters to perform baseline correction once amplifier saturation occurs. To emulate the residual baseline drift observed from platinum-iridium electrodes in human EEG, we applied 6th order digital high-pass filters with cutoff frequencies between 0.1 and 1 Hz


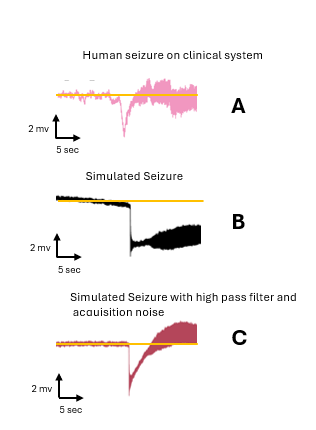


HPF = designfilt('highpassiir', ... % Response type

'FilterOrder',1, ... % Filter Order Specification

'HalfPowerFrequency',0.1, ...

'DesignMethod','butter', ... % Design method

'SampleRate',fs); % Sample rate

data = filter(HPF, data);

figure;

plot(t,data, 'Color',[180/255, 70/255, 90/255]);

title('Electrode drift')


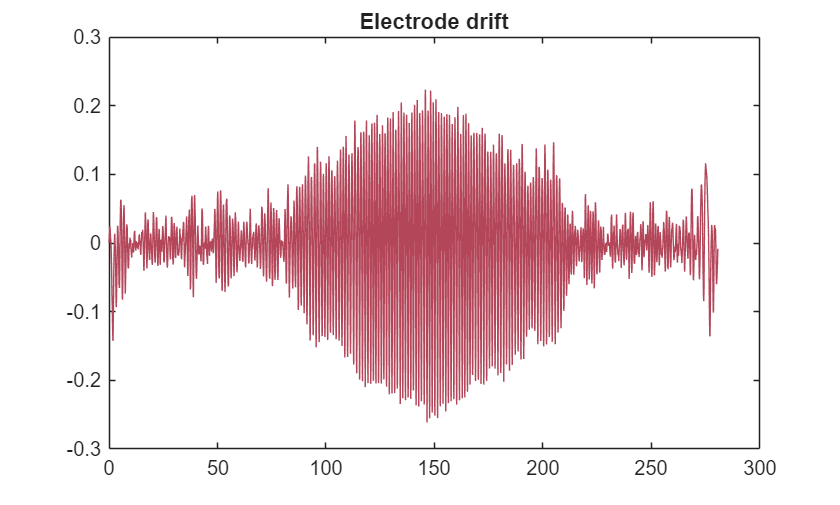


We added 0%, 20%, and 40% signal acquisition pink noise. In the first, we did not add any signal acquisition noise. In the second, we added pink noise that was 20% of the signal amplitude, to represent mediocre recording conditions. In the third, we added pink noise that was 40% of the signal amplitude, to represent poor recording conditions. Signal acquisition noise is picked up by the electrodes used to record brain activity. This neural activity of surrounding brain tissue can be modeled as “pink noise”, or 1/f spatial noise with a normal error distribution (Lennon 2000). The amplitude of signal acquisition noise depends on the signal-to-noise ratio. We generated several datasets to represent variability in clinical recording conditions. We found adding pink noise caused the power spectrums to match.


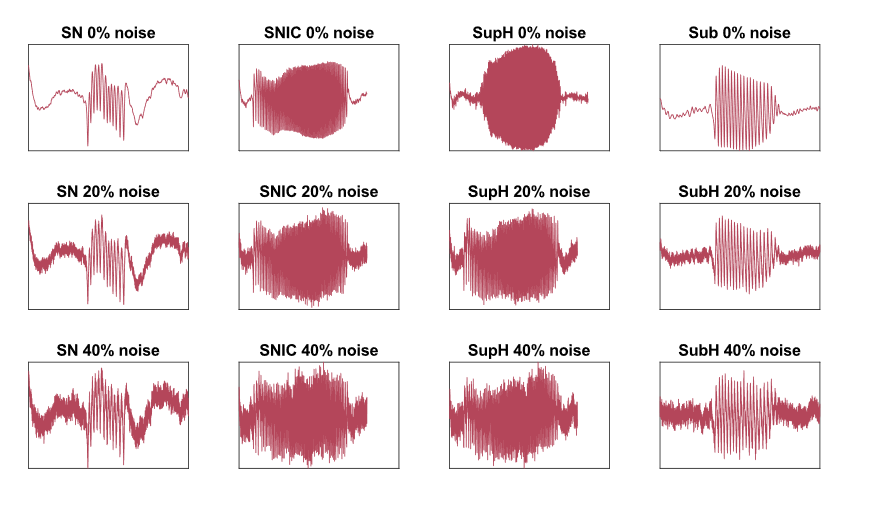


data = (data - min_val) / (max_val - min_val);

rms_signal = get_amp(data, fs);

normalized_data = data;

noisy_data_20 = add_pink_noise(normalized_data, rms_signal, 0.2, fs);

noisy_data_40 = add_pink_noise(normalized_data, rms_signal,0.4, fs);

%%Flipping the data to double the dataset

doubled_data = normalized_data;

flipped_data = (doubled_data*-1)+1;

%%Flipping the data to double the dataset

normalized_data_1 = noisy_data_20;

doubled_data = normalized_data_1;

flipped_data_1 = (doubled_data*-1)+1;

%%Flipping the data to double the dataset

normalized_data_40 = noisy_data_40;

doubled_data_40 = normalized_data_40;

flipped_data_40 = (doubled_data_40*-1)+1;

%Normalizing the data between 0 and 1 and plotting

figure

subplot(2, 3, 1);

data = normalized_data;

min_val = min(data(:));

max_val = max(data(:));

data = (data - min_val) / (max_val - min_val);

normalized_data = data;

plot(t,normalized_data, 'Color',[180/255, 70/255, 90/255]); % Interpolated data

xlabel('Time');

ylabel('Data, 0 percent noise');

subplot(2, 3, 2);

data = flipped_data;

min_val = min(data(:));

max_val = max(data(:));

data = (data - min_val) / (max_val - min_val);

flipped_data = data;

plot(t,flipped_data, 'Color',[180/255, 70/255, 90/255]); % Interpolated data

xlabel('Time');

ylabel('Data, 0 percent noise');

subplot(2, 3, 3);

data = normalized_data_1;

min_val = min(data(:));

max_val = max(data(:));

data = (data - min_val) / (max_val - min_val);

normalized_data_1 = data;

plot(t,normalized_data_1, 'Color',[180/255, 70/255, 90/255]);

xlabel('Time');

ylabel('Data, 20 percent noise');

subplot(2, 3, 4);

data = flipped_data_1;

min_val = min(data(:));

max_val = max(data(:));

data = (data - min_val) / (max_val - min_val);

flipped_data_1 = data;

plot(t,flipped_data_1, 'Color',[180/255, 70/255, 90/255]);

xlabel('Time');

ylabel('Data, 20 percent noise');

subplot(2, 3, 5);

data = normalized_data_40;

min_val = min(data(:));

max_val = max(data(:));

data = (data - min_val) / (max_val - min_val);

normalized_data_40 = data;

plot(t,normalized_data_40, 'Color',[180/255, 70/255, 90/255]);

xlabel('Time');

ylabel('Data, 40 percent noise');

subplot(2, 3, 6);

data = flipped_data_40;

min_val = min(data(:));

max_val = max(data(:));

data = (data - min_val) / (max_val - min_val);

flipped_data_40 = data;

plot(t,flipped_data_40, 'Color',[180/255, 70/255, 90/255]);

xlabel('Time');

ylabel('Data, 40 percent noise');


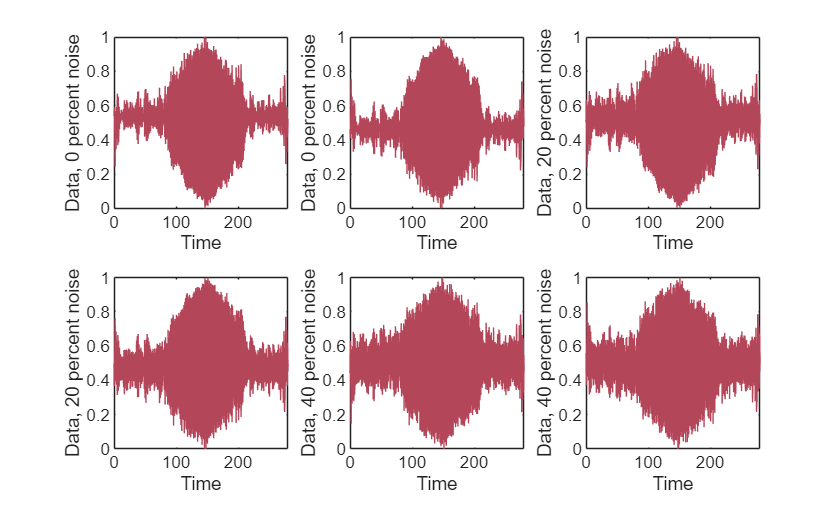


This plot shows the final post-processed data, once it has been electrode-drift corrected, flipped to double the dataset, and approximately 20% and 40%% signal acquisition noise has been added overtop.

# *Functions*

function x_rs = Resting_State(mu2,mu1,nu,N)

switch N

case 1 % resting state on upper branch

x_rs=mu2/(3*(mu1/2 + (mu1^2/4 - mu2^3/27)^(1/2))^(1/3)) + (mu1/2 + (mu1^2/4 - mu2^3/27)^(1/2))^(1/3);

case 2 % resting state on lower branch

x_rs=- mu2/(6*(mu1/2 + (mu1^2/4 - mu2^3/27)^(1/2))^(1/3)) - (mu1/2 + (mu1^2/4 - mu2^3/27)^(1/2))^(1/3)/2 - (3^(1/2)*i*(mu2/(3*(mu1/2 + (mu1^2/4 - mu2^3/27)^(1/2))^(1/3)) - (mu1/2 + (mu1^2/4 - mu2^3/27)^(1/2))^(1/3)))/2;

case 3

x_rs= (3^(1/2)*i*(mu2/(3*(mu1/2 + (mu1^2/4 - mu2^3/27)^(1/2))^(1/3)) - (mu1/2 + (mu1^2/4 - mu2^3/27)^(1/2))^(1/3)))/2 - (mu1/2 + (mu1^2/4 - mu2^3/27)^(1/2))^(1/3)/2 - mu2/(6*(mu1/2 + (mu1^2/4 - mu2^3/27)^(1/2))^(1/3));

end

end

function [E,F] = Parametrization_2PointsArc(A,B,R)

E = A./R;

F=cross(cross(A,B),A);

F=F./norm(F);

end

function x = pinknoise(DIM,BETA, MAG)

%% the function pinknoise(dimension,beta,amplitude) can be used to modify noise

% parameters. The beta parameter is the slope of 1/f^beta. Beta = 0 is white

%noise, beta = -1 is pink, and -2 is brownian. This parameter can be changed to fit the slope of

%desired noise (typically between -0.5 and 1.5), as demonstrated in Suppl Fig. 7 in Jirsa et al. Brain 2014.

%Magnitude can be modified to fit the desired noisiness of the data

% This function generates 1/f^b spatial noise, with a normal error

% distribution

%

% DIM is a two component vector that sets the size of the spatial pattern

% (DIM=[10,5] is a 10x5 spatial grid)

%

% BETA defines the spectral distribution.

% Spectral density S(f) = N f^BETA

% (f is the frequency, N is normalisation coeff).

% BETA = 0 is random white noise.

% BETA -1 is pink noise

% BETA = -2 is Brownian noise

% The fractal dimension is related to BETA by, D = (6+BETA)/2

%

% MAG is the scaling variable for the noise amplitude

%

% The method is briefly descirbed in Lennon, J.L. "Red-shifts and red

% herrings in geographical ecology", Ecography, Vol. 23, p101-113 (2000)

u = [(0:floor(DIM(1)/2)) -(ceil(DIM(1)/2)-1:-1:1)]'/DIM(1);

u = repmat(u,1,DIM(2));

v = [(0:floor(DIM(2)/2)) -(ceil(DIM(2)/2)-1:-1:1)]/DIM(2);

v = repmat(v,DIM(1),1);

S_f = (u.^2 + v.^2).^(BETA/2);

S_f(S_f==inf) = 0;

phi = rand(DIM);

y= S_f.^0.5 .* (cos(2*pi*phi)+i*sin(2*pi*phi));

y=y.*MAG/max(abs(y)); %set the mag to the level you want

x= ifft2(y);

x = real(x);

end

function Xdot = HysteresisLoop_Model(~,x,~,k,k_fast, alpha, R,dstar,E,F,N)

% Parametrization of the path in the spherical parameter space in terms of great

% circles

mu2=R*(E(1)*cos(x(3))+F(1)*sin(x(3)));

mu1=-R*(E(2)*cos(x(3))+F(2)*sin(x(3)));

nu=R*(E(3)*cos(x(3))+F(3)*sin(x(3)));

% x coordinate of resting state (i.e. upper branch of eq)

x_rs=real(Resting_State(mu2,mu1,nu, N));

%use this to integrate changes in alpha with the resting state

%x_rs=real(eval_resting_state_cartesian(alpha,mu2,mu1,N));

% equations

xdot = -k_fast* alpha*x(2);

ydot = -k_fast*(-(x(1)/alpha)^3 +mu2*(x(1)/alpha) +mu1 + x(2)*( nu + (x(1)/alpha) + (x(1)/alpha)^2));

zdot = -k*(sqrt(((x(1)/alpha)-x_rs)^2+x(2)^2)-dstar);

Xdot = [xdot;ydot;zdot];

end

function [E, F, C, r] = Parametrization_3PointsCircle(p1, p2, p3)

% Calculate unit direction vectors

p1 = p1';

p2 = p2';

p3 = p3';

V12 = (p1 - p2) / norm(p1 - p2);

V13 = (p1 - p3) / norm(p1 - p3);

% Compute the normal vector to the plane defined by the points

n = cross(V12, V13);

n = n / norm(n); % Normalize the normal vector

% Calculate the coefficients for the plane equations

dalpha = dot(p1, n);

dbeta = dot(V12, p1 + (p2 - p1) / 2);

dgamma = dot(V13, p1 + (p3 - p1) / 2);

% Set up the linear equations to find the center C

A = [n(1), n(2), n(3);

V12(1), V12(2), V12(3);

V13(1), V13(2), V13(3)];

b = [dalpha; dbeta; dgamma];

% Solve for C using least squares

C = A\b;

% Calculate E (unit vector from C to p1)

E = (p1 - C) / norm(p1 - C);

% Calculate F (perpendicular vector)

F = -cross(E, n);

% Calculate the radius r

r = norm(p1 - C);

end

function [Xdot, mu2, mu1,nu] = SlowWave_Model(~,x,~,k,k_fast,alpha, E,F,C,r)

% Parametrization of the path in the spherical parameter space in terms

% of a circle defined by 3 points

mu2=C(1)+r*(E(1)*cos(x(3))+F(1)*sin(x(3)));

mu1=-(C(2)+r*(E(2)*cos(x(3))+F(2)*sin(x(3))));

nu=C(3)+r*(E(3)*cos(x(3))+F(3)*sin(x(3)));

% System

xdot = -k_fast* alpha*x(2);

ydot = -k_fast*(-(x(1)/alpha)^3 +mu2*(x(1)/alpha) +mu1 + x(2)*( nu + (x(1)/alpha) + (x(1)/alpha)^2));

zdot = k;

Xdot = [xdot;ydot;zdot];

end

function get_plot()

marker_size = 10;

load('curves.mat')

load('curves2.mat')

load('bifurcation_crossing.mat')

load('sphere_mesh.mat')

load('testmesh.mat');

hold on;

linewidth = 2;

% Plot different meshes with assigned DisplayName for the legend

vertices = BCSmesh.vertices;

faces = BCSmesh.faces;

h1 = patch('Vertices', vertices, 'Faces', faces, ...

'FaceColor', [0.973, 0.965, 0.722], 'EdgeColor', 'none', 'FaceAlpha', 0.6, 'DisplayName', 'BCS Mesh');

vertices = Active_restmesh.vertices;

faces = Active_restmesh.faces;

h2 = patch('Vertices', vertices, 'Faces', faces, ...

'FaceColor', [0.9216, 0.9216, 0.9216], 'EdgeColor', 'none', 'FaceAlpha', 0.6, 'DisplayName', 'Active Rest Mesh');

vertices = Seizure_mesh.vertices;

faces = Seizure_mesh.faces;

h3 = trisurf(faces, vertices(:,1), vertices(:,2), vertices(:,3), ...

'FaceColor', [0.894, 0.706, 0.831], 'EdgeColor', 'none', 'FaceAlpha', 0.3, 'DisplayName', 'Seizure Mesh');

vertices = Bistable_Lcb_mesh.vertices;

faces = Bistable_Lcb_mesh.faces;

h4 = patch('Vertices', vertices, 'Faces', faces, ...

'FaceColor', [0.973, 0.965, 0.722], 'EdgeColor', 'none', 'FaceAlpha', 0.6, 'DisplayName', 'Bistable Lcb Mesh');

scale_array = [0.4];

scale_array = scale_array / 0.4;

% Arrays to store plot handles for the legend

legend_handles = [h1, h2, h3];

legend_names = {'Rest/ Seizure Bistable region', 'Active Rest/Rest Bistable region', 'Seizure region'};

% Scale factors to adjust radius from 0.4

for i = 1:length(scale_array)

% Scale the coordinates of the points for radius 0.39

Fold_of_cycles_scaled = scale_array(i) * Fold_of_cycles;

Homoclinic_to_saddle3_scaled = scale_array(i) * Homoclinic_to_saddle3;

Homoclinic_to_saddle2_scaled = scale_array(i) * Homoclinic_to_saddle2;

Homoclinic_to_saddle1_scaled = scale_array(i) * Homoclinic_to_saddle1;

Homoclinic_to_saddle_scaled = scale_array(i) * Homoclinic_to_saddle;

Fold_scaled = scale_array(i) * Fold;

Hopf_scaled = scale_array(i) * Hopf;

SNIC_scaled = scale_array(i) * SNIC;

% Plot all scaled lines with DisplayName for the legend

h5 = plot3(Fold_of_cycles_scaled(1, :), Fold_of_cycles_scaled(2, :), Fold_of_cycles_scaled(3, :), 'Color', [0.9725,0.2667,0.5843], 'LineWidth', linewidth, 'DisplayName', 'Fold of Cycles');

h6 = plot3(Homoclinic_to_saddle3_scaled(1, :), Homoclinic_to_saddle3_scaled(2, :), Homoclinic_to_saddle3_scaled(3, :), 'Color', [0.404, 0.702, 0.851], 'LineWidth', linewidth, 'LineStyle', '--', 'DisplayName', 'Homoclinic to Saddle 3');

h7 = plot3(Homoclinic_to_saddle2_scaled(1, :), Homoclinic_to_saddle2_scaled(2, :), Homoclinic_to_saddle2_scaled(3, :), 'Color', [0.404, 0.702, 0.851], 'LineWidth', linewidth, 'DisplayName', 'Homoclinic to Saddle 2');

h8 = plot3(Homoclinic_to_saddle1_scaled(1, :), Homoclinic_to_saddle1_scaled(2, :), Homoclinic_to_saddle1_scaled(3, :), 'Color', [0.404, 0.702, 0.851], 'LineWidth', linewidth, 'LineStyle', '--', 'DisplayName', 'Homoclinic to Saddle 1');

h9 = plot3(Homoclinic_to_saddle_scaled(1, :), Homoclinic_to_saddle_scaled(2, :), Homoclinic_to_saddle_scaled(3, :), 'Color', [0.404, 0.702, 0.851], 'LineWidth', linewidth, 'DisplayName', 'Homoclinic to Saddle');

h10 = plot3(Fold_scaled(1, 140:564), Fold_scaled(2, 140:564), Fold_scaled(3, 140:564), 'Color', [0.957, 0.612, 0.204], 'LineWidth', linewidth, 'DisplayName', 'Fold Part 1');

h11 = plot3(Fold_scaled(1, 575:end), Fold_scaled(2, 575:end), Fold_scaled(3, 575:end), 'Color', [0.957, 0.612, 0.204], 'LineWidth', linewidth, 'DisplayName', 'Fold Part 2');

h12 = plot3(Fold_scaled(1, 1:80), Fold_scaled(2, 1:80), Fold_scaled(3, 1:80), 'Color', [0.957, 0.612, 0.204], 'LineWidth', linewidth, 'DisplayName', 'Fold Part 3');

h13 = plot3(Hopf_scaled(1, 1:400), Hopf_scaled(2, 1:400), Hopf_scaled(3, 1:400), 'Color',[0.4549 ,0.7490 ,0.2706], 'LineWidth', linewidth, 'LineStyle', '--', 'DisplayName', 'Hopf Part 1');

h14 = plot3(Hopf_scaled(1, 400:973), Hopf_scaled(2, 400:973), Hopf_scaled(3, 400:973), 'Color',[0.4549 ,0.7490 ,0.2706], 'LineWidth', linewidth, 'DisplayName', 'Hopf Part 2');

h15 = plot3(SNIC_scaled(1, :), SNIC_scaled(2, :), SNIC_scaled(3, :), 'Color',[0.957, 0.612, 0.204], 'LineWidth', linewidth, 'LineStyle', '--', 'DisplayName', 'SNIC');

% Add line handles to the legend array

legend_handles = [legend_handles, h5, h9, h12, h13, h14, h15];

legend_names = [legend_names, 'Fold Limit Cycle', 'SH', 'SN', 'SubH', 'SupH', 'SNIC'];

end

% Add the sphere mesh with transparency

surf(X_sphere, Y_sphere, Z_sphere, 'FaceColor', [0.96, 0.96, 0.86], 'FaceAlpha', 0.2, 'EdgeColor', 'none', 'HandleVisibility', 'off');

% Add labels and view adjustment

xlabel('\mu_2');

ylabel('-\mu_1');

zlabel('\nu');

lineVector = [-0.19, 0.2, 0.07];

az = atan2d(lineVector(2), lineVector(1)); % Azimuth angle

el = atan2d(lineVector(3), norm(lineVector(1:2))); % Elevation angle

view(az, el);

% Display the legend with all handles and names

legend(legend_handles, legend_names);

end

function [onset_curve,offset_curve]=hysteresis_random_path(bifurcation)

load('curves.mat');

if bifurcation == '2s'

onset_curve=SNr_LCs;

offset_curve=SHl;

end

if bifurcation=='2b'

onset_curve=SNr_LCb;

offset_curve=SHb;

end

if bifurcation=='3s'

onset_curve=SNr_LCs;

offset_curve=SNl_ActiveRest;

end

if bifurcation=='4b'

onset_curve=SNr_LCb;

offset_curve=FLC_top;

end

if bifurcation=='10'

onset_curve=SNr_ActiveRest;

offset_curve=SHl;

end

if bifurcation=='11'

onset_curve= SNr_ActiveRest;

offset_curve=[[0.3171; -0.066; 0.2347], [0.3115; -0.0546; 0.2450],[0.3166; -0.0654; 0.2356]];%SNl_ActiveRest;

end

if bifurcation=='14'

onset_curve=subH;

offset_curve=SHb;

end

if bifurcation=='16'

onset_curve=subH;

offset_curve=FLC;

end

end

function [onset_curve,offset_curve,offset_curve2, flag] = slow_wave_circular_random_path(I)

load('curves.mat');

load('curves2.mat');

if I == 6

onset_curve = SHl(:,50:104); %55

offset_curve = [0.33, 0.11, 0.18]';

offset_curve2 = SNIC(:,1:35); %35

flag = 1;

end

if I == 5

onset_curve = SNIC; %44

offset_curve = SNIC; %44

offset_curve2 = [0.34,0.14,0.06]';

flag = 2;

end

if I == 1

onset_curve = SNIC(:,1:35); %35

offset_curve = [0.33, 0.11, 0.18]';

offset_curve2 = SNr_ActiveRest; %55

flag = 1;

end

if I == 7

onset_curve=SNIC; %44

offset_curve = Hopf(:,800:855); %56

offset_curve2 = [0.36,-0.12,0.12]';

flag = 3;

end

if I == 8

onset_curve=SNIC; %44

offset_curve = [0.34,0.2,-0.06]';

offset_curve2 = FLC(:,100:300); %201

flag = 1;

end

if I == 9

onset_curve= Hopf(:,800:855); %56

offset_curve = SNIC; %44

offset_curve2 = [0.36,-0.12,0.12]';

flag = 3;

end

if I == 12

onset_curve= Hopf(:,450:495);

offset_curve = FLC(:,60:100);

offset_curve2 = [-0.3, -0.2, -0.2]';

flag = 3;

end

if I == 13

onset_curve = Hopf(:,450:495); %201

offset_curve = [0.34,0.2,-0.06]';

offset_curve2 = SNIC;

flag = 1;

end

if I == 15

onset_curve= subH;%41

offset_curve = Hopf(:,450:495);%46

offset_curve2 = [-0.3, -0.2, -0.2]';

flag = 3;

end

end

function [Xdot, mu2,mu1,nu] = SlowWave_Model_piecewise(~,x,~,k,k_fast, alpha, mu2,mu1,nu)

% Parametrization of the path in the spherical parameter space in terms

% of a circle defined by 3 points

% System

xdot = -k_fast* alpha*x(2);

ydot = -k_fast*(-(x(1)/alpha)^3 +mu2*(x(1)/alpha) +mu1 + x(2)*( nu + (x(1)/alpha) + (x(1)/alpha)^2));

zdot = k;

Xdot = [xdot;ydot;zdot];

end

function [mu2,mu1,nu, theta] = sphereArcPath(k, tstep,point1, point2)

% sphereArcPath - Generates an arc path between two points on a sphere

%

% Syntax: arcPath = sphereArcPath(point1, point2, numPoints)

%

% Inputs:

% point1 - [x1, y1, z1] Coordinates of the first point on the sphere

% point2 - [x2, y2, z2] Coordinates of the second point on the sphere

% numPoints - Number of points along the arc

%

% Outputs:

% arcPath - An Nx3 matrix containing the coordinates of points along the arc

% Check the input points

radius = 0.4;

% if norm(point1) ~= radius || norm(point2) ~= radius

% error('The points must lie on the sphere of radius 0.4.');

% end

% Normalize the input points to make sure they are on the sphere

point1 = point1 / norm(point1) * radius;

point2 = point2 / norm(point2) * radius;

% Compute the quaternion for rotation

theta = acos(dot(point1, point2) / (radius^2));

axis = cross(point1, point2);

if norm(axis) == 0

error('The points are the same or antipodal.');

end

axis = axis / norm(axis);

% Compute points along the arc

numPoints = floor((theta/k)/tstep);

arcPath = zeros(numPoints, 3);

for i = 0:numPoints-1

t = i / (numPoints - 1);

angle = t * theta;

R = rotationMatrix(axis, angle);

arcPath(i+1, :) = (R * point1')';

end

mu2 = arcPath(:,1)';

mu1 = arcPath(:,2)';

nu = arcPath(:,3)';

end

function R = rotationMatrix(axis, angle)

% rotationMatrix - Generates a rotation matrix given an axis and an angle

%

% Syntax: R = rotationMatrix(axis, angle)

%

% Inputs:

% axis - A 3-element vector representing the axis of rotation

% angle - The angle of rotation in radians

%

% Outputs:

% R - A 3x3 rotation matrix

ux = axis(1);

uy = axis(2);

uz = axis(3);

c = cos(angle);

s = sin(angle);

t = 1 - c;

R = [t*ux*ux + c, t*ux*uy - s*uz, t*ux*uz + s*uy;

t*ux*uy + s*uz, t*uy*uy + c, t*uy*uz - s*ux;

t*ux*uz - s*uy, t*uy*uz + s*ux, t*uz*uz + c];

end

function point= get_random_point

radius = 0.4;

% Generate two random numbers

theta = 2 * pi * rand(); % Random angle between 0 and 2*pi

phi = acos(2 * rand() - 1); % Random angle between 0 and pi

% Convert spherical coordinates to Cartesian coordinates

x = radius * sin(phi) * cos(theta);

y = radius * sin(phi) * sin(theta);

z = radius * cos(phi);

point = [x,y,z];

% Display the point

end

function point= get_random_point_hopf

%load("map_regions.mat")

radius = 0.4;

% Loop until a valid point with y > 0 is found

while true

% Generate two random numbers

theta = 2 * pi * rand(); % Random angle between 0 and 2*pi

phi = acos(2 * rand() - 1); % Random angle between 0 and pi

% Convert spherical coordinates to Cartesian coordinates

x = radius * sin(phi) * cos(theta);

y = radius * sin(phi) * sin(theta);

z = radius * cos(phi);

% Check if y is positive

if y > 0

point = [x, y, z];

break;

end

end

% Display the point

end

%

% Display the point

function point= get_random_point_fixed

radius = 0.4;

% Loop until a valid point with y > 0 is found

while true

% Generate two random numbers

theta = 2 * pi * rand(); % Random angle between 0 and 2*pi

phi = acos(2 * rand() - 1); % Random angle between 0 and pi

% Convert spherical coordinates to Cartesian coordinates

x = radius * sin(phi) * cos(theta);

y = radius * sin(phi) * sin(theta);

z = radius * cos(phi);

% Check if y is positive

if y < 0

point = [x, y, z];

break;

end

end

% Display the point

end

function [mu2,mu1,nu] = sphere(point1, point2, numPoints)

% sphereArcPath - Generates an arc path between two points on a sphere

%

% Syntax: arcPath = sphereArcPath(point1, point2, numPoints)

%

% Inputs:

% point1 - [x1, y1, z1] Coordinates of the first point on the sphere

% point2 - [x2, y2, z2] Coordinates of the second point on the sphere

% numPoints - Number of points along the arc

%

% Outputs:

% arcPath - An Nx3 matrix containing the coordinates of points along the arc

% Check the input points

radius = 0.4;

% if norm(point1) ~= radius || norm(point2) ~= radius

% error('The points must lie on the sphere of radius 0.4.');

% end

% Normalize the input points to make sure they are on the sphere

point1 = point1 / norm(point1) * radius;

point2 = point2 / norm(point2) * radius;

% Compute the quaternion for rotation

theta = acos(dot(point1, point2) / (radius^2));

axis = cross(point1, point2);

if norm(axis) == 0

error('The points are the same or antipodal.');

end

axis = axis / norm(axis);

% Compute points along the arc

arcPath = zeros(numPoints, 3);

for i = 0:numPoints-1

t = i / (numPoints - 1);

angle = t * theta;

R = rotationMatrix(axis, angle);

arcPath(i+1, :) = (R * point1')';

end

mu2 = arcPath(:,1)';

mu1 = arcPath(:,2)';

nu = arcPath(:,3)';

end

function [arcLength, theta] = calculateArcLength(P1, P2, radius)

% calculateArcLength computes the arc length and central angle between two points on a sphere.

%

% Input:

% P1 - First point [x1, y1, z1]

% P2 - Second point [x2, y2, z2]

% radius - Radius of the sphere (default: 0.4 if not provided)

%

% Output:

% arcLength - Arc length between the two points

% theta - Central angle between the two points in radians

if nargin < 3

radius = 0.4;

end

% Compute the dot product of P1 and P2

dotProduct = dot(P1, P2);

% Compute the magnitudes of P1 and P2

magnitudeP1 = norm(P1);

magnitudeP2 = norm(P2);

% Compute the cosine of the central angle

cosTheta = dotProduct / (magnitudeP1 * magnitudeP2);

% Compute the central angle in radians

theta = acos(cosTheta);

% Compute the arc length

arcLength = radius * theta;

end

function [p0,p1,p1_5,p2,p3]=piecewise_random_path(bifurcation)

load('curves.mat');

load('bifurcation_crossing.mat')

load("curves2.mat")

if bifurcation==3

%fixed rest point

p0 = Hopf(:,930)';

%bifurcation curve

randomNumber = randi([145,170]);

p1 = Fold(:,145:170)';

randomNumber2 = randi([600,750]);

p1_5 = [0.3196, 0.2389, -0.0279];

%bifurcation curve

p2 = Hopf(:,600:750)' ;

%fixed rest

p3 = [ 0.1944 , 0.0893 , 0.3380];

end

if bifurcation==7

randomNumber = randi([600,750]);

randomNumber2 = randi([1,44]);

%fixed rest point

p0 = Fold(:,400)';

%bifurcation curve

p1 = SNIC' ;

%random point in limit cycle

p1_5 = [0.1314, 0.3298, -0.1843];

%bifurcation curve

p2 = Hopf(:,600:750)';

%fixed rest

p3 = [ 0.1944 , 0.0893 , 0.3380];

end

if bifurcation==9

randomNumber = randi([600,750]);

%fixed rest point

p0 = [ 0.1944 , 0.0893 , 0.3380];

%bifurcation curve

p1 = Hopf(:,600:750)';

%change here

randomNumber2 = randi([1,44]);

%random point in limit cycle

p1_5 = [-0.0441, 0.2591, -0.3015];

%bifurcation curve

p2 = SNIC' ;

%fixed rest

p3 = Fold(:,450)';

end

if bifurcation==10

randomNumber = randi([600,750]);

%fixed rest point

p0 = [ 0.1944 , 0.0893 , 0.3380];

%bifurcation curve

p1 = Hopf(:,600:750)';%get_nearest_hopf(p0(1),p0(2),p0(3))';

%change here

randomNumber2 = randi([1,124]);

%random point in limit cycle

p1_5 = [-0.123686721647726 0.338825918756816 -0.172912092308889];

%bifurcation curve

p2 = Homoclinic_to_saddle' ;

%fixed rest

p3 = Fold(:,400)';

end

if bifurcation==11

randomNumber = randi([600,750]);

%fixed rest point

p0 = [ 0.1944 , 0.0893 , 0.3380];

%bifurcation curve

p1 = Hopf(:,600:750)';

%change here

randomNumber2 = randi([600,750]);

%random point in limit cycle

p1_5 = [-0.2104, 0.3180, -0.1209];

%bifurcation curve

p2 = Hopf(:,600:750)' ;

%fixed rest

p3 = [ 0.1944 , 0.0893 , 0.3380];

end

end

function [new_X, new_Y, new_Z] = get_random_walk(p1,p2,region,k,tstep, minSteps, maxSteps)

load('logicgrid.mat')

% if region == 11

% load('logicgrid.mat'); % Load the logic grid from the .mat file

%

% elseif region == 'LCS_bistable'

% load("logicgrid_bistable_lcs.mat")

%

% elseif region == 'LCB_bistable'

% load("logicgrid_bistable_lcb.mat")

% end

grid = logic_grid;

[theta, phi, r] = cart2sph(p1(1), p1(2), p1(3));

theta_offset_deg = -40; % For example, a 30-degree rotation

% Convert the offset to radians

theta_offset_rad = deg2rad(theta_offset_deg);

theta_deg_p1 = rad2deg(theta+theta_offset_rad);

phi_deg_p1 = rad2deg(phi);

p1_5 = get_nearest_seizure_point(theta_deg_p1, phi_deg_p1, region);

[theta, phi, r] = cart2sph(p2(1), p2(2), p2(3));

theta_offset_deg = -40; % For example, a 30-degree rotation

% Convert the offset to radians

theta_offset_rad = deg2rad(theta_offset_deg);

theta_deg_p2 = rad2deg(theta+theta_offset_rad);

phi_deg_p2 = rad2deg(phi);

p2_5 = get_nearest_seizure_point(theta_deg_p2, phi_deg_p2, region);

% Load the grid (assuming the variable is 'grid')

% Define the start and end points

startPoint = p1_5; % Define the start point (row, column)

endPoint = p2_5; % Define the end point (row, column)

currentPos = startPoint;

%

% % Store the path

path = currentPos;

%

% % Define the movement directions (up, down, left, right)

directions = [0 1; 0 -1; 1 0; -1 0]; % [row_change, col_change]

% Get the size of the grid

[gridRows, gridCols] = size(grid);

% Loop until a valid path is found

% Loop until a valid path is found

while true

% Initialize the current position at the start point

currentPos = startPoint;

% Store the path, starting from the initial position

path = currentPos;

stepCount = 0;

% Perform the random walk until either endpoint is reached or maxSteps

while stepCount < maxSteps

% Check if the endpoint has been reached and steps are within bounds

if isequal(currentPos, endPoint) && stepCount >= minSteps

disp('Valid path found within the specified range of steps.');

break;

end

% Choose a random direction to move

randomDir = directions(randi(4), :);

% Compute the new position

newPos = currentPos + randomDir;

% Check if the new position is within bounds and valid (e.g., grid value is 1)

if newPos(1) > 0 && newPos(1) <= gridRows && newPos(2) > 0 && newPos(2) <= gridCols

if grid(newPos(1), newPos(2)) == 1

% Update the current position

currentPos = newPos;

% Add the new position to the path

path = [path; currentPos];

% Increment the step count

stepCount = stepCount + 1;

end

end

end

% Check if a valid path was found within bounds

if isequal(currentPos, endPoint) && stepCount >= minSteps && stepCount <= maxSteps

% Exit the loop if a valid path is found

break;

end

% If not, repeat the simulation

end

% Plot the random walk path

figure;

imagesc(grid); % Show the grid

colormap(gray); % Use a gray color map (1 = white, 0 = black)

hold on;

plot(path(:,2), path(:,1), 'r.-', 'LineWidth', 2, 'MarkerSize', 15); % Plot the path

% Plot the start point in green

plot(startPoint(2), startPoint(1), 'go', 'MarkerSize', 10, 'MarkerFaceColor', 'g');

% Plot the end point in blue

plot(endPoint(2), endPoint(1), 'bo', 'MarkerSize', 10, 'MarkerFaceColor', 'b');

title('2D Random Walk Path on the Logic Grid');

xlabel('X');

ylabel('Y');

if region == 11

load('logicgrid.mat'); % Load the logic grid from the .mat file

elseif region == 'LCS_bistable'

load("logicgrid_bistable_lcs.mat")

elseif region == 'LCB_bistable'

load("logicgrid_bistable_lcb.mat")

end

% Convert back to x, y coordinates

x_back = (path(:,1) - 1) / (grid_size(1) - 1) * (x_max - x_min) + x_min;

y_back = (path(:,2) - 1) / (grid_size(2) - 1) * (y_max - y_min) + y_min;

theta_rad = deg2rad(x_back) + deg2rad(-theta_offset_deg);

phi_rad = deg2rad(y_back);

radius = 0.4; % Set the radius of the sphere

[X, Y, Z] = sph2cart(theta_rad, phi_rad, radius);

r = 0.4;

P1 = [X(1);Y(1);Z(1)];

P2 = [X(2);Y(2);Z(2)];

% Compute the angle between the two points using the dot product formula

cos_theta = dot(P1, P2) / (r^2);

theta = acos(cos_theta); % Angle in radians

total_theta = (length(X)-1)*theta;

numPoints = floor((total_theta/k)/tstep);

% Create interpolation queries and interpolate X, Y, Z coordinates

xq = linspace(1, length(X), numPoints); % Query points for interpolation

new_X = interp1(1:length(X), X, xq, 'linear');

new_Y = interp1(1:length(Y), Y, xq, 'linear');

new_Z = interp1(1:length(Z), Z, xq, 'linear');

end

function noisy_signal = add_pink_noise(signal, rms_signal, noise_amplitude_ratio, fs)

% Inputs:

% signal - input signal (1D array)

% noise_amplitude_ratio - fraction of signal amplitude for noise (e.g., 0.4 for 40%)

% Compute the RMS amplitude of the signal

% Generate pink noise of the same length as the signal

% Pink noise can be generated using dsp.ColoredNoise in MATLAB

L = length(signal);

pink_noise = pinknoise([1,L],-1,10000)';

%

% % % Scale the noise so its amplitude is noise_amplitude_ratio of the signal's amplitude

% scaling_factor = noise_amplitude_ratio * (1 / rms_noise);

% scaled_noise = pink_noise * scaling_factor;

min_val = min(pink_noise(:));

max_val = max(pink_noise(:));

scaled_noise = noise_amplitude_ratio*(pink_noise - min_val) / (max_val - min_val);

min_val = min(signal(:));

max_val = max(signal(:));

scaled_signal = (signal - min_val) / (max_val - min_val);

% Add the scaled noise to the original signal

noisy_signal = scaled_signal + scaled_noise;

end

function amp = get_amp(signal, fs)

hpFilt = designfilt('highpassfir', 'FilterOrder', 5, ...

'CutoffFrequency', 5, 'SampleRate', fs);

signal= filter(hpFilt, signal);

[peaks,locs] = findpeaks(signal ,'MinPeakProminence', 0.15);

[troughs_neg,locs_troughs] = findpeaks(signal, 'MinPeakProminence', 0.15);

troughs = -1*troughs_neg;

newnew = [];

len = 0;

if length(troughs) > length(peaks)

len = length(peaks);

else

len = length(troughs);

end

for i = 1:len

newnew = [newnew; -1*troughs(i) + peaks(i)];

end

amp = mean(newnew);

end

function [start_index, stop_index, signal] = bifurcation_all_class(class, tstep, sigma)

R = 1;

N = 1;

b = 0;

x0 = [0;0;0];

k =0.007;

dstar = 0.3;

tstep = 0.1;

tmax = 75000;

if ismember(class, [1 5 6 8 12 13 15])

% Code for class 1

% Perform specific operations for class 1

[onset_curve,offset_curve,p3, flag] = slow_wave_circular_random_path(class);

onset_curve_length=length(onset_curve);

offset_curve_length=length(offset_curve);

random_onset_index=randsample(onset_curve_length,1);

random_offset_index=randsample(offset_curve_length,1);

if flag == 2 || flag == 3

p1 = onset_curve(:,random_onset_index);

p2 = offset_curve(:,random_offset_index);

else

p1 = onset_curve(:,random_onset_index);

p2 = p3(:,random_offset_index);

p3 = offset_curve;

end

% uncomment this code to do random path

% % One random path - select random point on onset curve and offset curve

tspan = 0:tstep:tmax;

% Create circular path based 3 defining points

[E, F, C, r] = Parametrization_3PointsCircle(p1',p2',p3');

if class == 13

E = -E;

end

N_t = length(tspan);

X = zeros(3,N_t);

xx = x0;

sigma = 40;

Rn = [pinknoise([1,N_t],-1, sigma);pinknoise([1,N_t],-1, 00);pinknoise([1,N_t],-1, 00)];

mu2_big = zeros(1, length(N_t));

mu1_big = zeros(1, length(N_t));

nu_big = zeros(1, length(N_t));

for n = 1:N_t

%Euler-Meruyama method

[Fxx, mu2, mu1,nu] = SlowWave_Model(tspan(n),xx,b,k,E,F,C,r);

xx = xx + tstep*Fxx + sqrt(tstep)*Rn(:,n);

X(:,n) = xx;

mu2_big(n) = mu2;

mu1_big(n) = mu1;

nu_big(n) = nu;

end

x = X';

t = tspan;

%%Onset and offset calculation, calculates radians to the bifurcation

%%curve, then uses tstep and k variables to compute onset location

plot_onset_offset = 0;

if(floor((((2*pi)/k)/tstep)) < N_t)

plot_onset_offset = 1;

point1 = p1' - C;

point2 = p2' - C;

point3 = p3'-C;

point1 = point1 / norm(point1) * r;

point2 = point2 / norm(point2) * r;

point3 = point3 / norm(point3) * r;

% Compute the quaternion for rotation

theta1 = acos(dot(point1, point2) / (r^2));

%%%change here

numPoints1 = floor((theta1/k)/tstep);

point = [mu2_big(numPoints1), -mu1_big(numPoints1), nu_big(numPoints1)];

if round(point,2) == round(p2,2)'

onset_index = numPoints1;

else

numPoints1 = floor(((2*pi - theta1)/k)/tstep);

onset_index = numPoints1;

theta1 = theta1-2*pi;

end

theta2 = acos(dot(point1, point3) / (r^2));

numPoints2 = floor(((theta2)/k)/tstep);

point = [mu2_big(numPoints2), -mu1_big(numPoints2), nu_big(numPoints2)];

if round(point,2) == round(p2,2)'

%offset_index = numPoints2;

else

numPoints2 = floor(((2*pi - theta2)/k)/tstep);

theta2 = 2*pi - theta2;

offset_index = numPoints2;

end

theta3 = 2*pi;

numPoints3 = floor(((theta3)/k)/tstep);

point = [mu2_big(numPoints3), -mu1_big(numPoints3), nu_big(numPoints3)];

offset_index = numPoints3;

end

if class == 15 || class == 12

onset_index_temp = onset_index;

onset_index = offset_index;

offset_index = onset_index_temp + floor((((2*pi)/k)/tstep));

end

start_index = max(1,onset_index-1000);

stop_index = min(offset_index+1000, length(x));

signal = x(start_index:stop_index,1);

elseif ismember(class, ['2s' '2b' '4b' '14' '16'])

k=0.01;

tmax = 75000;

dstar = 0.3;

[onset_curve,offset_curve]=hysteresis_random_path(class);

onset_curve_length=length(onset_curve);

offset_curve_length=length(offset_curve);

% uncomment this code to do random path

% % One random path - select random point on onset curve and offset curve

random_onset_index=randsample(onset_curve_length,1);

random_offset_index=randsample(offset_curve_length,1);

A = offset_curve(:,random_offset_index);

B = onset_curve(:,random_onset_index);

tspan = 0:tstep:tmax;

% Create circular path based 3 defining points

[E, F] = Parametrization_2PointsArc(A,B,R);

N_t = length(tspan);

X = zeros(3,N_t);

xx = x0;

Rn = [pinknoise([1,N_t],-1, sigma);pinknoise([1,N_t],-1, 00);pinknoise([1,N_t],-1, 00)];

for n = 1:N_t

% Euler-Meruyama method

Fxx = HysteresisLoop_Model(tspan(n),xx,b,k,R,dstar,E,F,N);

xx = xx + tstep*Fxx + sqrt(tstep)*Rn(:,n);

X(:,n) = xx;

end

x = X';

[pks,times]=findpeaks(x(:,3),'MinPeakProminence',0.03);

onset_time = times*tstep;

% Calculate Offset Times

[pks2,times2]=findpeaks(-x(:,3),'MinPeakProminence',0.03);

offset_time = times2*tstep;

% Single seizure

if offset_time(1)>onset_time(1) % if system starts at rest

start_index = times(1)-10000;

stop_index = times2(1)+10000;

start_index = max(1, start_index);

stop_index = min(length(x), stop_index);

signal = x(start_index:stop_index,1);

onset = times(1);

offset = stop_index-start_index-10000;

else % if system starts in a seizure

start_index = times(1)-10000;

stop_index = times2(2)+10000;

start_index = max(1, start_index);

stop_index = min(length(x), stop_index);

signal = x(start_index:stop_index,1);

onset = times(1);

offset = stop_index-start_index-10000;

end

elseif ismember(class, [3 7 9 10 11])

k = 0.005;

[p0,onset_curve,p1_5,offset_curve,p3]=piecewise_random_path(class);

onset_curve_length=length(onset_curve);

offset_curve_length=length(offset_curve);

onset_curve = onset_curve';

offset_curve = offset_curve';

% uncomment this code to do random path

% % One random path - select random point on onset curve and offset curve

random_onset_index=randsample(onset_curve_length,1);

random_offset_index=randsample(offset_curve_length,1);

p1 = onset_curve(:,random_onset_index)';

p2 = offset_curve(:,random_offset_index)';

stall_val = 30000;

[mu2_straight_path0,mu1_straight_path0,nu_straight_path0,rad1] = sphereArcPath(k,tstep,p0,p1);

[mu2_straight_path0_5,mu1_straight_path0_5,nu_straight_path0_5,rad2] = sphereArcPath(k,tstep,p1,p1_5);

points = repmat(p1_5, stall_val, 1)';

%path noise sigma

sigma_pathnoise = 100;

Rn = [pinknoise([1,length(points)],-1, sigma_pathnoise);pinknoise([1,length(points)],-1, sigma_pathnoise);pinknoise([1,length(points)],-1, sigma_pathnoise)];

points = points + Rn;

[mu2_straight_path,mu1_straight_path,nu_straight_path,rad3] = sphereArcPath(k,tstep,p1_5,p2);

[mu2_straight_path1,mu1_straight_path1,nu_straight_path1,rad4] = sphereArcPath(k,tstep,p2,p3);

mu2_all = [mu2_straight_path0, mu2_straight_path0_5, points(1, :), mu2_straight_path, mu2_straight_path1];

mu1_all = [mu1_straight_path0, mu1_straight_path0_5, points(2, :), mu1_straight_path, mu1_straight_path1];

mu1_all = -mu1_all;

nu_all = [nu_straight_path0, nu_straight_path0_5, points(3,:), nu_straight_path, nu_straight_path1];

N_t = length(mu2_all);

X = zeros(3,N_t);

xx = x0;

Rn = [pinknoise([1,N_t],-1, sigma);pinknoise([1,N_t],-1, 00);pinknoise([1,N_t],-1, 00)];

mu2_big = zeros(1, length(N_t));

mu1_big = zeros(1, length(N_t));

nu_big = zeros(1, length(N_t));

%%get onset index by finding Radians to bifurcation, and getting index

%%through k and tstep parameters

onset_index = floor((rad1/k)/tstep);

offset_index = floor(((rad1+rad2+rad3)/k)/tstep) + stall_val;

for n = 1:N_t

%%Euler-Meruyama method

[Fxx,mu2,mu1,nu] = SlowWave_Model_piecewise(0,xx,b,k,mu2_all(n), mu1_all(n),nu_all(n));

xx = xx + tstep*Fxx + sqrt(tstep)*Rn(:,n);

X(:,n) = xx;

mu2_big(n) = mu2;

mu1_big(n) = mu1;

nu_big(n) = nu;

end

x = X';

signal = x(:,1);

start_index = 1;

stop_index = length(signal);

end

end

function x_rs=eval_resting_state_cartesian(a,mu2,mu1,N)

switch N

case 1 % resting state

x_rs = ((a.^3 .* mu1)./2 + ((a.^6 .* mu1.^2)./4 - (a.^6 .* mu2.^3)./27).^(1/2)).^(1/3) + ...

(a.^2 .* mu2) ./ (3 .* ((a.^3 .* mu1)./2 + ((a.^6 .* mu1.^2)./4 - (a.^6 .* mu2.^3)./27).^(1/2)).^(1/3));

case 2

x_rs = - (sqrt(3) .* (((a.^3 .* mu1)./2 + ((a.^6 .* mu1.^2)./4 - (a.^6 .* mu2.^3)./27).^(1/2)).^(1/3) - (a.^2 .* mu2) ./ (3 .* ((a.^3 .* mu1)./2 + ((a.^6 .* mu1.^2)./4 - (a.^6 .* mu2.^3)./27).^(1/2)).^(1/3))) .* 1i) ./ 2 ...

- ((a.^3 .* mu1)./2 + ((a.^6 .* mu1.^2)./4 - (a.^6 .* mu2.^3)./27).^(1/2)).^(1/3) ./ 2 ...

- (a.^2 .* mu2) ./ (6 .* ((a.^3 .* mu1)./2 + ((a.^6 .* mu1.^2)./4 - (a.^6 .* mu2.^3)./27).^(1/2)).^(1/3));

case 3

x_rs = (sqrt(3) .* (((a.^3 .* mu1)./2 + ((a.^6 .* mu1.^2)./4 - (a.^6 .* mu2.^3)./27).^(1/2)).^(1/3) - (a.^2 .* mu2) ./ (3 .* ((a.^3 .* mu1)./2 + ((a.^6 .* mu1.^2)./4 - (a.^6 .* mu2.^3)./27).^(1/2)).^(1/3))) .* 1i) ./ 2 ...

- ((a.^3 .* mu1)./2 + ((a.^6 .* mu1.^2)./4 - (a.^6 .* mu2.^3)./27).^(1/2)).^(1/3) ./ 2 ...

- (a.^2 .* mu2) ./ (6 .* ((a.^3 .* mu1)./2 + ((a.^6 .* mu1.^2)./4 - (a.^6 .* mu2.^3)./27).^(1/2)).^(1/3));

end

end
