# Supplementary material for: Dynamotypes for Dummies: A Toolbox, Atlas, and Tutorial for Simulating a Comprehensive Range of Realistic Synthetic Seizures
Source: eNeuro. 2025 Oct 15;12(10):ENEURO.0200-25.2025. doi: 10.1523/ENEURO.0200-25.2025 (PMC12549069; doi:10.1523/ENEURO.0200-25.2025)
Supplement: Data 1 — Microsoft Word and PDF versions of the Matlab Live “Dynamotypes for Dummies Tutorial.” Download Data 1, ZIP file. [file eneuro-12-ENEURO.0200-25.2025-s002.zip › Dynamotypes_for_dummies_tutorial.pdf]

Dynamotypes for Dummies Model Walkthrough

Matlab Live Editor for **Dynamotypes for Dummies: A toolbox, atlas, and tutorial for simulating a comprehensive range of realistic synthetic seizures**

Christina Sheckler, Kathleen Kish, Zion Walker, Grant Barkelew, Matt Szuromi, Marisa Saggio, Dakota Crisp, William Stacey

Contact info: William Stacey (William.stacey@umich.edu), Christina Sheckler (ninashec@umich.edu), Kathleen Kish (katfin@umich.edu)

```
clear all
addpath('Walkthrough helper files');
```

Literature

Before we give a brief walkthrough of the model, we would like to list out resources that may be useful in learning the ins and outs of the model. We understand that people interested in the model may have varying degrees of mathematical proficiency. While it is not necessary to have a deep rigorous understanding of all the math involved in the construction and employment of this model, we do think that having some experience with differential equations and linear algebra will greatly improve the reader's ability to understand and utilize the model.

Here is the list of useful materials:

Using unfoldings of high codimension singularities to model fast-slow bursters (i.e. the approach used in this model):

- Bertram, Richard, et al. "Topological and phenomenological classification of bursting oscillations." *Bulletin of mathematical biology* 57.3 (1995): 413-439. **Uses these unfoldings for bursting**
- Izhikevich, Eugene. "Neural Excitability, Spiking, and Bursting." 2000. **A very accessible introduction to bursting, uses some unfoldings; establishes the taxonomy of fast-slow bursters**
- Golubitsky, Martin, Kresimir Josic, and Tasso J. Kaper. "An unfolding theory approach to bursting in fast–slow systems." *Global analysis of dynamical systems*. CRC Press, 2001. 282-313. **Formalizes and extends the approach of Bertram et al**
- Saggio, Maria Luisa et al. "Fast-Slow Bursting in the Unfolding of a High Codimension Singularity and the Ultra-Slow Transitions of Classes." 2017. **Systematically extends the work of Golubitsky et al to codimension 3 and proposes models for all classes of Izhikevich's taxonomy (planar only)**

Using fast-slow bursters to classify and model seizures

- Jirsa, Viktor et al. "On the Nature of Seizure Dynamics." 2014. **Extends Izhikevich taxonomy to seizures and proposes one type of burster to model the most common seizure type**
- Saggio, Maria Luisa et al. "A Taxonomy of Seizure Dynamotypes." 2020. **Extends the approach of Jirsa et al to the full (planar) taxonomy, using the model in the Saggio et al 2017**
- Crisp, Dakota et al. "Quantifying Epileptogenesis in Rats with Spontaneous and Responsive Brain State Dynamics." 2020. **Application of the taxonomy**
- Saggio, Maria Luisa & Jirsa, Viktor. "Phenomenological Mesoscopic Models for Seizure Activity." 2022. **A review**
- Depannemaecker, Damien et al. "A unified physiological framework of transitions between seizures, sustained ictal activity and depolarization block at the single neuron level." 2022. **It also maps a new neural model to the unfolding used in Saggio et al 2017**
- Szuromi, Matthew et. al. "Optimization of Ictal Aborting Stimulation Using the Dynamotype Taxonomy." *2023.* **Application of the model to stimulation**

If the reader is new to nonlinear dynamics, we highly suggest they start by reading Strogatz, Steven. *Nonlinear Dynamics and Chaos.*. Particularly, Parts I & II of the book. Part III is on chaos and does not apply. If one is in a rush, they could probably skip all of Chapter 4 and Sections 3.6-3.7, 6.5-6.8, 7.2-7.6, and 8.6-8.7.

Next, we suggest reading Izhikevich (2000), Jirsa (2014), Saggio (2017), and Saggio (2020) in chronological order. You could skip Izhikevich, but we think even a cursory reading helps set the stage well for the following papers. It also gives great visualizations.

The other papers are supplementary and discuss a range of uses and applications of the model. These may be interesting depending on what the reader intends to use the model for.

Finally, if the reader would like to understand the theory behind the construction of the model in Saggio (2017), they should read Bertram and Golubitsky.

Brief introduction to dynamical theory

Dynamical systems are systems whose state changes over time according to a defined rule. In the context of biological modeling, dynamical systems are most often realized in two forms: discrete maps and differential equations. Differential equations can also be ordinary or partial. For this work, we utilize ordinary differential equations (ODEs), to which we will restrict this introduction.

First-order ODEs consist of equations which relate the time derivative of an indpendent state variable (which may be vector valued) to the current state of the system and possibly external time-dependent forcing. Our core model contains no external forcing. Then general form is then:

$$\frac{d\mathbf{x}}{dt} = \mathbf{f}(\mathbf{x})$$

Given starting values for the components of the state variable, known as the initial conditions, the solutions of the ODEs prescribe a unique trajectory that describes the evolution of the state variables, beginning at the initial condition. Explicit solutions to ODEs are highly desirable as they tell us the exact value of the state variable at every time point. However, ODEs can be complex and their solutions highly nontrivial. This motivates the so-called qualitative study of ODEs.

Long-Term Behavior, Attractors, and Stability

The qualitative theory of ODEs is concerned with describing the long-term behavior of the dynamical system. Does the system "settle down" at a certain value? Does it persistently oscillate? Does it exhibit chaos? These questions motivate the first term of interest, a **stable attractor.** Attractors are most generally understood as a subset of possible values of the state variable towards which the system evolves. The attractor is stable in the since that, given a trajectory initialized on the attractor, a sufficently small, arbitrary perturbation away from the attractor results in the system returning to the attractor. Conversely, a set of states may be **unstable**, in which case small perturbations result in trajectories which flow away from that set. In this case, we may also call this a **repeller.**

The two types of attractors pertinent to this work are **stable fixed points** and **stable limit cycles**. So-called chaotic or strange attractors are explicitly dissallowed in our model, as they only exist in systems of 3 or more dimensions, while our fast-subsystem is only 2D. Further, degenerate solutions like line attractors or centers are not relevent to the present work.

First, fixed points are single states at which the system remains for all time when intitialized there. Mathematically, these are disconnected points at which $\frac{d\mathbf{x}}{dt} = \mathbf{0}$. Thus, fixed points are solutions of $\mathbf{f}(\mathbf{x}) = \mathbf{0}$. Writing this in components $f_i(\mathbf{x}) = 0$, for all components $1 \leq i \leq n$. For a single fixed i, the set of states that solves $f_i(\mathbf{x}) = 0$ is called a **nullcline.**

Thus, fixed points are the intersections of the n nullclines. Fixed points can be either stable, unstable or **saddles**. Stability of a fixed point is assessed by evaluating the **Jacobian matrix** at the fixed point. The Jacobian is given by

$$'J = \begin{bmatrix} \frac{\partial f_1}{\partial x_1} & \frac{\partial f_1}{\partial x_2} & \cdots & \frac{\partial f_1}{\partial x_n} \\ \frac{\partial f_2}{\partial x_1} & \frac{\partial f_2}{\partial x_2} & \cdots & \frac{\partial f_2}{\partial x_n} \\ \vdots & \vdots & \ddots & \vdots \\ \frac{\partial f_n}{\partial x_1} & \frac{\partial f_n}{\partial x_2} & \cdots & \frac{\partial f_n}{\partial x_n} \end{bmatrix}'$$

The Jacobian evaluated at the fixed point describes the growth or decay of small perturbation. A fixed point is stable when the real parts of the **eigenvalues** of the Jacobian are all negative. A fixed point is unstable if the real parts of all eigenvalues are positive. A fixed point with a mix of eigenvalues with positive and negative real parts is a saddle. If the eigenvalues are all real, the fixed point is known as **nodes**. If eigenvalues are complex, then we call the fixed point a **spiral**.

Limit cycles are periodic solutions of ODEs, $x(t)$, such that $x(t) = x(t + T)$ for some finite period T. Limit cycles can also be stable or unstable. The stability of limit cycles is assessed via **Floquet Theory**, but its development is involved, and we refrain from doing so in this work.

Stable Fixed point	A point in a dynamical system where, if the system is slightly perturbed in any direction, it will return to that point.	Like a marble at the bottom of a bowl—no matter where you nudge it, it will roll back to the bottom. Stable because it always returns to the same spot.
Unstable fixed point	An unstable fixed point is a point in a system that repels nearby trajectories. If the system is at that point and is perturbed, it will not return to that point.	Like a marble balanced on top of a dome—any tiny push will cause it to roll away. Unstable because even the smallest push will move it away.
Saddle fixed point	A point where the system may be attracted in some directions but repelled in others.	Like a marble on a horse saddle—it stays put if moved front-to-back but rolls off side-to-side. Saddle because stable in one direction but unstable in another.
Stable limit cycle	A stable limit cycle is a repeating pattern or cycle in a system that attracts nearby trajectories. If the system is slightly perturbed, it returns to this cycle.	Like a ceiling fan spinning at steady speed—even if slowed slightly, it returns to original rhythm. Stable because it settles into repeating motion.
Unstable limit cycle	An unstable limit cycle is a repeating pattern or cycle in a system that repels nearby trajectories.	If you spin a coin perfectly, slight wobble eventually causes it to fall. Unstable because the spinning motion is hard to maintain.

Bifurcations

In a dynamical system, **parameters** are fixed values that influence the systems behavior but do not evolve with time. For example, in the 1D equation, $\frac{dx}{dt} = f(x) = ax$, a (a real number) is the sole parameter. Clearly, choosing different parameters will produce distinct dynamics, changing the state trajectories from a given initial condition. Thus, varying parameters can shift the location of attractors in the **state space**. Furthermore, the qualitative dynamics may be altered. Loosely, a qualitative change in dynamics as parameters are varied is known as a **bifurcation** and the parameters that induced the bifurcation are known as **bifurcation parameters**. In the context of this work, bifurcations can be understood as (1) the creation/annhiliation of attractors/repellers, or (2) the change in stability of an attractor/repeller. We now give heuristic descriptions for the distinct bifurcations utilized in this work.

Saddle-Node (SN): We first presume that a stable fixed point and a saddle coexist a certain value of the bifurcation parameter. As the bifurcation parameter varies, these fixed point move towards one another. At a critical value, they collide and are both annhilated. Mathematically, this is realized as two distinct fixed point solutions becoming a double root (collision) and then complex conjugates. Varying the bifurcation parameter in reverse, the fixed points reappear at the point of collision and move away from one another.

Saddle-Node on an Invariant Circle (SNIC): We begin by assuming a stable fixed point and a saddle coexist on a closed trajectory. As we vary the bifurcation parameter, these fixed points move along the circle and gradually approach one another. At a critical value, they collide and annihilate each other, just like in a standard saddle-node bifurcation. However, this collision occurs on a periodic orbit, and their annhilation produces limit cycle. In reverse, the fixed points appear on the limit cycle, destroying the periodic solution.

Supercritical Hopf (supH): We start with a stable spiral that becomes increasingly oscillatory as the bifurcation parameter is varied. At the critical value, this fixed point loses stability and a stable limit cycle is born with zero amplitude — it grows continuously from the fixed point. Nearby trajectories that once spiraled into the fixed point now spiral outward and settle on the new periodic orbit. In reverse, the limit cycle shrinks and merges into the stable fixed point, restoring its stability.

Subcritical Hopf (subH): Here, a stable fixed point coexists with an unstable limit cycle that encircles it. As the bifurcation parameter changes, the fixed point becomes unstable, as the unstable limit cycle shrinks and merges with the fixed point, making the fixed point unstable. Reversing the bifurcation, the unstable limit cycle reappears and the fixed point becomes stable.

Saddle Homoclinic (SH): We consider a saddle fixed point with a trajectory that leaves and returns to the saddle along its own unstable and stable directions. As the bifurcation parameter varies, a nearby limit cycle expands and approaches this trajectory. At the bifurcation, the limit cycle collides with the saddle, causing its oscillations to become arbitrarily slow near the saddle. After the collision, the cycle disappears. In reverse, the limit cycle is born suddenly from the saddle’s orbit.

Fold Limit Cycle (FLC): Here, two limit cycles — one stable, one unstable — coexist. As the bifurcation parameter is varied, they approach one another in amplitude and shape. At the bifurcation point, they collide and annihilate, just like fixed points in a saddle-node bifurcation. Reversing the parameter change, the two cycles are born simultaneously.

Framework

(Much of the following is adapted or pulled directly from Saggio and Szuromi Papers.)

The model uses fast-slow bursting to simulate seizures. A simple fast-slow burster is characterized by two rhythms: the fast rhythms of oscillations in the active or bursting state and the slow rhythm of transitions between the active and resting state.

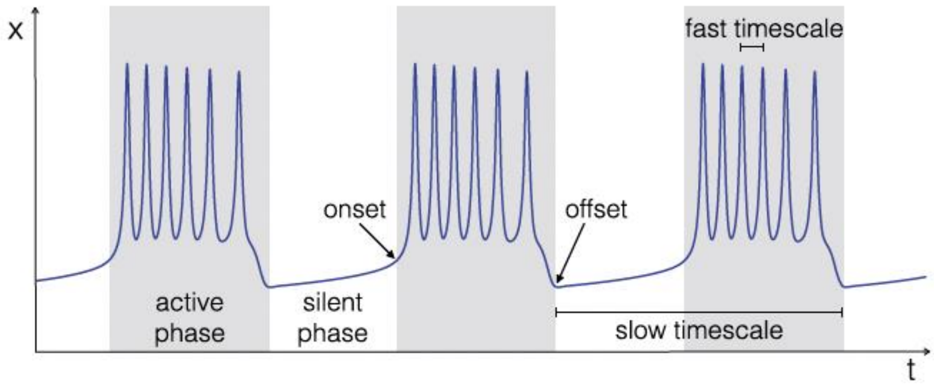

The oscillations in the bursting state are described by the time evolution of a fast subsystem (fast variables). In contrast, the transition between resting and bursting states is dictated by the time evolution of a slow subsystem (slow variables), which produces a bifurcation in the fast subsystem. Bifurcations are qualitative changes in the behavior of a dynamical system as specific parameters, known as the bifurcation parameters, are varied smoothly. For these transitions (bifurcations) to be determined by the slow subsystem, the bifurcation parameters of the fast subsystem must depend on the slow variables.

The general mathematical framework used to produce this bursting is:

$$\begin{cases} \dot{x} = \boldsymbol{f}(\boldsymbol{x}, z) \\ \dot{z} = k\,\boldsymbol{g}(\boldsymbol{x}, z) \end{cases} \qquad 0 < k \ll 1$$

Where $\boldsymbol{x} = \boldsymbol{x}(t)$ is the n-dimensional state vector of the fast variables, and $z = z(t)$ is the m-dimensional vector of slow variables. The dots represent derivatives with respect to time. $\boldsymbol{f}$ and $\boldsymbol{g}$ are functions. $k = \frac{1}{\tau}$, where τ is the characteristic time constant of the separation of the fast and slow rhythms. We require k to be significantly less than one to ensure a quasi-static variation of parameters.

Resting states are represented as stable fixed-point solutions, and oscillatory states are represented as stable limit cycles in the fast subsystem (Izhikevich, 2010). When no applied stimuli are present, to transition from a resting state to an oscillatory state, a bifurcation must occur that alters the existence or stability of the fixed point, and there must be a stable limit cycle after the bifurcation occurs. To transition back to rest from oscillations, the converse must occur.

Applied stimuli cause state switches by forcing a transition between coexisting attractors. In this case, the system must be bistable, and the forcing must move the system into the alternative attractor's basin.

Bursting Classes & The Unfolding

In planar systems, there are six codim-1 (i.e. requiring the change of only one parameter value) bifurcations that can act as either the onset or offset for the bursting state. Four act as onset bifurcations: Saddle-Node (SN), Saddle-Node on an Invariant Circle (SNIC), Supercritical Hopf (SupH), and Subcritical Hopf (SubH). Four act as offset bifurcations: SNIC, SupH, Saddle-Homoclinic (SH), and Fold Limit Cycle (FLC). This yields sixteen unique pairs of planar onset and offset bifurcations, shown in Table 1, which we call the bursting class (Izhikevich 2000, Saggio et al., 2017).

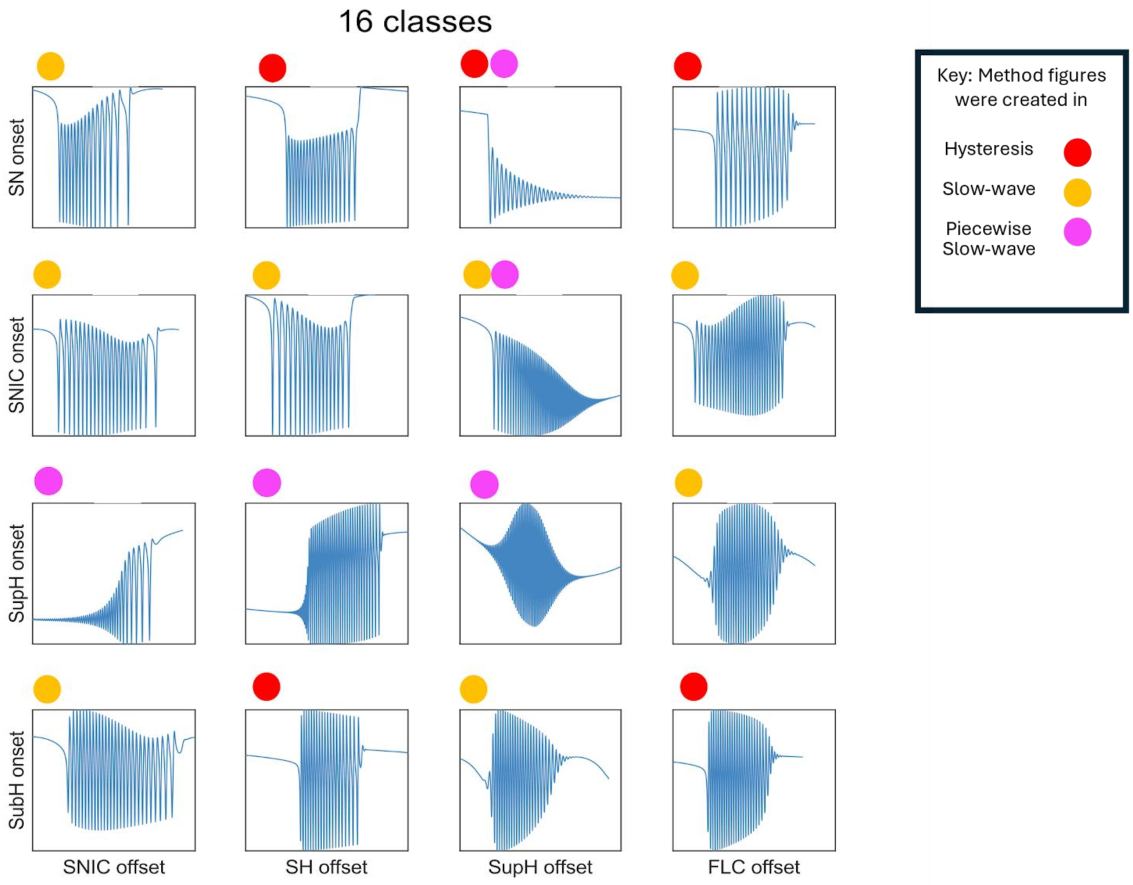

Table 1

A fast subsystem which allows for construction of all 16 bursting classes is created through "unfolding" a singularity of high codimension. The resultant equations are:

$$\dot{x} = -y$$
$$\dot{y} = x^3 - \mu_2 x - \mu_1 - y(\nu + x + x^2)$$

Here, $\vec{\mu} = \begin{bmatrix} \mu_2 \\ -\mu_1 \\ \nu \end{bmatrix}$ is our parameter vector, which defines the parameter space. We often examine spherical "slices" of this parameter space, as the topological structure for large 3D regions can be inferred from the segregated regions on the sphere. Bifurcation manifolds in parameter space intersect the sphere, leaving curves which segregate the surface.

In our diagrams below, we flatten this sphere for better visualization. Below is the 2d flattened diagram and the corresponding 3d projection.

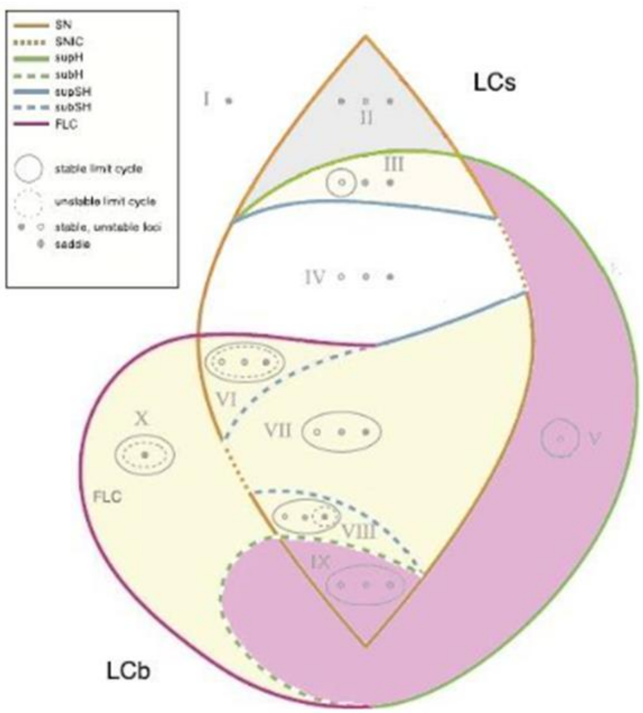

In this framework, a seizure corresponds to a stable limit cycle for sustained oscillations, while the resting state is modeled as a stable fixed point. There is also another fixed point solution, different from the resting state, which we named ‘active rest’ and consider as part of the ictal regime. Which, among these behaviors, are possible in the model depends on the values of its parameters. The input parameters m*u2, mu1,* and *nu* of the differential equations and algorithm that govern the Saggio-Jirsa fast subsystem rely on a three dimensional spherical map. At each point of the map, that is, for any precise choice of the three parameters values, the system exhibits specific behaviors. This map includes a rest and active rest region (grey), a rest/seizure or bistable region (yellow), and a seizure region (purple) that govern the behavior of the seizure. It also includes several bifurcation curves that enclose these regions and correspond to transitions among those behaviors. To manipulate the behavior of the model to create seizures, one would vary the input coordinates m*u2, mu1,* and *nu* to create a specific path within the map. Now understanding the behavior of these dynamical systems, you can begin to understand the behavior of the map in each region.

Region Number	Topology	Description of behavior
I	Single stable fixed point	Behavior in this region will be attraction to the stable fixed point, will see time series at a steady amplitude,
II	Two stable fixed points, one saddle point	Behavior in this region will be attraction to the two stable fixed points and repulsion from the saddle point. System may transition between two fixed points. Will see time series at a steady amplitude
III	One stable fixed point, one saddle point, one stable limit cycle with unstable point inside	Behavior in this region will be attraction to both the stable limit cycle and stable point, thus making it bistable, will see timeseries oscillating in limit cycle or at a steady amplitude at the fixed point
IV	One stable fixed point, one saddle	Behavior in this region will be attraction to the stable point and

	point, one unstable point	repulsion from the saddle and unstable point, timeseries will show rest at a steady value
V	Stable limit cycle with unstable point inside	Behavior in this region will be attraction to the limit cycle, timeseries will show oscillations
VI	Stable limit cycle with unstable limit cycle inside with stable, saddle, and unstable fixed points inside	Behavior in this region will be attraction to both the stable limit cycle and stable point, thus making it bistable, will see timeseries oscillating in limit cycle or at a steady amplitude at the fixed point
VII	Stable limit cycle with stable, saddle, and unstable fixed points inside	Behavior in this region will be attraction to both the stable limit cycle and stable point, thus making it bistable, will see timeseries oscillating in limit cycle or at a steady amplitude at the fixed point
VIII	Stable limit cycle with unstable point, saddle point and unstable limit cycle with stable fixed point inside	Behavior in this region will be attraction to both the stable limit cycle and stable point, thus making it bistable, will see timeseries oscillating in limit cycle or at a steady amplitude at the fixed point
IX	Stable limit cycle with two unstable points and one saddle point inside	Behavior in this region will be attraction to the limit cycle, timeseries will show oscillations
X	Stable limit cycle with unstable limit cycle and stable point inside	Behavior in this region will be attraction to both the stable limit cycle and stable point, thus making it bistable, will see timeseries oscillating in limit cycle or at a steady amplitude at the fixed point

These segregated regions define different dynamical regimes (shown in the next section), of which there are four predominant types: *monostable rest*, *monostable active*, and *bistable (rest/seizure) and bistable (rest/active rest)*. The bistable regions can be categorized further:

- *Limit Cycle big (LCb)* - A stable fixed point and a stable limit cycle exist. The fixed point lies on the interior of the limit cycle.
- *Limit Cycle small (LCs)* - A stable fixed point and a stable limit cycle exist. The fixed point lies on the exterior of the limit cycle.
- *Active Rest* - Two stable fixed points exist.

In the diagrams below, the topological structure of phase space (the fixed points and limit cycles) are sketched in gray in the corresponding regions of parameter space.

***Note:* when in an LCs or Active Rest bistable region, a jump between attractors results in a DC shift in the timeseries.**

```
figure;
get_plot()
axis off;
xlabel('\mu_2')
ylabel('-\mu_1')
zlabel('\nu')
```

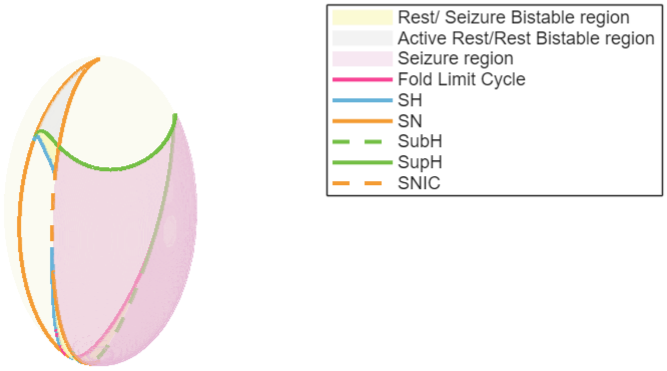

The Saggio-Jirsa model is based upon four different onset (Saddle Node (SN), Saddle Node Invariant Circle (SNIC), Supercritical Hopf (SupH), Subcritical Hopf (SubH)) and offset (Saddle Homoclinic (SH), SNIC, SupH, and Fold Limit Cycle (FLC)) bifurcations.

Each of these has distinct, well described dynamical properties.

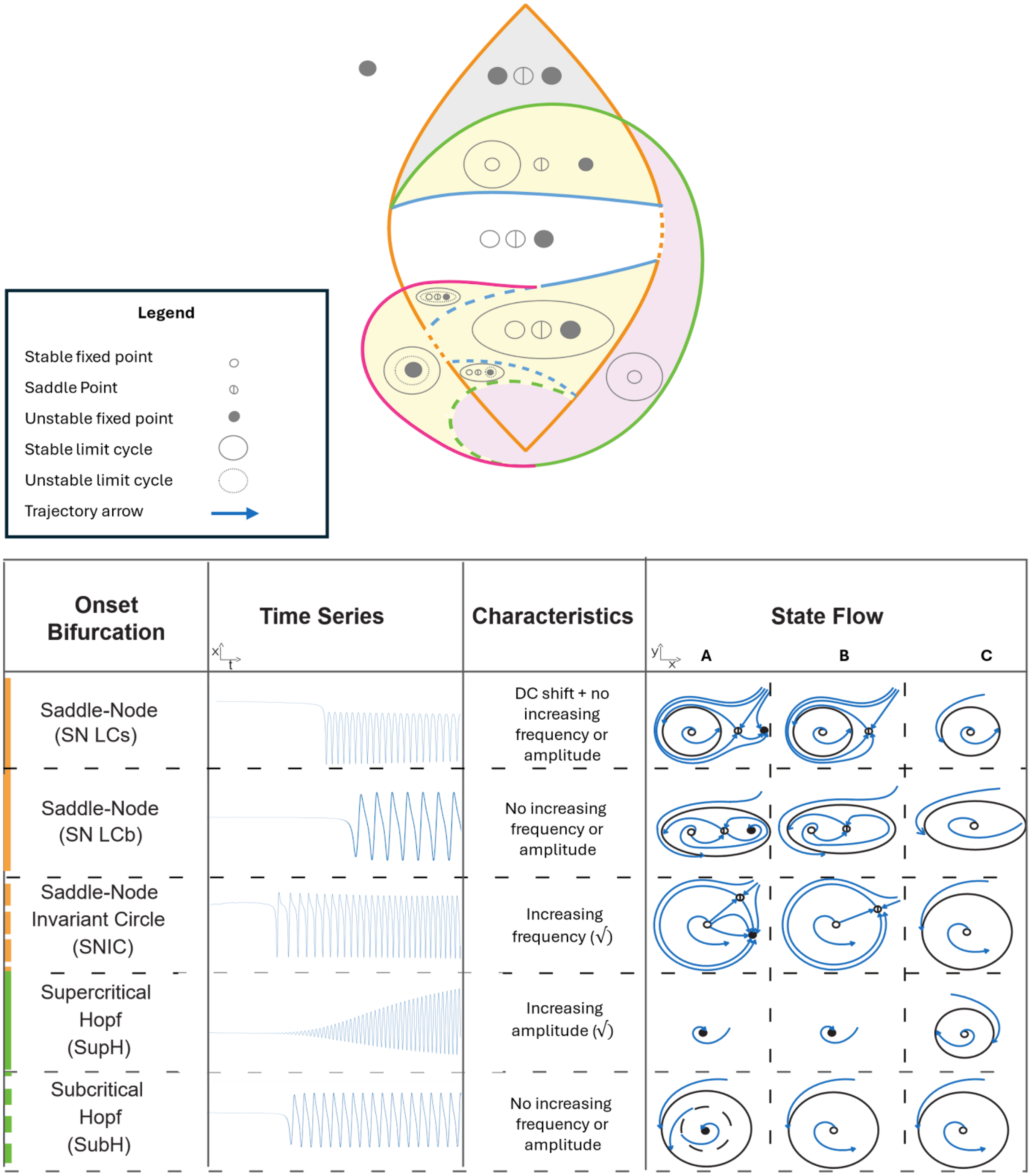

The top portion of this figure shows a bifurcation diagram of the Saggio-Jirsa model. The lower portion shows visualization of onset dynamics in the Saggio-Jirsa bursting model. Saddle Node onsets and Saddle Homoclinic offsets can both arise with or without direct current (DC) shifts, which were distinguished by the presence of big (LCb) or small (LCs) limit cycles in state space (Saggio et al., 2017). Bifurcations scale from zero based on the either square root or logarithmic scaling laws as indicated. The lower right portion of the figure illustrates the state flow diagrams for key bifurcations, showing how system trajectories evolve through state space during seizure onset.

Below, we detail the flow behavior and corresponding signal effect for each bifurcation.

1. Saddle-Node (SN) → DC Shift.

Flow Behavior: Pre-bifurcation: The system resides at a stable equilibrium point (node). A nearby unstable equilibrium (saddle point) exists but has no effect on the system's current state. At bifurcation: As a control parameter crosses a critical threshold, the stable node and saddle point collide and annihilate. Post-bifurcation: With no remaining equilibria, trajectories are forced to escape to the limit cycle.

Signal Effect: A sudden DC shift (voltage jump) occurs as the system transitions from rest to limit cycle.

2. Saddle-Node (SN) → no DC Shift.

Flow behavior: Pre-bifurcation: The system rests at a stable equilibrium (node), with a nearby unstable equilibrium (saddle). At bifurcation: The stable node and saddle collide and annihilate (fold bifurcation), but unlike the standard SN case, the points are enclosed in the limit cycle the system jumps to. Post-bifurcation: The system escapes to the limit cycle.

Signal Effect: No abrupt voltage shift (unlike standard SN) because system jumps to nearby limit cycle. Immediate onset of oscillations (frequency may depend on system parameters).

3. Saddle-Node on Invariant Cycle (SNIC) → Increasing Frequency.

Flow Behavior: Pre-bifurcation: Two equilibria (a stable node and a saddle) exist on a closed orbit (invariant cycle). The system can rest at the stable node. At bifurcation: The equilibria collide and vanish, leaving the invariant cycle intact but now devoid of fixed points. Post-bifurcation: Trajectories must flow continuously around the cycle, forming a limit cycle. Near the bifurcation, the period of oscillation is infinite. As the parameter moves further, the period decreases.

Signal Effect: Oscillations emerge with initially low frequency (long intervals between spikes). Frequency ramps up as the limit cycle tightens (period shortens).

4. Supercritical Hopf (SupH) → Increasing Amplitude.

Flow Behavior: Pre-bifurcation: The system is at a stable equilibrium (spiral sink). Small perturbations decay back to rest. At bifurcation: The equilibrium loses stability. A stable limit cycle emerges smoothly from the fixed point (radius grows from zero). Post-bifurcation: The limit cycle’s amplitude grows as the parameter moves away from the bifurcation point.

Signal Effect: Smooth onset of small oscillations that grow in amplitude.

5. Subcritical Hopf (SubH) → Arbitrary Jumps.

Flow Behavior: Pre-bifurcation: The equilibrium is stable, but an unstable limit cycle surrounds it. At bifurcation: The unstable cycle shrinks and merges with the equilibrium, destabilizing it. Trajectories now escape to a large-amplitude limit cycle. Post-bifurcation: The system exhibits large, sudden oscillations with hysteresis.

Signal Effect: Abrupt onset of high-amplitude oscillations (no smooth transition).

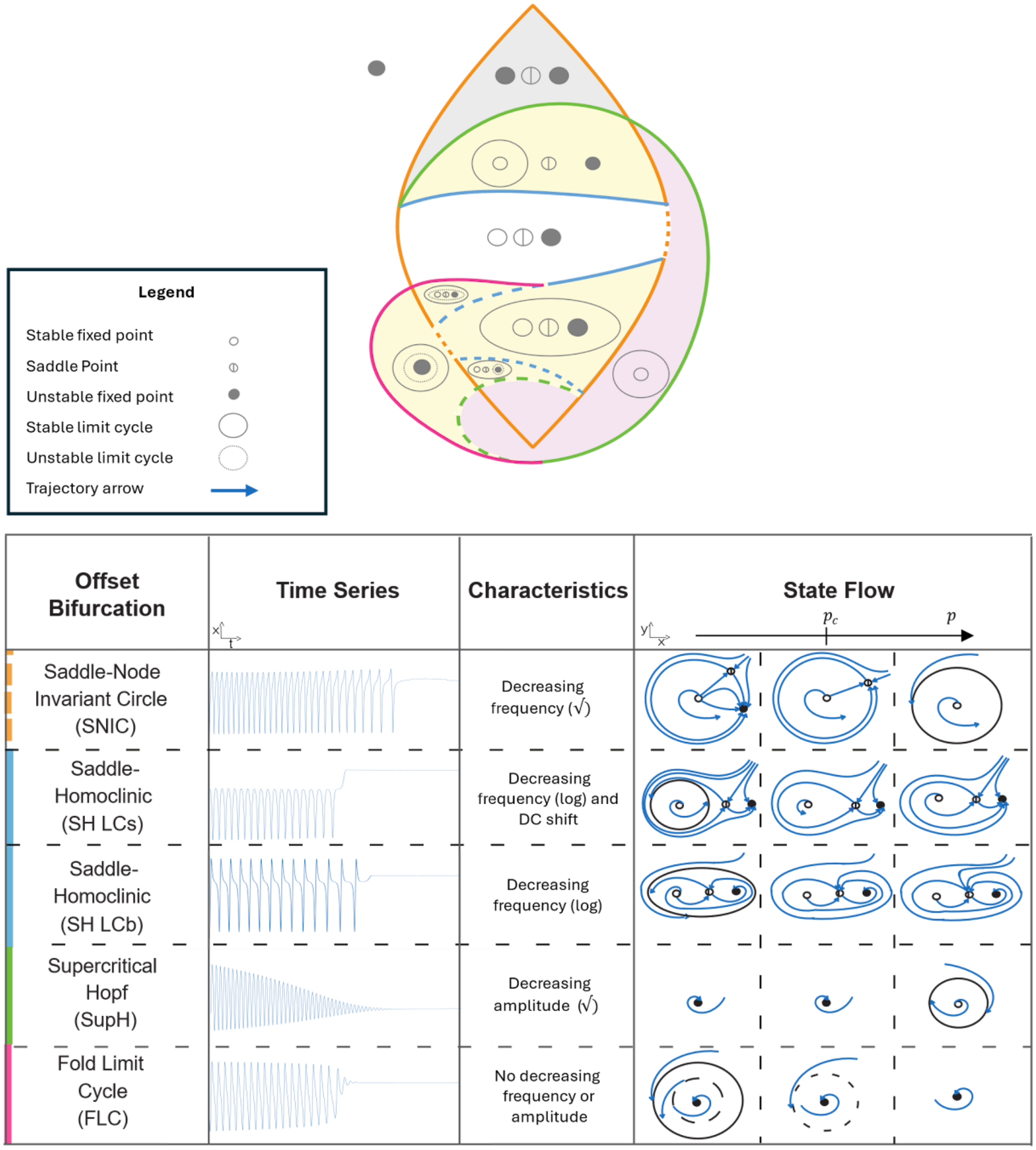

The top portion of this figure shows a bifurcation diagram of the Saggio-Jirsa model. The lower portion shows visualization of onset dynamics in the Saggio-Jirsa bursting model. Saddle Node onsets and Saddle Homoclinic offsets can both arise with or without direct current (DC) shifts, which were distinguished by the presence of big (LCb) or small (LCs) limit cycles in state space (Saggio et al., 2017). Bifurcations scale to zero based on the either square root or logarithmic scaling laws as indicated. The lower right portion of the figure illustrates the state flow diagrams for key bifurcations, showing how system trajectories evolve through state space during seizure offset. Each diagram highlights the pre- and post-bifurcation behavior, including fixed points, limit cycles, and trajectory directionality.

Below, we detail the flow behavior and corresponding signal effect for each bifurcation:

1.Saddle-Node on Invariant Circle (SNIC) → Decreasing Frequency.

Flow Behavior: Pre-termination: Oscillations follow a limit cycle with a "ghost" of the vanished saddle-node pair, causing trajectories to slow near the collision point. At bifurcation: Infinite-period orbit (trajectories take infinitely long to complete a cycle). Post-termination: System collapses to fixed point.

Signal Effect: Exponentially increasing inter-spike intervals (frequency ↘) as trajectories slow near the ghost saddle-node, then abrupt silence when the limit cycle collides.

2. Saddle-Homoclinic (SH) → Decreasing Frequency and DC shift.

Flow Behavior: Pre-termination: Limit cycle approaches a homoclinic orbit, causing trajectories to spend more time near the saddle. At bifurcation: Infinite-period loop forms, then breaks. Post-termination: Oscillations vanish; system returns to equilibrium.

Signal Effect: Spike-wave bursts with logarithmically slowing frequency as phase flow stretches near the saddle's unstable manifold, then voltage snaps to baseline when the homoclinic loop breaks.

3. Saddle-Homoclinic (SH LCb) → Decreasing Frequency.

Flow Behavior: Pre-termination: Limit cycle approaches a homoclinic orbit, causing trajectories to spend more time near the saddle. At bifurcation: Infinite-period loop forms, then breaks. Post-termination: Oscillations vanish; system returns to equilibrium.

Signal Effect: Decreasing frequency (logarithmic slowing) and No DC shift (returns to original baseline).

4. Supercritical Hopf (SupH) → Decreasing Amplitude.

Flow Behavior: Pre-termination: Stable limit cycle shrinks smoothly toward the now-stable equilibrium. At bifurcation: Cycle radius reaches zero; equilibrium regains stability. Post-termination: System rests at the fixed point.

Signal Effect: Smooth amplitude decay as the limit cycle shrinks radially to the equilibrium, no frequency change, then silence at bifurcation.

5. Fold Limit Cycle (FLC) → Sudden shift to zero.

Flow Behavior: Pre-termination: Stable and unstable limit cycles coexist (stable cycle is observable). At bifurcation: Cycles collide and annihilate. Post-termination: Oscillations abruptly cease; trajectories fall to equilibrium.

Signal Effect: Oscillations vanish mid-cycle (no slowing) when stable/unstable cycles collide, causing instant voltage return to baseline.

Bursting Paths & Burster Types

To create a burster, we must identify a path through parameter space that "connects" onset and offset bifurcation curves. We call this path the bursting path; it is the set of parameter values along which the system varies in order to exhibit the proper sequence of bifurcations that yield a burster. To create a burster of a particular class, we must find a path that appropriately connects the correct onset and offset bifurcations. Movement of the system along this path is accomplished by parameterizing the bursting path in terms of the slow variables, z.

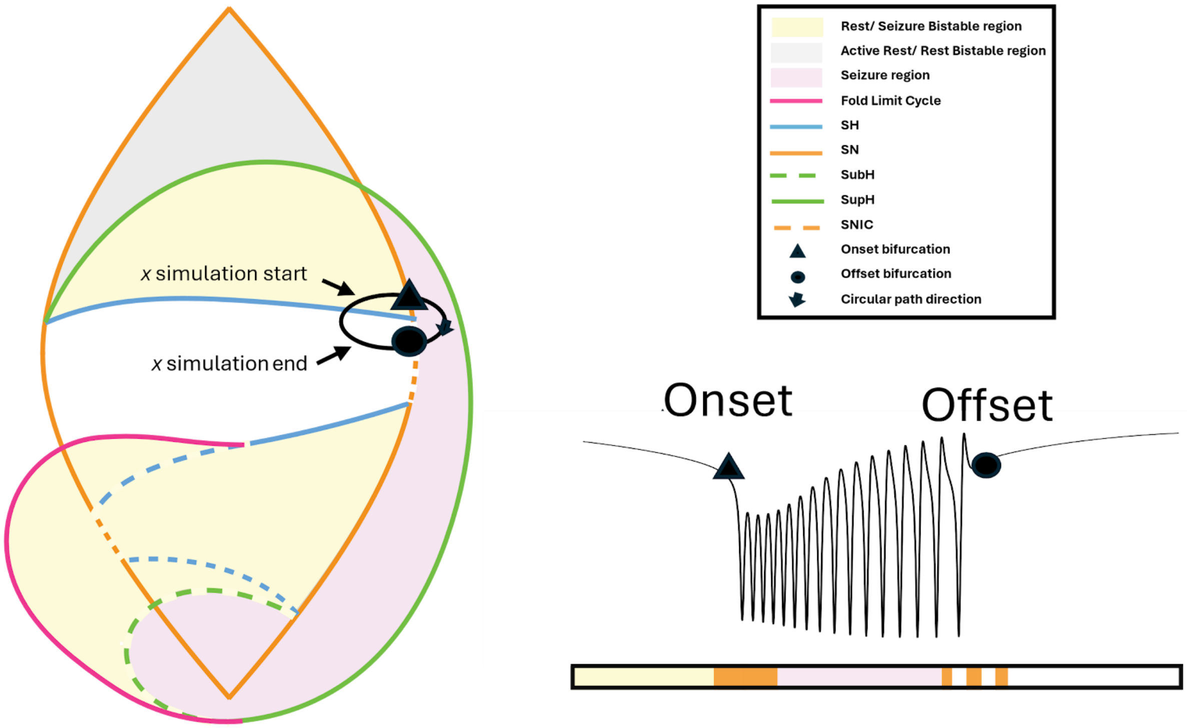

The traversal of this path is driven by the slow oscillation of the system. The slow oscillation can occur through two mechanisms:

- *Slow-wave burster* - The slow subsystem is a self-sustained oscillator, thus feedback from the fast to the slow subsystem is not required. In this case, the slow subsystem must be at least two-dimensional, $m = 2$.
- *Hysteresis-loop burster* - The slow subsystem oscillates due to feedback from the fast subsystem. This can occur if the fast subsystem shows hysteresis between the silent and active states, which can be used to inform the slow subsystem about the state of the fast subsystem (e.g., by baseline). In this case, one slow variable is enough, $m = 1$.

Adding noise into the equation of the simulation

Seizures generated using Saggio et al.'s dynamical model of fast-slow bursting are mostly realistic, but they lack certain features of human sEEG recordings, such as noise. We added dynamical pink noise to our simulated seizures. Pink noise was chosen because it closely resembles noise in the brain (i.e. 1/f noise).

Dynamical noise, or parametric noise, was added to the fast variable (*x*) of the model equations (see Saggio 2017). This represents noise in the brain (i.e. random voltage fluctuations) that creates small perturbations, some of which may push the system into or out of the seizure state (da Silva 2003, Maturana 2020). In the hysteresis model, additional noise stops the hysteresis effect, and the system undergoes noise induced transitions as opposed to bifurcation induced transitions.

In the slow wave model, individual bifurcations responded differently to additional dynamical noise. The SubH and SupH bifurcations produce seizures that were largely unaffected by noise because most of their path was in the obligatory seizure region. Note that the SubH theoretically might be more susceptible to noise when it is in the the fixed point/limit cycle bistability region, which we did not test. The SN and SNIC bifurcations do not clearly start the seizure or have the visual characteristics we typically associate with the bifurcation at high levels of noise. Note this may be a byproduct of the proximity to the bistability region. This may also explain why SNIC bifurcations are so hard to spot in human data and account for 3% of all human data seizures.

Hysteresis-Loop Bursters

Slow Dynamics

For hysteresis-loop bursters, the simplest construction of a path is an arc (a segment of a great circle) on the sphere. This arc is drawn from a point on the offset curve through a bistable region to a point on the onset curve. We design the slow dynamics so that the traversal of this path works as follows. When the system is at rest, the parameters should change so that the system moves towards the onset bifurcation. When the system is in an active state, the system should move towards offset. Using a one dimensional slow-subsystem, this means we want z to increase if the system is at rest, and decrease if the system is seizing. The following formulation accomplishes this:

$$g(\boldsymbol{x}, z) = d^* - \sqrt{(x - x_s)^2 - (y - y_s)^2}$$

Here, (x_s, y_s) are the coordinates of the resting state. We note that $y_s = 0$ always. We also note that $x_s = x_s\left(\overrightarrow{\mu}\right) = x_s(z)$ since $\overrightarrow{\mu} = \overrightarrow{\mu}(z)$.

This formulation essentially measures the distance between the current location of the system and the resting state. If this distance is below a threshold, d^*, z increases, and the parameters approach critical onset values. When above the threshold, g is negative, and we approach offset.

Parametrization

For the slow dynamics to generate a hysteresis-loop burster, we need an appropriate parametrization of $\overrightarrow{\mu}(z)$ (bursting path). As we said, the simplest way is to make an arc connecting points on onset and offset curves using a segment of a great circle.

First, we choose points in parameter space, $\boldsymbol{A}$ and $\boldsymbol{B}$. Where $\boldsymbol{A}$ lies on the offset curve, and $\boldsymbol{B}$ lies on the onset. Given these choices the parametric equation is then

$$\overrightarrow{\mu}(z) = R(\boldsymbol{E}\cos(z) + \boldsymbol{F}\sin(z))$$

where

$$\boldsymbol{E} = \frac{\boldsymbol{A}}{R} \quad \text{and} \quad \boldsymbol{F} = \frac{(\boldsymbol{A} \times \boldsymbol{B}) \times \boldsymbol{A}}{||(\boldsymbol{A} \times \boldsymbol{B}) \times \boldsymbol{A}||}$$

Given that the system begins at rest with $z = 0$, and that the arc path connecting $\boldsymbol{A}$ to $\boldsymbol{B}$ solely passes through bistable regions, this parametrization (coupled with the slow dynamics above) will produce a hysteresis-loop burster.

Paths

5 of the 16 burster types can be created using hysteresis-loop bursters

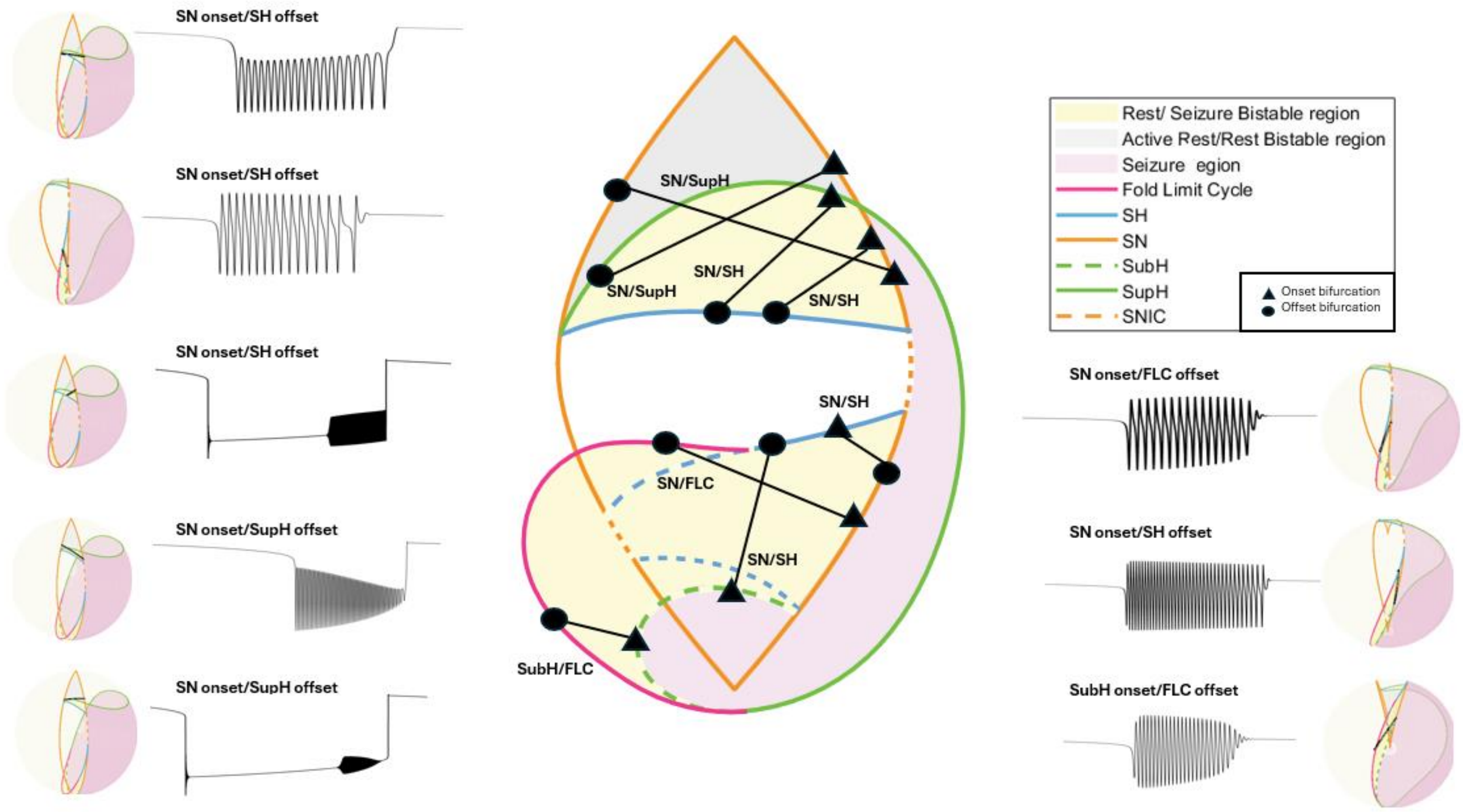

CODE

Settings

```
clear all

% Settings - Integration
x0=[0;0;0]; % initial conditions (must be a column)

% Settings - Model
% focus
b = 1.0;

% radius of the sphere, do not change
R = 0.4;

 %  The dstar parameter is an excitability parameter that controls the ratio between duration
of seizure and duration of rest. When dstar is smaller or equal to zero, no seizure activity is
possible, and the system will always stay at rest. For small';
 %   positive values of dstar, seizure and rest occur. For sufficiently big values of dstar,
only seizure activity is possible. '
dstar = 0.3;

%%'The parameter k determines how fast the slow variable traverses through
%%parameter space. A smaller k yields a slower traversal of k.
```

```
k=0.00015;

%the parameter k_fast determines how many oscillations are in the burst. A
%higher k is yields more bursts, a lower k yields less oscillations
k_fast = 1;

%the parameter alpha controls the amplitude of the burst. a lower alpha
%yields a smaller amplitude, a higher alpha yields a higher amplitude
alpha = 0.2;


% The N parameter controls solution of resting state. Upper Branch (Case 1): Smoother transitions, reduced hysteresis. Lower Branches (Cases 2 and 3): Potential for hysteresis, with complex, path-dependent responses and multiple equilibria. The systems
% state may not revert immediately when external conditions are reversed, creating the characteristic hysteresis loop.
N = 1;

%length of time the simulation will run for
tmax = 15000;

%Integration step/Sampling rate of the simulation, assume it is represented in miliseconds
tstep = 0.01;

%%class wanted to run, input '2s',  '2b', '3s','4b','10', '11' '14', '16'
%this shows what labels correspond to what class : '2s - SN/SH',  '2b-SN/SH', '3s- SN/SUP','4b - SN/FLC ','10-SN/SH', '11-SN/Sup' '14-SN/Sub', '16-SN/Sup'
class = '2s';

%% function takes in class, and gets a randomized point on each
%% bifurcation curve
[onset_curve,offset_curve]=hysteresis_random_path(class);


onset_curve_length=length(onset_curve);
offset_curve_length=length(offset_curve);


%%choose specific points. These points will be the index of the total curve
%%length found in the previous two lines. The number chosen must be less
%%than the total length of the particular curve
onset_index = 11;  %start at first point on the onset curve
offset_index = 3;  %end at 50th point on the offset curve
A = offset_curve(:,offset_index);
B = onset_curve(:,onset_index);



% uncomment this code to do random path
% % One random path - select random point on onset curve and offset curve
% random_onset_index=randsample(onset_curve_length,1);
% random_offset_index=randsample(offset_curve_length,1);
```

```
 % A = offset_curve(:,random_offset_index);
 % B = onset_curve(:,random_onset_index);
 tspan = 0:tstep:tmax;

 % Create arc path
 [E, F] = Parametrization_2PointsArc(A,B,R);

 N_t = length(tspan);
 X = zeros(3,N_t);
 xx = x0;

 %Dynamical pink noise, or parametric noise, is added to the fast variable (x) of'the governing
model equations. This represents noise in the brain
 %i.e. random voltage fluctuations) that creates small perturbations, some of which'may push
the system into or out of the seizure state.
 sigma = 00;
 Rn = [pinknoise([1,N_t],-1, sigma);pinknoise([1,N_t],-1, 00);pinknoise([1,N_t],-1, 00)];

 %integration to generate signal
 for n = 1:N_t

     % Euler-Meruyama method
     Fxx = HysteresisLoop_Model(tspan(n),xx,b,k,k_fast, alpha, R,dstar,E,F,N);
     xx = xx + tstep*Fxx + sqrt(tstep)*Rn(:,n);
     X(:,n) = xx;

 end

 x = X';
 t = tspan;

 % Onset happens when you leave the resting state. In the absence of noise you can use peaks in
the z variable.
 % With noise, you need to explicitly track the distance between the resting state and the
current state of the system.
 % When it exceeds dstar, that should be good. Vice versa for offset. In the model function you
will see another function
 % used called resting state. This function calculates the x coordinate for the fixed points,
x_rs (y coordinate is always 0).
 % Then just take the distance between (x_rs, 0) and (x, y), the current position of the
system.
 % If you look at the equations for zdot you will see this is how z “knows how to turn around
 z = x(:,3);

 % Calculate Bursting Path
 mu2 = R.*(E(1).*cos(z)+F(1).*sin(z));
 mu1 = -R.*(E(2).*cos(z)+F(2).*sin(z));
 nu = R.*(E(3).*cos(z)+F(3).*sin(z));

 figure;
 grid off;
 set(gca, 'XColor', 'none');
```

```
plot(t,x(:,1),'LineWidth',1,'DisplayName','x', 'Color','k');
%xticks([])
hold on
plot(t,x(:,3),'r','LineWidth',2,'DisplayName','z')
hold off
%xlabel('t')
title('Timeseries')
legend('Location','northwest','FontSize',18)
```

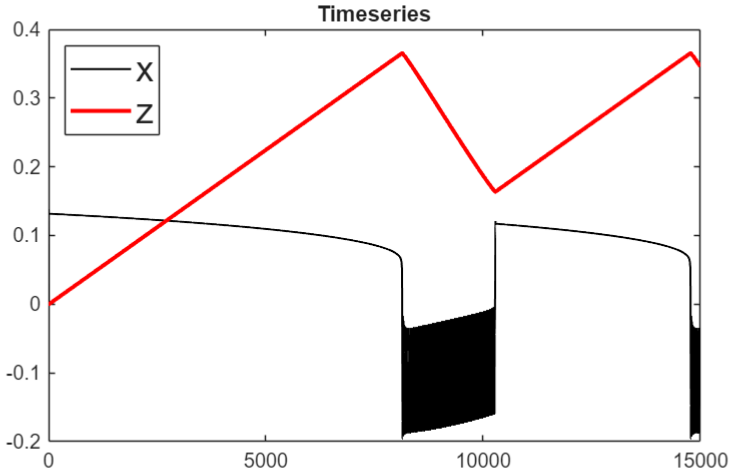


```
figure;
get_plot();
plot3(mu2,-mu1,nu,'k', 'DisplayName', 'Bursting Path', 'LineWidth',3);
xlabel('\mu_2')
ylabel('-\mu_1')
zlabel('\nu')
```

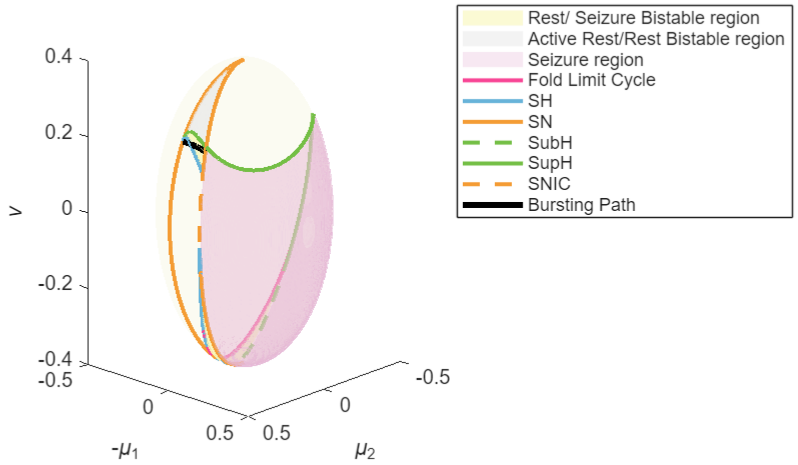

SLOW-WAVE BURSTERS - CIRCULAR

Parametrization

Slow wave bursters must be self-sustaining oscillations. In other words, the direction in which the bursting path is traversed will not change due to feedback from the fast subsystem (i.e. whether the system is active or resting). Thus,

the simplest way to construct a slow wave path is to draw a closed loop (circle) which is traversed in a single direction, driven by a dummy variable at constant velocity:

$$\dot{z} = k$$

We need 3 initial points in parameter space to define the circle on the sphere. Call them $\boldsymbol{P}_1$, $\boldsymbol{P}_2$, $\boldsymbol{P}_3$. Here, the traversal of the path is intended to pass through these points in sequential order. The unique circle which passes through these points can be described as the intersection of the sphere and the plane which passes through these 3 points. First, we define the plane. We start with the normal vector:

$$\hat{\boldsymbol{n}} = \frac{(\boldsymbol{P}_1 - \boldsymbol{P}_2) \times (\boldsymbol{P}_1 - \boldsymbol{P}_3)}{\|(\boldsymbol{P}_1 - \boldsymbol{P}_2) \times (\boldsymbol{P}_1 - \boldsymbol{P}_3)\|}$$

Letting $\hat{\boldsymbol{n}} = \begin{bmatrix} n_1 \\ n_2 \\ n_3 \end{bmatrix}$, the equation of the plane is the standard formulation:

$$n_1 x + n_2 y + n_3 z - \rho = 0$$

where $\rho = \hat{\boldsymbol{n}} \cdot \boldsymbol{P}_1$ is the signed distance from the origin to the plane. Note x, y, z refer to coordinates in parameter space here.

Using the pythagorean identity, we find that the radius of the smaller circle is $r = \sqrt{R^2 - \rho^2}$.

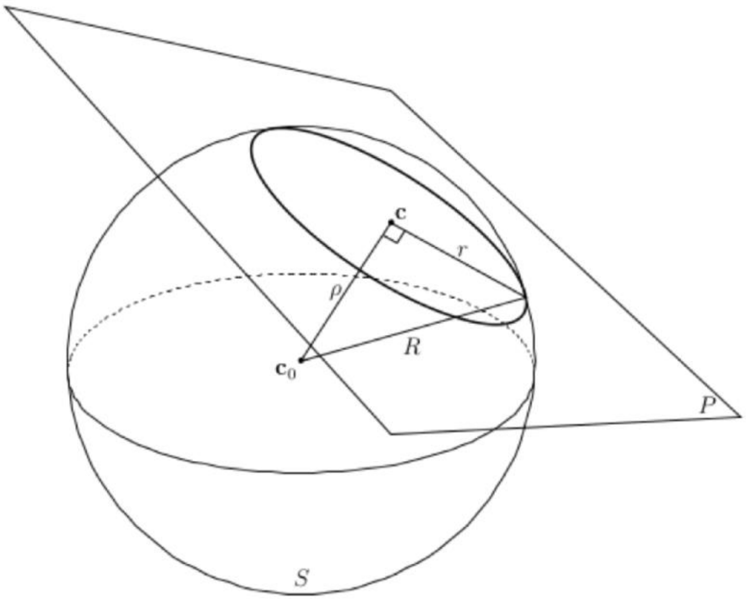

The center of the smaller circle is $\boldsymbol{C} = \rho\hat{\boldsymbol{n}}$. This allows us to construct the following parametrization:

$$\vec{\mu}(z) = \boldsymbol{C} + r(\boldsymbol{E}\cos(z) + \boldsymbol{F}\sin(z))$$

where

$$\boldsymbol{E} = \frac{\boldsymbol{P}_1 - \boldsymbol{C}}{r} \quad \text{and} \quad \boldsymbol{F} = \hat{\boldsymbol{n}} \times \boldsymbol{E}.$$

This step is similar to the hysteresis-loop path because it is constructed by making a great circle on the smaller sphere centered at C with radius r.

Paths

8 of the 16 burster types are created using slow-wave bursters

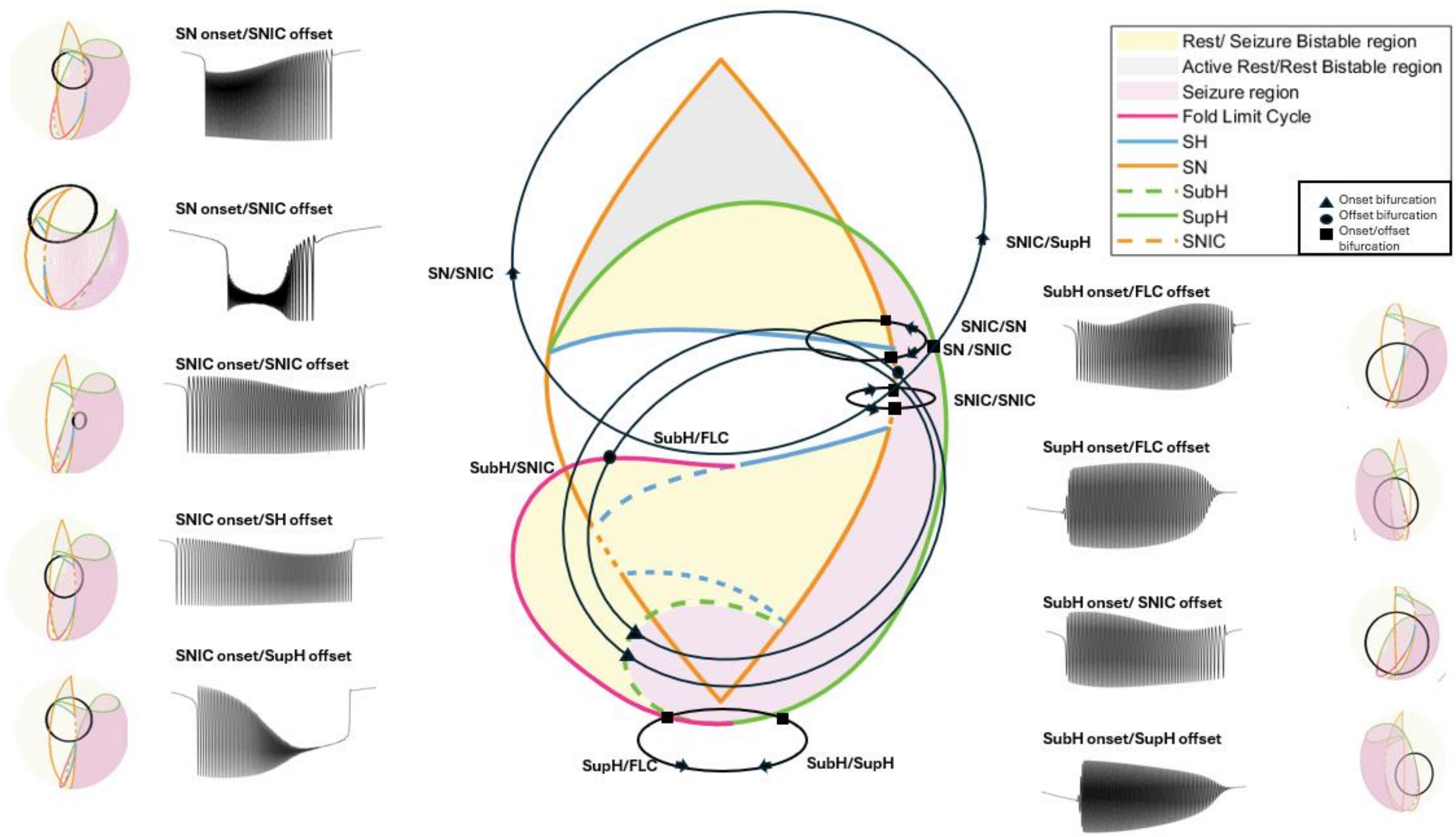

CODE

Settings

```
clear all

% SETTINGS - INTEGRATION
x0=[0;0;0]; % initial conditions (must be a column)

% Settings - Model
% focus
b = 1.0;

% radius of the sphere, do not change
R = 0.4;

%%'The parameter k determines how fast the slow variable traverses through
%%parameter space. A smaller k yields a slower traversal of k.
```

```
k=0.00035;

%the parameter k_fast determines how many oscillations are in the burst. A
%higher k is yields more bursts, a lower k yields less oscillations
k_fast = 1;

%the parameter alpha controls the amplitude of the burst. a lower alpha
%yields a smaller amplitude, a higher alpha yields a higher amplitude
alpha = 1;

%length of time the seizure will run for
tmax = 24000;

%Integration step/Sampling rate of the simulation
tstep = 0.01;

%%class wanted to run, input 1,5, 6,7,8,9,12,13,15
%this shows what labels correspond to what class : '1 - SN/SNIC',  '5
%-SNIC/SNIC', '6 - SNIC/SH','7 - SNIC/SUP','8-Sub/FLC', '9-Sup/SNIC'
%'12-Sup/FLC', '13-Sub/SNIC', '15-Sub/Sup',
class = 13;

%% function takes in class 2,4,14,16, and gets a randomized point on each bifurcation curve
[onset_curve,offset_curve,offset_curve2, flag] = slow_wave_circular_random_path(class);
onset_curve_length=length(onset_curve);
offset_curve_length=length(offset_curve);


%%choose specific points
onset_index = 1;
offset_index = 1;

if flag == 2 || flag == 3
p1 = onset_curve(:,onset_index);
p2 = offset_curve(:,offset_index);
p3 = offset_curve2;
else
p1 = onset_curve(:,onset_index);
p2 = offset_curve2(:,offset_index);
p3 = offset_curve;
end



% uncomment this code to do random path
% % One random path - select random point on onset curve and offset curve
% onset_curve_length=length(onset_curve);
%     offset_curve_length=length(offset_curve);
%     random_onset_index=randsample(onset_curve_length,1);
%     random_offset_index=randsample(offset_curve_length,1);
%     if flag == 2 || flag == 3
%
```

```
%         p1 = onset_curve(:,random_onset_index);
%         p2 = offset_curve(:,random_offset_index);
%         p3 = offset_curve2;
%     else
%         p1 = onset_curve(:,random_onset_index);
%         p2 = offset_curve2(:,random_offset_index);
%         p3 = offset_curve;
%     end
tspan = 0:tstep:tmax;

% Create circular path based 3 defining points
[E, F, C, r] = Parametrization_3PointsCircle(p1',p2',p3');


N_t = length(tspan);
X = zeros(3,N_t);
xx = x0;
sigma = 40;
Rn = [pinknoise([1,N_t],-1, sigma);pinknoise([1,N_t],-1, 00);pinknoise([1,N_t],-1, 00)];
mu2_big = zeros(1, length(N_t));
mu1_big = zeros(1, length(N_t));
nu_big = zeros(1, length(N_t));

%integration to generate signal
for n = 1:N_t
    %Euler-Meruyama method
    [Fxx, mu2, mu1,nu] = SlowWave_Model(tspan(n),xx,b,k,k_fast, alpha, E,F,C,r);
    xx = xx + tstep*Fxx + sqrt(tstep)*Rn(:,n);
    X(:,n) = xx;
     mu2_big(n) = mu2;
    mu1_big(n) = mu1;
    nu_big(n) = nu;


end


% Ensure p1, p2, p3 are column vectors (if not already)
p1 = p1(:);
p2 = p2(:);
p3 = p3(:);
mu1_big = -mu1_big;

tol = 1e-6;

if class == 5 || class == 1 || class == 6 || class == 8 || class == 13
    % Find onset_idx (match p1 in mu2_big, mu1_big, nu_big)
    onset_idx = find( ...
        abs(mu2_big(:) - p2(1)) < tol & ...
        abs(mu1_big(:) - p2(2)) < tol & ...
        abs(nu_big(:) - p2(3)) < tol ...
    );
```

```
    % Find offset_idx (match p2 in mu2_big, mu1_big, nu_big)
    offset_idx = find( ...
        abs(mu2_big(:) - p1(1)) < tol & ...
        abs(mu1_big(:) - p1(2)) < tol & ...
        abs(nu_big(:) - p1(3)) < tol ...
    );


else
    % Find onset_idx (match p1 in mu2_big, mu1_big, nu_big)
    onset_idx = find( ...
        abs(mu2_big(:) - p1(1)) < tol & ...
        abs(mu1_big(:) - p1(2)) < tol & ...
        abs(nu_big(:) - p1(3)) < tol ...
    );

    % Find offset_idx (match p3 in mu2_big, mu1_big, nu_big)
    offset_idx = find( ...
        abs(mu2_big(:) - p2(1)) < tol & ...
        abs(mu1_big(:) - p2(2)) < tol & ...
        abs(nu_big(:) - p2(3)) < tol ...
    );
end
onset_idx = unique(onset_idx);
    offset_idx = unique(offset_idx);
    onset_idx_ = onset_idx(1);
    offset_idx_ = offset_idx(2);


x = X';
t = tspan;



% Calculate Bursting Path
z=0:0.01:2*pi;
mu2=C(1)+r*(E(1)*cos(z)+F(1)*sin(z));
mu1=-(C(2)+r*(E(2)*cos(z)+F(2)*sin(z)));
nu=C(3)+r*(E(3)*cos(z)+F(3)*sin(z));

figure;
plot(t,x(:,1),'LineWidth',1, 'Color','k')
hold on
if(length(x) >= offset_idx)
scatter(t(onset_idx), 1, 'g');
scatter(t(offset_idx),1, 'r')
end
xlabel('t')
ylabel('x')
title('Timeseries')
```

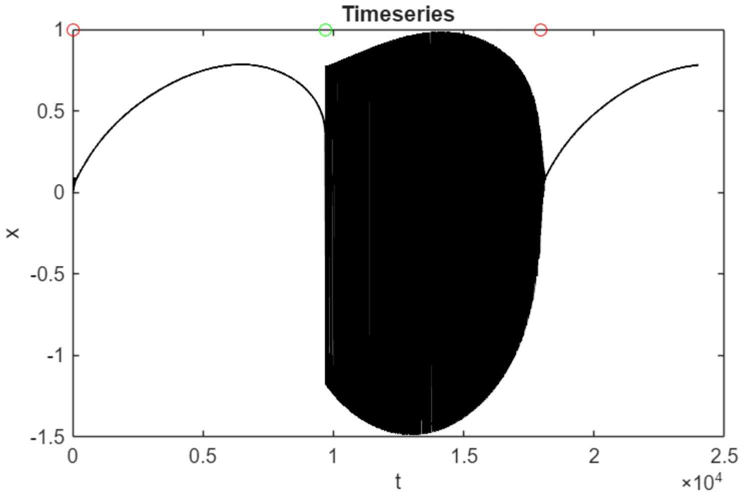


```
figure;
get_plot();
plot3(mu2, -mu1, nu, 'k', 'DisplayName', 'Bursting Path', 'LineWidth',3);

xlabel('\mu_2')
ylabel('-\mu_1')
zlabel('\nu')
```

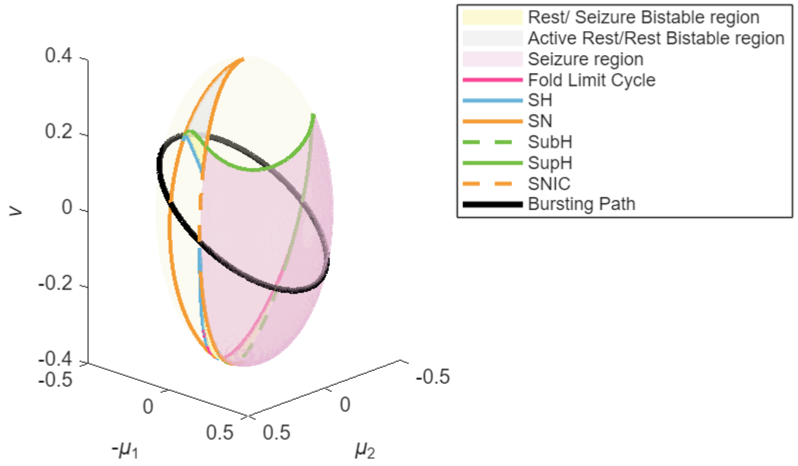

SLOW-WAVE BURSTERS - PIECEWISE

Parametrization

Slow wave bursters must be self-sustaining oscillations. In other words, the direction in which the bursting path is traversed will not change due to feedback from the fast subsystem (i.e., whether the system is active or resting. In certain classes, paths cross the saddle node bifurcation and exhibit dc shifts. If these secondary dynamics are undesirable, one can manipulate the path to not cross the saddle-node bifurcation. While slow wave circles are theoretically plausible to achieve this goal, they are practically difficult because some onset-offset pairs are challenging to connect with continuous pathways without crossing other bifurcations. To address this, we can construct piecewise paths using the slow wave method. In addition, this approach allows a bursting pathway to begin and end in different locations in the state space, rather than returning to the original starting point.

To do this, direct pathways between defined points are used to move through specific locations. Four arcs are created on the surface of the sphere to make a piecewise arc path using 4 points. The first point is a fixed point in the rest region. The second point is a point on the onset bifurcation curve. The third point is a randomized point in the limit cycle

region. The fourth point is a point in the offset bifurcation curve. The arc paths are created from the rest point to first onset bifurcation point, first bifurcation point to limit cycle point, limit cycle point to second bifurcation point, and offset bifurcation point to the rest point, to create a continuous path. Next, to calculate the total time the path traversed, the path was scaled by the k variable and t step variable. Note that unlike the previous two methods, this method only traverses the path one time during the simulation. We produced 5 classes with this method

Paths

5 of the 16 burster types are created using slow-wave piecewise bursters

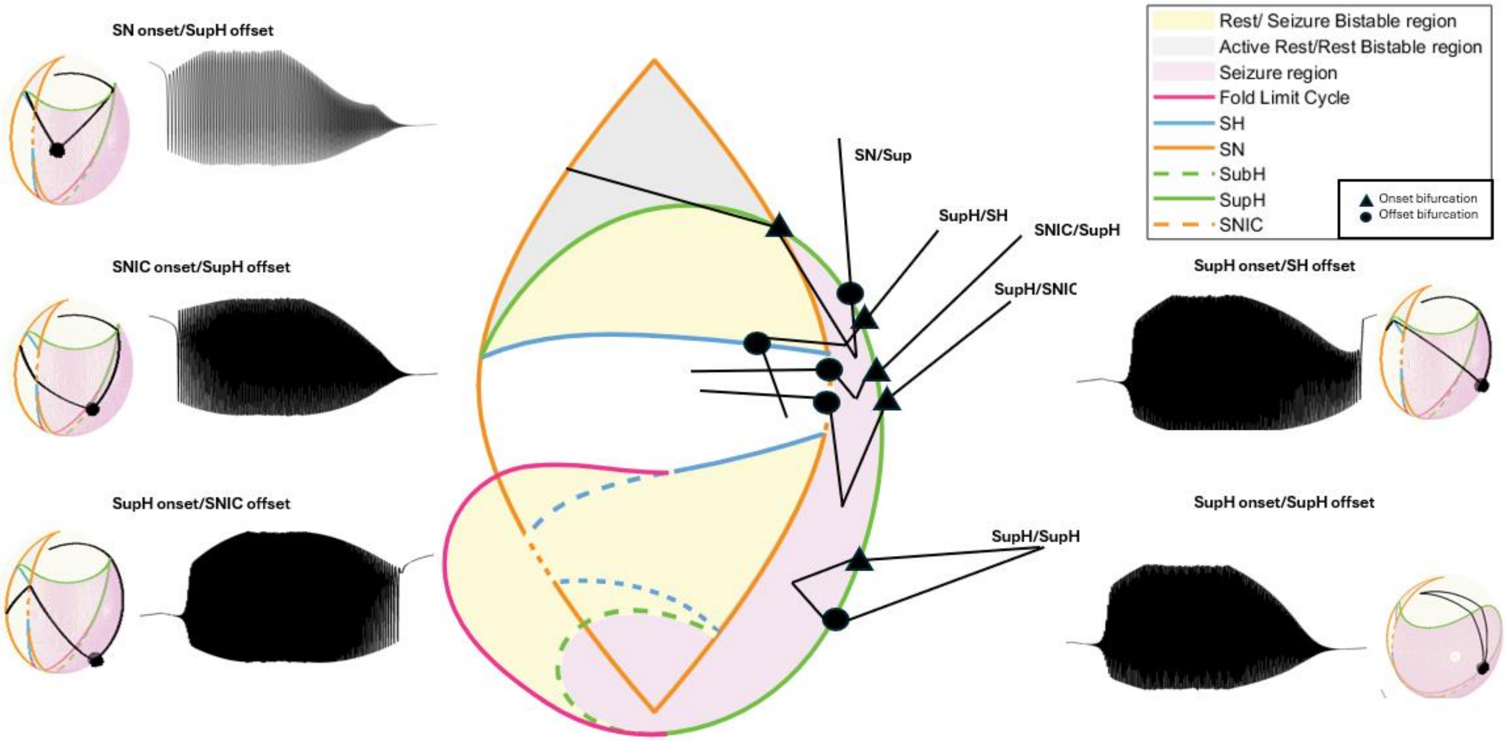

CODE

Settings

```
clear all

% SETTINGS - INTEGRATION
x0=[0;0;0]; % initial conditions (must be a column)

% Settings - Model
% focus
b = 1.0;

% radius of the sphere, do not change
R = 0.4;

%%'The parameter k determines how fast the slow variable traverses through
%%parameter space. A smaller k yields a slower traversal of k.
k=0.00015;
```

```
%the parameter k_fast determines how many oscillations are in the burst. A
%higher k is yields more bursts, a lower k yields less oscillations
k_fast = 0.05;

%the parameter alpha controls the amplitude of the burst. a lower alpha
%yields a smaller amplitude, a higher alpha yields a higher amplitude
alpha = 0.2;

%Integration step/Sampling rate of the simulation
tstep = 0.01;

%%class wanted to run, input 3,7,9,10,11
%this corresponds to '3 - SN/Sup', '7 - SNIC/Sup', '9- Sup/SNIC', '10 -Sup/SH',
% '11 - Sup/Sup'
class = 11;

[p0,onset_curve,p1_5,offset_curve,p3]=piecewise_random_path(class);

onset_curve_length=length(onset_curve);
offset_curve_length=length(offset_curve);


%%choose specific points
onset_index = 1;
offset_index = 44;
p1 = onset_curve(onset_index,:);
p2 = offset_curve(offset_index,:);



% uncomment this code to do random path
% % One random path - select random point on onset curve and offset curve
% random_onset_index=randsample(onset_curve_length,1);
% random_offset_index=randsample(offset_curve_length,1);
% p1 = onset_curve(:,random_onset_index);
% p2 = offset_curve(:,random_offset_index);


stall_val = 30000;
[mu2_straight_path0,mu1_straight_path0,nu_straight_path0,rad1] = sphereArcPath(k,tstep,p0,p1);
[mu2_straight_path0_5,mu1_straight_path0_5,nu_straight_path0_5,rad2] =
sphereArcPath(k,tstep,p1,p1_5);
points = repmat(p1_5, stall_val, 1)';
%path noise sigma
sigma = 100;
Rn = [pinknoise([1,length(points)],-1, sigma);pinknoise([1,length(points)],-1,
sigma);pinknoise([1,length(points)],-1, sigma)];
points = points + Rn;
[mu2_straight_path,mu1_straight_path,nu_straight_path,rad3] = sphereArcPath(k,tstep,p1_5,p2);
[mu2_straight_path1,mu1_straight_path1,nu_straight_path1,rad4] = sphereArcPath(k,tstep,p2,p3);
mu2_all = [mu2_straight_path0, mu2_straight_path0_5, points(1, :), mu2_straight_path,
mu2_straight_path1];
```

```
 mu1_all = [mu1_straight_path0, mu1_straight_path0_5, points(2, :), mu1_straight_path,
mu1_straight_path1];
 mu1_all = -mu1_all;
 nu_all = [nu_straight_path0, nu_straight_path0_5, points(3,:), nu_straight_path,
nu_straight_path1];

 N_t = length(mu2_all);
 X = zeros(3,N_t);
 xx = x0;
 %signal pink noise sigma
 sigma = 100;
 Rn = [pinknoise([1,N_t],-1, sigma);pinknoise([1,N_t],-1, 00);pinknoise([1,N_t],-1, 00)];
 mu2_big = zeros(1, length(N_t));
 mu1_big = zeros(1, length(N_t));
 nu_big = zeros(1, length(N_t));

 %%get onset index by finding Radians to bifurcation, and getting index
 %%through k and tstep parameters
 onset_index = floor((rad1/k)/tstep);
 offset_index = floor(((rad1+rad2+rad3)/k)/tstep) + stall_val;

 %integration to generate signal
 for n = 1:N_t
     %%Euler-Meruyama method
     [Fxx,mu2,mu1,nu] = SlowWave_Model_piecewise(0,xx,b,k,k_fast, alpha, mu2_all(n),
mu1_all(n),nu_all(n));
     xx = xx + tstep*Fxx + sqrt(tstep)*Rn(:,n);
     X(:,n) = xx;
     mu2_big(n) = mu2;
     mu1_big(n) = mu1;
     nu_big(n) = nu;
 end
 x = X';
 seizure = x(:,1);
 figure;
 plot(seizure,'k');
 hold on;
 scatter(onset_index,1, 'g');
 scatter(offset_index,1, 'r');
```

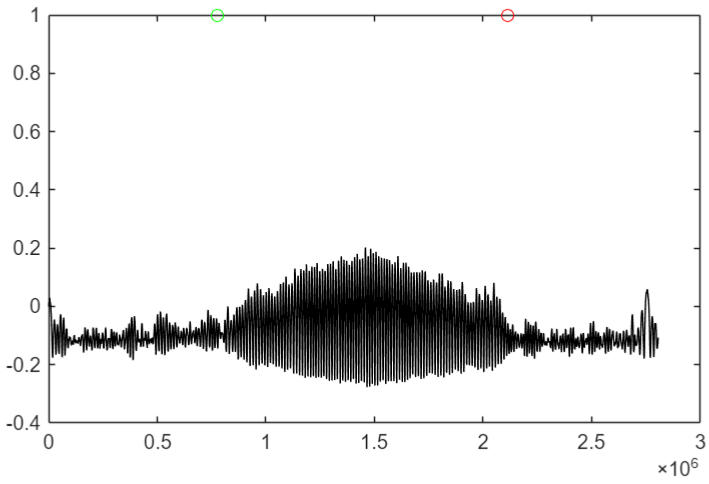


```
figure;
hold on;
get_plot();
hold on;
plot3(mu2_all, -mu1_all, nu_all, 'k', 'LineWidth',2, 'DisplayName', 'Bursting Path');
```

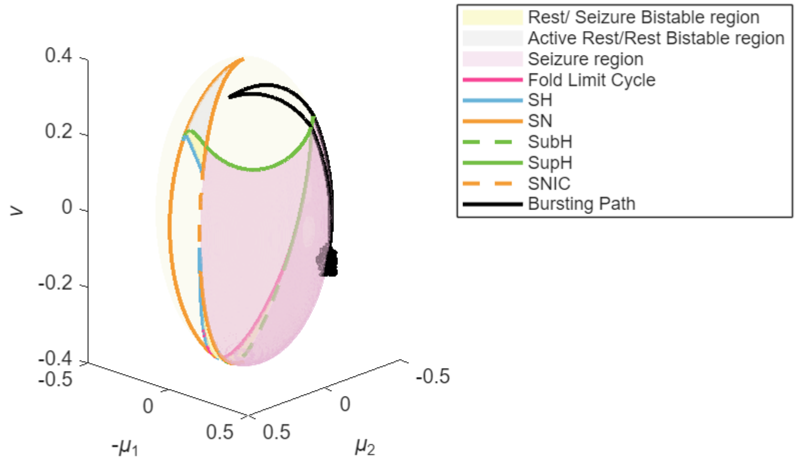

Postprocessing

Seizures generated using Saggio et al.'s dynamical model of fast-slow bursting have similar bursting patterns as human seizures, but they lack certain features of human sEEG recordings, such as noise. We added dynamical pink noise to our simulated seizures. Pink noise was chosen because it closely resembles noise in the brain. Dynamical noise, or parametric noise, was added to the fast variable (*x*) of the model equations (see Saggio 2017) to represent noise in the brain (i.e. random voltage fluctuations) that creates small perturbations, some of which may push the system into or out of the seizure state (da Silva 2003, Maturana 2020). In the hysteresis model, additional noise **stops** the hysteresis loop bursting, and the system undergoes noise induced transitions as opposed to bifurcation induced transitions.

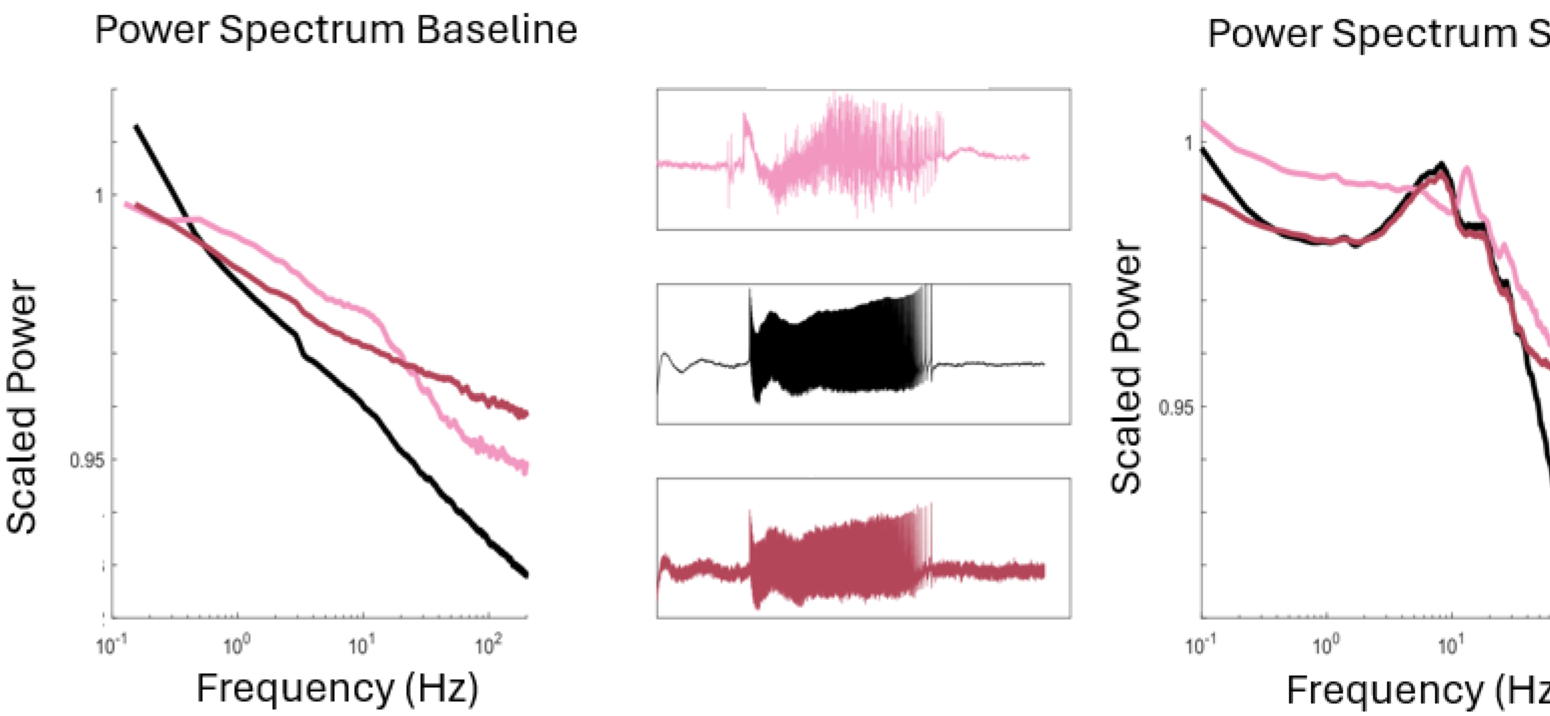


```
%%using previous signal
data = x(:,1);
[pks,locs] = findpeaks(data, 'MinPeakProminence', 0.10);
fs = 1/(0.01*tstep);
t = (0:length(data)-1) / fs;

figure;
plot(data, 'Color',[180/255, 70/255, 90/255]);
hold on;
plot(locs, pks, 'ro'); % Plot peaks with red circles
title(['Number of Peaks: ', num2str(numel(locs))]);
hold off;
```

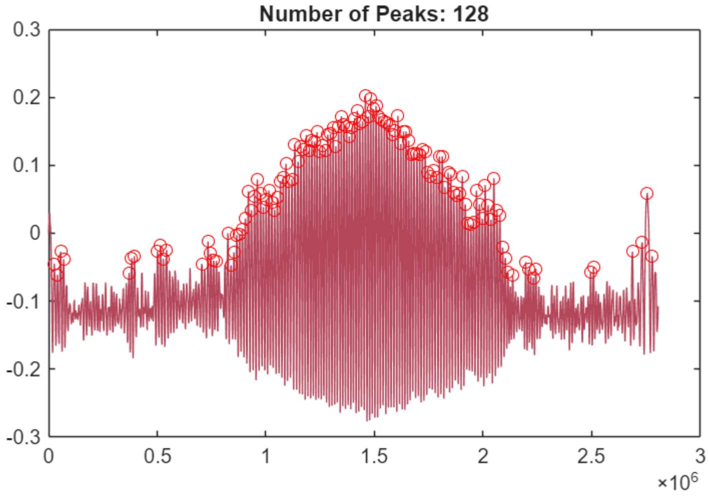


```
%%Getting average spike rate
time_in_seconds = locs / fs; % Convert peak indices to seconds
spike_rates = diff(time_in_seconds);
average_frequency = mean(spike_rates);
fprintf('The average frequency of the signal is %.2f Hz\n', average_frequency);
```

```
The average frequency of the signal is 2.17 Hz
```

We adjust the sampling rate of simulated seizures such that spike frequency was consistent with clinical guidelines. According to AES, a seizure consists of rhythmic bursting activity between 1-30 Hz

```
if average_frequency < 1 || average_frequency > 30
% Calculate spike rates
spike_rates = diff(time_in_seconds);
% Adjust spike rates to achieve a mean average spiking rate of 5 Hz
target_avg_spike_rate = (1/10); % Hz

% Calculate the current average spiking rate
current_avg_spike_rate = mean(spike_rates);

% Calculate the adjustment factor
adjustment_factor = target_avg_spike_rate / current_avg_spike_rate;

% Adjust spike rates
adjusted_spike_rates = spike_rates * adjustment_factor;

% Calculate the mean average spiking rate after adjustment
mean_avg_spike_rate = mean(adjusted_spike_rates);

% Calculate the new sampling frequency
new_sampling_frequency = fs / adjustment_factor;

disp('New sampling frequency (Hz):');
disp(new_sampling_frequency);
disp('New average frequency of the signal (Hz):');
disp(1/target_avg_spike_rate)
end
```

```
%add pink noise over
min_val = min(highpass(data(:), 5, fs));
max_val = max(highpass(data(:), 5, fs));
```

Spontaneous DC shifts reflect alterations in the excitation level of neuronal membrane potentials. The rapid charge accumulation at the electrode-electrolyte interface causes electrode polarization which induces a baseline drift that is distinguished by an electrode's material properties. Modern EEG recording equipment uses alternating current (AC) amplifiers with low-frequency high-pass filters to perform baseline correction once amplifier saturation occurs. To emulate the residual baseline drift observed from platinum-iridium electrodes in human EEG, we applied 6th order digital high-pass filters with cutoff frequencies between 0.1 and 1 Hz

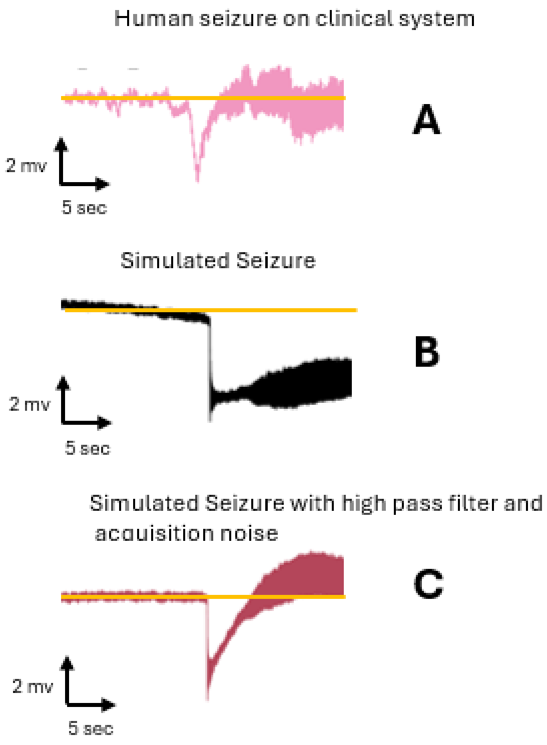


```
HPF = designfilt('highpassiir', ...        % Response type
       'FilterOrder',1, ...     % Filter Order Specification
       'HalfPowerFrequency',0.1, ...
       'DesignMethod','butter', ...      % Design method
       'SampleRate',fs);                 % Sample rate
data = filter(HPF, data);

figure;
plot(t,data, 'Color',[180/255, 70/255, 90/255]);
title('Electrode drift')
```

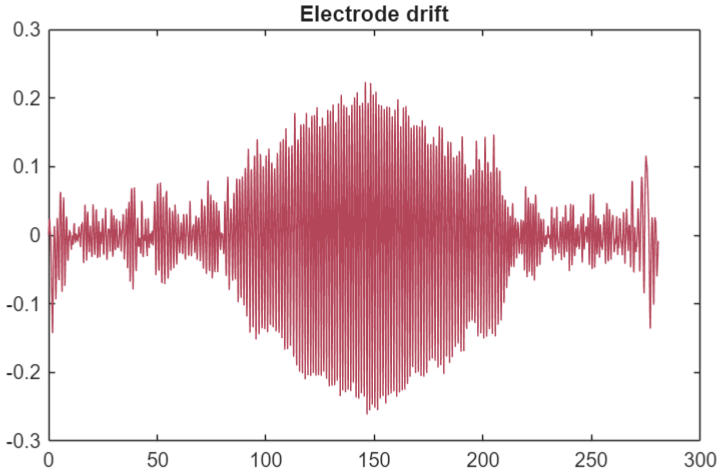

We added 0%, 20%, and 40% signal acquisition pink noise. In the first, we did not add any signal acquisition noise. In the second, we added pink noise that was 20% of the signal amplitude, to represent mediocre recording conditions. In the third, we added pink noise that was 40% of the signal amplitude, to represent poor recording conditions. Signal acquisition noise is picked up by the electrodes used to record brain activity. This neural activity of surrounding brain tissue can be modeled as “pink noise”, or 1/f spatial noise with a normal error distribution (Lennon 2000). The amplitude of signal acquisition noise depends on the signal-to-noise ratio. We generated several datasets to represent variability in clinical recording conditions. We found adding pink noise caused the power spectrums to match.

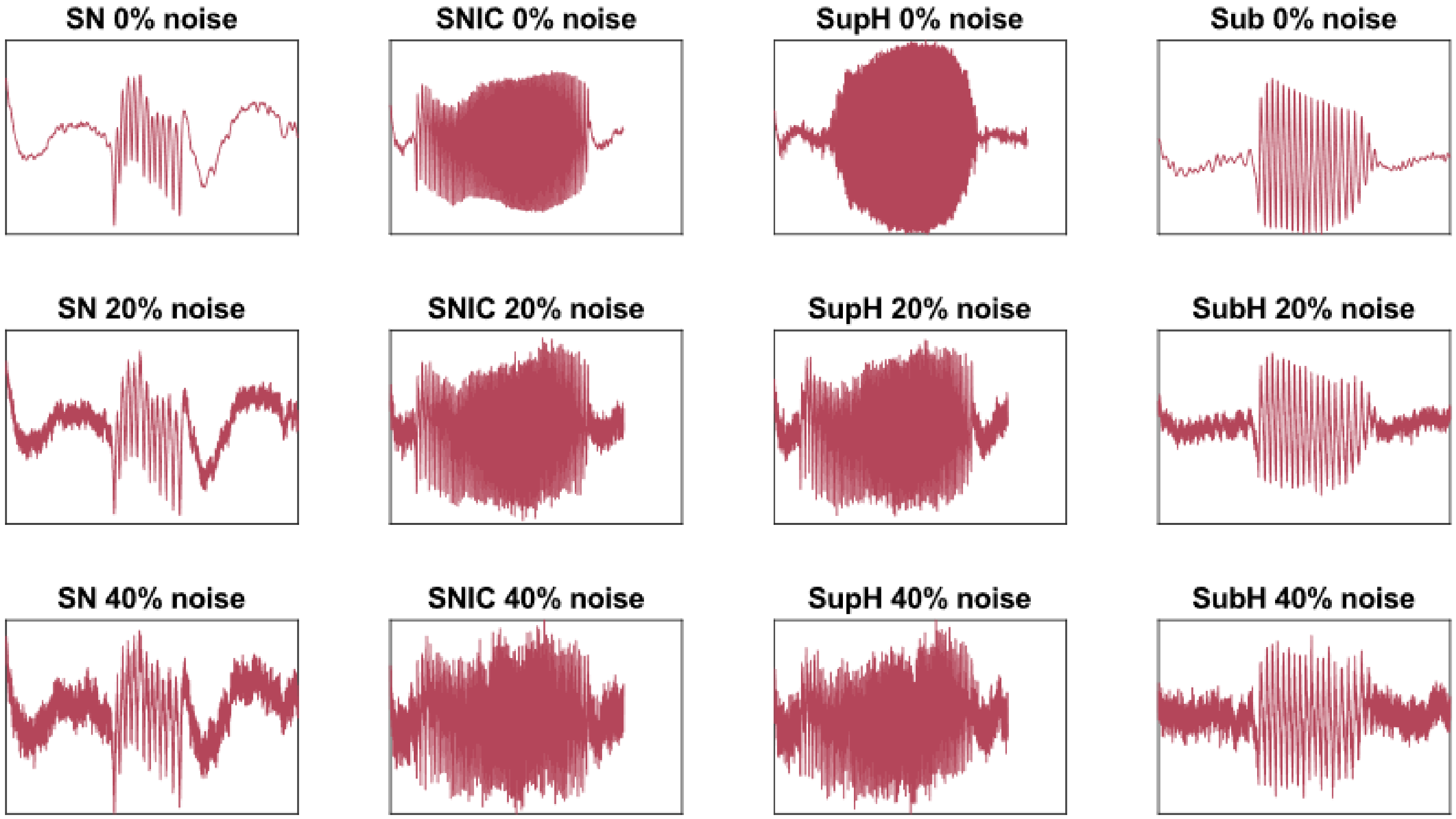


```
data = (data - min_val) / (max_val - min_val);
rms_signal = get_amp(data, fs);
```

```
normalized_data = data;
noisy_data_20 = add_pink_noise(normalized_data, rms_signal, 0.2, fs);
noisy_data_40 = add_pink_noise(normalized_data, rms_signal,0.4, fs);

%%Flipping the data to double the dataset
doubled_data = normalized_data;
flipped_data = (doubled_data*-1)+1;


 %%Flipping the data to double the dataset
 normalized_data_1 = noisy_data_20;
doubled_data = normalized_data_1;
flipped_data_1 = (doubled_data*-1)+1;


%%Flipping the data to double the dataset
normalized_data_40 = noisy_data_40;
doubled_data_40 = normalized_data_40;
flipped_data_40 = (doubled_data_40*-1)+1;


%Normalizing the data between 0 and 1 and plotting
figure
subplot(2, 3, 1);
data = normalized_data;
min_val = min(data(:));
max_val = max(data(:));
data = (data - min_val) / (max_val - min_val);

normalized_data = data;
plot(t,normalized_data, 'Color',[180/255, 70/255, 90/255]); % Interpolated data
xlabel('Time');
ylabel('Data, 0 percent noise');


subplot(2, 3, 2);
data = flipped_data;
min_val = min(data(:));
max_val = max(data(:));
data = (data - min_val) / (max_val - min_val);
flipped_data = data;
plot(t,flipped_data, 'Color',[180/255, 70/255, 90/255]); % Interpolated data
xlabel('Time');
ylabel('Data, 0 percent noise');


subplot(2, 3, 3);
data = normalized_data_1;
min_val = min(data(:));
max_val = max(data(:));
data = (data - min_val) / (max_val - min_val);
normalized_data_1 = data;
```

```
plot(t,normalized_data_1, 'Color',[180/255, 70/255, 90/255]);
xlabel('Time');
ylabel('Data, 20 percent noise');



subplot(2, 3, 4);
data = flipped_data_1;
min_val = min(data(:));
max_val = max(data(:));
data = (data - min_val) / (max_val - min_val);
flipped_data_1 = data;
plot(t,flipped_data_1, 'Color',[180/255, 70/255, 90/255]);
xlabel('Time');
ylabel('Data, 20 percent noise');


subplot(2, 3, 5);
data = normalized_data_40;
min_val = min(data(:));
max_val = max(data(:));
data = (data - min_val) / (max_val - min_val);
normalized_data_40 = data;
plot(t,normalized_data_40, 'Color',[180/255, 70/255, 90/255]);
xlabel('Time');
ylabel('Data, 40 percent noise');



subplot(2, 3, 6);
data = flipped_data_40;
min_val = min(data(:));
max_val = max(data(:));
data = (data - min_val) / (max_val - min_val);
flipped_data_40 = data;
plot(t,flipped_data_40, 'Color',[180/255, 70/255, 90/255]);
xlabel('Time');
ylabel('Data, 40 percent noise');
```

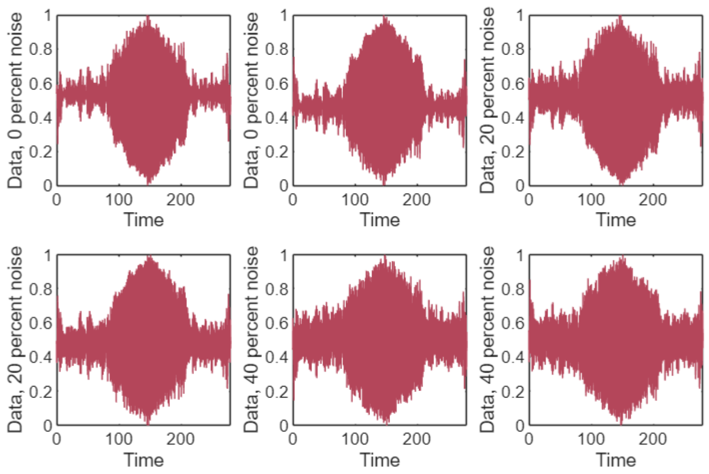

This plot shows the final post-processed data, once it has been electrode-drift corrected, flipped to double the dataset, and approximately 20% and 40%% signal acquisition noise has been added overtop.

Functions

```
function x_rs = Resting_State(mu2,mu1,nu,N)

    switch N
        case 1 % resting state on upper branch
            x_rs=mu2/(3*(mu1/2 + (mu1^2/4 - mu2^3/27)^(1/2))^(1/3)) + (mu1/2 + (mu1^2/4 -
mu2^3/27)^(1/2))^(1/3);

        case 2 % resting state on lower branch
            x_rs=- mu2/(6*(mu1/2 + (mu1^2/4 - mu2^3/27)^(1/2))^(1/3)) - (mu1/2 + (mu1^2/4 -
mu2^3/27)^(1/2))^(1/3)/2 - (3^(1/2)*i*(mu2/(3*(mu1/2 + (mu1^2/4 - mu2^3/27)^(1/2))^(1/3)) -
(mu1/2 + (mu1^2/4 - mu2^3/27)^(1/2))^(1/3)))/2;

        case 3
            x_rs= (3^(1/2)*i*(mu2/(3*(mu1/2 + (mu1^2/4 - mu2^3/27)^(1/2))^(1/3)) - (mu1/2 +
(mu1^2/4 - mu2^3/27)^(1/2))^(1/3)))/2 - (mu1/2 + (mu1^2/4 - mu2^3/27)^(1/2))^(1/3)/2 -
mu2/(6*(mu1/2 + (mu1^2/4 - mu2^3/27)^(1/2))^(1/3));
    end

end



function [E,F] = Parametrization_2PointsArc(A,B,R)

    E = A./R;

    F=cross(cross(A,B),A);
    F=F./norm(F);

end
function x = pinknoise(DIM,BETA, MAG)
%% the function pinknoise(dimension,beta,amplitude) can be used to modify noise
% parameters. The beta parameter is the slope of 1/f^beta. Beta = 0 is white
%noise, beta = -1 is pink, and -2 is brownian. This parameter can be changed to fit the slope
of
%desired noise (typically between -0.5 and 1.5), as demonstrated in Suppl Fig. 7 in Jirsa et
al. Brain 2014.
%Magnitude can be modified to fit the desired noisiness of the data


% This function generates 1/f^b spatial noise, with a normal error
% distribution
%
% DIM is a two component vector that sets the size of the spatial pattern
% (DIM=[10,5] is a 10x5 spatial grid)
%
% BETA defines the spectral distribution.
```

```
% Spectral density S(f) = N f^BETA
% (f is the frequency, N is normalisation coeff).
% BETA = 0 is random white noise.
% BETA -1 is pink noise
% BETA = -2 is Brownian noise
% The fractal dimension is related to BETA by, D = (6+BETA)/2
%
% MAG is the scaling variable for the noise amplitude
%
% The method is briefly described in Lennon, J.L. "Red-shifts and red
% herrings in geographical ecology", Ecography, Vol. 23, p101-113 (2000)
u = [(0:floor(DIM(1)/2)) -(ceil(DIM(1)/2)-1:-1:1)]'/DIM(1);
u = repmat(u,1,DIM(2));
v = [(0:floor(DIM(2)/2)) -(ceil(DIM(2)/2)-1:-1:1)]/DIM(2);
v = repmat(v,DIM(1),1);
S_f = (u.^2 + v.^2).^(BETA/2);
S_f(S_f==inf) = 0;
phi = rand(DIM);
y= S_f.^0.5 .* (cos(2*pi*phi)+i*sin(2*pi*phi));
y=y.*MAG/max(abs(y)); %set the mag to the level you want
x= ifft2(y);
x = real(x);
end
function Xdot = HysteresisLoop_Model(~,x,~,k,k_fast, alpha, R,dstar,E,F,N)

    % Parametrization of the path in the spherical parameter space in terms of great
    % circles
    mu2=R*(E(1)*cos(x(3))+F(1)*sin(x(3)));
    mu1=-R*(E(2)*cos(x(3))+F(2)*sin(x(3)));
    nu=R*(E(3)*cos(x(3))+F(3)*sin(x(3)));

    % x coordinate of resting state (i.e. upper branch of eq)
     x_rs=real(Resting_State(mu2,mu1,nu, N));
     %use this to integrate changes in alpha with the resting state
     %x_rs=real(eval_resting_state_cartesian(alpha,mu2,mu1,N));

    % equations
    xdot = -k_fast* alpha*x(2);
    ydot = -k_fast*(-(x(1)/alpha)^3 +mu2*(x(1)/alpha) +mu1 + x(2)*( nu + (x(1)/alpha) +
(x(1)/alpha)^2));
    zdot =  -k*(sqrt(((x(1)/alpha)-x_rs)^2+x(2)^2)-dstar);

    Xdot = [xdot;ydot;zdot];
end

function [E, F, C, r] = Parametrization_3PointsCircle(p1, p2, p3)
    % Calculate unit direction vectors
    p1 = p1';
    p2 = p2';
    p3 = p3';
    V12 = (p1 - p2) / norm(p1 - p2);
    V13 = (p1 - p3) / norm(p1 - p3);
```

```
    % Compute the normal vector to the plane defined by the points
    n = cross(V12, V13);
    n = n / norm(n); % Normalize the normal vector

    % Calculate the coefficients for the plane equations
    dalpha = dot(p1, n);
    dbeta = dot(V12, p1 + (p2 - p1) / 2);
    dgamma = dot(V13, p1 + (p3 - p1) / 2);

    % Set up the linear equations to find the center C
    A = [n(1), n(2), n(3);
         V12(1), V12(2), V12(3);
         V13(1), V13(2), V13(3)];

    b = [dalpha; dbeta; dgamma];

    % Solve for C using least squares
    C = A\b;

    % Calculate E (unit vector from C to p1)
    E = (p1 - C) / norm(p1 - C);

    % Calculate F (perpendicular vector)
    F = -cross(E, n);

    % Calculate the radius r
    r = norm(p1 - C);
end

function [Xdot, mu2, mu1,nu] = SlowWave_Model(~,x,~,k,k_fast,alpha, E,F,C,r)

    % Parametrization of the path in the spherical parameter space in terms
    % of a circle defined by 3 points
    mu2=C(1)+r*(E(1)*cos(x(3))+F(1)*sin(x(3)));
    mu1=-(C(2)+r*(E(2)*cos(x(3))+F(2)*sin(x(3))));
    nu=C(3)+r*(E(3)*cos(x(3))+F(3)*sin(x(3)));

    % System
    xdot = -k_fast* alpha*x(2);
    ydot = -k_fast*(-(x(1)/alpha)^3 +mu2*(x(1)/alpha) +mu1 + x(2)*( nu + (x(1)/alpha) +
(x(1)/alpha)^2));
    zdot = k;



    Xdot = [xdot;ydot;zdot];
end

function get_plot()
    marker_size = 10;
    load('curves.mat')
```

```
    load('curves2.mat')
    load('bifurcation_crossing.mat')
    load('sphere_mesh.mat')
    load('testmesh.mat');

    hold on;
    linewidth = 2;

    % Plot different meshes with assigned DisplayName for the legend
    vertices = BCSmesh.vertices;
    faces = BCSmesh.faces;
    h1 = patch('Vertices', vertices, 'Faces', faces, ...
          'FaceColor', [0.973, 0.965, 0.722], 'EdgeColor', 'none', 'FaceAlpha', 0.6,
'DisplayName', 'BCS Mesh');

    vertices = Active_restmesh.vertices;
    faces = Active_restmesh.faces;
    h2 = patch('Vertices', vertices, 'Faces', faces, ...
          'FaceColor', [0.9216, 0.9216, 0.9216], 'EdgeColor', 'none', 'FaceAlpha', 0.6,
'DisplayName', 'Active Rest Mesh');

    vertices = Seizure_mesh.vertices;
    faces = Seizure_mesh.faces;
    h3 = trisurf(faces, vertices(:,1), vertices(:,2), vertices(:,3), ...
            'FaceColor', [0.894, 0.706, 0.831], 'EdgeColor', 'none', 'FaceAlpha', 0.3,
'DisplayName', 'Seizure Mesh');

    vertices = Bistable_Lcb_mesh.vertices;
    faces = Bistable_Lcb_mesh.faces;
    h4 = patch('Vertices', vertices, 'Faces', faces, ...
          'FaceColor', [0.973, 0.965, 0.722], 'EdgeColor', 'none', 'FaceAlpha', 0.6,
'DisplayName', 'Bistable Lcb Mesh');

    scale_array = [0.4];
    scale_array = scale_array / 0.4;

    % Arrays to store plot handles for the legend
    legend_handles = [h1, h2, h3];
    legend_names = {'Rest/ Seizure Bistable region', 'Active Rest/Rest Bistable region',
'Seizure region'};

    % Scale factors to adjust radius from 0.4
    for i = 1:length(scale_array)
        % Scale the coordinates of the points for radius 0.39
        Fold_of_cycles_scaled = scale_array(i) * Fold_of_cycles;
        Homoclinic_to_saddle3_scaled = scale_array(i) * Homoclinic_to_saddle3;
        Homoclinic_to_saddle2_scaled = scale_array(i) * Homoclinic_to_saddle2;
        Homoclinic_to_saddle1_scaled = scale_array(i) * Homoclinic_to_saddle1;
        Homoclinic_to_saddle_scaled = scale_array(i) * Homoclinic_to_saddle;
        Fold_scaled = scale_array(i) * Fold;
        Hopf_scaled = scale_array(i) * Hopf;
        SNIC_scaled = scale_array(i) * SNIC;
```

```
        % Plot all scaled lines with DisplayName for the legend
        h5 = plot3(Fold_of_cycles_scaled(1, :), Fold_of_cycles_scaled(2, :),
Fold_of_cycles_scaled(3, :), 'Color', [0.9725,0.2667,0.5843], 'LineWidth', linewidth,
'DisplayName', 'Fold of Cycles');
        h6 = plot3(Homoclinic_to_saddle3_scaled(1, :), Homoclinic_to_saddle3_scaled(2, :),
Homoclinic_to_saddle3_scaled(3, :), 'Color', [0.404, 0.702, 0.851], 'LineWidth', linewidth,
'LineStyle', '--', 'DisplayName', 'Homoclinic to Saddle 3');
        h7 = plot3(Homoclinic_to_saddle2_scaled(1, :), Homoclinic_to_saddle2_scaled(2, :),
Homoclinic_to_saddle2_scaled(3, :), 'Color', [0.404, 0.702, 0.851],  'LineWidth', linewidth,
'DisplayName', 'Homoclinic to Saddle 2');
        h8 = plot3(Homoclinic_to_saddle1_scaled(1, :), Homoclinic_to_saddle1_scaled(2, :),
Homoclinic_to_saddle1_scaled(3, :), 'Color', [0.404, 0.702, 0.851],  'LineWidth', linewidth,
'LineStyle', '--', 'DisplayName', 'Homoclinic to Saddle 1');
        h9 = plot3(Homoclinic_to_saddle_scaled(1, :), Homoclinic_to_saddle_scaled(2, :),
Homoclinic_to_saddle_scaled(3, :), 'Color', [0.404, 0.702, 0.851],  'LineWidth', linewidth,
'DisplayName', 'Homoclinic to Saddle');
        h10 = plot3(Fold_scaled(1, 140:564), Fold_scaled(2, 140:564), Fold_scaled(3, 140:564),
'Color', [0.957, 0.612, 0.204], 'LineWidth', linewidth, 'DisplayName', 'Fold Part 1');
        h11 = plot3(Fold_scaled(1, 575:end), Fold_scaled(2, 575:end), Fold_scaled(3, 575:end),
'Color', [0.957, 0.612, 0.204], 'LineWidth', linewidth, 'DisplayName', 'Fold Part 2');
        h12 = plot3(Fold_scaled(1, 1:80), Fold_scaled(2, 1:80), Fold_scaled(3, 1:80), 'Color',
[0.957, 0.612, 0.204], 'LineWidth', linewidth, 'DisplayName', 'Fold Part 3');
        h13 = plot3(Hopf_scaled(1, 1:400), Hopf_scaled(2, 1:400), Hopf_scaled(3, 1:400),
'Color',[0.4549 ,0.7490 ,0.2706], 'LineWidth', linewidth, 'LineStyle', '--', 'DisplayName',
'Hopf Part 1');
        h14 = plot3(Hopf_scaled(1, 400:973), Hopf_scaled(2, 400:973), Hopf_scaled(3, 400:973),
'Color',[0.4549 ,0.7490 ,0.2706], 'LineWidth', linewidth, 'DisplayName', 'Hopf Part 2');
        h15 = plot3(SNIC_scaled(1, :), SNIC_scaled(2, :), SNIC_scaled(3, :), 'Color',[0.957,
0.612, 0.204], 'LineWidth', linewidth, 'LineStyle', '--', 'DisplayName', 'SNIC');

        % Add line handles to the legend array
        legend_handles = [legend_handles, h5,   h9,  h12, h13, h14, h15];
        legend_names = [legend_names, 'Fold Limit Cycle', 'SH', 'SN', 'SubH', 'SupH', 'SNIC'];
    end

    % Add the sphere mesh with transparency
    surf(X_sphere, Y_sphere, Z_sphere, 'FaceColor', [0.96, 0.96, 0.86], 'FaceAlpha', 0.2,
'EdgeColor', 'none', 'HandleVisibility', 'off');

    % Add labels and view adjustment
    xlabel('\mu_2');
    ylabel('-\mu_1');
    zlabel('\nu');

    lineVector = [-0.19, 0.2, 0.07];
    az = atan2d(lineVector(2), lineVector(1)); % Azimuth angle
    el = atan2d(lineVector(3), norm(lineVector(1:2))); % Elevation angle
    view(az, el);

    % Display the legend with all handles and names
    legend(legend_handles, legend_names);
```

```
end


function   [onset_curve,offset_curve]=hysteresis_random_path(bifurcation)


load('curves.mat');


if bifurcation == '2s'
    onset_curve=SNr_LCs;
    offset_curve=SHl;

end


if bifurcation=='2b'
    onset_curve=SNr_LCb;
    offset_curve=SHb;

end



if bifurcation=='3s'
    onset_curve=SNr_LCs;
    offset_curve=SNl_ActiveRest;
end



if bifurcation=='4b'
    onset_curve=SNr_LCb;
    offset_curve=FLC_top;
end

if bifurcation=='10'
    onset_curve=SNr_ActiveRest;
    offset_curve=SHl;
end


if bifurcation=='11'


    onset_curve= SNr_ActiveRest;
    offset_curve=[[0.3171; -0.066; 0.2347], [0.3115; -0.0546; 0.2450],[0.3166; -0.0654;
0.2356]];%SNl_ActiveRest;
end

if bifurcation=='14'
    onset_curve=subH;
```

```
    offset_curve=SHb;
end


if bifurcation=='16'
    onset_curve=subH;
    offset_curve=FLC;
end














end


function [onset_curve,offset_curve,offset_curve2, flag] = slow_wave_circular_random_path(I)

load('curves.mat');
load('curves2.mat');


if I == 6
    onset_curve = SHl(:,50:104); %55
    offset_curve = [0.33, 0.11, 0.18]';
    offset_curve2 = SNIC(:,1:35); %35
    flag = 1;
end


if I == 5
    onset_curve = SNIC; %44
    offset_curve = SNIC; %44
    offset_curve2 = [0.34,0.14,0.06]';
    flag = 2;
end


if I == 1
    onset_curve = SNIC(:,1:35); %35
    offset_curve = [0.33, 0.11, 0.18]';
    offset_curve2 = SNr_ActiveRest; %55
    flag = 1;
end
```

```
if I == 7
    onset_curve=SNIC; %44
    offset_curve = Hopf(:,800:855); %56
    offset_curve2 = [0.36,-0.12,0.12]';
    flag = 3;
end


if I == 8
    onset_curve=SNIC; %44
    offset_curve = [0.34,0.2,-0.06]';
    offset_curve2 = FLC(:,100:300); %201
    flag = 1;
end


if I == 9
    onset_curve= Hopf(:,800:855); %56
    offset_curve = SNIC; %44
    offset_curve2 = [0.36,-0.12,0.12]';
    flag = 3;
end


if I == 12
    onset_curve= Hopf(:,450:495);
    offset_curve = FLC(:,60:100);
    offset_curve2 = [-0.3, -0.2, -0.2]';
    flag = 3;
end


if I == 13
    onset_curve = Hopf(:,450:495); %201
    offset_curve = [0.34,0.2,-0.06]';
    offset_curve2 = SNIC;
    flag = 1;
end


if I == 15
    onset_curve= subH;%41
    offset_curve = Hopf(:,450:495);%46
    offset_curve2 = [-0.3, -0.2, -0.2]';
    flag = 3;
end
```

```
end
function [Xdot, mu2,mu1,nu] = SlowWave_Model_piecewise(~,x,~,k,k_fast, alpha, mu2,mu1,nu)
% Parametrization of the path in the spherical parameter space in terms
% of a circle defined by 3 points
% System
xdot = -k_fast* alpha*x(2);
ydot = -k_fast*(-(x(1)/alpha)^3 +mu2*(x(1)/alpha) +mu1 + x(2)*( nu + (x(1)/alpha) +
(x(1)/alpha)^2));
zdot = k;
Xdot = [xdot;ydot;zdot];
end
function [mu2,mu1,nu, theta] = sphereArcPath(k, tstep,point1, point2)
% sphereArcPath - Generates an arc path between two points on a sphere
%
% Syntax: arcPath = sphereArcPath(point1, point2, numPoints)
%
% Inputs:
% point1 - [x1, y1, z1] Coordinates of the first point on the sphere
% point2 - [x2, y2, z2] Coordinates of the second point on the sphere
% numPoints - Number of points along the arc
%
% Outputs:
% arcPath - An Nx3 matrix containing the coordinates of points along the arc
% Check the input points
radius = 0.4;
% if norm(point1) ~= radius || norm(point2) ~= radius
% error('The points must lie on the sphere of radius 0.4.');
% end
% Normalize the input points to make sure they are on the sphere
point1 = point1 / norm(point1) * radius;
point2 = point2 / norm(point2) * radius;
% Compute the quaternion for rotation
theta = acos(dot(point1, point2) / (radius^2));
axis = cross(point1, point2);
if norm(axis) == 0
error('The points are the same or antipodal.');
end
axis = axis / norm(axis);
% Compute points along the arc
numPoints = floor((theta/k)/tstep);
arcPath = zeros(numPoints, 3);
for i = 0:numPoints-1
t = i / (numPoints - 1);
angle = t * theta;
R = rotationMatrix(axis, angle);
arcPath(i+1, :) = (R * point1')';
end
mu2 = arcPath(:,1)';
mu1 = arcPath(:,2)';
nu = arcPath(:,3)';
end
```

```
function R = rotationMatrix(axis, angle)
% rotationMatrix - Generates a rotation matrix given an axis and an angle
%
% Syntax: R = rotationMatrix(axis, angle)
%
% Inputs:
% axis - A 3-element vector representing the axis of rotation
% angle - The angle of rotation in radians
%
% Outputs:
% R - A 3x3 rotation matrix
ux = axis(1);
uy = axis(2);
uz = axis(3);
c = cos(angle);
s = sin(angle);
t = 1 - c;
R = [t*ux*ux + c, t*ux*uy - s*uz, t*ux*uz + s*uy;
t*ux*uy + s*uz, t*uy*uy + c, t*uy*uz - s*ux;
t*ux*uz - s*uy, t*uy*uz + s*ux, t*uz*uz + c];
end
function point= get_random_point
radius = 0.4;
% Generate two random numbers
theta = 2 * pi * rand(); % Random angle between 0 and 2*pi
phi = acos(2 * rand() - 1); % Random angle between 0 and pi
% Convert spherical coordinates to Cartesian coordinates
x = radius * sin(phi) * cos(theta);
y = radius * sin(phi) * sin(theta);
z = radius * cos(phi);
point = [x,y,z];
% Display the point
end
function point= get_random_point_hopf
%load("map_regions.mat")
radius = 0.4;
% Loop until a valid point with y > 0 is found
while true
% Generate two random numbers
theta = 2 * pi * rand(); % Random angle between 0 and 2*pi
phi = acos(2 * rand() - 1); % Random angle between 0 and pi
% Convert spherical coordinates to Cartesian coordinates
x = radius * sin(phi) * cos(theta);
y = radius * sin(phi) * sin(theta);
z = radius * cos(phi);
% Check if y is positive
if y > 0
point = [x, y, z];
break;
end
end
% Display the point
```

```
end
%


% Display the point
function point= get_random_point_fixed
radius = 0.4;
% Loop until a valid point with y > 0 is found
while true
% Generate two random numbers
theta = 2 * pi * rand(); % Random angle between 0 and 2*pi
phi = acos(2 * rand() - 1); % Random angle between 0 and pi
% Convert spherical coordinates to Cartesian coordinates
x = radius * sin(phi) * cos(theta);
y = radius * sin(phi) * sin(theta);
z = radius * cos(phi);
% Check if y is positive
if y < 0
point = [x, y, z];
break;
end
end
% Display the point
end


function [mu2,mu1,nu] = sphere(point1, point2, numPoints)
% sphereArcPath - Generates an arc path between two points on a sphere
%
% Syntax: arcPath = sphereArcPath(point1, point2, numPoints)
%
% Inputs:
% point1 - [x1, y1, z1] Coordinates of the first point on the sphere
% point2 - [x2, y2, z2] Coordinates of the second point on the sphere
% numPoints - Number of points along the arc
%
% Outputs:
% arcPath - An Nx3 matrix containing the coordinates of points along the arc
% Check the input points
radius = 0.4;
% if norm(point1) ~= radius || norm(point2) ~= radius
% error('The points must lie on the sphere of radius 0.4.');
% end
% Normalize the input points to make sure they are on the sphere
point1 = point1 / norm(point1) * radius;
point2 = point2 / norm(point2) * radius;
% Compute the quaternion for rotation
theta = acos(dot(point1, point2) / (radius^2));
axis = cross(point1, point2);
if norm(axis) == 0
error('The points are the same or antipodal.');
end
```

```
axis = axis / norm(axis);
% Compute points along the arc
arcPath = zeros(numPoints, 3);
for i = 0:numPoints-1
t = i / (numPoints - 1);
angle = t * theta;
R = rotationMatrix(axis, angle);
arcPath(i+1, :) = (R * point1')';
end
mu2 = arcPath(:,1)';
mu1 = arcPath(:,2)';
nu = arcPath(:,3)';
end

function [arcLength, theta] = calculateArcLength(P1, P2, radius)
 % calculateArcLength computes the arc length and central angle between two points on a
sphere.
 %
 % Input:
 % P1 - First point [x1, y1, z1]
 % P2 - Second point [x2, y2, z2]
 % radius - Radius of the sphere (default: 0.4 if not provided)
 %
 % Output:
 % arcLength - Arc length between the two points
 % theta - Central angle between the two points in radians

 if nargin < 3
 radius = 0.4;
 end

 % Compute the dot product of P1 and P2
 dotProduct = dot(P1, P2);

 % Compute the magnitudes of P1 and P2
 magnitudeP1 = norm(P1);
 magnitudeP2 = norm(P2);

 % Compute the cosine of the central angle
 cosTheta = dotProduct / (magnitudeP1 * magnitudeP2);

 % Compute the central angle in radians
 theta = acos(cosTheta);

 % Compute the arc length
 arcLength = radius * theta;
end



function   [p0,p1,p1_5,p2,p3]=piecewise_random_path(bifurcation)
```

```
load('curves.mat');
load('bifurcation_crossing.mat')
load("curves2.mat")

if bifurcation==3
    %fixed rest point
    p0 = Hopf(:,930)';
    %bifurcation curve
    randomNumber = randi([145,170]);
    p1 = Fold(:,145:170)';
    randomNumber2 = randi([600,750]);
   p1_5 = [0.3196, 0.2389, -0.0279];
    %bifurcation curve
    p2 = Hopf(:,600:750)' ;
    %fixed rest
    p3 = [ 0.1944 , 0.0893 , 0.3380];

end

if bifurcation==7
    randomNumber = randi([600,750]);
 randomNumber2 = randi([1,44]);
%fixed rest point
p0 = Fold(:,400)';
%bifurcation curve
p1 =  SNIC' ;
%random point in limit cycle
p1_5 = [0.1314, 0.3298, -0.1843];
%bifurcation curve
p2 = Hopf(:,600:750)';
%fixed rest
p3 = [ 0.1944 , 0.0893 , 0.3380];
end

if bifurcation==9
    randomNumber = randi([600,750]);
    %fixed rest point
    p0 = [ 0.1944 , 0.0893 , 0.3380];
    %bifurcation curve
    p1 = Hopf(:,600:750)';
    %change here
    randomNumber2 = randi([1,44]);
    %random point in limit cycle
    p1_5 = [-0.0441, 0.2591, -0.3015];
    %bifurcation curve
    p2 = SNIC' ;
    %fixed rest
    p3 = Fold(:,450)';
end

if bifurcation==10
    randomNumber = randi([600,750]);
```

```
    %fixed rest point
    p0 = [ 0.1944 , 0.0893 , 0.3380];
    %bifurcation curve
    p1 = Hopf(:,600:750)';%get_nearest_hopf(p0(1),p0(2),p0(3))';
    %change here
    randomNumber2 = randi([1,124]);
    %random point in limit cycle
    p1_5 = [-0.123686721647726      0.338825918756816  -0.172912092308889];
    %bifurcation curve
    p2 = Homoclinic_to_saddle' ;
    %fixed rest
    p3 = Fold(:,400)';
end

if bifurcation==11
     randomNumber = randi([600,750]);
%fixed rest point
p0 = [ 0.1944 , 0.0893 , 0.3380];
%bifurcation curve
p1 = Hopf(:,600:750)';
%change here
randomNumber2 = randi([600,750]);
%random point in limit cycle
p1_5 = [-0.2104, 0.3180, -0.1209];
%bifurcation curve
p2 = Hopf(:,600:750)' ;
%fixed rest
p3 = [ 0.1944 , 0.0893 , 0.3380];
end


end

function [new_X, new_Y, new_Z] = get_random_walk(p1,p2,region,k,tstep, minSteps, maxSteps)
load('logicgrid.mat')
% if region == 11
% load('logicgrid.mat'); % Load the logic grid from the .mat file
%
% elseif region == 'LCS_bistable'
%         load("logicgrid_bistable_lcs.mat")
%
% elseif region == 'LCB_bistable'
%         load("logicgrid_bistable_lcb.mat")
% end
grid = logic_grid;

[theta, phi, r] = cart2sph(p1(1), p1(2), p1(3));
theta_offset_deg = -40;  % For example, a 30-degree rotation

% Convert the offset to radians
theta_offset_rad = deg2rad(theta_offset_deg);
theta_deg_p1 = rad2deg(theta+theta_offset_rad);
```

```
phi_deg_p1 = rad2deg(phi);

p1_5 = get_nearest_seizure_point(theta_deg_p1, phi_deg_p1, region);
[theta, phi, r] = cart2sph(p2(1), p2(2), p2(3));
theta_offset_deg = -40;  % For example, a 30-degree rotation

% Convert the offset to radians
theta_offset_rad = deg2rad(theta_offset_deg);
theta_deg_p2 = rad2deg(theta+theta_offset_rad);
phi_deg_p2 = rad2deg(phi);
p2_5 = get_nearest_seizure_point(theta_deg_p2, phi_deg_p2, region);
% Load the grid (assuming the variable is 'grid')



% Define the start and end points
startPoint = p1_5; % Define the start point (row, column)
endPoint = p2_5; % Define the end point (row, column)

 currentPos = startPoint;
%
% % Store the path
 path = currentPos;
%
% % Define the movement directions (up, down, left, right)
directions = [0 1; 0 -1; 1 0; -1 0]; % [row_change, col_change]


% Get the size of the grid
[gridRows, gridCols] = size(grid);

% Loop until a valid path is found
% Loop until a valid path is found
while true
    % Initialize the current position at the start point
    currentPos = startPoint;

    % Store the path, starting from the initial position
    path = currentPos;
    stepCount = 0;

    % Perform the random walk until either endpoint is reached or maxSteps
    while stepCount < maxSteps
        % Check if the endpoint has been reached and steps are within bounds
        if isequal(currentPos, endPoint) && stepCount >= minSteps
            disp('Valid path found within the specified range of steps.');
            break;
        end

        % Choose a random direction to move
        randomDir = directions(randi(4), :);
```

```
        % Compute the new position
        newPos = currentPos + randomDir;

        % Check if the new position is within bounds and valid (e.g., grid value is 1)
        if newPos(1) > 0 && newPos(1) <= gridRows && newPos(2) > 0 && newPos(2) <= gridCols
            if grid(newPos(1), newPos(2)) == 1
                % Update the current position
                currentPos = newPos;

                % Add the new position to the path
                path = [path; currentPos];

                % Increment the step count
                stepCount = stepCount + 1;
            end
        end
    end

    % Check if a valid path was found within bounds
    if isequal(currentPos, endPoint) && stepCount >= minSteps && stepCount <= maxSteps
        % Exit the loop if a valid path is found
        break;
    end

    % If not, repeat the simulation
end
% Plot the random walk path
figure;
imagesc(grid); % Show the grid
colormap(gray); % Use a gray color map (1 = white, 0 = black)
hold on;
plot(path(:,2), path(:,1), 'r.-', 'LineWidth', 2, 'MarkerSize', 15); % Plot the path
% Plot the start point in green
plot(startPoint(2), startPoint(1), 'go', 'MarkerSize', 10, 'MarkerFaceColor', 'g');

% Plot the end point in blue
plot(endPoint(2), endPoint(1), 'bo', 'MarkerSize', 10, 'MarkerFaceColor', 'b');
title('2D Random Walk Path on the Logic Grid');
xlabel('X');
ylabel('Y');



 if region == 11
load('logicgrid.mat'); % Load the logic grid from the .mat file

elseif region == 'LCS_bistable'
        load("logicgrid_bistable_lcs.mat")

elseif region == 'LCB_bistable'
        load("logicgrid_bistable_lcb.mat")
end
```

```
    % Convert back to x, y coordinates
    x_back = (path(:,1) - 1) / (grid_size(1) - 1) * (x_max - x_min) + x_min;
    y_back = (path(:,2) - 1) / (grid_size(2) - 1) * (y_max - y_min) + y_min;




    theta_rad = deg2rad(x_back) + deg2rad(-theta_offset_deg);
phi_rad = deg2rad(y_back);
radius = 0.4; % Set the radius of the sphere


[X, Y, Z] = sph2cart(theta_rad, phi_rad, radius);

r = 0.4;
P1 = [X(1);Y(1);Z(1)];
P2 = [X(2);Y(2);Z(2)];
% Compute the angle between the two points using the dot product formula
cos_theta = dot(P1, P2) / (r^2);
theta = acos(cos_theta);  % Angle in radians

total_theta = (length(X)-1)*theta;

numPoints = floor((total_theta/k)/tstep);
% Create interpolation queries and interpolate X, Y, Z coordinates
xq = linspace(1, length(X), numPoints); % Query points for interpolation

new_X = interp1(1:length(X), X, xq, 'linear');
new_Y = interp1(1:length(Y), Y, xq, 'linear');
new_Z = interp1(1:length(Z), Z, xq, 'linear');



end


function noisy_signal = add_pink_noise(signal, rms_signal, noise_amplitude_ratio, fs)
    % Inputs:
    % signal - input signal (1D array)
    % noise_amplitude_ratio - fraction of signal amplitude for noise (e.g., 0.4 for 40%)

    % Compute the RMS amplitude of the signal


    % Generate pink noise of the same length as the signal
    % Pink noise can be generated using dsp.ColoredNoise in MATLAB
    L = length(signal);
    pink_noise = pinknoise([1,L],-1,10000)';
```

```
    %
    % % % Scale the noise so its amplitude is noise_amplitude_ratio of the signal's amplitude
    % scaling_factor = noise_amplitude_ratio * (1 / rms_noise);
    % scaled_noise = pink_noise * scaling_factor;
    min_val = min(pink_noise(:));
max_val = max(pink_noise(:));
scaled_noise = noise_amplitude_ratio*(pink_noise - min_val) / (max_val - min_val);
min_val = min(signal(:));
max_val = max(signal(:));
scaled_signal = (signal - min_val) / (max_val - min_val);
    % Add the scaled noise to the original signal
    noisy_signal = scaled_signal + scaled_noise;
end

function amp = get_amp(signal, fs)
hpFilt = designfilt('highpassfir', 'FilterOrder', 5, ...
                   'CutoffFrequency', 5, 'SampleRate', fs);

signal= filter(hpFilt, signal);
[peaks,locs] = findpeaks(signal ,'MinPeakProminence', 0.15);
[troughs_neg,locs_troughs] = findpeaks(signal, 'MinPeakProminence', 0.15);
troughs = -1*troughs_neg;

newnew = [];
len = 0;
if length(troughs) > length(peaks)
    len = length(peaks);
else
    len = length(troughs);
end
for i = 1:len
    newnew = [newnew; -1*troughs(i) + peaks(i)];
end
amp = mean(newnew);
end

function [start_index, stop_index, signal] = bifurcation_all_class(class, tstep, sigma)
R = 1;
N = 1;
b = 0;
x0 = [0;0;0];
k =0.007;
dstar = 0.3;
tstep = 0.1;
tmax = 75000;
if ismember(class, [1 5 6 8 12 13 15])
    % Code for class 1
    % Perform specific operations for class 1
    [onset_curve,offset_curve,p3, flag] = slow_wave_circular_random_path(class);
    onset_curve_length=length(onset_curve);
    offset_curve_length=length(offset_curve);
    random_onset_index=randsample(onset_curve_length,1);
```

```
    random_offset_index=randsample(offset_curve_length,1);
    if flag == 2 || flag == 3

        p1 = onset_curve(:,random_onset_index);
        p2 = offset_curve(:,random_offset_index);
    else
        p1 = onset_curve(:,random_onset_index);
        p2 = p3(:,random_offset_index);
        p3 = offset_curve;
    end



% uncomment this code to do random path
% % One random path - select random point on onset curve and offset curve

tspan = 0:tstep:tmax;

% Create circular path based 3 defining points
[E, F, C, r] = Parametrization_3PointsCircle(p1',p2',p3');
if class == 13
    E = -E;
end


N_t = length(tspan);
X = zeros(3,N_t);
xx = x0;
sigma = 40;
Rn = [pinknoise([1,N_t],-1, sigma);pinknoise([1,N_t],-1, 00);pinknoise([1,N_t],-1, 00)];
mu2_big = zeros(1, length(N_t));
mu1_big = zeros(1, length(N_t));
nu_big = zeros(1, length(N_t));

for n = 1:N_t
    %Euler-Meruyama method
    [Fxx, mu2, mu1,nu] = SlowWave_Model(tspan(n),xx,b,k,E,F,C,r);
    xx = xx + tstep*Fxx + sqrt(tstep)*Rn(:,n);
    X(:,n) = xx;
     mu2_big(n) = mu2;
    mu1_big(n) = mu1;
    nu_big(n) = nu;


end

x = X';
t = tspan;

%%Onset and offset calculation, calculates radians to the bifurcation
%%curve, then uses tstep and k variables to compute onset location
        plot_onset_offset = 0;
```

```
        if(floor((((2*pi)/k)/tstep)) < N_t)
            plot_onset_offset = 1;
        point1 = p1' - C;
        point2 = p2' - C;
        point3 = p3'-C;
        point1 = point1 / norm(point1) * r;
        point2 = point2 / norm(point2) * r;

        point3 = point3 / norm(point3) * r;
        % Compute the quaternion for rotation
        theta1 = acos(dot(point1, point2) / (r^2));
        %%%change here
        numPoints1 = floor((theta1/k)/tstep);
        point = [mu2_big(numPoints1), -mu1_big(numPoints1), nu_big(numPoints1)];
        if round(point,2) == round(p2,2)'
        onset_index = numPoints1;
        else
        numPoints1 = floor(((2*pi - theta1)/k)/tstep);
        onset_index = numPoints1;
        theta1 = theta1-2*pi;
        end
        theta2 = acos(dot(point1, point3) / (r^2));
        numPoints2 = floor(((theta2)/k)/tstep);
        point = [mu2_big(numPoints2), -mu1_big(numPoints2), nu_big(numPoints2)];
        if round(point,2) == round(p2,2)'
        %offset_index = numPoints2;
        else
        numPoints2 = floor(((2*pi  - theta2)/k)/tstep);
        theta2 = 2*pi - theta2;
        offset_index = numPoints2;
        end
        theta3 = 2*pi;
        numPoints3 = floor(((theta3)/k)/tstep);
        point = [mu2_big(numPoints3), -mu1_big(numPoints3), nu_big(numPoints3)];
        offset_index = numPoints3;
        end
        if class == 15 || class == 12
             onset_index_temp = onset_index;
        onset_index = offset_index;
        offset_index = onset_index_temp + floor((((2*pi)/k)/tstep));
        end
        start_index = max(1,onset_index-1000);
        stop_index = min(offset_index+1000, length(x));
        signal = x(start_index:stop_index,1);


elseif ismember(class, ['2s' '2b' '4b' '14' '16'])
    k=0.01;
    tmax = 75000;
    dstar = 0.3;
   [onset_curve,offset_curve]=hysteresis_random_path(class);
   onset_curve_length=length(onset_curve);
```

```
offset_curve_length=length(offset_curve);



% uncomment this code to do random path
% % One random path - select random point on onset curve and offset curve
random_onset_index=randsample(onset_curve_length,1);
random_offset_index=randsample(offset_curve_length,1);
A = offset_curve(:,random_offset_index);
B = onset_curve(:,random_onset_index);
    tspan = 0:tstep:tmax;

% Create circular path based 3 defining points
[E, F] = Parametrization_2PointsArc(A,B,R);

N_t = length(tspan);
X = zeros(3,N_t);
xx = x0;


Rn = [pinknoise([1,N_t],-1, sigma);pinknoise([1,N_t],-1, 00);pinknoise([1,N_t],-1, 00)];

for n = 1:N_t

    % Euler-Meruyama method
    Fxx = HysteresisLoop_Model(tspan(n),xx,b,k,R,dstar,E,F,N);
    xx = xx + tstep*Fxx + sqrt(tstep)*Rn(:,n);
    X(:,n) = xx;

end

x = X';

[pks,times]=findpeaks(x(:,3),'MinPeakProminence',0.03);
onset_time = times*tstep;

% Calculate Offset Times
[pks2,times2]=findpeaks(-x(:,3),'MinPeakProminence',0.03);
offset_time = times2*tstep;


% Single seizure
if offset_time(1)>onset_time(1) % if system starts at rest
    start_index = times(1)-10000;
    stop_index = times2(1)+10000;
    start_index = max(1, start_index);
    stop_index = min(length(x), stop_index);
    signal = x(start_index:stop_index,1);
    onset = times(1);
    offset = stop_index-start_index-10000;
else % if system starts in a seizure
    start_index = times(1)-10000;
```

```
    stop_index = times2(2)+10000;
    start_index = max(1, start_index);
    stop_index = min(length(x), stop_index);
    signal = x(start_index:stop_index,1);
    onset = times(1);
    offset = stop_index-start_index-10000;
end




elseif ismember(class, [3 7 9 10 11])
    k = 0.005;
    [p0,onset_curve,p1_5,offset_curve,p3]=piecewise_random_path(class);
onset_curve_length=length(onset_curve);
offset_curve_length=length(offset_curve);
onset_curve = onset_curve';
offset_curve = offset_curve';


% uncomment this code to do random path
% % One random path - select random point on onset curve and offset curve
random_onset_index=randsample(onset_curve_length,1);
random_offset_index=randsample(offset_curve_length,1);
p1 = onset_curve(:,random_onset_index)';
p2 = offset_curve(:,random_offset_index)';


stall_val = 30000;
[mu2_straight_path0,mu1_straight_path0,nu_straight_path0,rad1] = sphereArcPath(k,tstep,p0,p1);
[mu2_straight_path0_5,mu1_straight_path0_5,nu_straight_path0_5,rad2] =
sphereArcPath(k,tstep,p1,p1_5);
points = repmat(p1_5, stall_val, 1)';
%path noise sigma
sigma_pathnoise = 100;
Rn = [pinknoise([1,length(points)],-1, sigma_pathnoise);pinknoise([1,length(points)],-1,
sigma_pathnoise);pinknoise([1,length(points)],-1, sigma_pathnoise)];
points = points + Rn;
[mu2_straight_path,mu1_straight_path,nu_straight_path,rad3] = sphereArcPath(k,tstep,p1_5,p2);
[mu2_straight_path1,mu1_straight_path1,nu_straight_path1,rad4] = sphereArcPath(k,tstep,p2,p3);
mu2_all = [mu2_straight_path0, mu2_straight_path0_5, points(1, :), mu2_straight_path,
mu2_straight_path1];
mu1_all = [mu1_straight_path0, mu1_straight_path0_5, points(2, :), mu1_straight_path,
mu1_straight_path1];
mu1_all = -mu1_all;
nu_all = [nu_straight_path0, nu_straight_path0_5, points(3,:), nu_straight_path,
nu_straight_path1];

N_t = length(mu2_all);
X = zeros(3,N_t);
xx = x0;
```

```
Rn = [pinknoise([1,N_t],-1, sigma);pinknoise([1,N_t],-1, 00);pinknoise([1,N_t],-1, 00)];
mu2_big = zeros(1, length(N_t));
mu1_big = zeros(1, length(N_t));
nu_big = zeros(1, length(N_t));

%%get onset index by finding Radians to bifurcation, and getting index
%%through k and tstep parameters
onset_index = floor((rad1/k)/tstep);
offset_index = floor(((rad1+rad2+rad3)/k)/tstep) + stall_val;
for n = 1:N_t
    %%Euler-Meruyama method
    [Fxx,mu2,mu1,nu] = SlowWave_Model_piecewise(0,xx,b,k,mu2_all(n), mu1_all(n),nu_all(n));
    xx = xx + tstep*Fxx + sqrt(tstep)*Rn(:,n);
    X(:,n) = xx;
    mu2_big(n) = mu2;
    mu1_big(n) = mu1;
    nu_big(n) = nu;
end
x = X';
signal = x(:,1);
start_index = 1;
stop_index = length(signal);
end



end

function x_rs=eval_resting_state_cartesian(a,mu2,mu1,N)

switch N
    case 1 % resting state
        x_rs = ((a.^3 .* mu1)./2 + ((a.^6 .* mu1.^2)./4 - (a.^6 .* mu2.^3)./27).^(1/2)).^(1/3)
+ ...
       (a.^2 .* mu2) ./ (3 .* ((a.^3 .* mu1)./2 + ((a.^6 .* mu1.^2)./4 - (a.^6 .*
mu2.^3)./27).^(1/2)).^(1/3));

    case 2
        x_rs = - (sqrt(3) .* (((a.^3 .* mu1)./2 + ((a.^6 .* mu1.^2)./4 - (a.^6 .*
mu2.^3)./27).^(1/2)).^(1/3) - (a.^2 .* mu2) ./ (3 .* ((a.^3 .* mu1)./2 + ((a.^6 .* mu1.^2)./4 -
(a.^6 .* mu2.^3)./27).^(1/2)).^(1/3))) .* 1i) ./ 2 ...
      - ((a.^3 .* mu1)./2 + ((a.^6 .* mu1.^2)./4 - (a.^6 .* mu2.^3)./27).^(1/2)).^(1/3) ./ 2
...
      - (a.^2 .* mu2) ./ (6 .* ((a.^3 .* mu1)./2 + ((a.^6 .* mu1.^2)./4 - (a.^6 .*
mu2.^3)./27).^(1/2)).^(1/3));

    case 3
        x_rs = (sqrt(3) .* (((a.^3 .* mu1)./2 + ((a.^6 .* mu1.^2)./4 - (a.^6 .*
mu2.^3)./27).^(1/2)).^(1/3) - (a.^2 .* mu2) ./ (3 .* ((a.^3 .* mu1)./2 + ((a.^6 .* mu1.^2)./4 -
(a.^6 .* mu2.^3)./27).^(1/2)).^(1/3))) .* 1i) ./ 2 ...
      - ((a.^3 .* mu1)./2 + ((a.^6 .* mu1.^2)./4 - (a.^6 .* mu2.^3)./27).^(1/2)).^(1/3) ./ 2
...
```

```
      - (a.^2 .* mu2) ./ (6 .* ((a.^3 .* mu1)./2 + ((a.^6 .* mu1.^2)./4 - (a.^6 .*
mu2.^3)./27).^(1/2)).^(1/3));

 end

 end
```
