# Supplementary material for: Dynamotypes for Dummies: A Toolbox, Atlas, and Tutorial for Simulating a Comprehensive Range of Realistic Synthetic Seizures
Source: eNeuro. 2025 Oct 15;12(10):ENEURO.0200-25.2025. doi: 10.1523/ENEURO.0200-25.2025 (PMC12549069; doi:10.1523/ENEURO.0200-25.2025)

Dynamotype Atlas

The following section shows examples of the 16 dynamotypes of seizures generated by the model and paths that were used to generate these simulations.

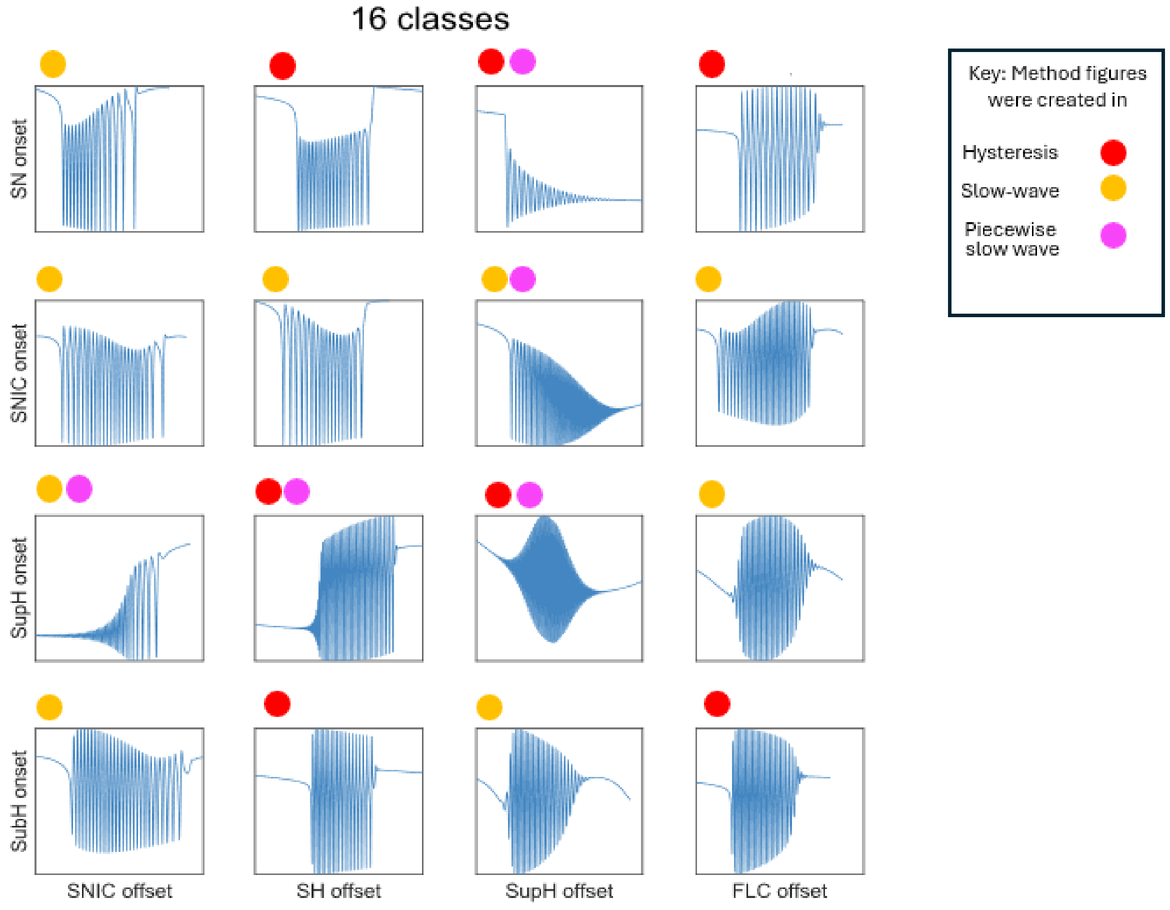

To generate simulated seizures, we traversed the bifurcation map using three methods. First, we made arc-shaped paths between an onset bifurcation curve and an offset bifurcation curve through a bistable region. In this case, slow variables are given feedback from the fast subsystem to drive the transition between states (Saggio 2017), to create a boomerang-like path with a hysteresis effect. We made direct paths along the arc between an onset and offset bifurcation through a bistable region. We varied the location of the onset and offset point to produce a range of potential pathways for each dynamotype. The 5 classes produced with this method are known as hysteresis bursters (Saggio 2017).

The second method for traversing the bifurcation map to generate simulated seizures is called slow-wave bursting. In this case, a circular path is created using one point on the onset curve, one point on the offset curve, and a third fixed point. There is no feedback from the fast subsystem; rather, the slow subsystem travels along the closed path at a self-sustained orbit

and constant speed (Saggio 2017). As before, we spaced points evenly along the entire length of each onset and offset curve and generated several possible paths for each dynamotype. The 8 classes produced with this method are known as slow-wave bursters (Saggio 2017).

The third method for traversing the map is a hybrid of the slow-wave bursting, which we introduce in this work. This method was necessary to create classes that did not traverse multiple bifurcation curves in a way that creates complex dynamics. In addition, this method allows a bursting pathway to begin and end in arbitrary locations in the parameter space, rather than returning to the original starting point. To do this, direct pathways between defined points are used to move through specific locations. Four arcs are created on the surface of the sphere to make a piecewise arc path using 4 points. The first point is a fixed point in the rest region. The second point is a point on the onset bifurcation curve. The third point is a randomized point in the limit cycle region. The fourth point is a point in the offset bifurcation curve. The arc paths are created from the rest point to first onset bifurcation point, first bifurcation point to limit cycle point, limit cycle point to second bifurcation point, and offset bifurcation point to the rest point, to create a continuous path. Next, to calculate the total time the path traversed, the path was scaled by the k variable and t step variable. Note that unlike the previous two methods that can continue to burst repeatedly if the simulation duration is long enough (hysteresis and slow wave bursting), this method only traverses the path one time during the simulation. The 5 classes produced with this method are called “piecewise” slow wave bursters.

In the following section, we provide an in-depth illustration of simulated seizures for all 16 classes relative to their path through parameter space. The diagram has a boxed area that zooms in on six points: three onset and three offset points. The onset points are in orange, the offset points are in blue, and each onset point is connected to an offset point by a gray path. The gray path represents the bursting path of the seizure. The diagram shows five paths for five seizures. For the seizure models, the three onset and offset points are connected in five different ways to show five different paths corresponding to the paths shown in the parameter space diagrams. Noise levels (low, medium, and high) are shown for each of the 5 seizures.

HYSTERESIS BURSTERS: SN/SH, SN/FLC, SubH/SH, SubH/FLC,

There are multiple pathways that generate SN/SH (saddle node (SN) onset bifurcation and a saddle homoclinic (SH) offset bifurcation) dynamotypes, each of which has a distinct dynamical profile due to the effects of DC shifts. We here present an example of all three.

SN/SH Dynamotype, example 1

The first example of SN/SH seizures was previously modeled as the Epileptor (Jirsa et al. 2014), which is one of the most prevalent dynamotypes in humans (Saggio et al. 2020). In the *x* time series, there is a DC shift that begins when the seizure starts and ends when the seizure stops. Spikes also slow logarithmically in frequency at seizure offset. High noise is able to move the system back and forth across the bistability independent of slow variable, causing frequent short bursts that stutter on and off.

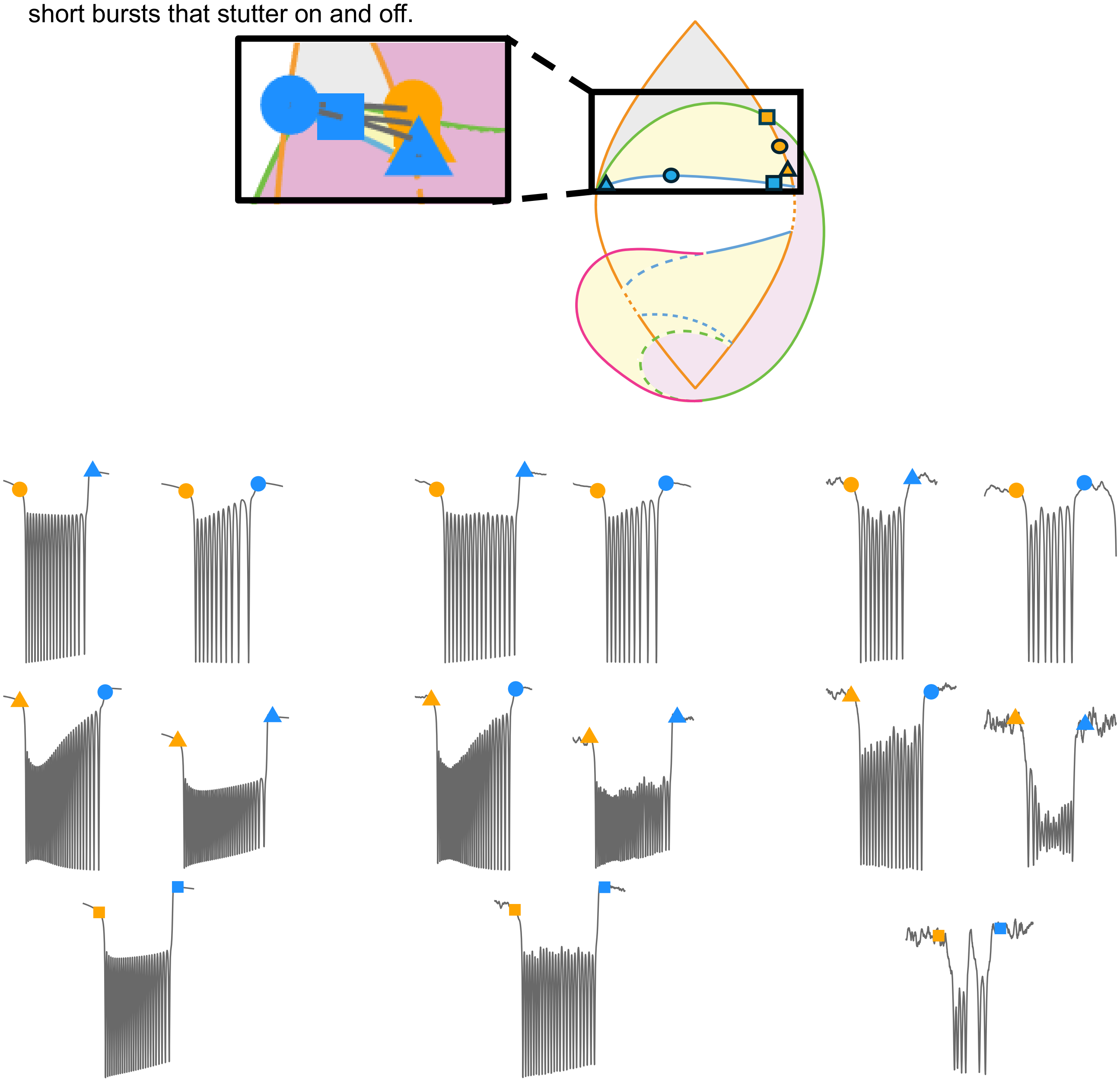

SN/SH Dynamotype, example 2

SN/SH seizures have a saddle node (SN) onset bifurcation and a saddle homoclinic (SH) offset bifurcation. In the *x* time series, this appears as a DC shift that begins when the seizure starts and ends when the seizure stops. Spikes also slow, logarithmically, in frequency at seizure offset. The bursting in this example does not start until the system crosses the SupH bifurcation. Mathematically, this would indicate that the SupH is the onset bifurcation, as proposed in (Saggio et al. 2017). However, as described in the main text, our convention is that the prior DC shift from first crossing the SN bifurcation means this will be classified as a SN onset. This trajectory was used to generate the seizure in Fig. 8 of the main text, which is very similar to some human seizures we identified. The examples in the far right are stuttering burst due to high noise as in the previous example. In this case, only the first short bursts of the stuttering are shown.

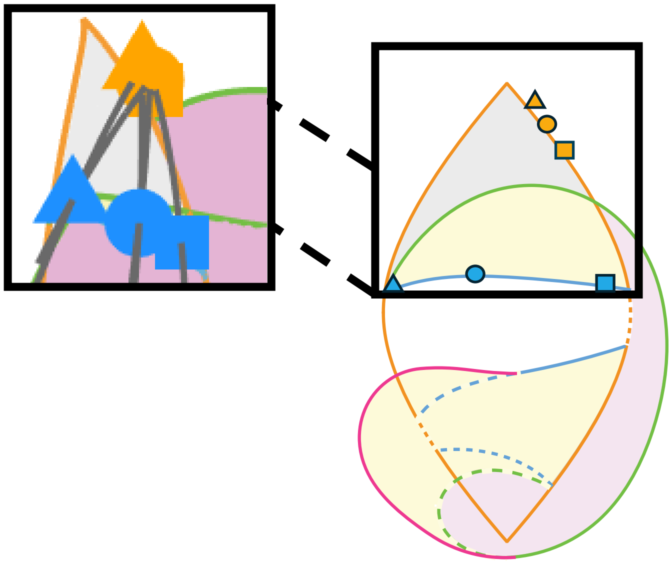

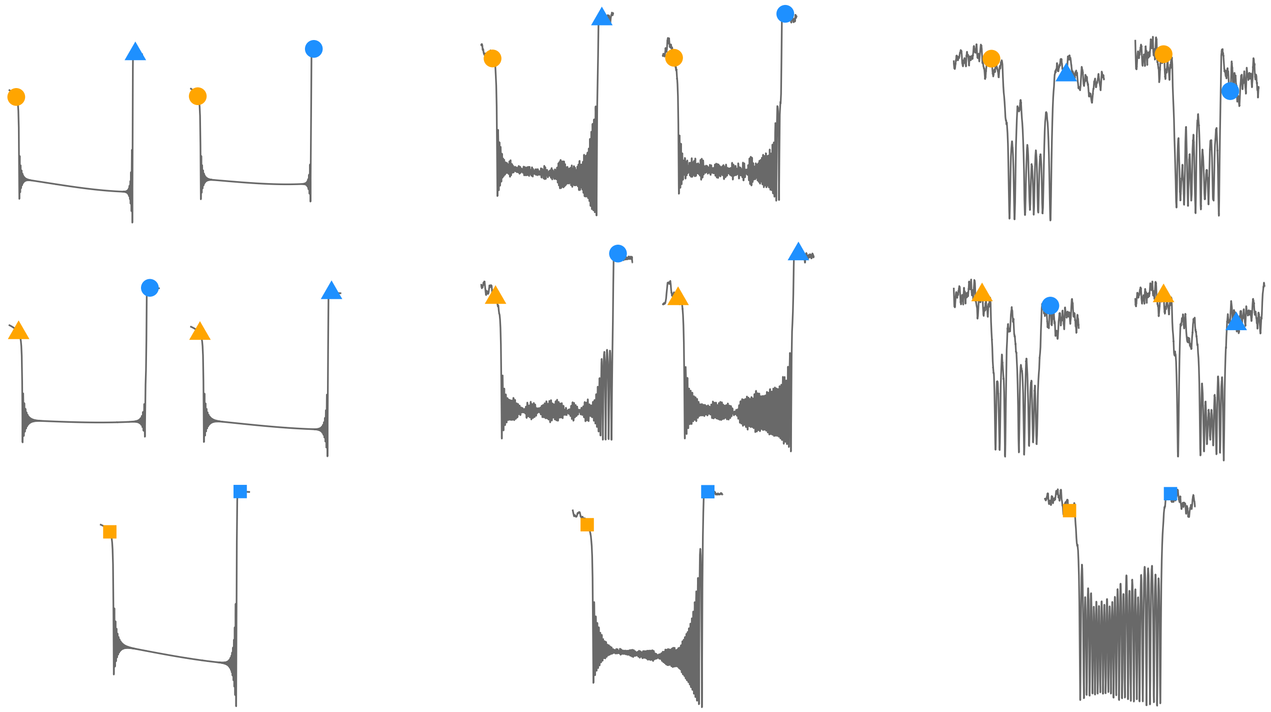

SN/SH Dynamotype, example 3

The third example is generated when the system is located in the lower region of the parameter space diagram (Figure 3). In the upper region, the stable fixed point lies to the right of the limit cycle, such that entering the limit cycle causes a baseline shift in the *x* time series. In the lower region, the stable fixed point lies inside the limit cycle, so there is no baseline shift in the *x* time series. In other words, there is no DC shift during the seizure. At seizure offset, the spiking rate slows, logarithmically in frequency.

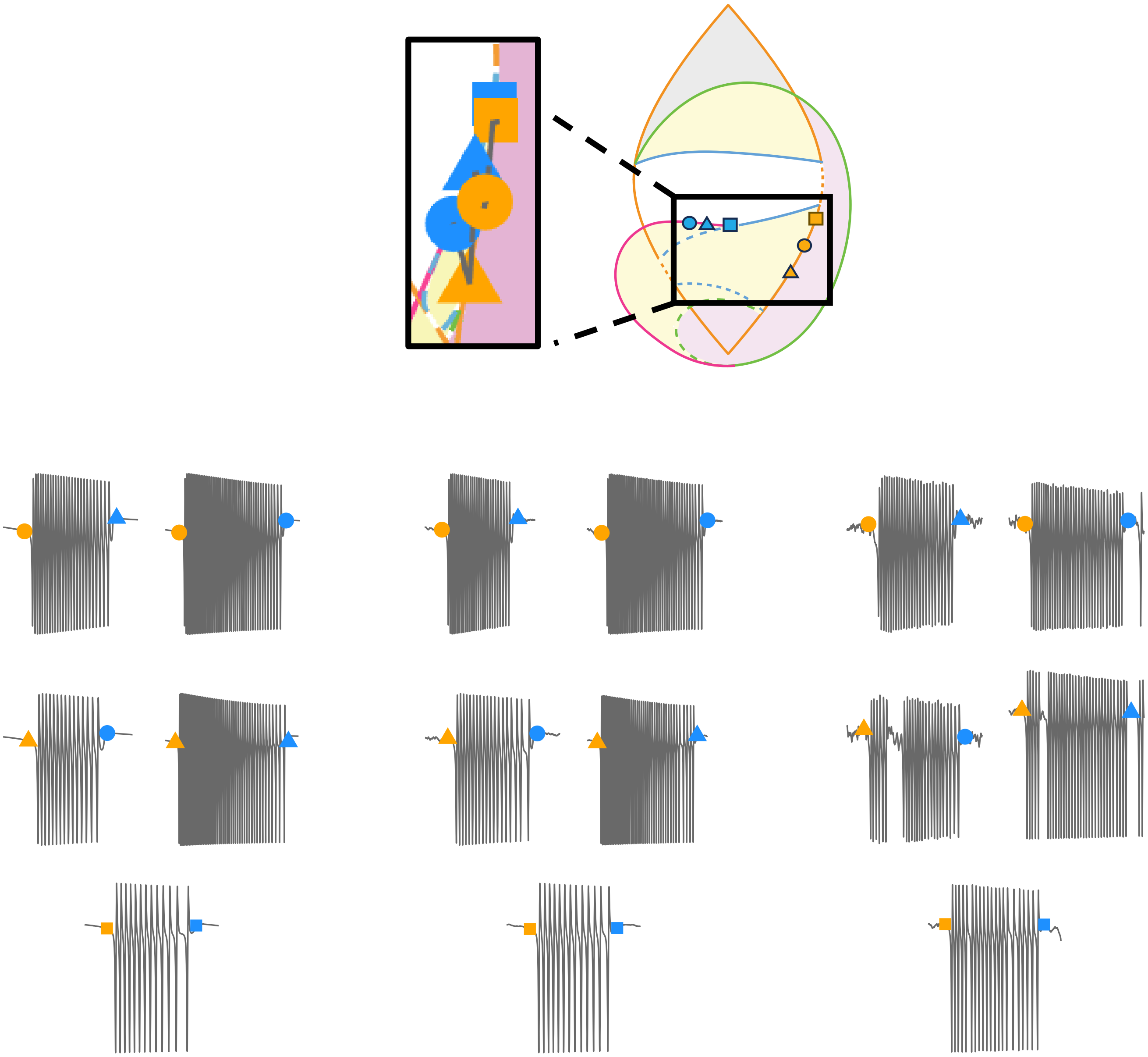

SN/SupH Dynamotype, example 1

SN/SupH seizures have a saddle node (SN) onset bifurcation and a supercritical Hopf (SupH) offset bifurcation. In the *x* time series, there is a DC shift at seizure onset and decreasing amplitude at seizure offset.

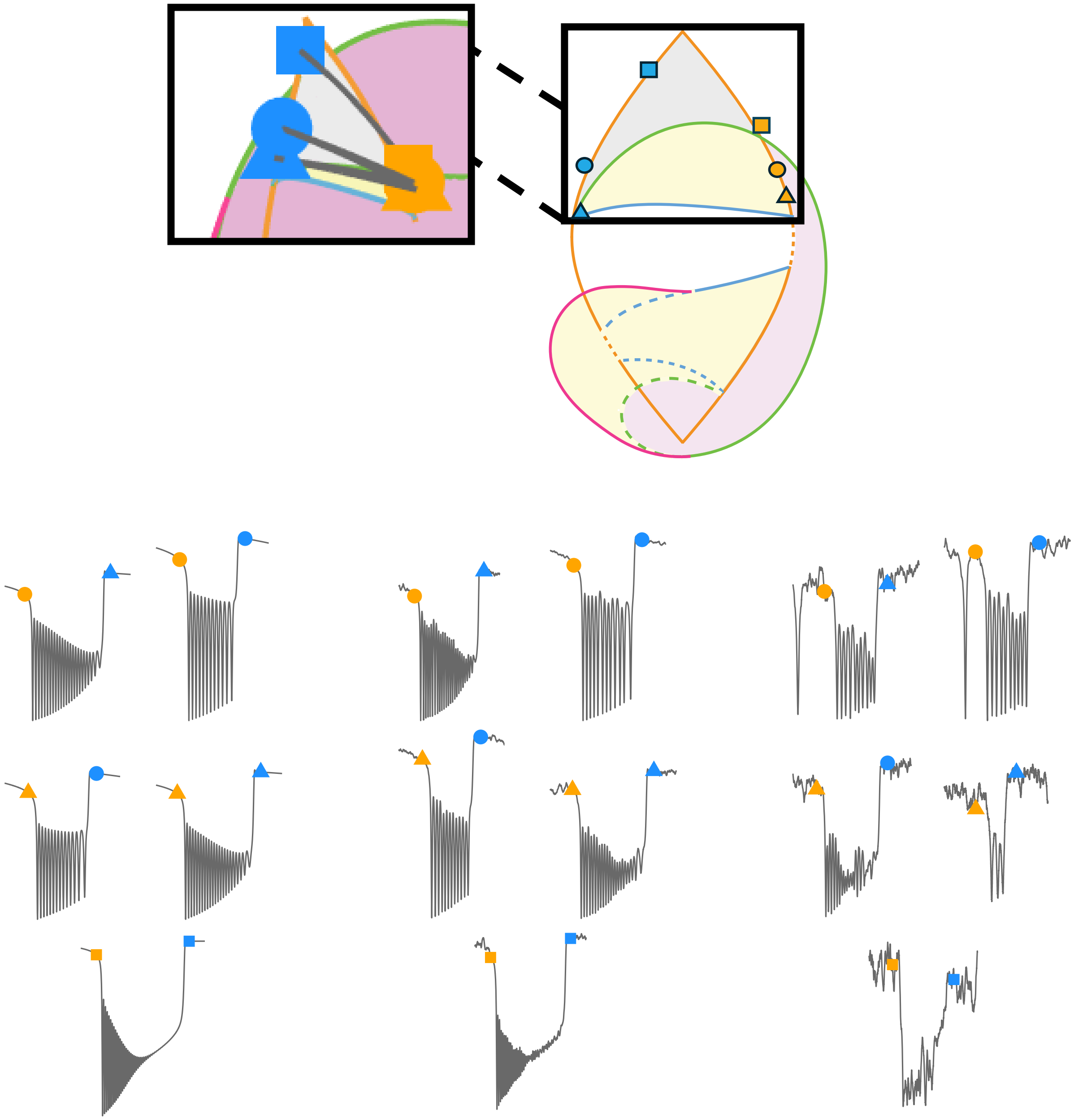

SN/SupH Dynamotype, example 2

SN/SupH seizures have a saddle node (SN) onset bifurcation and a supercritical Hopf (SupH) offset bifurcation. In the *x* time series, there is a DC shift at seizure onset and decreasing amplitude at seizure offset. Similar to the SN/SH example 2, bursting does not begin until a second bifurcation crossing at the SupH bifurcation, but since the system first must cross the SN bifurcation to have a DC shift, our convention is that this will be classified as a SN onset.

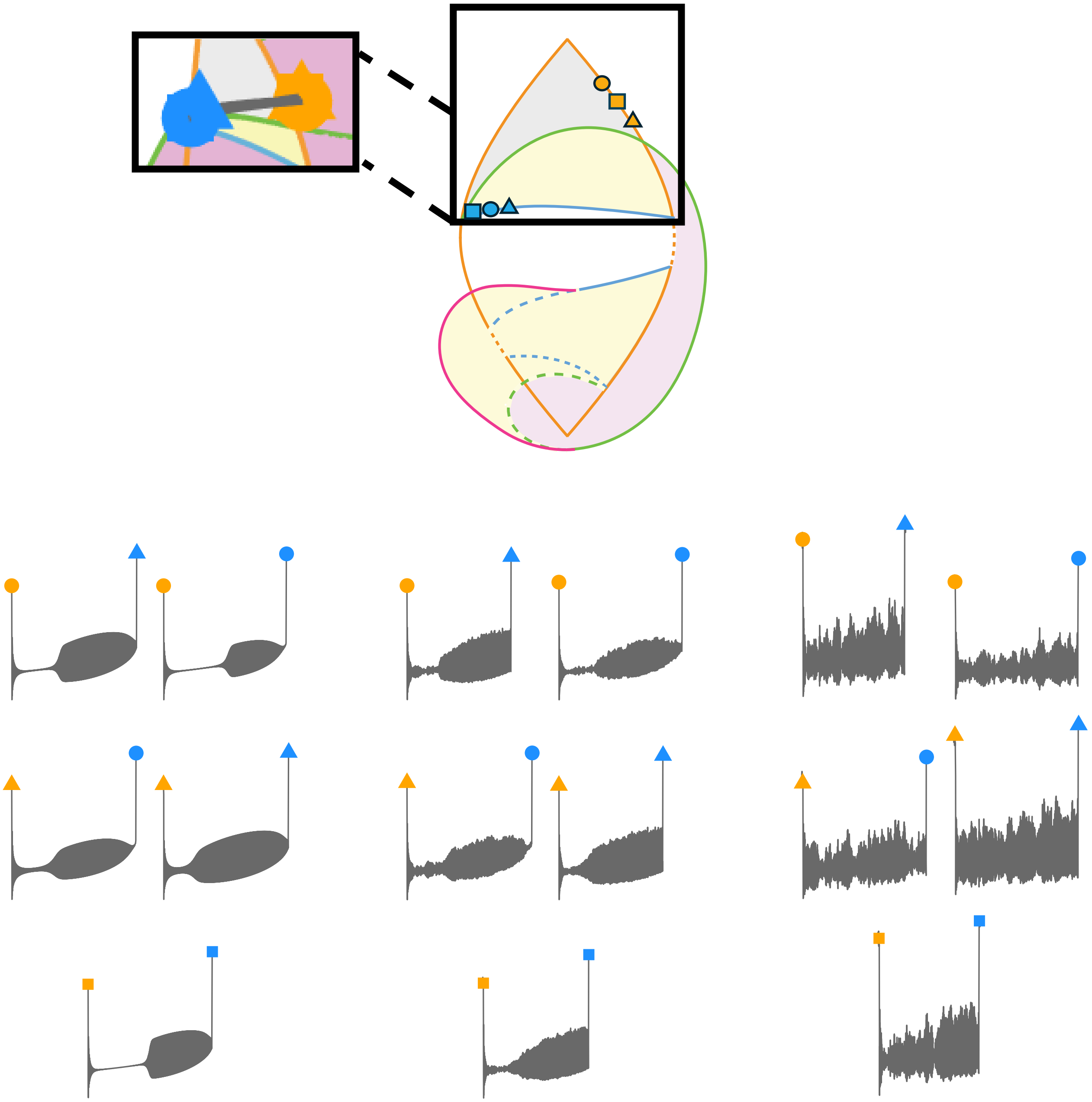

SN/FLC Dynamotype

SN/FLC seizures have a saddle node (SN) onset bifurcation and a fold limit cycle (FLC) offset bifurcation. Like SN/SH seizures, the path is located in the lower region of the parameter space diagram, such that no DC shift occurs at seizure onset. The *x* time series does not exhibit any distinguishing features for the FLC offset (i.e., no amplitude scaling, no frequency scaling, no baseline shift). Therefore, these seizures have generally arbitrary onset and offset dynamics.

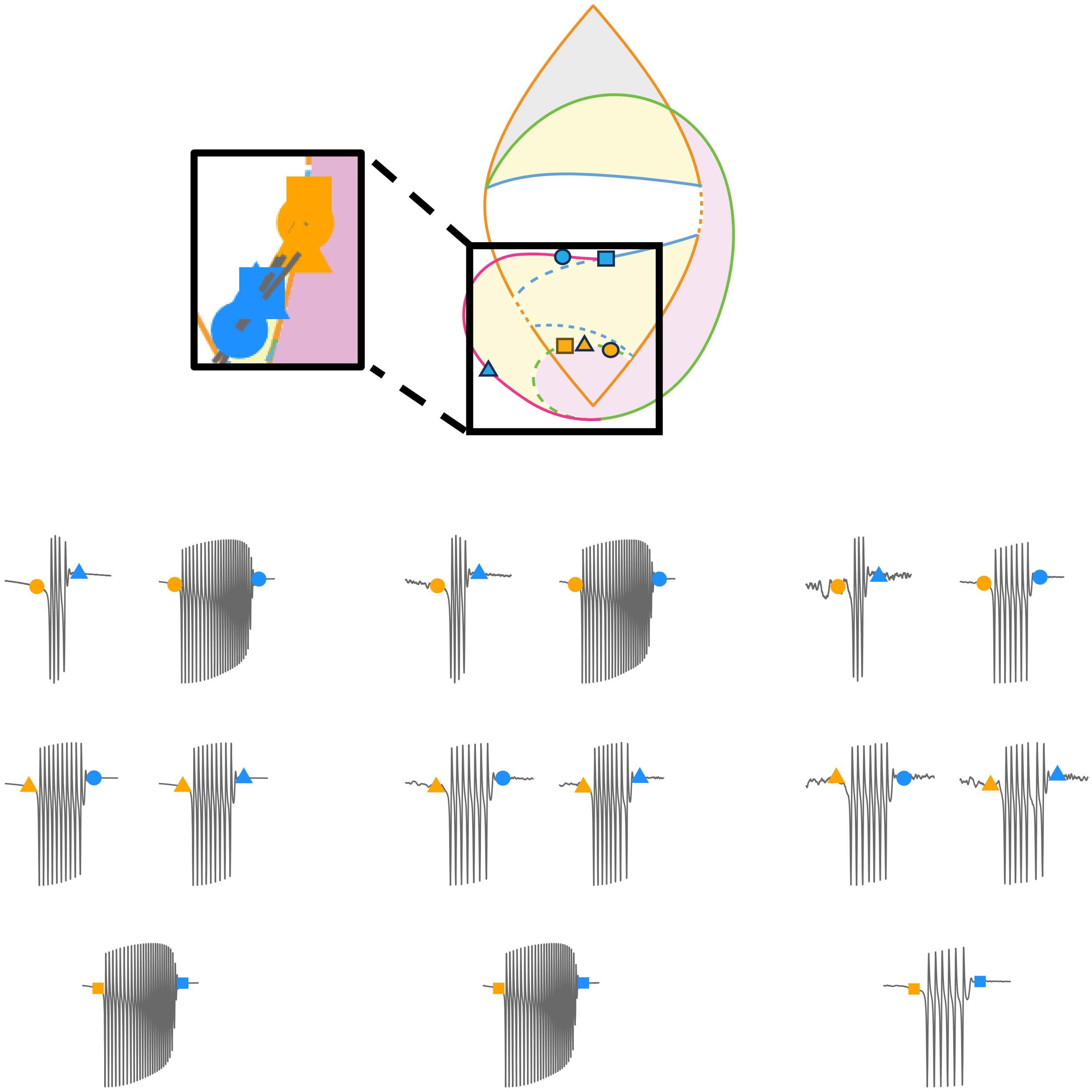

SubH/SH Dynamotype

SubH/SH seizures have a subcritical Hopf (SubH) onset bifurcation and a saddle homoclinic (SH) offset bifurcation. A SubH onset bifurcation has arbitrary dynamics, with no specific scaling rule for amplitude or frequency. Since the path is located in the lower region of the parameter space diagram, no DC shift occurs at seizure offset, but the spikes exhibit frequency slowing logarithmically. As in prior examples, high levels of noise cause stuttering that appears as short bursts of spikes (bottom right).

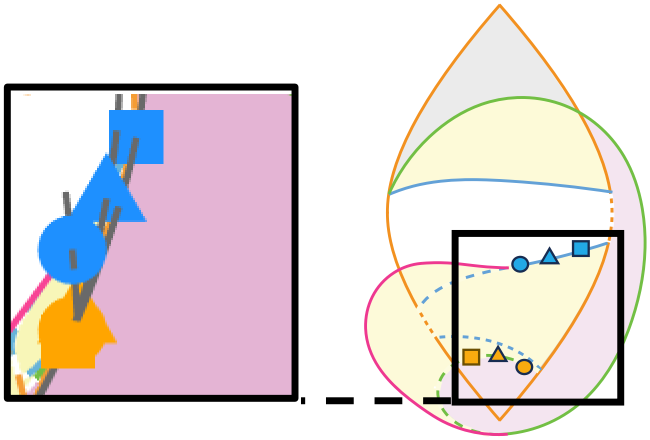

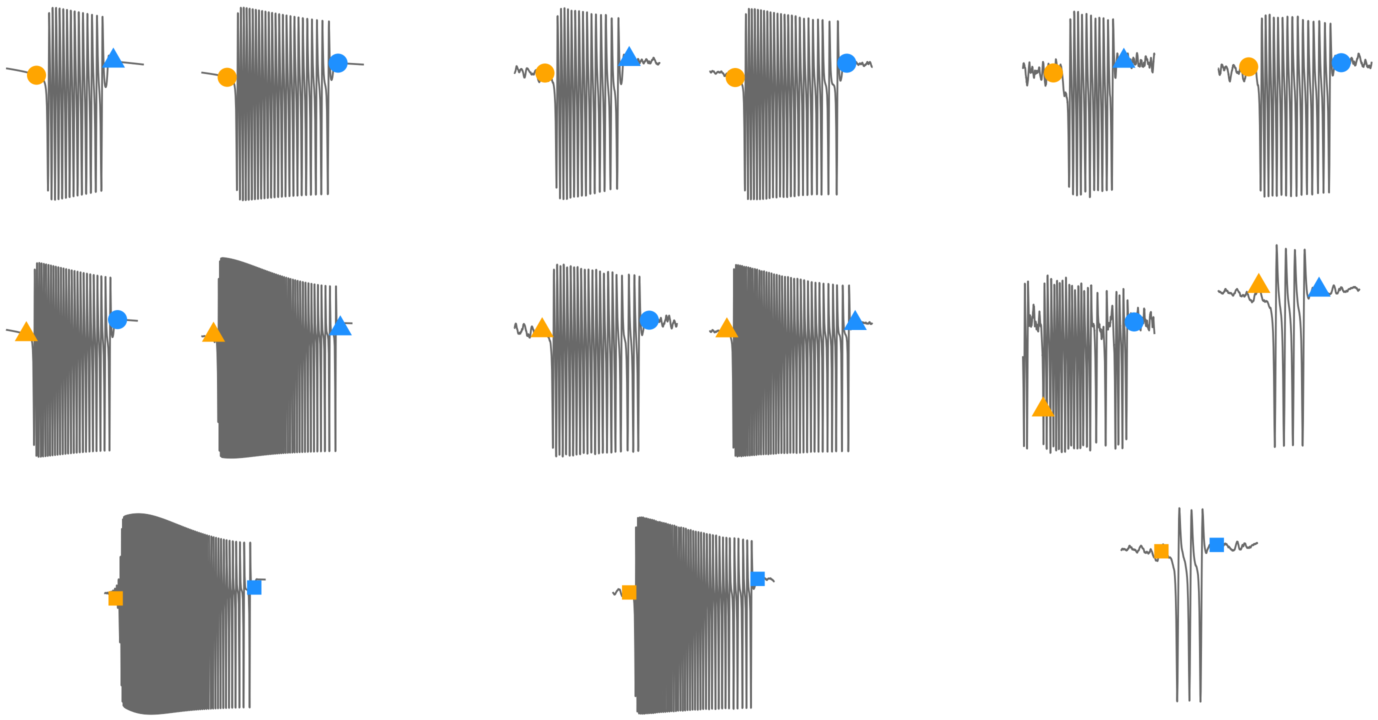

SubH/FLC Dynamotype

SubH/FLC seizures have a Subcritical Hopf (SubH) onset bifurcation and a fold limit cycle (FLC) offset bifurcation. Neither of these bifurcations have any distinguishing features in the *x* time series (i.e., no amplitude scaling, no frequency scaling, no baseline shift). In other words, SubH/FLC seizures have generally arbitrary onset and offset dynamics. Simulated seizures for SubH/FLC and SN/FLC are therefore indistinguishable when observing the raw data.

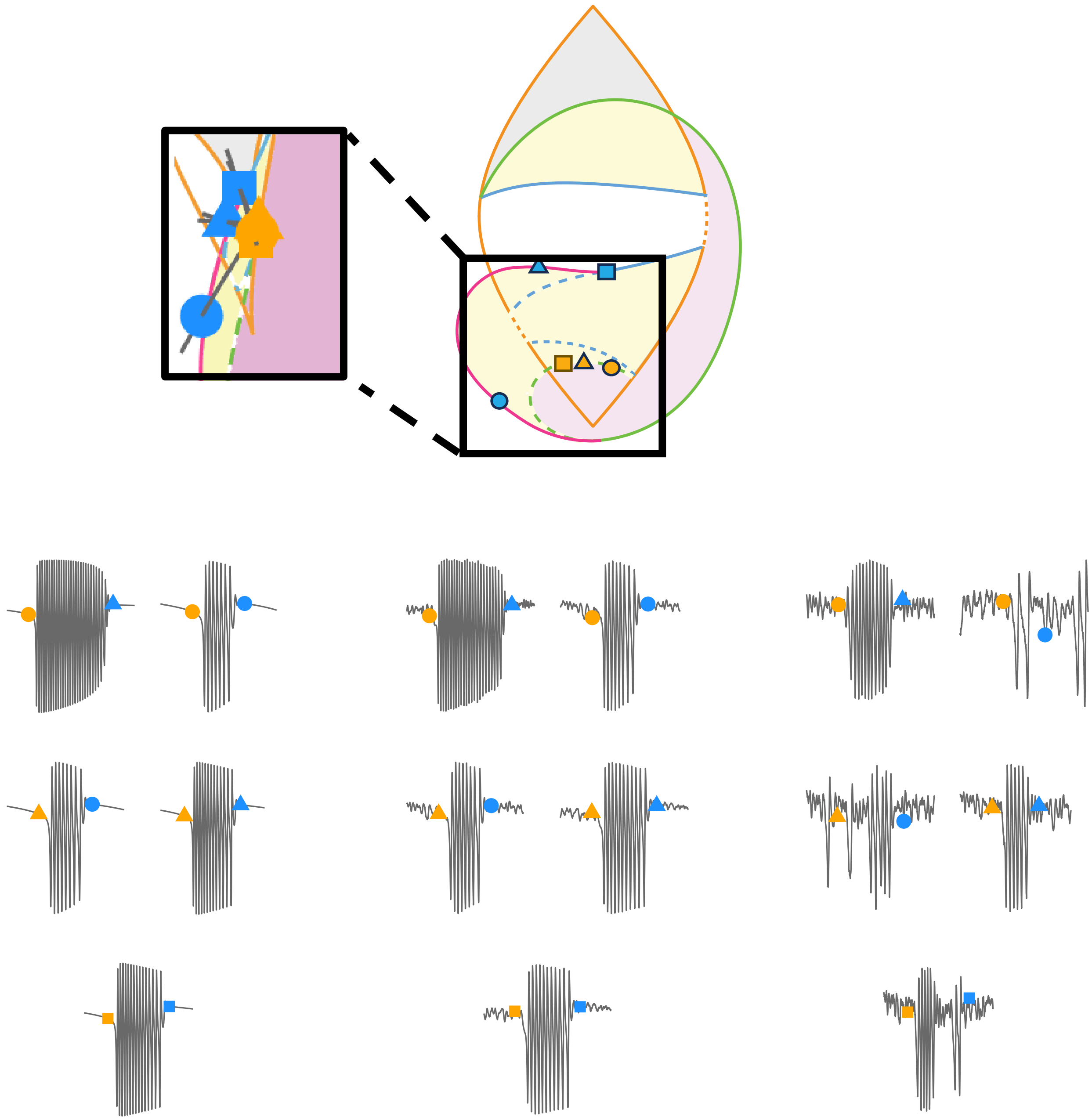

SLOW-WAVE BURSTERS: SN/SNIC, SNIC/SNIC, SNIC/SH, SNIC/SupH, SNIC/FLC, SupH/FLC, SubH/SNIC, SubH/SupH

SN/SNIC, example 1

SN/SNIC seizures have a saddle node (SN) onset and a saddle node invariant cycle (SNIC) offset. These seizures lie in the upper region of the parameter space diagram. As a result, the stable fixed point lies to the right of the limit cycle, such that entering the limit cycle causes a baseline shift in the *x* time series. This baseline shift is analogous to a DC shift at seizure onset. At offset, SNIC creates a logarithmic scaling of the frequency, with the spiking rate decreasing in frequency.

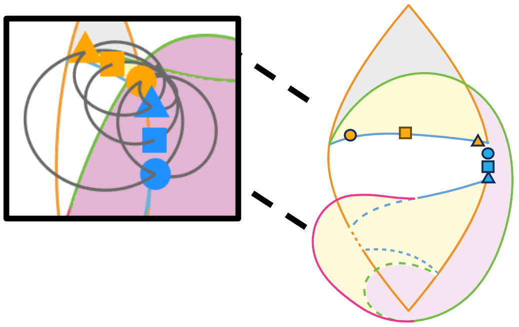

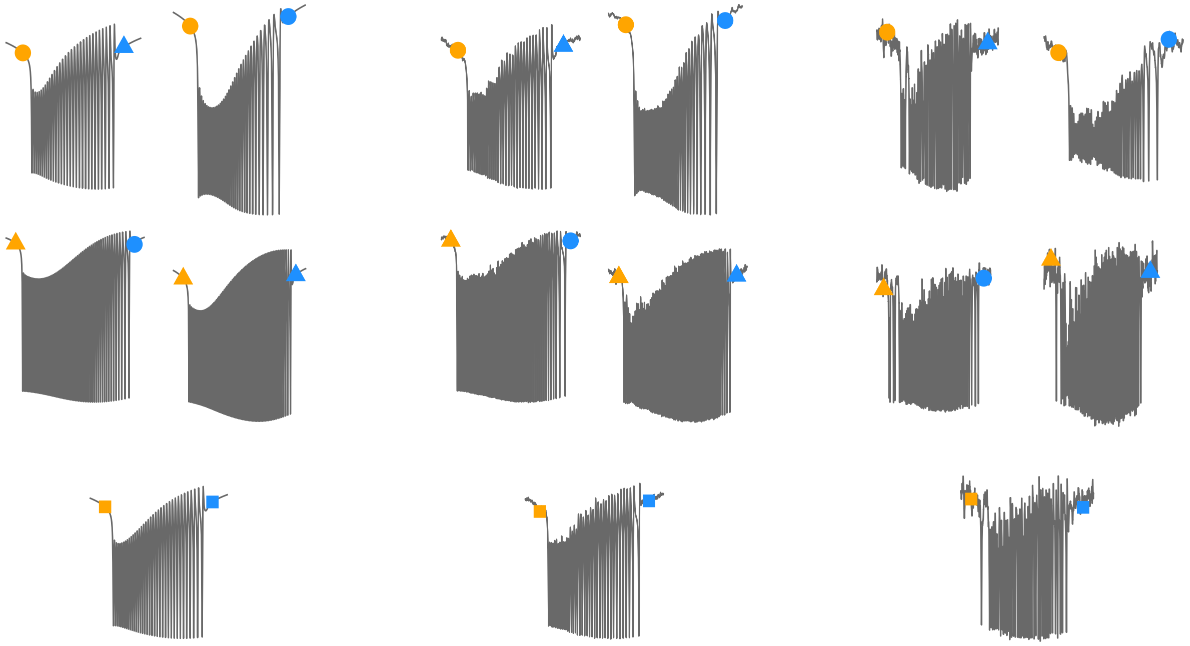

SN/SNIC, example 2

This onset is similar to example 1, except it traverses a SupH bifurcation after the DC-shift-inducing SN bifurcation. This is similar to SN/SH example 2 in the hysteresis bursters, where by convention we label the seizure by the DC shift at SN bifurcation, rather than the subsequent SupH bifurcation. Therefore, there is first a DC shift at onset. At seizure offset the saddle node invariant cycle (SNIC) onset creates a square root scaling of decreasing frequency. There is no DC shift at offset.

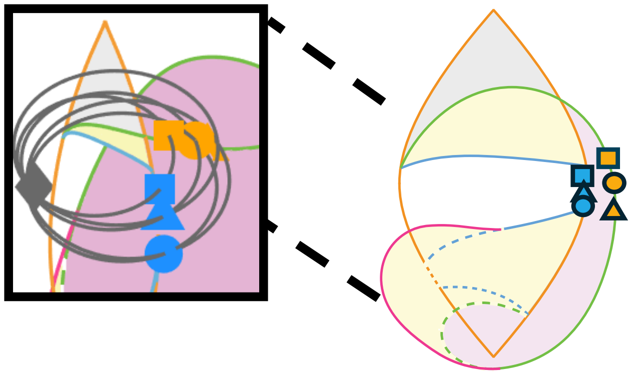

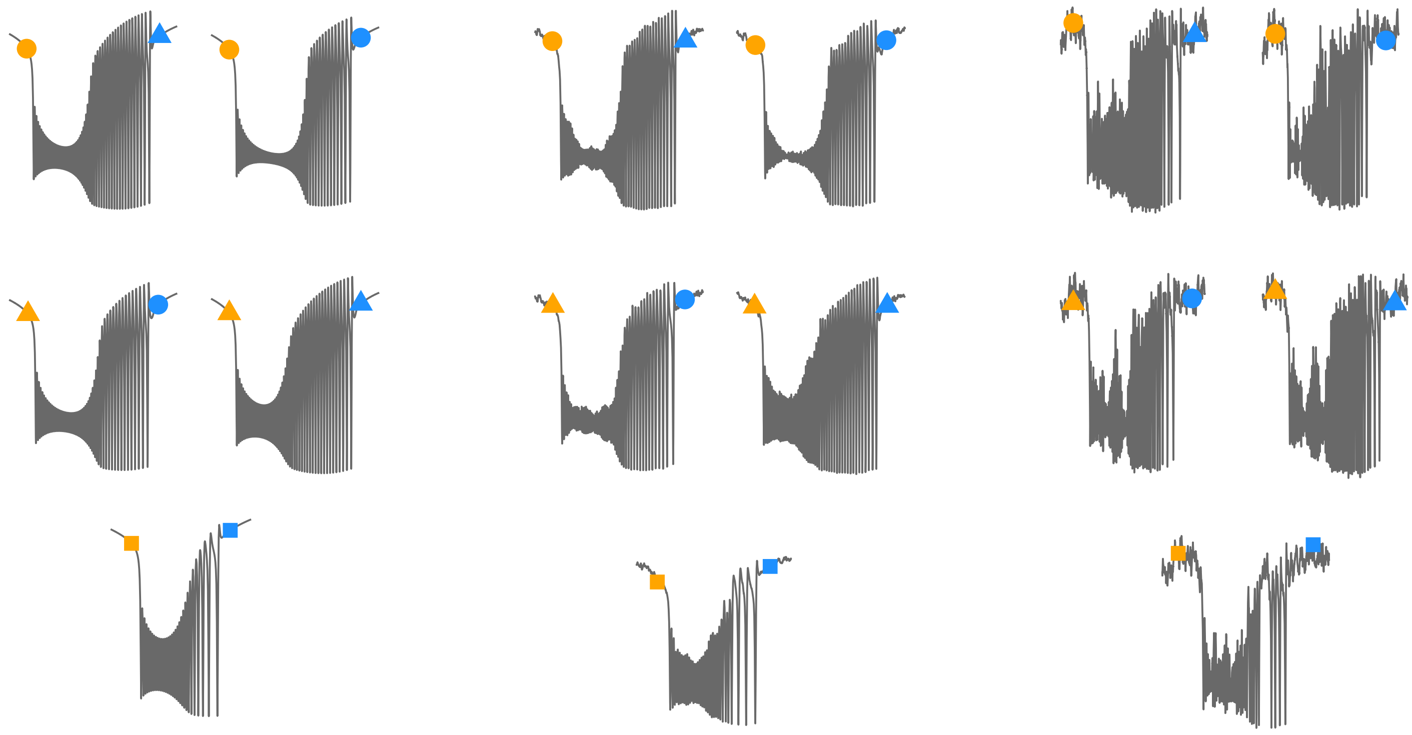

SNIC/SNIC Dynamotype

SNIC/SNIC seizures have a saddle node invariant cycle onset (SNIC) and a saddle node invariant cycle (SNIC) offset. In the *x* time series, the SNIC bifurcation for both onset and offset creates a square root scaling of the frequency. The spikes exhibit frequency increasing at onset and decreasing at offset. There is no DC shift occurring at onset or offset.

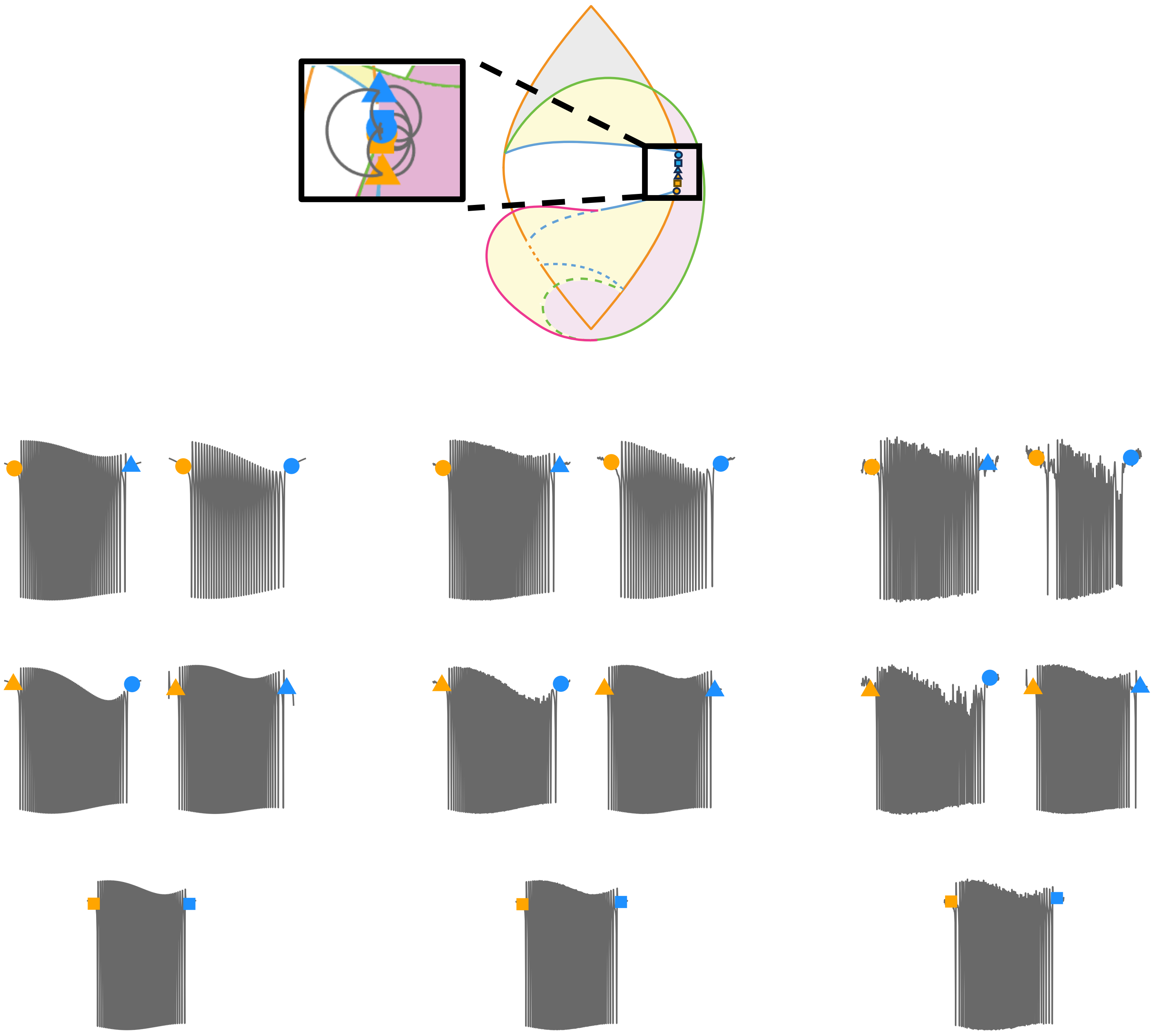

SNIC/SH Dynamotype

SNIC/SH seizures have a saddle node invariant cycle (SNIC) onset and a saddle homoclinic (SH) offset. In the *x* time series, the SNIC onset creates a square root scaling of the frequency and the SH offset creates a logarithmic scaling of the frequency. At seizure onset, no DC shift occurs and the spiking rate increases in frequency. At seizure offset, there is a DC shift and the spiking rate decreases in frequency.

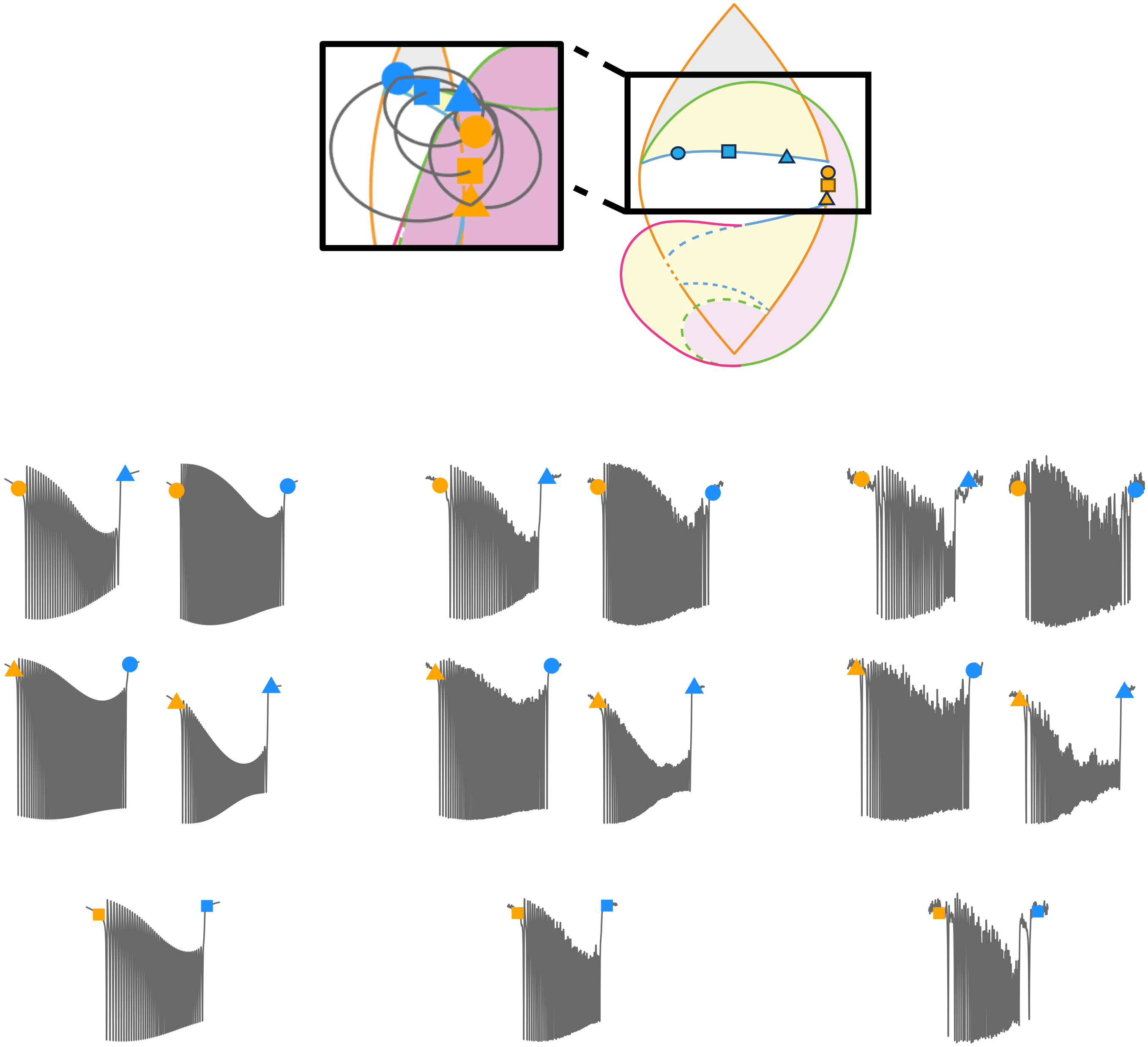

SNIC/SupH Dynamotype

SNIC/SupH seizures have a saddle node invariant cycle (SNIC) onset and a supercritical Hopf (SupH) offset. At seizure onset, in the *x* time series, the saddle node invariant cycle (SNIC) onset creates a square root scaling of increasing frequency. The SupH offset creates logarithmically decreasing amplitude. This trajectory induces a DC shift at seizure offset.

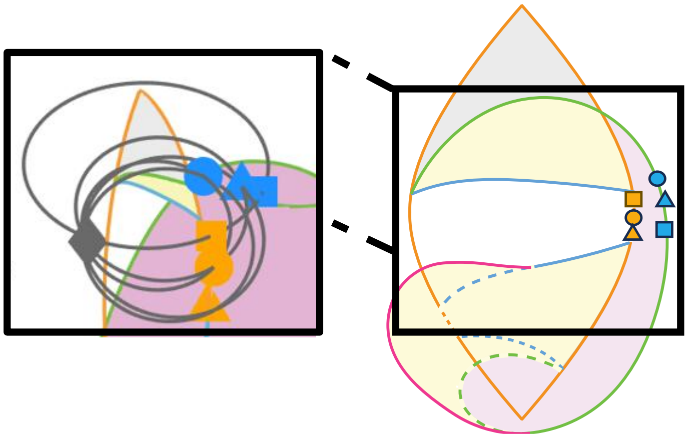

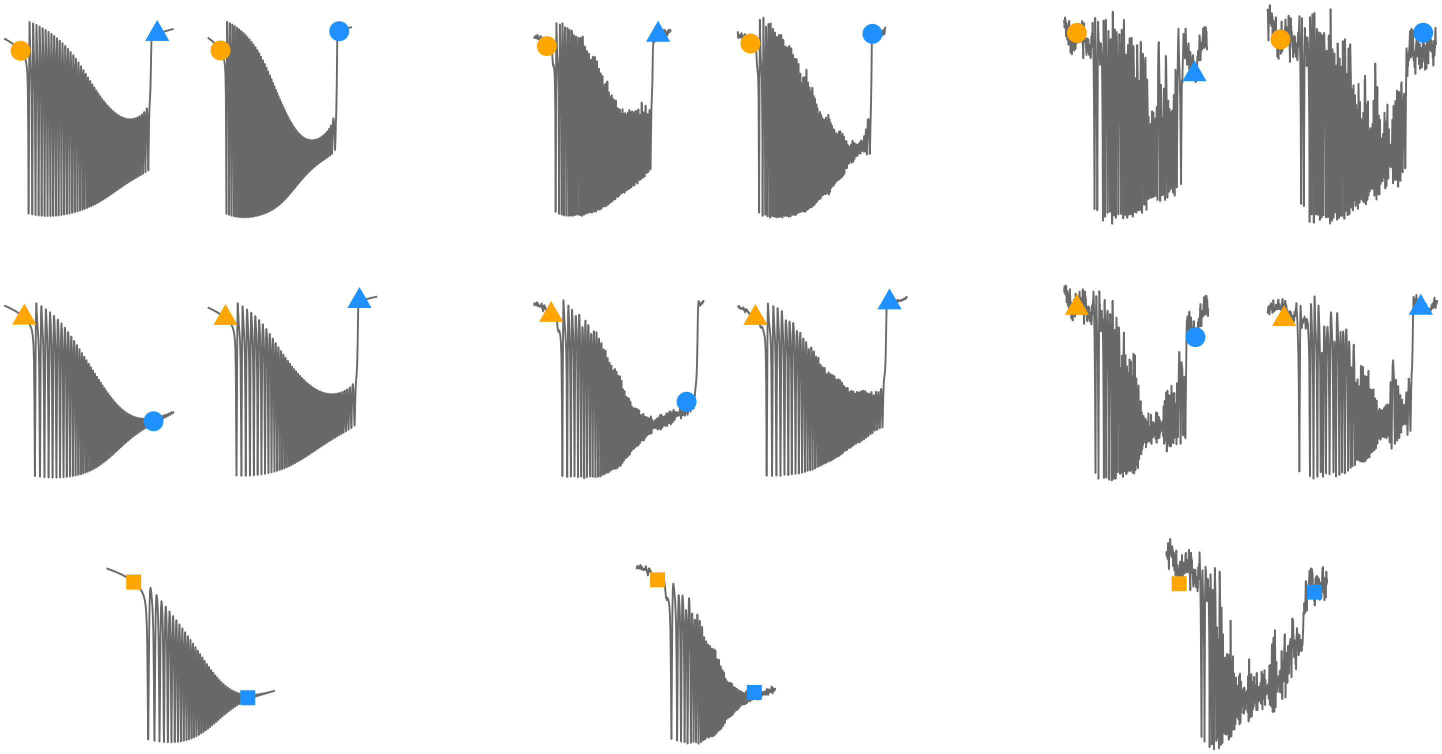

SNIC/FLC Dynamotype

SNIC/FLC seizures have a saddle node invariant cycle (SNIC) onset and a fold limit cycle (FLC) offset. In the *x* time series, the SNIC onset creates a square root scaling of the frequency. The spikes exhibit the frequency increasing during seizure onset. During seizure offset, the time series *x* does not exhibit any distinguishing features (i.e., no amplitude scaling, no frequency scaling, no baseline shift). There is no DC shift occurring at onset or offset.

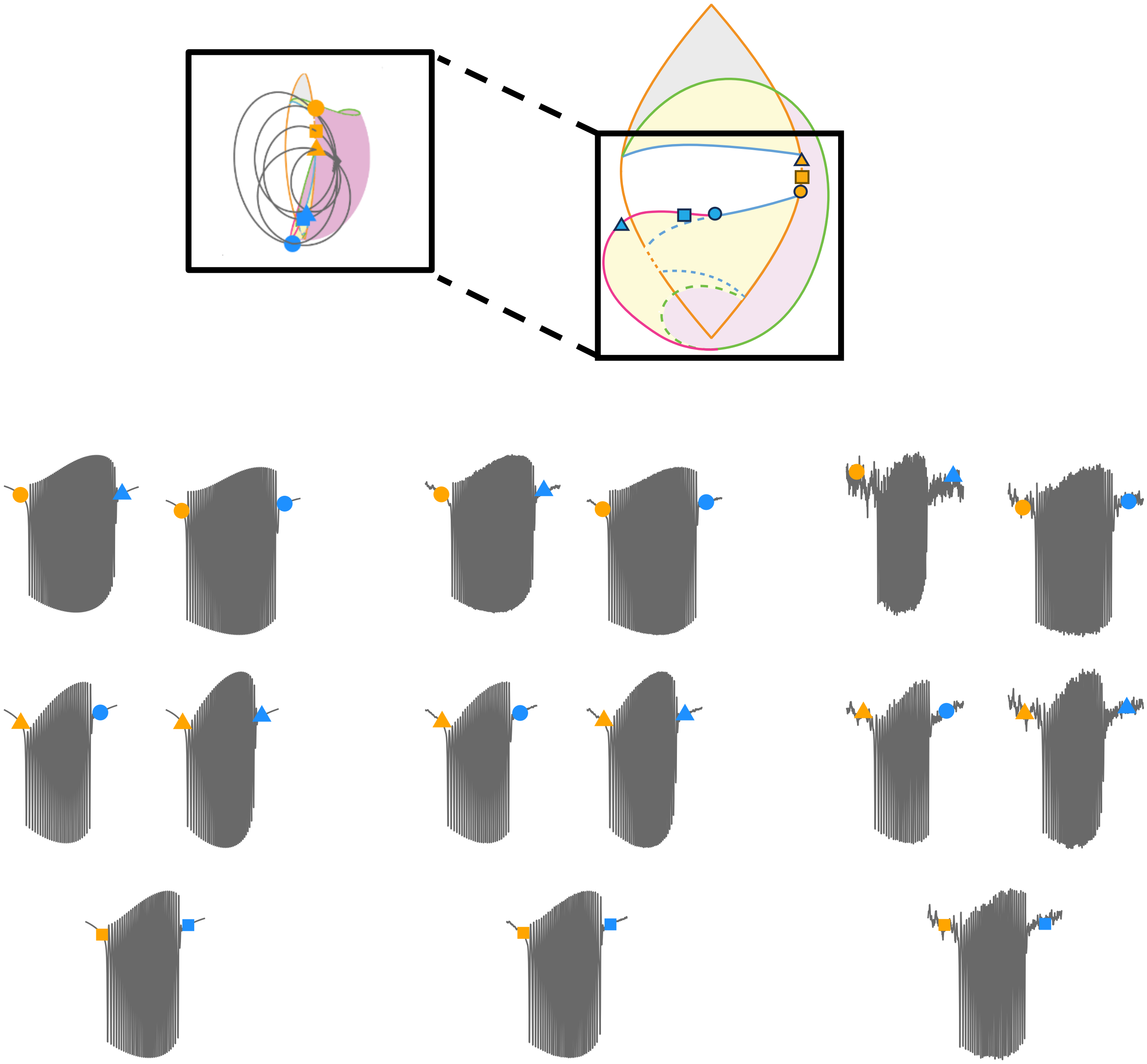

SupH/FLC Dynamotype

SupH/FLC seizures have a supercritical Hopf (SupH) cycle onset and a fold limit cycle (FLC) offset. In the time series *x*, the amplitude increases during seizure onset. During seizure offset, the time series *x* does not exhibit any distinguishing features (i.e., no amplitude scaling, no frequency scaling, no baseline shift). There is no DC shift occurring at onset or offset.

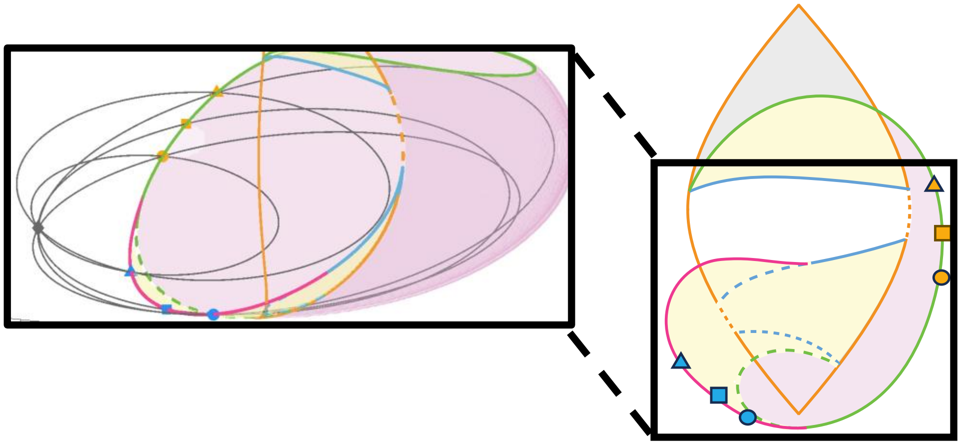

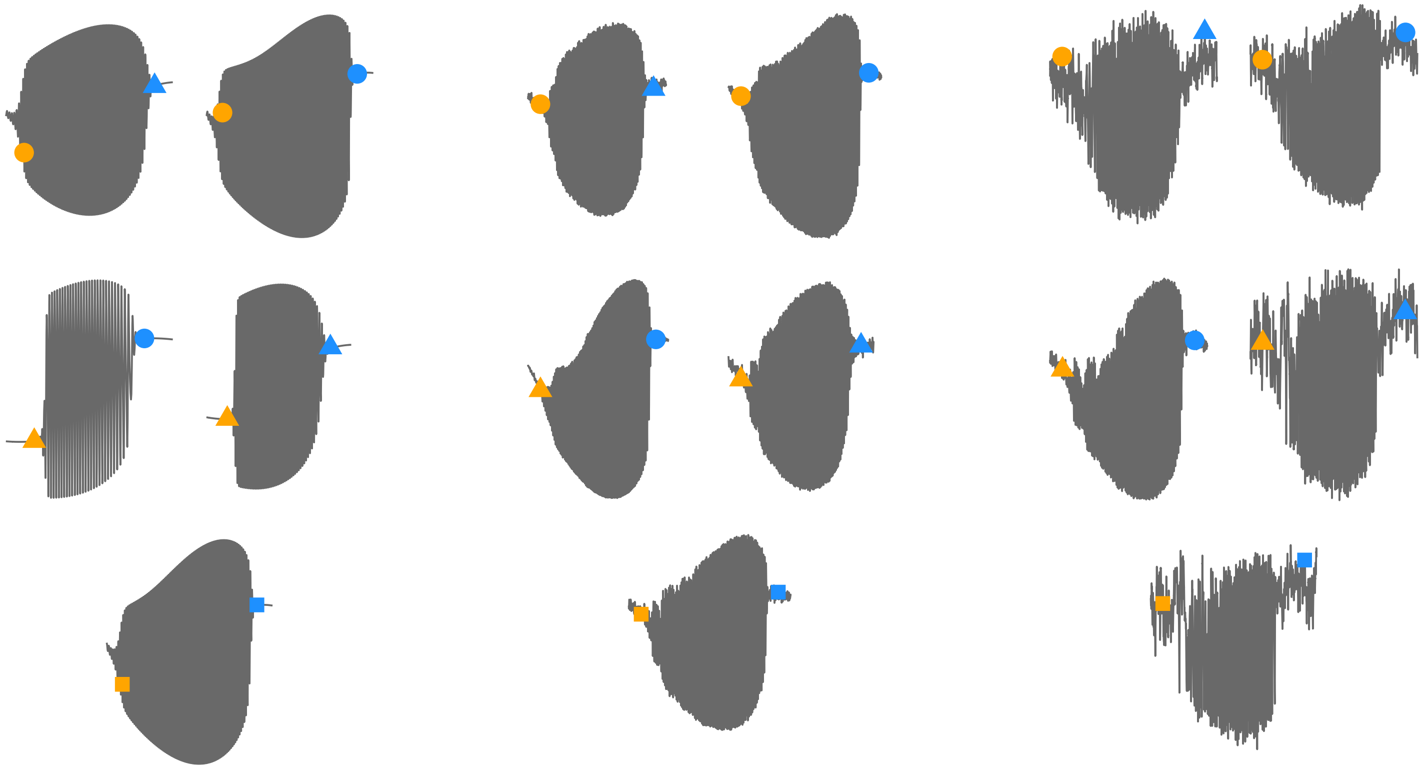

SubH/SNIC Dynamotype

SubH/SNIC seizures have a Subcritical Hopf (SubH) onset and a saddle node invariant cycle (SNIC) offset. During seizure onset, the time series *x* does not exhibit any distinguishing features (no amplitude scaling, no frequency scaling, no baseline shift). Also, in the *x* time series, the SNIC offset creates a square root scaling of the frequency. The spikes exhibit the frequency slowing during seizure offset. There is no DC shift occurring at onset or offset.

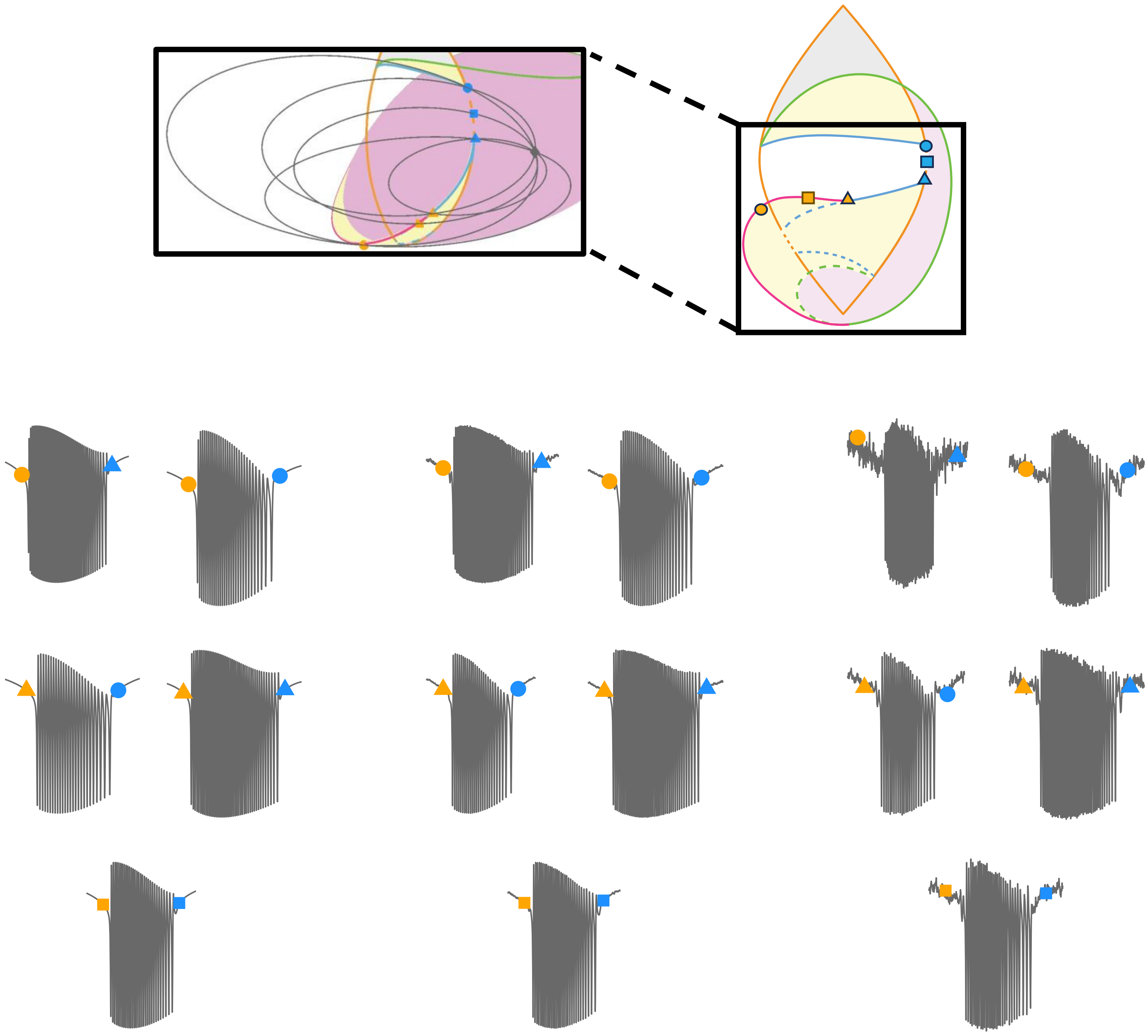

SubH/SupH Dynamotype

SubH/SupH seizures have a subcritical Hopf (SubH) onset and a supercritical Hopf (SupH) offset. During seizure onset, the time series *x* does not exhibit any distinguishing features(i.e., no amplitude scaling, no frequency scaling, no baseline shift). Also, in the time series *x*, the amplitude decreases during seizure offset. There is no DC shift occurring at onset or offset.

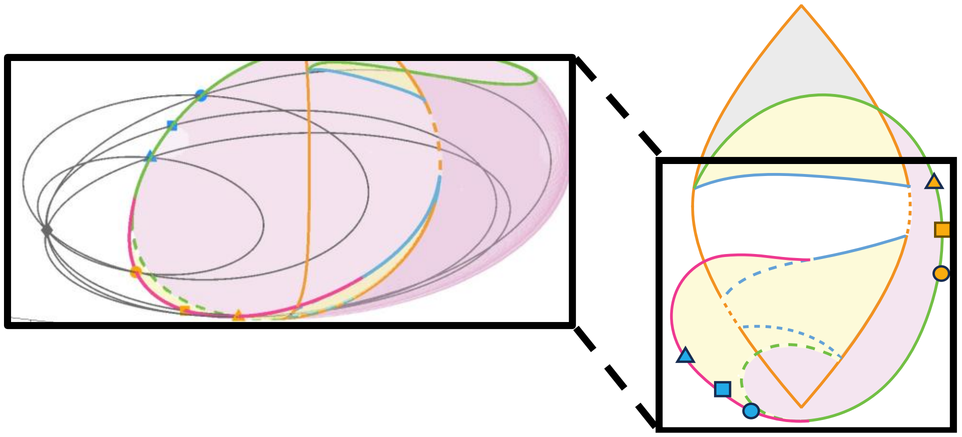

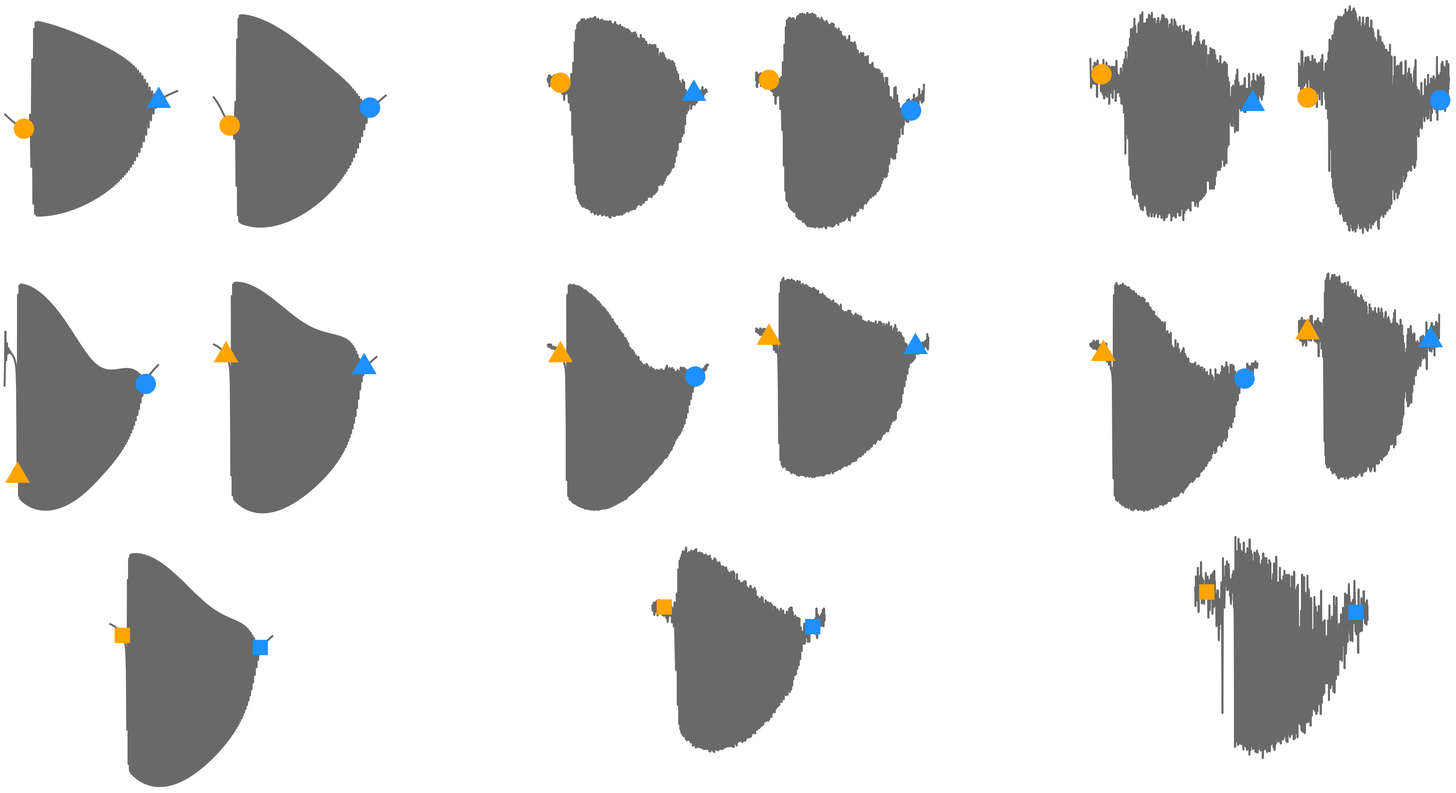

PIECEWISE SLOW-WAVE BURSTERS: SN/SupH, SNIC/SupH, SupH/SNIC, SupH/SH, SupH/SupH,

SN/SupH Dynamotype

SN/SupH seizures have a saddle node (SN) onset bifurcation and a supercritical Hopf (SupH) offset bifurcation. In the *x* time series, this appears as a DC shift that begins when the seizure starts and an amplitude decreasing to zero at the end of the seizure. Since the SupH offset curve is not adjacent to a rest region on the parameter space map, the bursting path does not return to the same baseline as at offset. As a result, there is a new baseline at seizure offset.

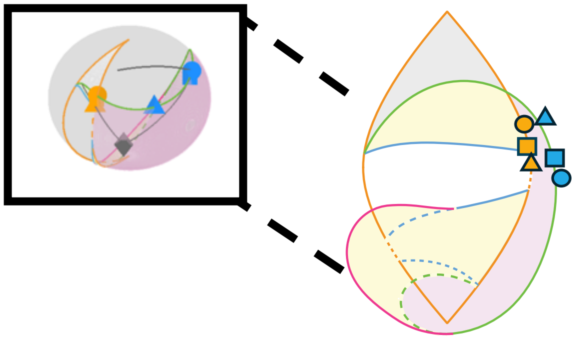

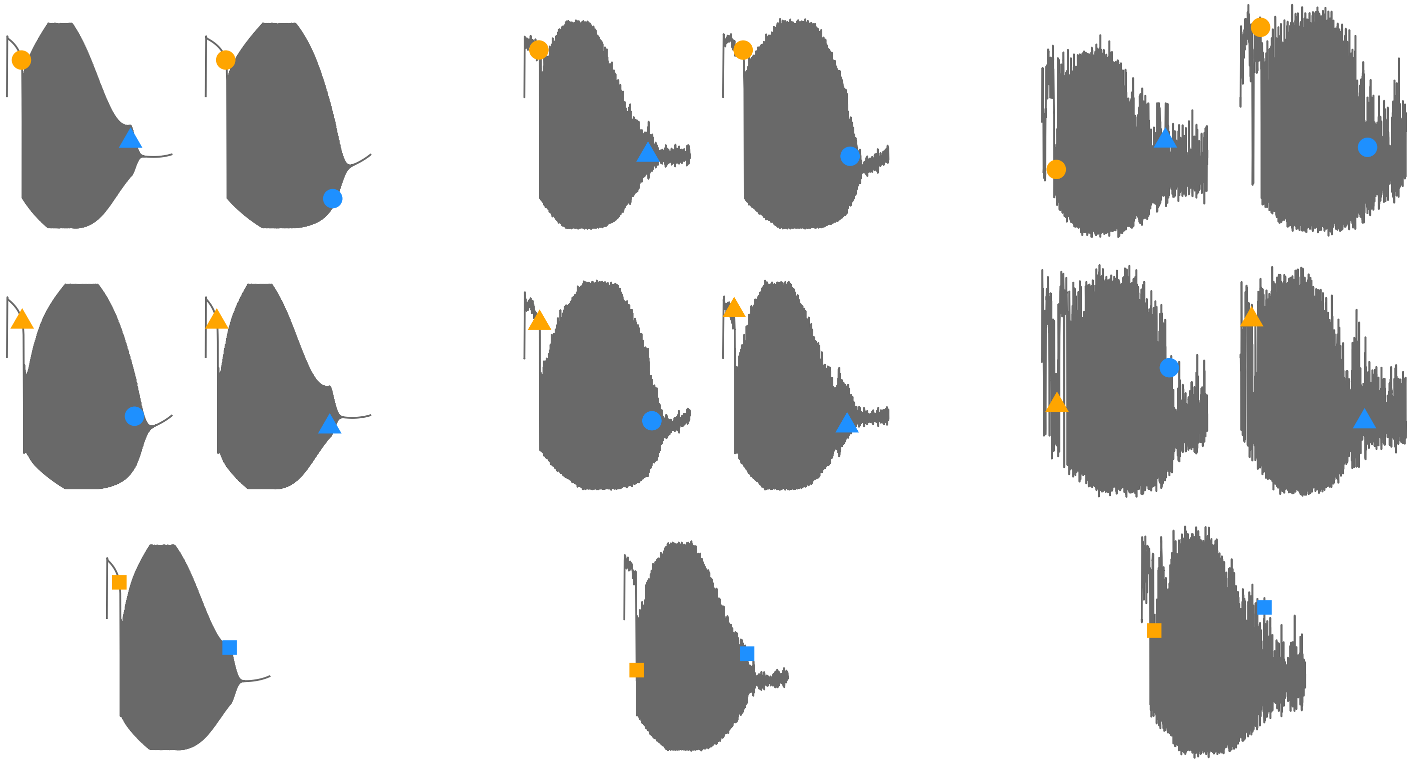

SNIC/SupH Dynamotype

SNIC/SupH seizures have a saddle node invariant cycle (SNIC) onset and a supercritical hopf (SupH) offset. At seizure onset, in the *x* time series, the saddle node invariant cycle (SNIC) onset creates a square root scaling of the frequency. The SH offset creates a logarithmic scaling of the frequency. There is no DC shift at onset. As in the previous SN/SupH piecewise example, the bursting path does not return to the same baseline at offset.

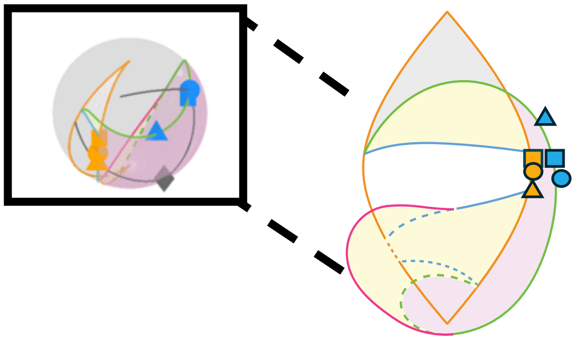

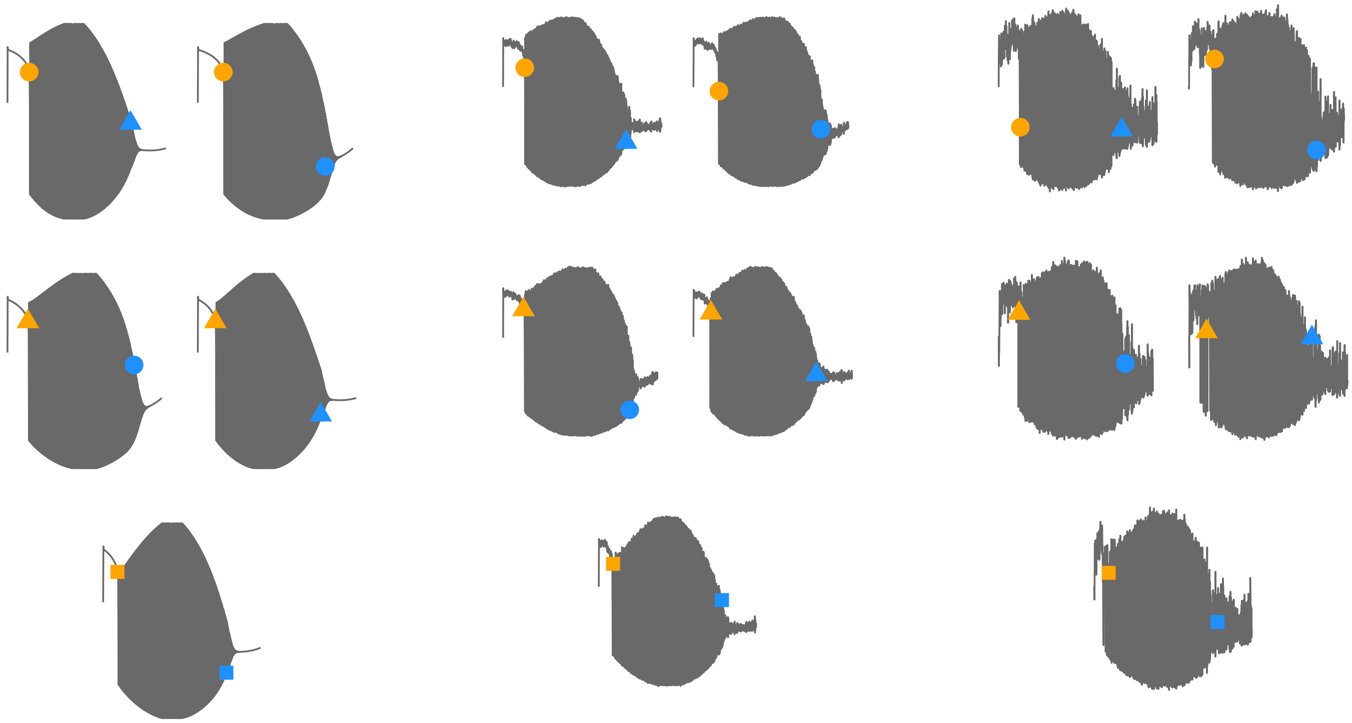

SupH/SNIC Dynamotype

SupH/SNIC seizure have a saddle node invariant cycle (SNIC) onset and a supercritical Hopf (SupH) offset. The SupH onset creates an increase in amplitude in the signal. At seizure offset, in the *x* time series, the saddle node invariant cycle (SNIC) onset creates a square root scaling of the frequency. There is no DC shift at onset, and there is a new baseline after seizure offset.

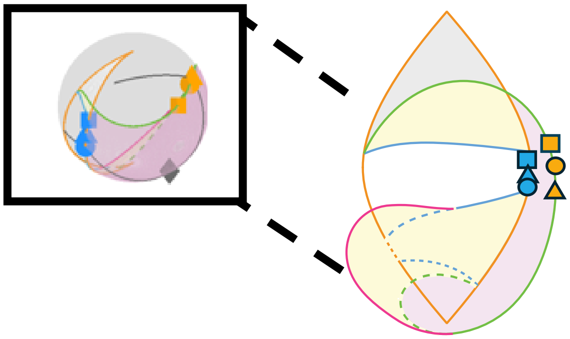

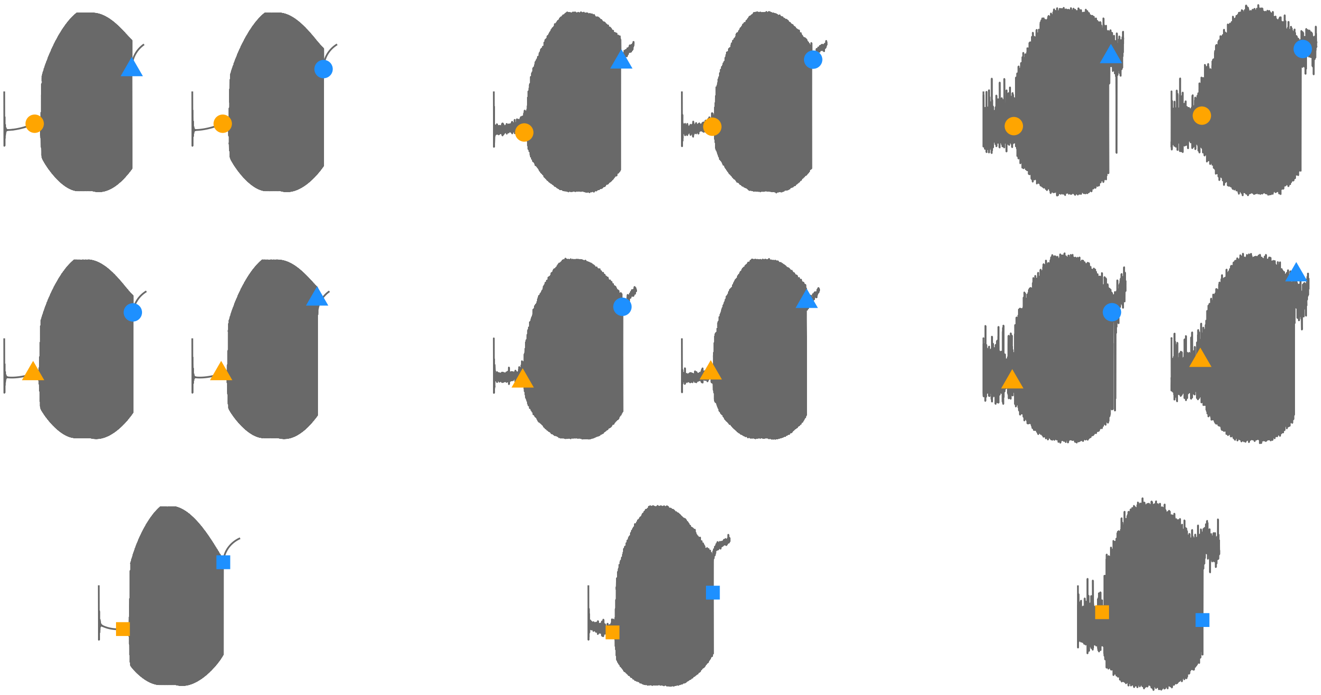

SupH/SH Dynamotype

SupH/SH seizures have a supercritical Hopf (SupH) onset bifurcation and a saddle homoclinic (SH) offset bifurcation. At seizure onset, in the *x* time series, there is an increase in amplitude. There is no DC shift at seizure onset. Also, the SH offset creates a logarithmic scaling of the frequency and there is a DC shift at seizure offset.

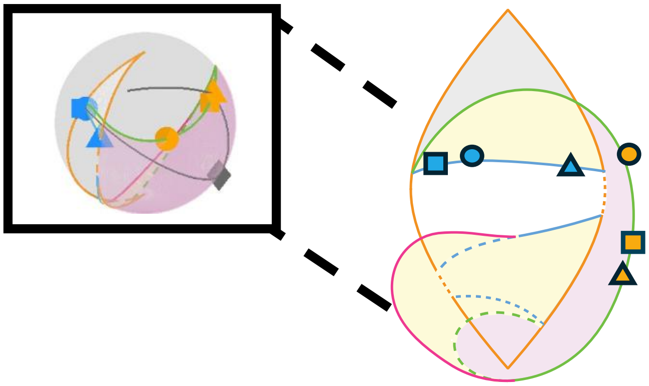

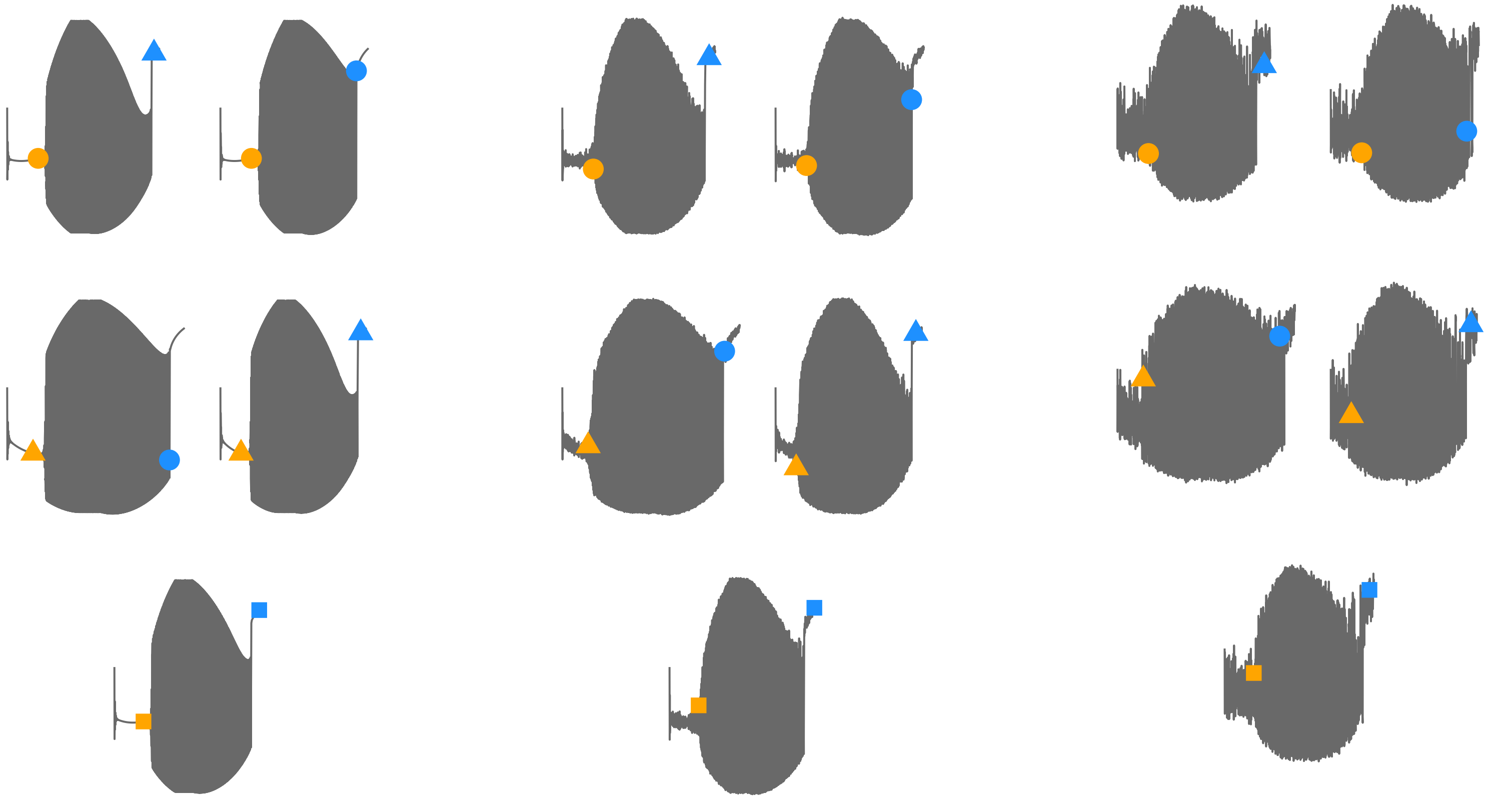

SupH/SupH Dynamotype

SupH/SupH seizures have a supercritical Hopf (SupH) onset bifurcation and a supercritical Hopf (SupH) offset bifurcation. At seizure onset, in the *x* time series, there is an increase of amplitude and an amplitude decreasing to zero at seizure offset. There is no DC shift occurring at onset or offset.

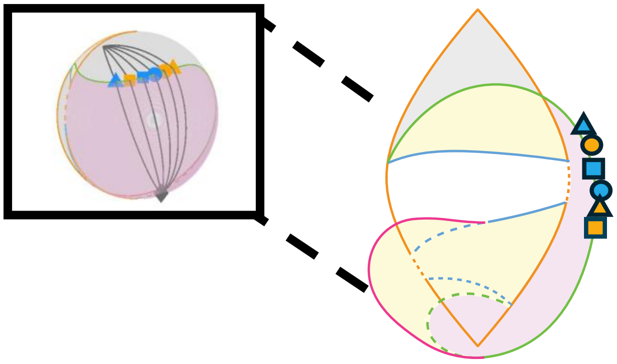

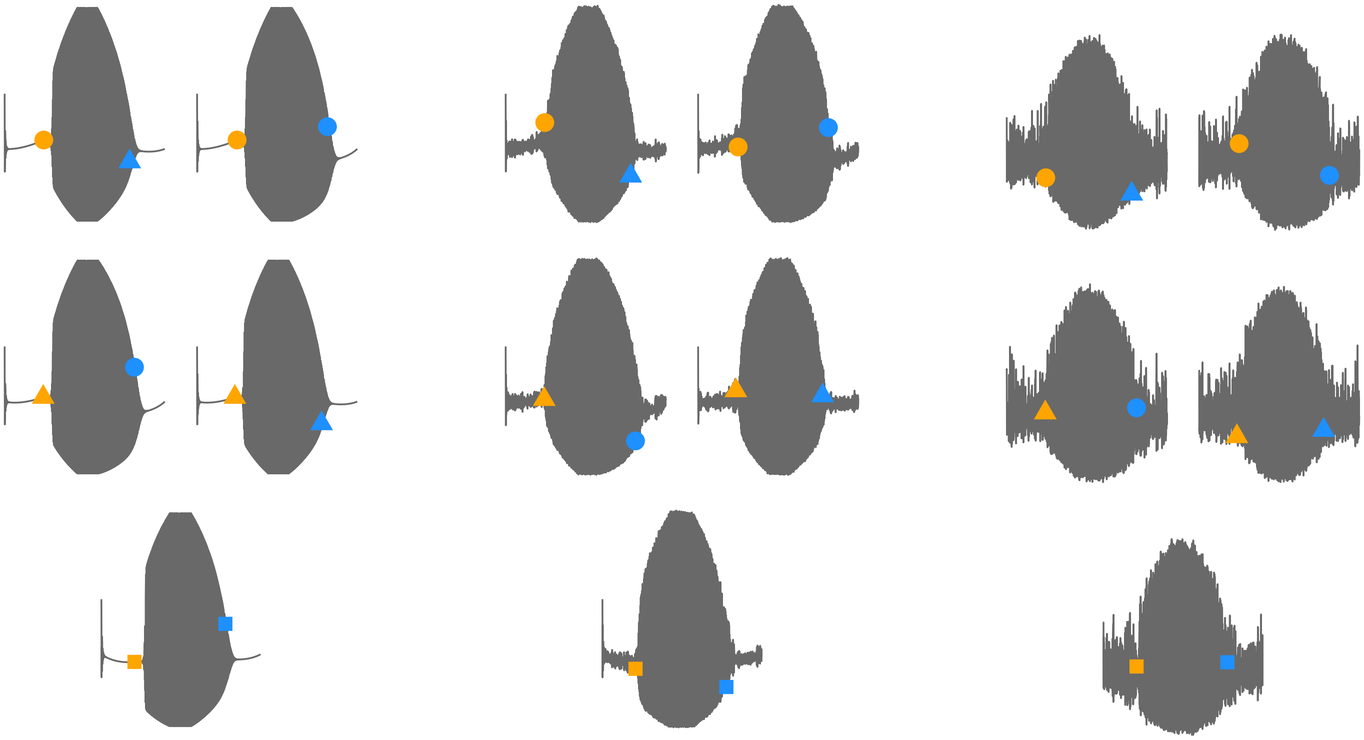

Supplement: Data 2 — PDF version of the atlas of all 16 dynamotypes. Download Data 2, ZIP file. [file eneuro-12-ENEURO.0200-25.2025-s003.zip › Atlas.pdf]
