# Supplementary material for: Dynamotypes for Dummies: A Toolbox, Atlas, and Tutorial for Simulating a Comprehensive Range of Realistic Synthetic Seizures
Source: eNeuro. 2025 Oct 15;12(10):ENEURO.0200-25.2025. doi: 10.1523/ENEURO.0200-25.2025 (PMC12549069; doi:10.1523/ENEURO.0200-25.2025)
Supplement: Data 3 — Microsoft Word and PDF versions of the Matlab Live script “create_database,” which generates large numbers of seizures of different dynamotypes. Download Data 3, ZIP file. [file eneuro-12-ENEURO.0200-25.2025-s004.zip › Create_database.docx]

This script is intended for performing a sweep of the onset and offset curve points to generate simulated seizures across dynamotype classes. It outputs a cell array of seizures, each labeled with its corresponding onset and offset times. The current implementation does **not** sweep over noise levels or model parameters such as k, k_fast, alpha, sigma, or dstar; it uses fixed values for these. The selection of noise is based on .fit models tied to the bifurcation path point for the hysteresis and slow-wave methods. If users wish to vary noise or sweep through additional parameters, they must implement this functionality themselves.

**Challenges of note if you are to also sweep the k, k_fast, alpha, sigma, and dstar parameters**

- Currently the code uses .fit models to pick a noise value associated with the onset and offset point on the bifurcation curve for the hysteresis and slow wave methods. If one wanted to choose noise a different way, note **noise is proportional to seizure length and path length**, which can affect the clarity and consistency of the simulated signal.
- **The bistability region may introduce stuttering** in both the seizure waveform and the x3 variable under high noise conditions for the hysteresis method, making detection more difficult.
- **If you are to vary the k value, smaller k values result in longer seizures**, which may not terminate within the simulation window—so the simulation duration should be extended accordingly for lower k.
- **Parameter values are arbitrary but not random**—not all combinations of k_fast, dtsar, alpha, and k will reliably produce seizures, so parameter tuning is necessary for successful simulation.

addpath('Create Database Helper files');

**Hysteresis generator**

clear all; clc;

seizures = {};

% Settings - Integration

x0=[0;0;0]; % initial conditions (must be a column)

% Settings - Model

% focus

b = 1.0;

% radius of the sphere, do not change

R = 0.4;

% The N parameter controls solution of resting state. Upper Branch (Case 1): Smoother transitions, reduced hysteresis. Lower Branches (Cases 2 and 3): Potential for hysteresis, with complex, path-dependent responses and multiple equilibria. The systems

% state may not revert immediately when external conditions are reversed, creating the characteristic hysteresis loop.

N = 1;

%length of time the simulation will run for

tmax = 100000;

%Integration step/Sampling rate of the simulation, assume it is represented in miliseconds

tstep = 0.01;

%%class wanted to run, input '2s', '2b', '3s','4b','10', '11' '14', '16'

%this shows what labels correspond to what class : '2s - SN/SH', '2b-SN/SH', '3s- SN/SUP','4b - SN/FLC ','10-SN/SH', '11-SN/Sup' '14-SN/Sub', '16-SN/Sup'

class = '2s';

%% function takes in class, and gets a randomized point on each

%% bifurcation curve

[onset_curve,offset_curve]=hysteresis_random_path(class);

onset_curve_length=length(onset_curve);

offset_curve_length=length(offset_curve);

% for loops for k_fast, alpha, k, and dstar parameters if you are to try

% to sweep these parameters

%for k_fast = 0.2:1.3:1.5

%for alpha = 0.2:1.8:2

%for k = 0.0001:0.0009:0.001

%for dstar = 0.3:0.2:0.5

for on=1:5:onset_curve_length

for off=1:20:offset_curve_length

tspan = 0:tstep:tmax;

A = offset_curve(:,off);

B = onset_curve(:,on);

% Create arc path

[E, F] = Parametrization_2PointsArc(A,B,R);

%fixed values of parameters dstar, alpha, k_fast

dstar = 0.3;

k = 0.001;

alpha = 1;

k_fast = 1;

N_t = length(tspan);

X = zeros(3,N_t);

xx = x0;

load("class2s_fit.mat")

%Dynamical pink noise, or parametric noise, is added to the fast variable (x) of'the governing model equations. This represents noise in the brain

%i.e. random voltage fluctuations) that creates small perturbations, some of which'may push the system into or out of the seizure state.

sigma = f(on, off);

Rn = [pinknoise([1,N_t],-1, sigma);pinknoise([1,N_t],-1, 00);pinknoise([1,N_t],-1, 00)];

k = 0.001;

dstar = 0.3;

for n = 1:N_t

% Euler-Meruyama method

Fxx = HysteresisLoop_Model(tspan(n),xx,b,k,k_fast, alpha, R,dstar,E,F,N);

xx = xx + tstep*Fxx + sqrt(tstep)*Rn(:,n);

X(:,n) = xx;

end

x = X';

z = x(:,3);

% Calculate Onset Times

[pks,times]=findpeaks(x(:,3),'MinPeakProminence',0.03);

onset_time = times*tstep;

% Calculate Offset Times

[pks2,times2]=findpeaks(-x(:,3),'MinPeakProminence',0.03);

offset_time = times2*tstep;

% Single seizure

if offset_time(1)>onset_time(1) % if system starts at rest

start_index = max(times(1)-10000,0);

stop_index = min(times2(1)+10000, length(x));

seizure = x(start_index:stop_index,1);

onset = 10000;

offset = stop_index-start_index-10000;

else % if system starts in a seizure

start_index = max(times(1)-10000,0);

stop_index = min(times2(2)+10000, length(x));

seizure = x(start_index:stop_index,1);

onset = 10000;

offset = stop_index-start_index-10000;

end

seizure_arr = {seizure, onset, offset};

seizures{end+1} = seizure_arr;

end

end

% end

% end

% end

% end

# **Slow wave generator**

clear all

seizures = {};

% SETTINGS - INTEGRATION

x0=[0;0;0]; % initial conditions (must be a column)

% Settings - Model

% focus

b = 1.0;

% radius of the sphere, do not change

R = 0.4;

%length of time the seizure will run for

%Integration step/Sampling rate of the simulation

tstep = 0.01;

%%class wanted to run, input 1,5, 6,7,8,9,12,13,15

%this shows what labels correspond to what class : '1 - SN/SNIC', '5

%-SNIC/SNIC', '6 - SNIC/SH','7 - SNIC/SUP','8-Sub/FLC', '9-Sup/SNIC'

%'12-Sup/FLC', '13-Sub/SNIC', '15-Sub/Sup',

class = 1;

load('class1_fit.mat')

%% function takes in class 1,5, 6,7,8,9,12,13,15, and gets a randomized point on each bifurcation curve

[onset_curve,offset_curve,offset_curve2, flag] = slow_wave_circular_random_path(class);

onset_curve_length=length(onset_curve);

offset_curve_length=length(offset_curve);

% for loops for k_fast, alpha, k, and dstar parameters if you are to try

% to sweep these parameters

% for k_fast = 0.1:2:2

% for alpha = 0.1:2:2

% for k = 0.0005:0.001:0.001

for on=1:20:onset_curve_length

for off=1:50:offset_curve_length

%tmax = 2*(2*pi/(k))/tstep;

tmax = 12000;

k = 0.005;

k_fast = 1;

alpha = 1;

if flag == 2 || flag == 3

p1 = onset_curve(:,on);

p2 = offset_curve(:,off);

p3 = offset_curve2;

else

p1 = onset_curve(:,on);

p2 = offset_curve2(:,off);

p3 = offset_curve;

end

tspan = 0:tstep:tmax;

% Create circular path based 3 defining points

[E, F, C, r] = Parametrization_3PointsCircle(p1',p2',p3');

N_t = length(tspan);

X = zeros(3,N_t);

xx = x0;

sigma = f(on, off);

Rn = [pinknoise([1,N_t],-1, sigma);pinknoise([1,N_t],-1, 00);pinknoise([1,N_t],-1, 00)];

mu2_big = zeros(1, length(N_t));

mu1_big = zeros(1, length(N_t));

nu_big = zeros(1, length(N_t));

for n = 1:N_t

%Euler-Meruyama method

[Fxx, mu2, mu1,nu] = SlowWave_Model(tspan(n),xx,b,k,k_fast, alpha, E,F,C,r);

xx = xx + tstep*Fxx + sqrt(tstep)*Rn(:,n);

X(:,n) = xx;

mu2_big(n) = mu2;

mu1_big(n) = mu1;

nu_big(n) = nu;

end

x = X';

% Ensure p1, p2, p3 are column vectors (if not already)

p1 = p1(:);

p2 = p2(:);

p3 = p3(:);

mu1_big = -mu1_big;

%get onset/offset points

tol = 1e-5/2; % Tolerance for floating-point comparison

onset_matches = [];

offset_matches = [];

for i = 1:size(onset_curve, 2)

current_point = onset_curve(:, i);

idx = find( ...

abs(mu2_big(:) - current_point(1)) < tol & ...

abs(mu1_big(:) - current_point(2)) < tol & ...

abs(nu_big(:) - current_point(3)) < tol ...

);

onset_matches = [onset_matches; idx];

end

onset_idx = unique(onset_matches);

if flag == 1

for i = 1:size(offset_curve, 2)

current_point = offset_curve(:, i);

idx = find( ...

abs(mu2_big(:) - current_point(1)) < tol & ...

abs(mu1_big(:) - current_point(2)) < tol & ...

abs(nu_big(:) - current_point(3)) < tol ...

);

offset_matches = [offset_matches; idx];

end

else

for i = 1:size(offset_curve2, 2)

current_point = offset_curve2(:, i);

idx = find( ...

abs(mu2_big(:) - current_point(1)) < tol & ...

abs(mu1_big(:) - current_point(2)) < tol & ...

abs(nu_big(:) - current_point(3)) < tol ...

);

offset_matches = [offset_matches; idx];

end

end

offset_idx = unique(offset_matches);

if(onset_idx(1) < offset_idx(1))

seizure = x(1:onset_idx(2));

seizures{end+1} = {seizure, onset_idx(1), offset_idx(1)};

else

seizure = x(offset_idx(1):onset_idx(2));

seizures{end+1} = {seizure, onset_idx(1)-offset_idx(1), offset_idx(2)-offset_idx(1)};

end

end

end

% end

% end

% end

# Piecewise generator

clear all

seizures = {};

x0=[0;0;0];

% Settings - Model

% focus

b = 1.0;

% radius of the sphere, do not change

R = 0.4;

%Integration step/Sampling rate of the simulation

tstep = 0.01;

%%class wanted to run, input 3,7,9,10,11

%this corresponds to '3 - SN/Sup', '7 - SNIC/Sup', '9- Sup/SNIC', '10 -Sup/SH',

% '11 - Sup/Sup'

class = 11;

[p0,onset_curve,p1_5,offset_curve,p3]=piecewise_random_path(class);

onset_curve_length=length(onset_curve);

offset_curve_length=length(offset_curve);

%for k_fast = 0.1:0.1:2

%for alpha = 0.1:0.1:2

%for k = 0.00001:0.00001:0.001

for on=1:50:onset_curve_length

for off=1:50:offset_curve_length

%for noise= 0:100:3000

noise = 600;

k = 0.0001;

k_fast = 1;

alpha = 1;

p1 = onset_curve(on, :);

p2 = offset_curve(off,:);

stall_val = 30000;

[mu2_straight_path0,mu1_straight_path0,nu_straight_path0,rad1] = sphereArcPath(k,tstep,p0,p1);

[mu2_straight_path0_5,mu1_straight_path0_5,nu_straight_path0_5,rad2] = sphereArcPath(k,tstep,p1,p1_5);

points = repmat(p1_5, stall_val, 1)';

%path noise sigma

sigma = 100;

Rn = [pinknoise([1,length(points)],-1, sigma);pinknoise([1,length(points)],-1, sigma);pinknoise([1,length(points)],-1, sigma)];

points = points + Rn;

[mu2_straight_path,mu1_straight_path,nu_straight_path,rad3] = sphereArcPath(k,tstep,p1_5,p2);

[mu2_straight_path1,mu1_straight_path1,nu_straight_path1,rad4] = sphereArcPath(k,tstep,p2,p3);

mu2_all = [mu2_straight_path0, mu2_straight_path0_5, points(1, :), mu2_straight_path, mu2_straight_path1];

mu1_all = [mu1_straight_path0, mu1_straight_path0_5, points(2, :), mu1_straight_path, mu1_straight_path1];

mu1_all = -mu1_all;

nu_all = [nu_straight_path0, nu_straight_path0_5, points(3,:), nu_straight_path, nu_straight_path1];

N_t = length(mu2_all);

X = zeros(3,N_t);

xx = x0;

%signal pink noise sigma

sigma = noise;

Rn = [pinknoise([1,N_t],-1, sigma);pinknoise([1,N_t],-1, 00);pinknoise([1,N_t],-1, 00)];

mu2_big = zeros(1, length(N_t));

mu1_big = zeros(1, length(N_t));

nu_big = zeros(1, length(N_t));

%%get onset index by finding Radians to bifurcation, and getting index

%%through k and tstep parameters

onset_index = floor((rad1/k)/tstep);

offset_index = floor(((rad1+rad2+rad3)/k)/tstep) + stall_val;

for n = 1:N_t

%%Euler-Meruyama method

[Fxx,mu2,mu1,nu] = SlowWave_Model_piecewise(0,xx,b,k,k_fast, alpha, mu2_all(n), mu1_all(n),nu_all(n));

xx = xx + tstep*Fxx + sqrt(tstep)*Rn(:,n);

X(:,n) = xx;

mu2_big(n) = mu2;

mu1_big(n) = mu1;

nu_big(n) = nu;

end

x = X';

seizure = x(:,1);

seizures{end+1} = {seizure, onset_index, offset_index};

end

end

% end

% end

% end

# Post-processing

post_processed_seizures= {};

for seizure = 1:length(seizures)

data = seizures{seizure}{1};

[pks,locs] = findpeaks(data, 'MinPeakProminence', 0.10);

fs = 1/(0.01*tstep);

t = (0:length(data)-1) / fs;

%%Getting average spike rate

time_in_seconds = locs / fs; % Convert peak indices to seconds

spike_rates = diff(time_in_seconds);

average_frequency = mean(spike_rates);

if average_frequency < 1 || average_frequency > 30

% Calculate spike rates

spike_rates = diff(time_in_seconds);

% Adjust spike rates to achieve a mean average spiking rate of 5 Hz

target_avg_spike_rate = (1/10); % Hz

% Calculate the current average spiking rate

current_avg_spike_rate = mean(spike_rates);

% Calculate the adjustment factor

adjustment_factor = target_avg_spike_rate / current_avg_spike_rate;

% Adjust spike rates

adjusted_spike_rates = spike_rates * adjustment_factor;

% Calculate the mean average spiking rate after adjustment

mean_avg_spike_rate = mean(adjusted_spike_rates);

% Calculate the new sampling frequency

new_sampling_frequency = fs / adjustment_factor;

end

HPF = designfilt('highpassiir', ... % Response type

'FilterOrder',1, ... % Filter Order Specification

'HalfPowerFrequency',0.1, ...

'DesignMethod','butter', ... % Design method

'SampleRate',new_sampling_frequency); % Sample rate

%add pink noise over

data = filter(HPF, data);

min_val = min(data);

max_val = max(data);

data = (data - min_val) / (max_val - min_val);

rms_signal = get_amp(data, new_sampling_frequency);

normalized_data = data;

noisy_data_20 = add_pink_noise(normalized_data, rms_signal, 0.2, new_sampling_frequency);

post_processed_seizures{end+1} = {noisy_data_20, seizures{seizure}{2}, seizures{seizure}{3}};

end

## **Visualize Seizures**

% Select seizure to plot

idx = 1; % choose seizure number

signal = post_processed_seizures{idx}{1}; % processed signal

onset = post_processed_seizures{idx}{2}; % onset index

offset = post_processed_seizures{idx}{3}; % offset index

figure;

plot(signal, 'k', 'LineWidth', 1.2);

hold on;

xline(onset, '--g', 'Onset', 'LineWidth', 1.2);

xline(offset, '--r', 'Offset', 'LineWidth', 1.2);

ylabel('Normalized Amplitude');

title(sprintf('Processed Seizure #%d', idx));

grid on;

# *Functions*

function x_rs = Resting_State(mu2,mu1,nu,N)

switch N

case 1 % resting state on upper branch

x_rs=mu2/(3*(mu1/2 + (mu1^2/4 - mu2^3/27)^(1/2))^(1/3)) + (mu1/2 + (mu1^2/4 - mu2^3/27)^(1/2))^(1/3);

case 2 % resting state on lower branch

x_rs=- mu2/(6*(mu1/2 + (mu1^2/4 - mu2^3/27)^(1/2))^(1/3)) - (mu1/2 + (mu1^2/4 - mu2^3/27)^(1/2))^(1/3)/2 - (3^(1/2)*i*(mu2/(3*(mu1/2 + (mu1^2/4 - mu2^3/27)^(1/2))^(1/3)) - (mu1/2 + (mu1^2/4 - mu2^3/27)^(1/2))^(1/3)))/2;

case 3

x_rs= (3^(1/2)*i*(mu2/(3*(mu1/2 + (mu1^2/4 - mu2^3/27)^(1/2))^(1/3)) - (mu1/2 + (mu1^2/4 - mu2^3/27)^(1/2))^(1/3)))/2 - (mu1/2 + (mu1^2/4 - mu2^3/27)^(1/2))^(1/3)/2 - mu2/(6*(mu1/2 + (mu1^2/4 - mu2^3/27)^(1/2))^(1/3));

end

end

function x_rs=eval_resting_state_cartesian(a,mu2,mu1,N)

switch N

case 1 % resting state

x_rs = ((a.^3 .* mu1)./2 + ((a.^6 .* mu1.^2)./4 - (a.^6 .* mu2.^3)./27).^(1/2)).^(1/3) + ...

(a.^2 .* mu2) ./ (3 .* ((a.^3 .* mu1)./2 + ((a.^6 .* mu1.^2)./4 - (a.^6 .* mu2.^3)./27).^(1/2)).^(1/3));

case 2

x_rs = - (sqrt(3) .* (((a.^3 .* mu1)./2 + ((a.^6 .* mu1.^2)./4 - (a.^6 .* mu2.^3)./27).^(1/2)).^(1/3) - (a.^2 .* mu2) ./ (3 .* ((a.^3 .* mu1)./2 + ((a.^6 .* mu1.^2)./4 - (a.^6 .* mu2.^3)./27).^(1/2)).^(1/3))) .* 1i) ./ 2 ...

- ((a.^3 .* mu1)./2 + ((a.^6 .* mu1.^2)./4 - (a.^6 .* mu2.^3)./27).^(1/2)).^(1/3) ./ 2 ...

- (a.^2 .* mu2) ./ (6 .* ((a.^3 .* mu1)./2 + ((a.^6 .* mu1.^2)./4 - (a.^6 .* mu2.^3)./27).^(1/2)).^(1/3));

case 3

x_rs = (sqrt(3) .* (((a.^3 .* mu1)./2 + ((a.^6 .* mu1.^2)./4 - (a.^6 .* mu2.^3)./27).^(1/2)).^(1/3) - (a.^2 .* mu2) ./ (3 .* ((a.^3 .* mu1)./2 + ((a.^6 .* mu1.^2)./4 - (a.^6 .* mu2.^3)./27).^(1/2)).^(1/3))) .* 1i) ./ 2 ...

- ((a.^3 .* mu1)./2 + ((a.^6 .* mu1.^2)./4 - (a.^6 .* mu2.^3)./27).^(1/2)).^(1/3) ./ 2 ...

- (a.^2 .* mu2) ./ (6 .* ((a.^3 .* mu1)./2 + ((a.^6 .* mu1.^2)./4 - (a.^6 .* mu2.^3)./27).^(1/2)).^(1/3));

end

end

function [E,F] = Parametrization_2PointsArc(A,B,R)

E = A./R;

F=cross(cross(A,B),A);

F=F./norm(F);

end

function x = pinknoise(DIM,BETA, MAG)

%% the function pinknoise(dimension,beta,amplitude) can be used to modify noise

% parameters. The beta parameter is the slope of 1/f^beta. Beta = 0 is white

%noise, beta = -1 is pink, and -2 is brownian. This parameter can be changed to fit the slope of

%desired noise (typically between -0.5 and 1.5), as demonstrated in Suppl Fig. 7 in Jirsa et al. Brain 2014.

%Magnitude can be modified to fit the desired noisiness of the data

% This function generates 1/f spatial noise, with a normal error

% distribution

%

% DIM is a two component vector that sets the size of the spatial pattern

% (DIM=[10,5] is a 10x5 spatial grid)

%

% BETA defines the spectral distribution.

% Spectral density S(f) = N f^BETA

% (f is the frequency, N is normalisation coeff).

% BETA = 0 is random white noise.

% BETA -1 is pink noise

% BETA = -2 is Brownian noise

% The fractal dimension is related to BETA by, D = (6+BETA)/2

%

% MAG is the scaling variable for the noise amplitude

%

% The method is briefly descirbed in Lennon, J.L. "Red-shifts and red

% herrings in geographical ecology", Ecography, Vol. 23, p101-113 (2000)

u = [(0:floor(DIM(1)/2)) -(ceil(DIM(1)/2)-1:-1:1)]'/DIM(1);

u = repmat(u,1,DIM(2));

v = [(0:floor(DIM(2)/2)) -(ceil(DIM(2)/2)-1:-1:1)]/DIM(2);

v = repmat(v,DIM(1),1);

S_f = (u.^2 + v.^2).^(BETA/2);

S_f(S_f==inf) = 0;

phi = rand(DIM);

y= S_f.^0.5 .* (cos(2*pi*phi)+i*sin(2*pi*phi));

y=y.*MAG/max(abs(y)); %set the mag to the level you want

x= ifft2(y);

x = real(x);

end

function Xdot = HysteresisLoop_Model(~,x,~,k,k_fast, alpha, R,dstar,E,F,N)

% Parametrization of the path in the spherical parameter space in terms of great

% circles

mu2=R*(E(1)*cos(x(3))+F(1)*sin(x(3)));

mu1=-R*(E(2)*cos(x(3))+F(2)*sin(x(3)));

nu=R*(E(3)*cos(x(3))+F(3)*sin(x(3)));

% x coordinate of resting state (i.e. upper branch of eq)

x_rs=real(Resting_State(mu2,mu1,nu, N));

%use this to integrate changes in alpha with the resting state

%x_rs=real(eval_resting_state_cartesian(alpha,mu2,mu1,N));

% equations

xdot = -k_fast* alpha*x(2);

ydot = -k_fast*(-(x(1)/alpha)^3 +mu2*(x(1)/alpha) +mu1 + x(2)*( nu + (x(1)/alpha) + (x(1)/alpha)^2));

zdot = -k*(sqrt(((x(1)/alpha)-x_rs)^2+x(2)^2)-dstar);

Xdot = [xdot;ydot;zdot];

end

function [E, F, C, r] = Parametrization_3PointsCircle(p1, p2, p3)

% Calculate unit direction vectors

p1 = p1';

p2 = p2';

p3 = p3';

V12 = (p1 - p2) / norm(p1 - p2);

V13 = (p1 - p3) / norm(p1 - p3);

% Compute the normal vector to the plane defined by the points

n = cross(V12, V13);

n = n / norm(n); % Normalize the normal vector

% Calculate the coefficients for the plane equations

dalpha = dot(p1, n);

dbeta = dot(V12, p1 + (p2 - p1) / 2);

dgamma = dot(V13, p1 + (p3 - p1) / 2);

% Set up the linear equations to find the center C

A = [n(1), n(2), n(3);

V12(1), V12(2), V12(3);

V13(1), V13(2), V13(3)];

b = [dalpha; dbeta; dgamma];

% Solve for C using least squares

C = A\b;

% Calculate E (unit vector from C to p1)

E = (p1 - C) / norm(p1 - C);

% Calculate F (perpendicular vector)

F = -cross(E, n);

% Calculate the radius r

r = norm(p1 - C);

end

function [Xdot, mu2, mu1,nu] = SlowWave_Model(~,x,~,k,k_fast,alpha, E,F,C,r)

% Parametrization of the path in the spherical parameter space in terms

% of a circle defined by 3 points

mu2=C(1)+r*(E(1)*cos(x(3))+F(1)*sin(x(3)));

mu1=-(C(2)+r*(E(2)*cos(x(3))+F(2)*sin(x(3))));

nu=C(3)+r*(E(3)*cos(x(3))+F(3)*sin(x(3)));

% System

xdot = -k_fast* alpha*x(2);

ydot = -k_fast*(-(x(1)/alpha)^3 +mu2*(x(1)/alpha) +mu1 + x(2)*( nu + (x(1)/alpha) + (x(1)/alpha)^2));

zdot = k;

Xdot = [xdot;ydot;zdot];

end

function get_plot()

marker_size = 10;

load('curves.mat')

load('curves2.mat')

load('bifurcation_crossing.mat')

load('sphere_mesh.mat')

load('testmesh.mat');

hold on;

linewidth = 2;

% Plot different meshes with assigned DisplayName for the legend

vertices = BCSmesh.vertices;

faces = BCSmesh.faces;

h1 = patch('Vertices', vertices, 'Faces', faces, ...

'FaceColor', [0.973, 0.965, 0.722], 'EdgeColor', 'none', 'FaceAlpha', 0.6, 'DisplayName', 'BCS Mesh');

vertices = Active_restmesh.vertices;

faces = Active_restmesh.faces;

h2 = patch('Vertices', vertices, 'Faces', faces, ...

'FaceColor', [0.9216, 0.9216, 0.9216], 'EdgeColor', 'none', 'FaceAlpha', 0.6, 'DisplayName', 'Active Rest Mesh');

vertices = Seizure_mesh.vertices;

faces = Seizure_mesh.faces;

h3 = trisurf(faces, vertices(:,1), vertices(:,2), vertices(:,3), ...

'FaceColor', [0.894, 0.706, 0.831], 'EdgeColor', 'none', 'FaceAlpha', 0.3, 'DisplayName', 'Seizure Mesh');

vertices = Bistable_Lcb_mesh.vertices;

faces = Bistable_Lcb_mesh.faces;

h4 = patch('Vertices', vertices, 'Faces', faces, ...

'FaceColor', [0.973, 0.965, 0.722], 'EdgeColor', 'none', 'FaceAlpha', 0.6, 'DisplayName', 'Bistable Lcb Mesh');

scale_array = [0.4];

scale_array = scale_array / 0.4;

% Arrays to store plot handles for the legend

legend_handles = [h1, h2, h3];

legend_names = {'Rest/ Seizure Bistable region', 'Active Rest/Rest Bistable region', 'Seizure region'};

% Scale factors to adjust radius from 0.4

for i = 1:length(scale_array)

% Scale the coordinates of the points for radius 0.39

Fold_of_cycles_scaled = scale_array(i) * Fold_of_cycles;

Homoclinic_to_saddle3_scaled = scale_array(i) * Homoclinic_to_saddle3;

Homoclinic_to_saddle2_scaled = scale_array(i) * Homoclinic_to_saddle2;

Homoclinic_to_saddle1_scaled = scale_array(i) * Homoclinic_to_saddle1;

Homoclinic_to_saddle_scaled = scale_array(i) * Homoclinic_to_saddle;

Fold_scaled = scale_array(i) * Fold;

Hopf_scaled = scale_array(i) * Hopf;

SNIC_scaled = scale_array(i) * SNIC;

% Plot all scaled lines with DisplayName for the legend

h5 = plot3(Fold_of_cycles_scaled(1, :), Fold_of_cycles_scaled(2, :), Fold_of_cycles_scaled(3, :), 'Color', [0.9725,0.2667,0.5843], 'LineWidth', linewidth, 'DisplayName', 'Fold of Cycles');

h6 = plot3(Homoclinic_to_saddle3_scaled(1, :), Homoclinic_to_saddle3_scaled(2, :), Homoclinic_to_saddle3_scaled(3, :), 'Color', [0.404, 0.702, 0.851], 'LineWidth', linewidth, 'LineStyle', '--', 'DisplayName', 'Homoclinic to Saddle 3');

h7 = plot3(Homoclinic_to_saddle2_scaled(1, :), Homoclinic_to_saddle2_scaled(2, :), Homoclinic_to_saddle2_scaled(3, :), 'Color', [0.404, 0.702, 0.851], 'LineWidth', linewidth, 'DisplayName', 'Homoclinic to Saddle 2');

h8 = plot3(Homoclinic_to_saddle1_scaled(1, :), Homoclinic_to_saddle1_scaled(2, :), Homoclinic_to_saddle1_scaled(3, :), 'Color', [0.404, 0.702, 0.851], 'LineWidth', linewidth, 'LineStyle', '--', 'DisplayName', 'Homoclinic to Saddle 1');

h9 = plot3(Homoclinic_to_saddle_scaled(1, :), Homoclinic_to_saddle_scaled(2, :), Homoclinic_to_saddle_scaled(3, :), 'Color', [0.404, 0.702, 0.851], 'LineWidth', linewidth, 'DisplayName', 'Homoclinic to Saddle');

h10 = plot3(Fold_scaled(1, 140:564), Fold_scaled(2, 140:564), Fold_scaled(3, 140:564), 'Color', [0.957, 0.612, 0.204], 'LineWidth', linewidth, 'DisplayName', 'Fold Part 1');

h11 = plot3(Fold_scaled(1, 575:end), Fold_scaled(2, 575:end), Fold_scaled(3, 575:end), 'Color', [0.957, 0.612, 0.204], 'LineWidth', linewidth, 'DisplayName', 'Fold Part 2');

h12 = plot3(Fold_scaled(1, 1:80), Fold_scaled(2, 1:80), Fold_scaled(3, 1:80), 'Color', [0.957, 0.612, 0.204], 'LineWidth', linewidth, 'DisplayName', 'Fold Part 3');

h13 = plot3(Hopf_scaled(1, 1:400), Hopf_scaled(2, 1:400), Hopf_scaled(3, 1:400), 'Color',[0.4549 ,0.7490 ,0.2706], 'LineWidth', linewidth, 'LineStyle', '--', 'DisplayName', 'Hopf Part 1');

h14 = plot3(Hopf_scaled(1, 400:973), Hopf_scaled(2, 400:973), Hopf_scaled(3, 400:973), 'Color',[0.4549 ,0.7490 ,0.2706], 'LineWidth', linewidth, 'DisplayName', 'Hopf Part 2');

h15 = plot3(SNIC_scaled(1, :), SNIC_scaled(2, :), SNIC_scaled(3, :), 'Color',[0.957, 0.612, 0.204], 'LineWidth', linewidth, 'LineStyle', '--', 'DisplayName', 'SNIC');

% Add line handles to the legend array

legend_handles = [legend_handles, h5, h9, h12, h13, h14, h15];

legend_names = [legend_names, 'Fold Limit Cycle', 'SH', 'SN', 'SubH', 'SupH', 'SNIC'];

end

% Add the sphere mesh with transparency

surf(X_sphere, Y_sphere, Z_sphere, 'FaceColor', [0.96, 0.96, 0.86], 'FaceAlpha', 0.2, 'EdgeColor', 'none', 'HandleVisibility', 'off');

% Add labels and view adjustment

xlabel('\mu_2');

ylabel('-\mu_1');

zlabel('\nu');

lineVector = [-0.19, 0.2, 0.07];

az = atan2d(lineVector(2), lineVector(1)); % Azimuth angle

el = atan2d(lineVector(3), norm(lineVector(1:2))); % Elevation angle

view(az, el);

% Display the legend with all handles and names

legend(legend_handles, legend_names);

end

function [onset_curve,offset_curve]=hysteresis_random_path(bifurcation)

load('curves.mat');

if bifurcation == '2s'

onset_curve=SNr_LCs;

offset_curve=SHl;

end

if bifurcation=='2b'

onset_curve=SNr_LCb;

offset_curve=SHb;

end

if bifurcation=='3s'

onset_curve=SNr_LCs;

offset_curve=SNl_ActiveRest;

end

if bifurcation=='4b'

onset_curve=SNr_LCb;

offset_curve=FLC_top;

end

if bifurcation=='10'

onset_curve=SNr_ActiveRest;

offset_curve=SHl;

end

if bifurcation=='11'

onset_curve= SNr_ActiveRest;

offset_curve=[[0.3171; -0.066; 0.2347], [0.3115; -0.0546; 0.2450],[0.3166; -0.0654; 0.2356]];%SNl_ActiveRest;

end

if bifurcation=='14'

onset_curve=subH;

offset_curve=SHb;

end

if bifurcation=='16'

onset_curve=subH;

offset_curve=FLC;

end

end

function [onset_curve,offset_curve,offset_curve2, flag] = slow_wave_circular_random_path(I)

load('curves2.mat');

if I == 1

onset_curve = SHl(:,50:104); %55

offset_curve = [0.33, 0.11, 0.18]';

offset_curve2 = SNIC(:,1:35); %35

flag = 1;

end

if I == 5

onset_curve = SNIC; %44

offset_curve = SNIC; %44

offset_curve2 = [0.34,0.14,0.06]';

flag = 2;

end

if I == 6

onset_curve = SNIC(:,1:35); %35

offset_curve = [0.33, 0.11, 0.18]';

offset_curve2 = SHl(:,50:104); %55

flag = 1;

end

if I == 7

onset_curve=SNIC; %44

offset_curve = Hopf(:,800:855); %56

offset_curve2 = [0.36,-0.12,0.12]';

flag = 3;

end

if I == 8

onset_curve=SNIC; %44

offset_curve = [0.34,0.2,-0.06]';

offset_curve2 = FLC(:,100:300); %201

flag = 1;

end

if I == 9

onset_curve= Hopf(:,800:855); %56

offset_curve = SNIC; %44

offset_curve2 = [0.36,-0.12,0.12]';

flag = 3;

end

if I == 12

onset_curve= Hopf(:,450:495);

offset_curve = FLC(:,60:100);

offset_curve2 = [-0.3, -0.2, -0.2]';

flag = 3;

end

if I == 13

onset_curve = FLC(:,100:300); %201

offset_curve = [0.34,0.2,-0.06]';

offset_curve2 = SNIC;

flag = 1;

end

if I == 15

onset_curve= FLC(:,60:100);%41

offset_curve = Hopf(:,450:495);%46

offset_curve2 = [-0.3, -0.2, -0.2]';

flag = 3;

end

end

function [Xdot, mu2,mu1,nu] = SlowWave_Model_piecewise(~,x,~,k,k_fast, alpha, mu2,mu1,nu)

% Parametrization of the path in the spherical parameter space in terms

% of a circle defined by 3 points

% System

xdot = -k_fast* alpha*x(2);

ydot = -k_fast*(-(x(1)/alpha)^3 +mu2*(x(1)/alpha) +mu1 + x(2)*( nu + (x(1)/alpha) + (x(1)/alpha)^2));

zdot = k;

Xdot = [xdot;ydot;zdot];

end

function [mu2,mu1,nu, theta] = sphereArcPath(k, tstep,point1, point2)

% sphereArcPath - Generates an arc path between two points on a sphere

%

% Syntax: arcPath = sphereArcPath(point1, point2, numPoints)

%

% Inputs:

% point1 - [x1, y1, z1] Coordinates of the first point on the sphere

% point2 - [x2, y2, z2] Coordinates of the second point on the sphere

% numPoints - Number of points along the arc

%

% Outputs:

% arcPath - An Nx3 matrix containing the coordinates of points along the arc

% Check the input points

radius = 0.4;

% if norm(point1) ~= radius || norm(point2) ~= radius

% error('The points must lie on the sphere of radius 0.4.');

% end

% Normalize the input points to make sure they are on the sphere

point1 = point1 / norm(point1) * radius;

point2 = point2 / norm(point2) * radius;

% Compute the quaternion for rotation

theta = acos(dot(point1, point2) / (radius^2));

axis = cross(point1, point2);

if norm(axis) == 0

error('The points are the same or antipodal.');

end

axis = axis / norm(axis);

% Compute points along the arc

numPoints = floor((theta/k)/tstep);

arcPath = zeros(numPoints, 3);

for i = 0:numPoints-1

t = i / (numPoints - 1);

angle = t * theta;

R = rotationMatrix(axis, angle);

arcPath(i+1, :) = (R * point1')';

end

mu2 = arcPath(:,1)';

mu1 = arcPath(:,2)';

nu = arcPath(:,3)';

end

function R = rotationMatrix(axis, angle)

% rotationMatrix - Generates a rotation matrix given an axis and an angle

%

% Syntax: R = rotationMatrix(axis, angle)

%

% Inputs:

% axis - A 3-element vector representing the axis of rotation

% angle - The angle of rotation in radians

%

% Outputs:

% R - A 3x3 rotation matrix

ux = axis(1);

uy = axis(2);

uz = axis(3);

c = cos(angle);

s = sin(angle);

t = 1 - c;

R = [t*ux*ux + c, t*ux*uy - s*uz, t*ux*uz + s*uy;

t*ux*uy + s*uz, t*uy*uy + c, t*uy*uz - s*ux;

t*ux*uz - s*uy, t*uy*uz + s*ux, t*uz*uz + c];

end

function point= get_random_point

radius = 0.4;

% Generate two random numbers

theta = 2 * pi * rand(); % Random angle between 0 and 2*pi

phi = acos(2 * rand() - 1); % Random angle between 0 and pi

% Convert spherical coordinates to Cartesian coordinates

x = radius * sin(phi) * cos(theta);

y = radius * sin(phi) * sin(theta);

z = radius * cos(phi);

point = [x,y,z];

% Display the point

end

function point= get_random_point_hopf

%load("map_regions.mat")

radius = 0.4;

% Loop until a valid point with y > 0 is found

while true

% Generate two random numbers

theta = 2 * pi * rand(); % Random angle between 0 and 2*pi

phi = acos(2 * rand() - 1); % Random angle between 0 and pi

% Convert spherical coordinates to Cartesian coordinates

x = radius * sin(phi) * cos(theta);

y = radius * sin(phi) * sin(theta);

z = radius * cos(phi);

% Check if y is positive

if y > 0

point = [x, y, z];

break;

end

end

% Display the point

end

%

% Display the point

function point= get_random_point_fixed

radius = 0.4;

% Loop until a valid point with y > 0 is found

while true

% Generate two random numbers

theta = 2 * pi * rand(); % Random angle between 0 and 2*pi

phi = acos(2 * rand() - 1); % Random angle between 0 and pi

% Convert spherical coordinates to Cartesian coordinates

x = radius * sin(phi) * cos(theta);

y = radius * sin(phi) * sin(theta);

z = radius * cos(phi);

% Check if y is positive

if y < 0

point = [x, y, z];

break;

end

end

% Display the point

end

function [mu2,mu1,nu] = sphere(point1, point2, numPoints)

% sphereArcPath - Generates an arc path between two points on a sphere

%

% Syntax: arcPath = sphereArcPath(point1, point2, numPoints)

%

% Inputs:

% point1 - [x1, y1, z1] Coordinates of the first point on the sphere

% point2 - [x2, y2, z2] Coordinates of the second point on the sphere

% numPoints - Number of points along the arc

%

% Outputs:

% arcPath - An Nx3 matrix containing the coordinates of points along the arc

% Check the input points

radius = 0.4;

% if norm(point1) ~= radius || norm(point2) ~= radius

% error('The points must lie on the sphere of radius 0.4.');

% end

% Normalize the input points to make sure they are on the sphere

point1 = point1 / norm(point1) * radius;

point2 = point2 / norm(point2) * radius;

% Compute the quaternion for rotation

theta = acos(dot(point1, point2) / (radius^2));

axis = cross(point1, point2);

if norm(axis) == 0

error('The points are the same or antipodal.');

end

axis = axis / norm(axis);

% Compute points along the arc

arcPath = zeros(numPoints, 3);

for i = 0:numPoints-1

t = i / (numPoints - 1);

angle = t * theta;

R = rotationMatrix(axis, angle);

arcPath(i+1, :) = (R * point1')';

end

mu2 = arcPath(:,1)';

mu1 = arcPath(:,2)';

nu = arcPath(:,3)';

end

function [arcLength, theta] = calculateArcLength(P1, P2, radius)

% calculateArcLength computes the arc length and central angle between two points on a sphere.

%

% Input:

% P1 - First point [x1, y1, z1]

% P2 - Second point [x2, y2, z2]

% radius - Radius of the sphere (default: 0.4 if not provided)

%

% Output:

% arcLength - Arc length between the two points

% theta - Central angle between the two points in radians

if nargin < 3

radius = 0.4;

end

% Compute the dot product of P1 and P2

dotProduct = dot(P1, P2);

% Compute the magnitudes of P1 and P2

magnitudeP1 = norm(P1);

magnitudeP2 = norm(P2);

% Compute the cosine of the central angle

cosTheta = dotProduct / (magnitudeP1 * magnitudeP2);

% Compute the central angle in radians

theta = acos(cosTheta);

% Compute the arc length

arcLength = radius * theta;

end

function [p0,p1,p1_5,p2,p3]=piecewise_random_path(bifurcation)

load('curves.mat');

load('bifurcation_crossing.mat')

load("curves2.mat")

if bifurcation==3

%fixed rest point

p0 = Hopf(:,930)';

%bifurcation curve

randomNumber = randi([145,170]);

p1 = Fold(:,145:170)';

randomNumber2 = randi([600,750]);

p1_5 = [0.3196, 0.2389, -0.0279];

%bifurcation curve

p2 = Hopf(:,600:750)' ;

%fixed rest

p3 = [ 0.1944 , 0.0893 , 0.3380];

end

if bifurcation==7

randomNumber = randi([600,750]);

randomNumber2 = randi([1,44]);

%fixed rest point

p0 = Fold(:,400)';

%bifurcation curve

p1 = SNIC' ;

%random point in limit cycle

p1_5 = [0.1314, 0.3298, -0.1843];

%bifurcation curve

p2 = Hopf(:,600:750)';

%fixed rest

p3 = [ 0.1944 , 0.0893 , 0.3380];

end

if bifurcation==9

randomNumber = randi([600,750]);

%fixed rest point

p0 = [ 0.1944 , 0.0893 , 0.3380];

%bifurcation curve

p1 = Hopf(:,600:750)';

%change here

randomNumber2 = randi([1,44]);

%random point in limit cycle

p1_5 = [-0.0441, 0.2591, -0.3015];

%bifurcation curve

p2 = SNIC' ;

%fixed rest

p3 = Fold(:,450)';

end

if bifurcation==10

randomNumber = randi([600,750]);

%fixed rest point

p0 = [ 0.1944 , 0.0893 , 0.3380];

%bifurcation curve

p1 = Hopf(:,600:750)';%get_nearest_hopf(p0(1),p0(2),p0(3))';

%change here

randomNumber2 = randi([1,124]);

%random point in limit cycle

p1_5 = [-0.123686721647726 0.338825918756816 -0.172912092308889];

%bifurcation curve

p2 = Homoclinic_to_saddle' ;

%fixed rest

p3 = Fold(:,400)';

end

if bifurcation==11

randomNumber = randi([600,750]);

%fixed rest point

p0 = [ 0.1944 , 0.0893 , 0.3380];

%bifurcation curve

p1 = Hopf(:,600:750)';

%change here

randomNumber2 = randi([600,750]);

%random point in limit cycle

p1_5 = [-0.2104, 0.3180, -0.1209];

%bifurcation curve

p2 = Hopf(:,600:750)' ;

%fixed rest

p3 = [ 0.1944 , 0.0893 , 0.3380];

end

end

function [new_X, new_Y, new_Z] = get_random_walk(p1,p2,region,k,tstep, minSteps, maxSteps)

load('logicgrid.mat')

% if region == 11

% load('logicgrid.mat'); % Load the logic grid from the .mat file

%

% elseif region == 'LCS_bistable'

% load("logicgrid_bistable_lcs.mat")

%

% elseif region == 'LCB_bistable'

% load("logicgrid_bistable_lcb.mat")

% end

grid = logic_grid;

[theta, phi, r] = cart2sph(p1(1), p1(2), p1(3));

theta_offset_deg = -40; % For example, a 30-degree rotation

% Convert the offset to radians

theta_offset_rad = deg2rad(theta_offset_deg);

theta_deg_p1 = rad2deg(theta+theta_offset_rad);

phi_deg_p1 = rad2deg(phi);

p1_5 = get_nearest_seizure_point(theta_deg_p1, phi_deg_p1, region);

[theta, phi, r] = cart2sph(p2(1), p2(2), p2(3));

theta_offset_deg = -40; % For example, a 30-degree rotation

% Convert the offset to radians

theta_offset_rad = deg2rad(theta_offset_deg);

theta_deg_p2 = rad2deg(theta+theta_offset_rad);

phi_deg_p2 = rad2deg(phi);

p2_5 = get_nearest_seizure_point(theta_deg_p2, phi_deg_p2, region);

% Load the grid (assuming the variable is 'grid')

% Define the start and end points

startPoint = p1_5; % Define the start point (row, column)

endPoint = p2_5; % Define the end point (row, column)

currentPos = startPoint;

%

% % Store the path

path = currentPos;

%

% % Define the movement directions (up, down, left, right)

directions = [0 1; 0 -1; 1 0; -1 0]; % [row_change, col_change]

% Get the size of the grid

[gridRows, gridCols] = size(grid);

% Loop until a valid path is found

% Loop until a valid path is found

while true

% Initialize the current position at the start point

currentPos = startPoint;

% Store the path, starting from the initial position

path = currentPos;

stepCount = 0;

% Perform the random walk until either endpoint is reached or maxSteps

while stepCount < maxSteps

% Check if the endpoint has been reached and steps are within bounds

if isequal(currentPos, endPoint) && stepCount >= minSteps

disp('Valid path found within the specified range of steps.');

break;

end

% Choose a random direction to move

randomDir = directions(randi(4), :);

% Compute the new position

newPos = currentPos + randomDir;

% Check if the new position is within bounds and valid (e.g., grid value is 1)

if newPos(1) > 0 && newPos(1) <= gridRows && newPos(2) > 0 && newPos(2) <= gridCols

if grid(newPos(1), newPos(2)) == 1

% Update the current position

currentPos = newPos;

% Add the new position to the path

path = [path; currentPos];

% Increment the step count

stepCount = stepCount + 1;

end

end

end

% Check if a valid path was found within bounds

if isequal(currentPos, endPoint) && stepCount >= minSteps && stepCount <= maxSteps

% Exit the loop if a valid path is found

break;

end

% If not, repeat the simulation

end

% Plot the random walk path

figure;

imagesc(grid); % Show the grid

colormap(gray); % Use a gray color map (1 = white, 0 = black)

hold on;

plot(path(:,2), path(:,1), 'r.-', 'LineWidth', 2, 'MarkerSize', 15); % Plot the path

% Plot the start point in green

plot(startPoint(2), startPoint(1), 'go', 'MarkerSize', 10, 'MarkerFaceColor', 'g');

% Plot the end point in blue

plot(endPoint(2), endPoint(1), 'bo', 'MarkerSize', 10, 'MarkerFaceColor', 'b');

title('2D Random Walk Path on the Logic Grid');

xlabel('X');

ylabel('Y');

if region == 11

load('logicgrid.mat'); % Load the logic grid from the .mat file

elseif region == 'LCS_bistable'

load("logicgrid_bistable_lcs.mat")

elseif region == 'LCB_bistable'

load("logicgrid_bistable_lcb.mat")

end

% Convert back to x, y coordinates

x_back = (path(:,1) - 1) / (grid_size(1) - 1) * (x_max - x_min) + x_min;

y_back = (path(:,2) - 1) / (grid_size(2) - 1) * (y_max - y_min) + y_min;

theta_rad = deg2rad(x_back) + deg2rad(-theta_offset_deg);

phi_rad = deg2rad(y_back);

radius = 0.4; % Set the radius of the sphere

[X, Y, Z] = sph2cart(theta_rad, phi_rad, radius);

r = 0.4;

P1 = [X(1);Y(1);Z(1)];

P2 = [X(2);Y(2);Z(2)];

% Compute the angle between the two points using the dot product formula

cos_theta = dot(P1, P2) / (r^2);

theta = acos(cos_theta); % Angle in radians

total_theta = (length(X)-1)*theta;

numPoints = floor((total_theta/k)/tstep);

% Create interpolation queries and interpolate X, Y, Z coordinates

xq = linspace(1, length(X), numPoints); % Query points for interpolation

new_X = interp1(1:length(X), X, xq, 'linear');

new_Y = interp1(1:length(Y), Y, xq, 'linear');

new_Z = interp1(1:length(Z), Z, xq, 'linear');

end

function nearestPoint= get_nearest_seizure_point(theta,phi, region)

load('logicgrid.mat'); % Load the logic grid from the .mat file

% if region == 11

% load('logicgrid.mat'); % Load the logic grid from the .mat file

%

% elseif region == 'LCS_bistable'

% load("logicgrid_bistable_lcs.mat")

%

% elseif region == 'LCB_bistable'

% load("logicgrid_bistable_lcb.mat")

% end

x = [theta,phi];

[row, col] = find(logic_grid);

x(1) = round((theta - x_min) / (x_max - x_min) * (grid_size(1) - 1)) + 1;

x(2) = round((phi - y_min) / (y_max - y_min) * (grid_size(2) - 1)) + 1;

% Compute the Euclidean distance between x and each point in the grid

distances = sqrt((row - x(1)).^2 + (col - x(2)).^2);

% Find the index of the minimum distance

[~, minIndex] = min(distances);

% Get the coordinates of the nearest point

nearestPoint = [row(minIndex), col(minIndex)];

end

function noisy_signal = add_pink_noise(signal, rms_signal, noise_amplitude_ratio, fs)

% Inputs:

% signal - input signal (1D array)

% noise_amplitude_ratio - fraction of signal amplitude for noise (e.g., 0.4 for 40%)

% Compute the RMS amplitude of the signal

% Generate pink noise of the same length as the signal

% Pink noise can be generated using dsp.ColoredNoise in MATLAB

L = length(signal);

pink_noise = pinknoise([1,L],-1,10000)';

%

% % % Scale the noise so its amplitude is noise_amplitude_ratio of the signal's amplitude

% scaling_factor = noise_amplitude_ratio * (1 / rms_noise);

% scaled_noise = pink_noise * scaling_factor;

min_val = min(pink_noise(:));

max_val = max(pink_noise(:));

scaled_noise = noise_amplitude_ratio*(pink_noise - min_val) / (max_val - min_val);

min_val = min(signal(:));

max_val = max(signal(:));

scaled_signal = (signal - min_val) / (max_val - min_val);

% Add the scaled noise to the original signal

noisy_signal = scaled_signal + scaled_noise;

end

function amp = get_amp(signal, fs)

[peaks,locs] = findpeaks(signal ,'MinPeakProminence', 0.15);

[troughs_neg,locs_troughs] = findpeaks(signal, 'MinPeakProminence', 0.15);

troughs = -1*troughs_neg;

newnew = [];

len = 0;

if length(troughs) > length(peaks)

len = length(peaks);

else

len = length(troughs);

end

for i = 1:len

newnew = [newnew; abs(troughs(i) - peaks(i))];

end

amp = mean(newnew);

end

function [start_index, stop_index, signal] = bifurcation_all_class(class, tstep, sigma)

R = 1;

N = 1;

b = 0;

x0 = [0;0;0];

k =0.007;

dstar = 0.3;

tstep = 0.1;

tmax = 75000;

if ismember(class, [1 5 6 8 12 13 15])

% Code for class 1

% Perform specific operations for class 1

[onset_curve,offset_curve,p3, flag] = slow_wave_circular_random_path(class);

onset_curve_length=length(onset_curve);

offset_curve_length=length(offset_curve);

random_onset_index=randsample(onset_curve_length,1);

random_offset_index=randsample(offset_curve_length,1);

if flag == 2 || flag == 3

p1 = onset_curve(:,random_onset_index);

p2 = offset_curve(:,random_offset_index);

else

p1 = onset_curve(:,random_onset_index);

p2 = p3(:,random_offset_index);

p3 = offset_curve;

end

% uncomment this code to do random path

% % One random path - select random point on onset curve and offset curve

tspan = 0:tstep:tmax;

% Create circular path based 3 defining points

[E, F, C, r] = Parametrization_3PointsCircle(p1',p2',p3');

if class == 13

E = -E;

end

N_t = length(tspan);

X = zeros(3,N_t);

xx = x0;

sigma = 40;

Rn = [pinknoise([1,N_t],-1, sigma);pinknoise([1,N_t],-1, 00);pinknoise([1,N_t],-1, 00)];

mu2_big = zeros(1, length(N_t));

mu1_big = zeros(1, length(N_t));

nu_big = zeros(1, length(N_t));

for n = 1:N_t

%Euler-Meruyama method

[Fxx, mu2, mu1,nu] = SlowWave_Model(tspan(n),xx,b,k,E,F,C,r);

xx = xx + tstep*Fxx + sqrt(tstep)*Rn(:,n);

X(:,n) = xx;

mu2_big(n) = mu2;

mu1_big(n) = mu1;

nu_big(n) = nu;

end

x = X';

t = tspan;

%%Onset and offset calculation, calculates radians to the bifurcation

%%curve, then uses tstep and k variables to compute onset location

plot_onset_offset = 0;

if(floor((((2*pi)/k)/tstep)) < N_t)

plot_onset_offset = 1;

point1 = p1' - C;

point2 = p2' - C;

point3 = p3'-C;

point1 = point1 / norm(point1) * r;

point2 = point2 / norm(point2) * r;

point3 = point3 / norm(point3) * r;

% Compute the quaternion for rotation

theta1 = acos(dot(point1, point2) / (r^2));

%%%change here

numPoints1 = floor((theta1/k)/tstep);

point = [mu2_big(numPoints1), -mu1_big(numPoints1), nu_big(numPoints1)];

if round(point,2) == round(p2,2)'

onset_index = numPoints1;

else

numPoints1 = floor(((2*pi - theta1)/k)/tstep);

onset_index = numPoints1;

theta1 = theta1-2*pi;

end

theta2 = acos(dot(point1, point3) / (r^2));

numPoints2 = floor(((theta2)/k)/tstep);

point = [mu2_big(numPoints2), -mu1_big(numPoints2), nu_big(numPoints2)];

if round(point,2) == round(p2,2)'

%offset_index = numPoints2;

else

numPoints2 = floor(((2*pi - theta2)/k)/tstep);

theta2 = 2*pi - theta2;

offset_index = numPoints2;

end

theta3 = 2*pi;

numPoints3 = floor(((theta3)/k)/tstep);

point = [mu2_big(numPoints3), -mu1_big(numPoints3), nu_big(numPoints3)];

offset_index = numPoints3;

end

if class == 15 || class == 12

onset_index_temp = onset_index;

onset_index = offset_index;

offset_index = onset_index_temp + floor((((2*pi)/k)/tstep));

end

start_index = max(1,onset_index-1000);

stop_index = min(offset_index+1000, length(x));

signal = x(start_index:stop_index,1);

elseif ismember(class, ['2s' '2b' '4b' '14' '16'])

k=0.01;

tmax = 75000;

dstar = 0.3;

[onset_curve,offset_curve]=hysteresis_random_path(class);

onset_curve_length=length(onset_curve);

offset_curve_length=length(offset_curve);

% uncomment this code to do random path

% % One random path - select random point on onset curve and offset curve

random_onset_index=randsample(onset_curve_length,1);

random_offset_index=randsample(offset_curve_length,1);

A = offset_curve(:,random_offset_index);

B = onset_curve(:,random_onset_index);

tspan = 0:tstep:tmax;

% Create circular path based 3 defining points

[E, F] = Parametrization_2PointsArc(A,B,R);

N_t = length(tspan);

X = zeros(3,N_t);

xx = x0;

Rn = [pinknoise([1,N_t],-1, sigma);pinknoise([1,N_t],-1, 00);pinknoise([1,N_t],-1, 00)];

for n = 1:N_t

% Euler-Meruyama method

Fxx = HysteresisLoop_Model(tspan(n),xx,b,k,R,dstar,E,F,N);

xx = xx + tstep*Fxx + sqrt(tstep)*Rn(:,n);

X(:,n) = xx;

end

x = X';

[pks,times]=findpeaks(x(:,3),'MinPeakProminence',0.03);

onset_time = times*tstep;

% Calculate Offset Times

[pks2,times2]=findpeaks(-x(:,3),'MinPeakProminence',0.03);

offset_time = times2*tstep;

% Single seizure

if offset_time(1)>onset_time(1) % if system starts at rest

start_index = times(1)-10000;

stop_index = times2(1)+10000;

start_index = max(1, start_index);

stop_index = min(length(x), stop_index);

signal = x(start_index:stop_index,1);

onset = times(1);

offset = stop_index-start_index-10000;

else % if system starts in a seizure

start_index = times(1)-10000;

stop_index = times2(2)+10000;

start_index = max(1, start_index);

stop_index = min(length(x), stop_index);

signal = x(start_index:stop_index,1);

onset = times(1);

offset = stop_index-start_index-10000;

end

elseif ismember(class, [3 7 9 10 11])

k = 0.005;

[p0,onset_curve,p1_5,offset_curve,p3]=piecewise_random_path(class);

onset_curve_length=length(onset_curve);

offset_curve_length=length(offset_curve);

onset_curve = onset_curve';

offset_curve = offset_curve';

% uncomment this code to do random path

% % One random path - select random point on onset curve and offset curve

random_onset_index=randsample(onset_curve_length,1);

random_offset_index=randsample(offset_curve_length,1);

p1 = onset_curve(:,random_onset_index)';

p2 = offset_curve(:,random_offset_index)';

stall_val = 30000;

[mu2_straight_path0,mu1_straight_path0,nu_straight_path0,rad1] = sphereArcPath(k,tstep,p0,p1);

[mu2_straight_path0_5,mu1_straight_path0_5,nu_straight_path0_5,rad2] = sphereArcPath(k,tstep,p1,p1_5);

points = repmat(p1_5, stall_val, 1)';

%path noise sigma

sigma_pathnoise = 100;

Rn = [pinknoise([1,length(points)],-1, sigma_pathnoise);pinknoise([1,length(points)],-1, sigma_pathnoise);pinknoise([1,length(points)],-1, sigma_pathnoise)];

points = points + Rn;

[mu2_straight_path,mu1_straight_path,nu_straight_path,rad3] = sphereArcPath(k,tstep,p1_5,p2);

[mu2_straight_path1,mu1_straight_path1,nu_straight_path1,rad4] = sphereArcPath(k,tstep,p2,p3);

mu2_all = [mu2_straight_path0, mu2_straight_path0_5, points(1, :), mu2_straight_path, mu2_straight_path1];

mu1_all = [mu1_straight_path0, mu1_straight_path0_5, points(2, :), mu1_straight_path, mu1_straight_path1];

mu1_all = -mu1_all;

nu_all = [nu_straight_path0, nu_straight_path0_5, points(3,:), nu_straight_path, nu_straight_path1];

N_t = length(mu2_all);

X = zeros(3,N_t);

xx = x0;

Rn = [pinknoise([1,N_t],-1, sigma);pinknoise([1,N_t],-1, 00);pinknoise([1,N_t],-1, 00)];

mu2_big = zeros(1, length(N_t));

mu1_big = zeros(1, length(N_t));

nu_big = zeros(1, length(N_t));

%%get onset index by finding Radians to bifurcation, and getting index

%%through k and tstep parameters

onset_index = floor((rad1/k)/tstep);

offset_index = floor(((rad1+rad2+rad3)/k)/tstep) + stall_val;

for n = 1:N_t

%%Euler-Meruyama method

[Fxx,mu2,mu1,nu] = SlowWave_Model_piecewise(0,xx,b,k,mu2_all(n), mu1_all(n),nu_all(n));

xx = xx + tstep*Fxx + sqrt(tstep)*Rn(:,n);

X(:,n) = xx;

mu2_big(n) = mu2;

mu1_big(n) = mu1;

nu_big(n) = nu;

end

x = X';

signal = x(:,1);

start_index = 1;

stop_index = length(signal);

end

end
