# Supplementary material for: Dynamotypes for Dummies: A Toolbox, Atlas, and Tutorial for Simulating a Comprehensive Range of Realistic Synthetic Seizures
Source: eNeuro. 2025 Oct 15;12(10):ENEURO.0200-25.2025. doi: 10.1523/ENEURO.0200-25.2025 (PMC12549069; doi:10.1523/ENEURO.0200-25.2025)
Supplement: Data 3 — Microsoft Word and PDF versions of the Matlab Live script “create_database,” which generates large numbers of seizures of different dynamotypes. Download Data 3, ZIP file. [file eneuro-12-ENEURO.0200-25.2025-s004.zip › Create_database.pdf]

This script is intended for performing a sweep of the onset and offset curve points to generate simulated seizures across dynamotype classes. It outputs a cell array of seizures, each labeled with its corresponding onset and offset times. The current implementation does **not** sweep over noise levels or model parameters such as `k`, `k_fast`, `alpha`, `sigma`, or `dstar`; it uses fixed values for these. The selection of noise is based on `.fit` models tied to the bifurcation path point for the hysteresis and slow-wave methods. If users wish to vary noise or sweep through additional parameters, they must implement this functionality themselves.

Challenges of note if you are to also sweep the k, k_fast, alpha, sigma, and dstar parameters

- Currently the code uses .fit models to pick a noise value associated with the onset and offset point on the bifurcation curve for the hysteresis and slow wave methods. If one wanted to choose noise a different way, note **noise is proportional to seizure length and path length**, which can affect the clarity and consistency of the simulated signal.
- **The bistability region may introduce stuttering** in both the seizure waveform and the x3 variable under high noise conditions for the hysteresis method, making detection more difficult.
- **If you are to vary the k value, smaller k values result in longer seizures**, which may not terminate within the simulation window—so the simulation duration should be extended accordingly for lower k.
- **Parameter values are arbitrary but not random**—not all combinations of k_fast, dtsar, alpha, and k will reliably produce seizures, so parameter tuning is necessary for successful simulation.

```
addpath('Create Database Helper files');
```

Hysteresis generator

```
clear all; clc;
seizures = {};

% Settings - Integration
x0=[0;0;0]; % initial conditions (must be a column)

% Settings - Model
% focus
b = 1.0;

% radius of the sphere, do not change
R = 0.4;


 % The N parameter controls solution of resting state. Upper Branch (Case 1): Smoother
transitions, reduced hysteresis. Lower Branches (Cases 2 and 3): Potential for hysteresis, with
complex, path-dependent responses and multiple equilibria. The systems
 % state may not revert immediately when external conditions are reversed, creating the
characteristic hysteresis loop.
 N = 1;

 %length of time the simulation will run for
 tmax = 100000;

 %Integration step/Sampling rate of the simulation, assume it is represented in miliseconds
```

```
tstep = 0.01;

%%class wanted to run, input '2s',  '2b', '3s','4b','10', '11' '14', '16'
%this shows what labels correspond to what class : '2s - SN/SH',  '2b-SN/SH', '3s- SN/SUP','4b
- SN/FLC ','10-SN/SH', '11-SN/Sup' '14-SN/Sub', '16-SN/Sup'
class = '2s';

%% function takes in class, and gets a randomized point on each
%% bifurcation curve
[onset_curve,offset_curve]=hysteresis_random_path(class);


onset_curve_length=length(onset_curve);
offset_curve_length=length(offset_curve);

% for loops for k_fast, alpha, k, and dstar parameters if you are to try
% to sweep these parameters
%for k_fast = 0.2:1.3:1.5
%for alpha = 0.2:1.8:2
%for k = 0.0001:0.0009:0.001
%for dstar = 0.3:0.2:0.5
for on=1:5:onset_curve_length
for off=1:20:offset_curve_length
tspan = 0:tstep:tmax;
A = offset_curve(:,off);
B = onset_curve(:,on);
% Create arc path
[E, F] = Parametrization_2PointsArc(A,B,R);

%fixed values of parameters dstar, alpha, k_fast
dstar = 0.3;
k = 0.001;
alpha = 1;
k_fast = 1;
N_t = length(tspan);
X = zeros(3,N_t);
xx = x0;
load("class2s_fit.mat")

%Dynamical pink noise, or parametric noise, is added to the fast variable (x) of'the governing
model equations. This represents noise in the brain
%i.e. random voltage fluctuations) that creates small perturbations, some of which'may push
the system into or out of the seizure state.
sigma = f(on, off);
Rn = [pinknoise([1,N_t],-1, sigma);pinknoise([1,N_t],-1, 00);pinknoise([1,N_t],-1, 00)];
k = 0.001;
dstar = 0.3;
for n = 1:N_t

    % Euler-Meruyama method
    Fxx = HysteresisLoop_Model(tspan(n),xx,b,k,k_fast, alpha, R,dstar,E,F,N);
    xx = xx + tstep*Fxx + sqrt(tstep)*Rn(:,n);
```

```
    X(:,n) = xx;

end

x = X';
z = x(:,3);


% Calculate Onset Times
[pks,times]=findpeaks(x(:,3),'MinPeakProminence',0.03);
onset_time = times*tstep;


% Calculate Offset Times
[pks2,times2]=findpeaks(-x(:,3),'MinPeakProminence',0.03);
offset_time = times2*tstep;




% Single seizure
if offset_time(1)>onset_time(1) % if system starts at rest
    start_index = max(times(1)-10000,0);
    stop_index = min(times2(1)+10000, length(x));
    seizure = x(start_index:stop_index,1);
    onset = 10000;
    offset = stop_index-start_index-10000;
else % if system starts in a seizure
    start_index = max(times(1)-10000,0);
    stop_index = min(times2(2)+10000, length(x));
    seizure = x(start_index:stop_index,1);
    onset = 10000;
    offset = stop_index-start_index-10000;
end
seizure_arr = {seizure, onset, offset};
seizures{end+1} = seizure_arr;



    end
end
% end
% end
% end
% end
```

Slow wave generator

```
clear all
seizures = {};
% SETTINGS - INTEGRATION
```

```
x0=[0;0;0]; % initial conditions (must be a column)

% Settings - Model
% focus
b = 1.0;

% radius of the sphere, do not change
R = 0.4;


%length of time the seizure will run for


%Integration step/Sampling rate of the simulation
tstep = 0.01;

%%class wanted to run, input 1,5, 6,7,8,9,12,13,15
%this shows what labels correspond to what class : '1 - SN/SNIC',  '5
%-SNIC/SNIC', '6 - SNIC/SH','7 - SNIC/SUP','8-Sub/FLC', '9-Sup/SNIC'
%'12-Sup/FLC', '13-Sub/SNIC', '15-Sub/Sup',
class = 1;
load('class1_fit.mat')
%% function takes in class 1,5, 6,7,8,9,12,13,15, and gets a randomized point on each
bifurcation curve
[onset_curve,offset_curve,offset_curve2, flag] = slow_wave_circular_random_path(class);
onset_curve_length=length(onset_curve);
offset_curve_length=length(offset_curve);

% for loops for k_fast, alpha, k, and dstar parameters if you are to try
% to sweep these parameters
% for k_fast = 0.1:2:2
% for alpha = 0.1:2:2
% for k = 0.0005:0.001:0.001
for on=1:20:onset_curve_length
for off=1:50:offset_curve_length
%tmax = 2*(2*pi/(k))/tstep;
tmax = 12000;
k = 0.005;
k_fast = 1;
alpha = 1;
if flag == 2 || flag == 3
p1 = onset_curve(:,on);
p2 = offset_curve(:,off);
p3 = offset_curve2;
else
p1 = onset_curve(:,on);
p2 = offset_curve2(:,off);
p3 = offset_curve;
end


tspan = 0:tstep:tmax;
```

```
% Create circular path based 3 defining points
[E, F, C, r] = Parametrization_3PointsCircle(p1',p2',p3');


N_t = length(tspan);
X = zeros(3,N_t);
xx = x0;

sigma = f(on, off);
Rn = [pinknoise([1,N_t],-1, sigma);pinknoise([1,N_t],-1, 00);pinknoise([1,N_t],-1, 00)];
mu2_big = zeros(1, length(N_t));
mu1_big = zeros(1, length(N_t));
nu_big = zeros(1, length(N_t));

for n = 1:N_t
    %Euler-Meruyama method
    [Fxx, mu2, mu1,nu] = SlowWave_Model(tspan(n),xx,b,k,k_fast, alpha, E,F,C,r);
    xx = xx + tstep*Fxx + sqrt(tstep)*Rn(:,n);
    X(:,n) = xx;
     mu2_big(n) = mu2;
    mu1_big(n) = mu1;
    nu_big(n) = nu;


end

x = X';
% Ensure p1, p2, p3 are column vectors (if not already)
p1 = p1(:);
p2 = p2(:);
p3 = p3(:);
mu1_big = -mu1_big;

%get onset/offset points
tol = 1e-5/2;  % Tolerance for floating-point comparison


    onset_matches = [];
    offset_matches = [];


    for i = 1:size(onset_curve, 2)

        current_point = onset_curve(:, i);


        idx = find( ...
            abs(mu2_big(:) - current_point(1)) < tol & ...
            abs(mu1_big(:) - current_point(2)) < tol & ...
            abs(nu_big(:) - current_point(3)) < tol ...
        );
```

```
        onset_matches = [onset_matches; idx];
    end


    onset_idx = unique(onset_matches);


    if flag == 1
    for i = 1:size(offset_curve, 2)
        current_point = offset_curve(:, i);
        idx = find( ...
            abs(mu2_big(:) - current_point(1)) < tol & ...
            abs(mu1_big(:) - current_point(2)) < tol & ...
            abs(nu_big(:) - current_point(3)) < tol ...
        );
        offset_matches = [offset_matches; idx];
    end
    else
    for i = 1:size(offset_curve2, 2)
        current_point = offset_curve2(:, i);
        idx = find( ...
            abs(mu2_big(:) - current_point(1)) < tol & ...
            abs(mu1_big(:) - current_point(2)) < tol & ...
            abs(nu_big(:) - current_point(3)) < tol ...
        );
        offset_matches = [offset_matches; idx];
    end
    end
    offset_idx = unique(offset_matches);

if(onset_idx(1) < offset_idx(1))
seizure = x(1:onset_idx(2));
seizures{end+1} = {seizure, onset_idx(1), offset_idx(1)};
else
    seizure = x(offset_idx(1):onset_idx(2));
    seizures{end+1} = {seizure, onset_idx(1)-offset_idx(1), offset_idx(2)-offset_idx(1)};
end

end
end


% end
% end
% end
```

Piecewise generator

```
clear all
```

```
seizures = {};

x0=[0;0;0];

% Settings - Model
% focus
b = 1.0;

% radius of the sphere, do not change
R = 0.4;

%Integration step/Sampling rate of the simulation
tstep = 0.01;

%%class wanted to run, input 3,7,9,10,11
%this corresponds to '3 - SN/Sup', '7 - SNIC/Sup', '9- Sup/SNIC', '10 -Sup/SH',
% '11 - Sup/Sup'
class = 11;

[p0,onset_curve,p1_5,offset_curve,p3]=piecewise_random_path(class);

onset_curve_length=length(onset_curve);
offset_curve_length=length(offset_curve);


%for k_fast = 0.1:0.1:2
%for alpha = 0.1:0.1:2
%for k = 0.00001:0.00001:0.001
for on=1:50:onset_curve_length
for off=1:50:offset_curve_length
%for noise= 0:100:3000

noise = 600;
k = 0.0001;
k_fast = 1;
alpha = 1;


 p1 = onset_curve(on, :);
p2 = offset_curve(off,:);


stall_val = 30000;
[mu2_straight_path0,mu1_straight_path0,nu_straight_path0,rad1] = sphereArcPath(k,tstep,p0,p1);
[mu2_straight_path0_5,mu1_straight_path0_5,nu_straight_path0_5,rad2] =
sphereArcPath(k,tstep,p1,p1_5);
points = repmat(p1_5, stall_val, 1)';
%path noise sigma
sigma = 100;
Rn = [pinknoise([1,length(points)],-1, sigma);pinknoise([1,length(points)],-1,
sigma);pinknoise([1,length(points)],-1, sigma)];
points = points + Rn;
```

```
[mu2_straight_path,mu1_straight_path,nu_straight_path,rad3] = sphereArcPath(k,tstep,p1_5,p2);
[mu2_straight_path1,mu1_straight_path1,nu_straight_path1,rad4] = sphereArcPath(k,tstep,p2,p3);
mu2_all = [mu2_straight_path0, mu2_straight_path0_5, points(1, :), mu2_straight_path,
mu2_straight_path1];
mu1_all = [mu1_straight_path0, mu1_straight_path0_5, points(2, :), mu1_straight_path,
mu1_straight_path1];
mu1_all = -mu1_all;
nu_all = [nu_straight_path0, nu_straight_path0_5, points(3,:), nu_straight_path,
nu_straight_path1];

N_t = length(mu2_all);
X = zeros(3,N_t);
xx = x0;
%signal pink noise sigma
sigma = noise;
Rn = [pinknoise([1,N_t],-1, sigma);pinknoise([1,N_t],-1, 00);pinknoise([1,N_t],-1, 00)];
mu2_big = zeros(1, length(N_t));
mu1_big = zeros(1, length(N_t));
nu_big = zeros(1, length(N_t));

%%get onset index by finding Radians to bifurcation, and getting index
%%through k and tstep parameters
onset_index = floor((rad1/k)/tstep);
offset_index = floor(((rad1+rad2+rad3)/k)/tstep) + stall_val;
for n = 1:N_t
    %%Euler-Meruyama method
    [Fxx,mu2,mu1,nu] = SlowWave_Model_piecewise(0,xx,b,k,k_fast, alpha, mu2_all(n),
mu1_all(n),nu_all(n));
    xx = xx + tstep*Fxx + sqrt(tstep)*Rn(:,n);
    X(:,n) = xx;
    mu2_big(n) = mu2;
    mu1_big(n) = mu1;
    nu_big(n) = nu;
end
x = X';
seizure = x(:,1);
seizures{end+1} = {seizure, onset_index, offset_index};
end
end
% end
% end
% end
```

Post-processing

```
post_processed_seizures= {};
for seizure = 1:length(seizures)
data = seizures{seizure}{1};
[pks,locs] = findpeaks(data, 'MinPeakProminence', 0.10);
fs = 1/(0.01*tstep);
```

```
t = (0:length(data)-1) / fs;

%%Getting average spike rate
time_in_seconds = locs / fs; % Convert peak indices to seconds
spike_rates = diff(time_in_seconds);
average_frequency = mean(spike_rates);

if average_frequency < 1 || average_frequency > 30
% Calculate spike rates
spike_rates = diff(time_in_seconds);
% Adjust spike rates to achieve a mean average spiking rate of 5 Hz
target_avg_spike_rate = (1/10); % Hz

% Calculate the current average spiking rate
current_avg_spike_rate = mean(spike_rates);

% Calculate the adjustment factor
adjustment_factor = target_avg_spike_rate / current_avg_spike_rate;

% Adjust spike rates
adjusted_spike_rates = spike_rates * adjustment_factor;

% Calculate the mean average spiking rate after adjustment
mean_avg_spike_rate = mean(adjusted_spike_rates);

% Calculate the new sampling frequency
new_sampling_frequency = fs / adjustment_factor;


end





HPF = designfilt('highpassiir', ...        % Response type
       'FilterOrder',1, ...     % Filter Order Specification
       'HalfPowerFrequency',0.1, ...
       'DesignMethod','butter', ...      % Design method
       'SampleRate',new_sampling_frequency);               % Sample rate
%add pink noise over

data = filter(HPF, data);
min_val = min(data);
max_val = max(data);
data = (data - min_val) / (max_val - min_val);
rms_signal = get_amp(data, new_sampling_frequency);
normalized_data = data;
noisy_data_20 = add_pink_noise(normalized_data, rms_signal, 0.2, new_sampling_frequency);


post_processed_seizures{end+1} = {noisy_data_20, seizures{seizure}{2}, seizures{seizure}{3}};
end
```

Visualize Seizures

```
% Select seizure to plot
idx = 1; % choose seizure number

signal = post_processed_seizures{idx}{1};  % processed signal
onset  = post_processed_seizures{idx}{2};  % onset index
offset = post_processed_seizures{idx}{3};  % offset index



figure;
plot(signal, 'k', 'LineWidth', 1.2);
hold on;
xline(onset, '--g', 'Onset', 'LineWidth', 1.2);
xline(offset, '--r', 'Offset', 'LineWidth', 1.2);
ylabel('Normalized Amplitude');
title(sprintf('Processed Seizure #%d', idx));
grid on;
```

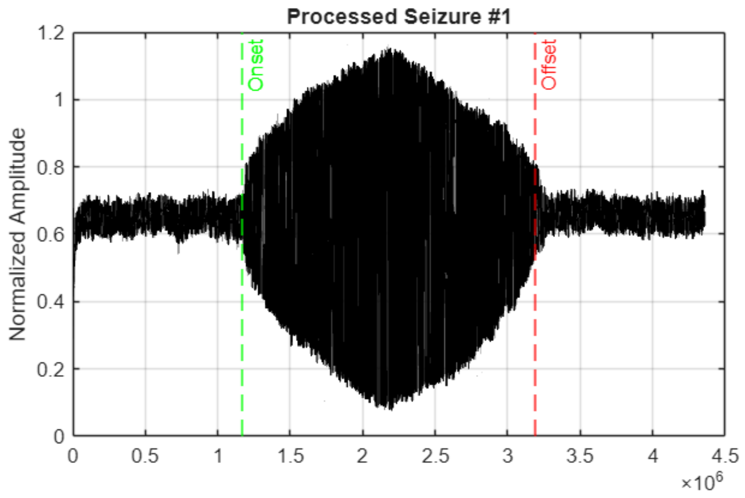

Functions

```
function x_rs = Resting_State(mu2,mu1,nu,N)

    switch N
        case 1 % resting state on upper branch
            x_rs=mu2/(3*(mu1/2 + (mu1^2/4 - mu2^3/27)^(1/2))^(1/3)) + (mu1/2 + (mu1^2/4 - 
mu2^3/27)^(1/2))^(1/3);

        case 2 % resting state on lower branch
            x_rs=- mu2/(6*(mu1/2 + (mu1^2/4 - mu2^3/27)^(1/2))^(1/3)) - (mu1/2 + (mu1^2/4 - 
mu2^3/27)^(1/2))^(1/3)/2 - (3^(1/2)*i*(mu2/(3*(mu1/2 + (mu1^2/4 - mu2^3/27)^(1/2))^(1/3)) - 
(mu1/2 + (mu1^2/4 - mu2^3/27)^(1/2))^(1/3)))/2;
```

```
        case 3
            x_rs= (3^(1/2)*i*(mu2/(3*(mu1/2 + (mu1^2/4 - mu2^3/27)^(1/2))^(1/3)) - (mu1/2 +
(mu1^2/4 - mu2^3/27)^(1/2))^(1/3)))/2 - (mu1/2 + (mu1^2/4 - mu2^3/27)^(1/2))^(1/3)/2 -
mu2/(6*(mu1/2 + (mu1^2/4 - mu2^3/27)^(1/2))^(1/3));
    end

end

function x_rs=eval_resting_state_cartesian(a,mu2,mu1,N)

switch N
    case 1 % resting state
        x_rs = ((a.^3 .* mu1)./2 + ((a.^6 .* mu1.^2)./4 - (a.^6 .* mu2.^3)./27).^(1/2)).^(1/3)
+ ...
       (a.^2 .* mu2) ./ (3 .* ((a.^3 .* mu1)./2 + ((a.^6 .* mu1.^2)./4 - (a.^6 .*
mu2.^3)./27).^(1/2)).^(1/3));

    case 2
        x_rs = - (sqrt(3) .* (((a.^3 .* mu1)./2 + ((a.^6 .* mu1.^2)./4 - (a.^6 .*
mu2.^3)./27).^(1/2)).^(1/3) - (a.^2 .* mu2) ./ (3 .* ((a.^3 .* mu1)./2 + ((a.^6 .* mu1.^2)./4 -
(a.^6 .* mu2.^3)./27).^(1/2)).^(1/3))) .* 1i) ./ 2 ...
      - ((a.^3 .* mu1)./2 + ((a.^6 .* mu1.^2)./4 - (a.^6 .* mu2.^3)./27).^(1/2)).^(1/3) ./ 2
...
      - (a.^2 .* mu2) ./ (6 .* ((a.^3 .* mu1)./2 + ((a.^6 .* mu1.^2)./4 - (a.^6 .*
mu2.^3)./27).^(1/2)).^(1/3));

    case 3
        x_rs = (sqrt(3) .* (((a.^3 .* mu1)./2 + ((a.^6 .* mu1.^2)./4 - (a.^6 .*
mu2.^3)./27).^(1/2)).^(1/3) - (a.^2 .* mu2) ./ (3 .* ((a.^3 .* mu1)./2 + ((a.^6 .* mu1.^2)./4 -
(a.^6 .* mu2.^3)./27).^(1/2)).^(1/3))) .* 1i) ./ 2 ...
      - ((a.^3 .* mu1)./2 + ((a.^6 .* mu1.^2)./4 - (a.^6 .* mu2.^3)./27).^(1/2)).^(1/3) ./ 2
...
      - (a.^2 .* mu2) ./ (6 .* ((a.^3 .* mu1)./2 + ((a.^6 .* mu1.^2)./4 - (a.^6 .*
mu2.^3)./27).^(1/2)).^(1/3));

end

end


function [E,F] = Parametrization_2PointsArc(A,B,R)

    E = A./R;

    F=cross(cross(A,B),A);
    F=F./norm(F);

end
function x = pinknoise(DIM,BETA, MAG)
%% the function pinknoise(dimension,beta,amplitude) can be used to modify noise
% parameters. The beta parameter is the slope of 1/f^beta. Beta = 0 is white
```

```
 %noise, beta = -1 is pink, and -2 is brownian. This parameter can be changed to fit the slope
of
 %desired noise (typically between -0.5 and 1.5), as demonstrated in Suppl Fig. 7 in Jirsa et
al. Brain 2014.
 %Magnitude can be modified to fit the desired noisiness of the data

 % This function generates 1/f spatial noise, with a normal error
 % distribution
 %
 % DIM is a two component vector that sets the size of the spatial pattern
 % (DIM=[10,5] is a 10x5 spatial grid)
 %
 % BETA defines the spectral distribution.
 % Spectral density S(f) = N f^BETA
 % (f is the frequency, N is normalisation coeff).
 % BETA = 0 is random white noise.
 % BETA -1 is pink noise
 % BETA = -2 is Brownian noise
 % The fractal dimension is related to BETA by, D = (6+BETA)/2
 %
 % MAG is the scaling variable for the noise amplitude
 %
 % The method is briefly descirbed in Lennon, J.L. "Red-shifts and red
 % herrings in geographical ecology", Ecography, Vol. 23, p101-113 (2000)
 u = [(0:floor(DIM(1)/2)) -(ceil(DIM(1)/2)-1:-1:1)]'/DIM(1);
 u = repmat(u,1,DIM(2));
 v = [(0:floor(DIM(2)/2)) -(ceil(DIM(2)/2)-1:-1:1)]/DIM(2);
 v = repmat(v,DIM(1),1);
 S_f = (u.^2 + v.^2).^(BETA/2);
 S_f(S_f==inf) = 0;
 phi = rand(DIM);
 y= S_f.^0.5 .* (cos(2*pi*phi)+i*sin(2*pi*phi));
 y=y.*MAG/max(abs(y)); %set the mag to the level you want
 x= ifft2(y);
 x = real(x);
 end
 function Xdot = HysteresisLoop_Model(~,x,~,k,k_fast, alpha, R,dstar,E,F,N)

     % Parametrization of the path in the spherical parameter space in terms of great
     % circles
     mu2=R*(E(1)*cos(x(3))+F(1)*sin(x(3)));
     mu1=-R*(E(2)*cos(x(3))+F(2)*sin(x(3)));
     nu=R*(E(3)*cos(x(3))+F(3)*sin(x(3)));

     % x coordinate of resting state (i.e. upper branch of eq)
      x_rs=real(Resting_State(mu2,mu1,nu, N));
      %use this to integrate changes in alpha with the resting state
      %x_rs=real(eval_resting_state_cartesian(alpha,mu2,mu1,N));

     % equations
     xdot = -k_fast* alpha*x(2);
```

```
    ydot = -k_fast*(-(x(1)/alpha)^3 +mu2*(x(1)/alpha) +mu1 + x(2)*( nu + (x(1)/alpha) +
(x(1)/alpha)^2));
    zdot =  -k*(sqrt(((x(1)/alpha)-x_rs)^2+x(2)^2)-dstar);

    Xdot = [xdot;ydot;zdot];
end

function [E, F, C, r] = Parametrization_3PointsCircle(p1, p2, p3)
    % Calculate unit direction vectors
    p1 = p1';
    p2 = p2';
    p3 = p3';
    V12 = (p1 - p2) / norm(p1 - p2);
    V13 = (p1 - p3) / norm(p1 - p3);

    % Compute the normal vector to the plane defined by the points
    n = cross(V12, V13);
    n = n / norm(n); % Normalize the normal vector

    % Calculate the coefficients for the plane equations
    dalpha = dot(p1, n);
    dbeta = dot(V12, p1 + (p2 - p1) / 2);
    dgamma = dot(V13, p1 + (p3 - p1) / 2);

    % Set up the linear equations to find the center C
    A = [n(1), n(2), n(3);
         V12(1), V12(2), V12(3);
         V13(1), V13(2), V13(3)];

    b = [dalpha; dbeta; dgamma];

    % Solve for C using least squares
    C = A\b;

    % Calculate E (unit vector from C to p1)
    E = (p1 - C) / norm(p1 - C);

    % Calculate F (perpendicular vector)
    F = -cross(E, n);

    % Calculate the radius r
    r = norm(p1 - C);
end

function [Xdot, mu2, mu1,nu] = SlowWave_Model(~,x,~,k,k_fast,alpha, E,F,C,r)

    % Parametrization of the path in the spherical parameter space in terms
    % of a circle defined by 3 points
    mu2=C(1)+r*(E(1)*cos(x(3))+F(1)*sin(x(3)));
    mu1=-(C(2)+r*(E(2)*cos(x(3))+F(2)*sin(x(3))));
    nu=C(3)+r*(E(3)*cos(x(3))+F(3)*sin(x(3)));
```

```
    % System
    xdot = -k_fast* alpha*x(2);
    ydot = -k_fast*(-(x(1)/alpha)^3 +mu2*(x(1)/alpha) +mu1 + x(2)*( nu + (x(1)/alpha) +
(x(1)/alpha)^2));
    zdot = k;



    Xdot = [xdot;ydot;zdot];
end

function get_plot()
    marker_size = 10;
    load('curves.mat')
    load('curves2.mat')
    load('bifurcation_crossing.mat')
    load('sphere_mesh.mat')
    load('testmesh.mat');

    hold on;
    linewidth = 2;

    % Plot different meshes with assigned DisplayName for the legend
    vertices = BCSmesh.vertices;
    faces = BCSmesh.faces;
    h1 = patch('Vertices', vertices, 'Faces', faces, ...
          'FaceColor', [0.973, 0.965, 0.722], 'EdgeColor', 'none', 'FaceAlpha', 0.6,
'DisplayName', 'BCS Mesh');

    vertices = Active_restmesh.vertices;
    faces = Active_restmesh.faces;
    h2 = patch('Vertices', vertices, 'Faces', faces, ...
          'FaceColor', [0.9216, 0.9216, 0.9216], 'EdgeColor', 'none', 'FaceAlpha', 0.6,
'DisplayName', 'Active Rest Mesh');

    vertices = Seizure_mesh.vertices;
    faces = Seizure_mesh.faces;
    h3 = trisurf(faces, vertices(:,1), vertices(:,2), vertices(:,3), ...
            'FaceColor', [0.894, 0.706, 0.831], 'EdgeColor', 'none', 'FaceAlpha', 0.3,
'DisplayName', 'Seizure Mesh');

    vertices = Bistable_Lcb_mesh.vertices;
    faces = Bistable_Lcb_mesh.faces;
    h4 = patch('Vertices', vertices, 'Faces', faces, ...
          'FaceColor', [0.973, 0.965, 0.722], 'EdgeColor', 'none', 'FaceAlpha', 0.6,
'DisplayName', 'Bistable Lcb Mesh');

    scale_array = [0.4];
    scale_array = scale_array / 0.4;

    % Arrays to store plot handles for the legend
    legend_handles = [h1, h2, h3];
```

```
    legend_names = {'Rest/ Seizure Bistable region', 'Active Rest/Rest Bistable region',
'Seizure region'};

    % Scale factors to adjust radius from 0.4
    for i = 1:length(scale_array)
        % Scale the coordinates of the points for radius 0.39
        Fold_of_cycles_scaled = scale_array(i) * Fold_of_cycles;
        Homoclinic_to_saddle3_scaled = scale_array(i) * Homoclinic_to_saddle3;
        Homoclinic_to_saddle2_scaled = scale_array(i) * Homoclinic_to_saddle2;
        Homoclinic_to_saddle1_scaled = scale_array(i) * Homoclinic_to_saddle1;
        Homoclinic_to_saddle_scaled = scale_array(i) * Homoclinic_to_saddle;
        Fold_scaled = scale_array(i) * Fold;
        Hopf_scaled = scale_array(i) * Hopf;
        SNIC_scaled = scale_array(i) * SNIC;

        % Plot all scaled lines with DisplayName for the legend
        h5 = plot3(Fold_of_cycles_scaled(1, :), Fold_of_cycles_scaled(2, :),
Fold_of_cycles_scaled(3, :), 'Color', [0.9725,0.2667,0.5843], 'LineWidth', linewidth,
'DisplayName', 'Fold of Cycles');
        h6 = plot3(Homoclinic_to_saddle3_scaled(1, :), Homoclinic_to_saddle3_scaled(2, :),
Homoclinic_to_saddle3_scaled(3, :), 'Color', [0.404, 0.702, 0.851], 'LineWidth', linewidth,
'LineStyle', '--', 'DisplayName', 'Homoclinic to Saddle 3');
        h7 = plot3(Homoclinic_to_saddle2_scaled(1, :), Homoclinic_to_saddle2_scaled(2, :),
Homoclinic_to_saddle2_scaled(3, :), 'Color', [0.404, 0.702, 0.851],  'LineWidth', linewidth,
'DisplayName', 'Homoclinic to Saddle 2');
        h8 = plot3(Homoclinic_to_saddle1_scaled(1, :), Homoclinic_to_saddle1_scaled(2, :),
Homoclinic_to_saddle1_scaled(3, :), 'Color', [0.404, 0.702, 0.851],  'LineWidth', linewidth,
'LineStyle', '--', 'DisplayName', 'Homoclinic to Saddle 1');
        h9 = plot3(Homoclinic_to_saddle_scaled(1, :), Homoclinic_to_saddle_scaled(2, :),
Homoclinic_to_saddle_scaled(3, :), 'Color', [0.404, 0.702, 0.851],  'LineWidth', linewidth,
'DisplayName', 'Homoclinic to Saddle');
        h10 = plot3(Fold_scaled(1, 140:564), Fold_scaled(2, 140:564), Fold_scaled(3, 140:564),
'Color', [0.957, 0.612, 0.204], 'LineWidth', linewidth, 'DisplayName', 'Fold Part 1');
        h11 = plot3(Fold_scaled(1, 575:end), Fold_scaled(2, 575:end), Fold_scaled(3, 575:end),
'Color', [0.957, 0.612, 0.204], 'LineWidth', linewidth, 'DisplayName', 'Fold Part 2');
        h12 = plot3(Fold_scaled(1, 1:80), Fold_scaled(2, 1:80), Fold_scaled(3, 1:80), 'Color',
[0.957, 0.612, 0.204], 'LineWidth', linewidth, 'DisplayName', 'Fold Part 3');
        h13 = plot3(Hopf_scaled(1, 1:400), Hopf_scaled(2, 1:400), Hopf_scaled(3, 1:400),
'Color',[0.4549 ,0.7490 ,0.2706], 'LineWidth', linewidth, 'LineStyle', '--', 'DisplayName',
'Hopf Part 1');
        h14 = plot3(Hopf_scaled(1, 400:973), Hopf_scaled(2, 400:973), Hopf_scaled(3, 400:973),
'Color',[0.4549 ,0.7490 ,0.2706], 'LineWidth', linewidth, 'DisplayName', 'Hopf Part 2');
        h15 = plot3(SNIC_scaled(1, :), SNIC_scaled(2, :), SNIC_scaled(3, :), 'Color',[0.957,
0.612, 0.204], 'LineWidth', linewidth, 'LineStyle', '--', 'DisplayName', 'SNIC');

        % Add line handles to the legend array
        legend_handles = [legend_handles, h5,   h9,  h12, h13, h14, h15];
        legend_names = [legend_names, 'Fold Limit Cycle', 'SH', 'SN', 'SubH', 'SupH', 'SNIC'];
    end

    % Add the sphere mesh with transparency
```

```
    surf(X_sphere, Y_sphere, Z_sphere, 'FaceColor', [0.96, 0.96, 0.86], 'FaceAlpha', 0.2,
'EdgeColor', 'none', 'HandleVisibility', 'off');

    % Add labels and view adjustment
    xlabel('\mu_2');
    ylabel('-\mu_1');
    zlabel('\nu');

    lineVector = [-0.19, 0.2, 0.07];
    az = atan2d(lineVector(2), lineVector(1)); % Azimuth angle
    el = atan2d(lineVector(3), norm(lineVector(1:2))); % Elevation angle
    view(az, el);

    % Display the legend with all handles and names
    legend(legend_handles, legend_names);
end


function   [onset_curve,offset_curve]=hysteresis_random_path(bifurcation)


load('curves.mat');


if bifurcation == '2s'
    onset_curve=SNr_LCs;
    offset_curve=SHl;

end


if bifurcation=='2b'
    onset_curve=SNr_LCb;
    offset_curve=SHb;

end



if bifurcation=='3s'
    onset_curve=SNr_LCs;
    offset_curve=SNl_ActiveRest;
end



if bifurcation=='4b'
    onset_curve=SNr_LCb;
    offset_curve=FLC_top;
end

if bifurcation=='10'
```

```
    onset_curve=SNr_ActiveRest;
    offset_curve=SHl;
end


if bifurcation=='11'


    onset_curve= SNr_ActiveRest;
    offset_curve=[[0.3171; -0.066; 0.2347], [0.3115; -0.0546; 0.2450],[0.3166; -0.0654;
0.2356]];%SNl_ActiveRest;
end

if bifurcation=='14'
    onset_curve=subH;
    offset_curve=SHb;
end


if bifurcation=='16'
    onset_curve=subH;
    offset_curve=FLC;
end













end


function [onset_curve,offset_curve,offset_curve2, flag] = slow_wave_circular_random_path(I)


load('curves2.mat');


if I == 1
    onset_curve = SHl(:,50:104); %55
    offset_curve = [0.33, 0.11, 0.18]';
    offset_curve2 = SNIC(:,1:35); %35
    flag = 1;
end
```

```
if I == 5
    onset_curve = SNIC; %44
    offset_curve = SNIC; %44
    offset_curve2 = [0.34,0.14,0.06]';
    flag = 2;
end


if I == 6
    onset_curve = SNIC(:,1:35); %35
    offset_curve = [0.33, 0.11, 0.18]';
    offset_curve2 = SHl(:,50:104); %55
    flag = 1;
end


if I == 7
    onset_curve=SNIC; %44
    offset_curve = Hopf(:,800:855); %56
    offset_curve2 = [0.36,-0.12,0.12]';
    flag = 3;
end


if I == 8
    onset_curve=SNIC; %44
    offset_curve = [0.34,0.2,-0.06]';
    offset_curve2 = FLC(:,100:300); %201
    flag = 1;
end


if I == 9
    onset_curve= Hopf(:,800:855); %56
    offset_curve = SNIC; %44
    offset_curve2 = [0.36,-0.12,0.12]';
    flag = 3;
end


if I == 12
    onset_curve= Hopf(:,450:495);
    offset_curve = FLC(:,60:100);
    offset_curve2 = [-0.3, -0.2, -0.2]';
    flag = 3;
end


if I == 13
    onset_curve = FLC(:,100:300); %201
    offset_curve = [0.34,0.2,-0.06]';
```

```
    offset_curve2 = SNIC;
    flag = 1;
end


if I == 15
    onset_curve= FLC(:,60:100);%41
    offset_curve = Hopf(:,450:495);%46
    offset_curve2 = [-0.3, -0.2, -0.2]';
    flag = 3;
end






end
function [Xdot, mu2,mu1,nu] = SlowWave_Model_piecewise(~,x,~,k,k_fast, alpha, mu2,mu1,nu)
% Parametrization of the path in the spherical parameter space in terms
% of a circle defined by 3 points
% System
xdot = -k_fast* alpha*x(2);
ydot = -k_fast*(-(x(1)/alpha)^3 +mu2*(x(1)/alpha) +mu1 + x(2)*( nu + (x(1)/alpha) +
(x(1)/alpha)^2));
zdot = k;
Xdot = [xdot;ydot;zdot];
end
function [mu2,mu1,nu, theta] = sphereArcPath(k, tstep,point1, point2)
% sphereArcPath - Generates an arc path between two points on a sphere
%
% Syntax: arcPath = sphereArcPath(point1, point2, numPoints)
%
% Inputs:
% point1 - [x1, y1, z1] Coordinates of the first point on the sphere
% point2 - [x2, y2, z2] Coordinates of the second point on the sphere
% numPoints - Number of points along the arc
%
% Outputs:
% arcPath - An Nx3 matrix containing the coordinates of points along the arc
% Check the input points
radius = 0.4;
% if norm(point1) ~= radius || norm(point2) ~= radius
% error('The points must lie on the sphere of radius 0.4.');
% end
% Normalize the input points to make sure they are on the sphere
point1 = point1 / norm(point1) * radius;
point2 = point2 / norm(point2) * radius;
% Compute the quaternion for rotation
theta = acos(dot(point1, point2) / (radius^2));
axis = cross(point1, point2);
if norm(axis) == 0
error('The points are the same or antipodal.');
```

```
end
axis = axis / norm(axis);
% Compute points along the arc
numPoints = floor((theta/k)/tstep);
arcPath = zeros(numPoints, 3);
for i = 0:numPoints-1
t = i / (numPoints - 1);
angle = t * theta;
R = rotationMatrix(axis, angle);
arcPath(i+1, :) = (R * point1')';
end
mu2 = arcPath(:,1)';
mu1 = arcPath(:,2)';
nu = arcPath(:,3)';
end
function R = rotationMatrix(axis, angle)
% rotationMatrix - Generates a rotation matrix given an axis and an angle
%
% Syntax: R = rotationMatrix(axis, angle)
%
% Inputs:
% axis - A 3-element vector representing the axis of rotation
% angle - The angle of rotation in radians
%
% Outputs:
% R - A 3x3 rotation matrix
ux = axis(1);
uy = axis(2);
uz = axis(3);
c = cos(angle);
s = sin(angle);
t = 1 - c;
R = [t*ux*ux + c, t*ux*uy - s*uz, t*ux*uz + s*uy;
t*ux*uy + s*uz, t*uy*uy + c, t*uy*uz - s*ux;
t*ux*uz - s*uy, t*uy*uz + s*ux, t*uz*uz + c];
end
function point= get_random_point
radius = 0.4;
% Generate two random numbers
theta = 2 * pi * rand(); % Random angle between 0 and 2*pi
phi = acos(2 * rand() - 1); % Random angle between 0 and pi
% Convert spherical coordinates to Cartesian coordinates
x = radius * sin(phi) * cos(theta);
y = radius * sin(phi) * sin(theta);
z = radius * cos(phi);
point = [x,y,z];
% Display the point
end
function point= get_random_point_hopf
%load("map_regions.mat")
radius = 0.4;
% Loop until a valid point with y > 0 is found
```

```
while true
% Generate two random numbers
theta = 2 * pi * rand(); % Random angle between 0 and 2*pi
phi = acos(2 * rand() - 1); % Random angle between 0 and pi
% Convert spherical coordinates to Cartesian coordinates
x = radius * sin(phi) * cos(theta);
y = radius * sin(phi) * sin(theta);
z = radius * cos(phi);
% Check if y is positive
if y > 0
point = [x, y, z];
break;
end
end
% Display the point
end
%


% Display the point
function point= get_random_point_fixed
radius = 0.4;
% Loop until a valid point with y > 0 is found
while true
% Generate two random numbers
theta = 2 * pi * rand(); % Random angle between 0 and 2*pi
phi = acos(2 * rand() - 1); % Random angle between 0 and pi
% Convert spherical coordinates to Cartesian coordinates
x = radius * sin(phi) * cos(theta);
y = radius * sin(phi) * sin(theta);
z = radius * cos(phi);
% Check if y is positive
if y < 0
point = [x, y, z];
break;
end
end
% Display the point
end


function [mu2,mu1,nu] = sphere(point1, point2, numPoints)
% sphereArcPath - Generates an arc path between two points on a sphere
%
% Syntax: arcPath = sphereArcPath(point1, point2, numPoints)
%
% Inputs:
% point1 - [x1, y1, z1] Coordinates of the first point on the sphere
% point2 - [x2, y2, z2] Coordinates of the second point on the sphere
% numPoints - Number of points along the arc
%
% Outputs:
```

```
% arcPath - An Nx3 matrix containing the coordinates of points along the arc
% Check the input points
radius = 0.4;
% if norm(point1) ~= radius || norm(point2) ~= radius
% error('The points must lie on the sphere of radius 0.4.');
% end
% Normalize the input points to make sure they are on the sphere
point1 = point1 / norm(point1) * radius;
point2 = point2 / norm(point2) * radius;
% Compute the quaternion for rotation
theta = acos(dot(point1, point2) / (radius^2));
axis = cross(point1, point2);
if norm(axis) == 0
error('The points are the same or antipodal.');
end
axis = axis / norm(axis);
% Compute points along the arc
arcPath = zeros(numPoints, 3);
for i = 0:numPoints-1
t = i / (numPoints - 1);
angle = t * theta;
R = rotationMatrix(axis, angle);
arcPath(i+1, :) = (R * point1')';
end
mu2 = arcPath(:,1)';
mu1 = arcPath(:,2)';
nu = arcPath(:,3)';
end

function [arcLength, theta] = calculateArcLength(P1, P2, radius)
 % calculateArcLength computes the arc length and central angle between two points on a
sphere.
 %
 % Input:
 % P1 - First point [x1, y1, z1]
 % P2 - Second point [x2, y2, z2]
 % radius - Radius of the sphere (default: 0.4 if not provided)
 %
 % Output:
 % arcLength - Arc length between the two points
 % theta - Central angle between the two points in radians

 if nargin < 3
 radius = 0.4;
 end

 % Compute the dot product of P1 and P2
 dotProduct = dot(P1, P2);

 % Compute the magnitudes of P1 and P2
 magnitudeP1 = norm(P1);
 magnitudeP2 = norm(P2);
```

```
 % Compute the cosine of the central angle
 cosTheta = dotProduct / (magnitudeP1 * magnitudeP2);

 % Compute the central angle in radians
 theta = acos(cosTheta);

 % Compute the arc length
 arcLength = radius * theta;
end



function   [p0,p1,p1_5,p2,p3]=piecewise_random_path(bifurcation)

load('curves.mat');
load('bifurcation_crossing.mat')
load("curves2.mat")

if bifurcation==3
    %fixed rest point
    p0 = Hopf(:,930)';
    %bifurcation curve
    randomNumber = randi([145,170]);
    p1 = Fold(:,145:170)';
    randomNumber2 = randi([600,750]);
   p1_5 = [0.3196, 0.2389, -0.0279];
    %bifurcation curve
    p2 = Hopf(:,600:750)' ;
    %fixed rest
    p3 = [ 0.1944 , 0.0893 , 0.3380];

end

if bifurcation==7
    randomNumber = randi([600,750]);
 randomNumber2 = randi([1,44]);
%fixed rest point
p0 = Fold(:,400)';
%bifurcation curve
p1 =  SNIC' ;
%random point in limit cycle
p1_5 = [0.1314, 0.3298, -0.1843];
%bifurcation curve
p2 = Hopf(:,600:750)';
%fixed rest
p3 = [ 0.1944 , 0.0893 , 0.3380];
end

if bifurcation==9
    randomNumber = randi([600,750]);
    %fixed rest point
```

```
    p0 = [ 0.1944 , 0.0893 , 0.3380];
    %bifurcation curve
    p1 = Hopf(:,600:750)';
    %change here
    randomNumber2 = randi([1,44]);
    %random point in limit cycle
    p1_5 = [-0.0441, 0.2591, -0.3015];
    %bifurcation curve
    p2 = SNIC' ;
    %fixed rest
    p3 = Fold(:,450)';
end

if bifurcation==10
    randomNumber = randi([600,750]);
    %fixed rest point
    p0 = [ 0.1944 , 0.0893 , 0.3380];
    %bifurcation curve
    p1 = Hopf(:,600:750)';%get_nearest_hopf(p0(1),p0(2),p0(3))';
    %change here
    randomNumber2 = randi([1,124]);
    %random point in limit cycle
    p1_5 = [-0.123686721647726      0.338825918756816  -0.172912092308889];
    %bifurcation curve
    p2 = Homoclinic_to_saddle' ;
    %fixed rest
    p3 = Fold(:,400)';
end

if bifurcation==11
     randomNumber = randi([600,750]);
%fixed rest point
p0 = [ 0.1944 , 0.0893 , 0.3380];
%bifurcation curve
p1 = Hopf(:,600:750)';
%change here
randomNumber2 = randi([600,750]);
%random point in limit cycle
p1_5 = [-0.2104, 0.3180, -0.1209];
%bifurcation curve
p2 = Hopf(:,600:750)' ;
%fixed rest
p3 = [ 0.1944 , 0.0893 , 0.3380];
end


end

function [new_X, new_Y, new_Z] = get_random_walk(p1,p2,region,k,tstep, minSteps, maxSteps)
load('logicgrid.mat')
% if region == 11
% load('logicgrid.mat'); % Load the logic grid from the .mat file
```

```
%
% elseif region == 'LCS_bistable'
%         load("logicgrid_bistable_lcs.mat")
%
% elseif region == 'LCB_bistable'
%         load("logicgrid_bistable_lcb.mat")
% end
grid = logic_grid;

[theta, phi, r] = cart2sph(p1(1), p1(2), p1(3));
theta_offset_deg = -40;  % For example, a 30-degree rotation

% Convert the offset to radians
theta_offset_rad = deg2rad(theta_offset_deg);
theta_deg_p1 = rad2deg(theta+theta_offset_rad);
phi_deg_p1 = rad2deg(phi);

p1_5 = get_nearest_seizure_point(theta_deg_p1, phi_deg_p1, region);
[theta, phi, r] = cart2sph(p2(1), p2(2), p2(3));
theta_offset_deg = -40;  % For example, a 30-degree rotation

% Convert the offset to radians
theta_offset_rad = deg2rad(theta_offset_deg);
theta_deg_p2 = rad2deg(theta+theta_offset_rad);
phi_deg_p2 = rad2deg(phi);
p2_5 = get_nearest_seizure_point(theta_deg_p2, phi_deg_p2, region);
% Load the grid (assuming the variable is 'grid')



% Define the start and end points
startPoint = p1_5; % Define the start point (row, column)
endPoint = p2_5; % Define the end point (row, column)

 currentPos = startPoint;
%
% % Store the path
 path = currentPos;
%
% % Define the movement directions (up, down, left, right)
directions = [0 1; 0 -1; 1 0; -1 0]; % [row_change, col_change]


% Get the size of the grid
[gridRows, gridCols] = size(grid);

% Loop until a valid path is found
% Loop until a valid path is found
while true
    % Initialize the current position at the start point
    currentPos = startPoint;
```

```
    % Store the path, starting from the initial position
    path = currentPos;
    stepCount = 0;

    % Perform the random walk until either endpoint is reached or maxSteps
    while stepCount < maxSteps
        % Check if the endpoint has been reached and steps are within bounds
        if isequal(currentPos, endPoint) && stepCount >= minSteps
            disp('Valid path found within the specified range of steps.');
            break;
        end

        % Choose a random direction to move
        randomDir = directions(randi(4), :);

        % Compute the new position
        newPos = currentPos + randomDir;

        % Check if the new position is within bounds and valid (e.g., grid value is 1)
        if newPos(1) > 0 && newPos(1) <= gridRows && newPos(2) > 0 && newPos(2) <= gridCols
            if grid(newPos(1), newPos(2)) == 1
                % Update the current position
                currentPos = newPos;

                % Add the new position to the path
                path = [path; currentPos];

                % Increment the step count
                stepCount = stepCount + 1;
            end
        end
    end

    % Check if a valid path was found within bounds
    if isequal(currentPos, endPoint) && stepCount >= minSteps && stepCount <= maxSteps
        % Exit the loop if a valid path is found
        break;
    end

    % If not, repeat the simulation
end
% Plot the random walk path
figure;
imagesc(grid); % Show the grid
colormap(gray); % Use a gray color map (1 = white, 0 = black)
hold on;
plot(path(:,2), path(:,1), 'r.-', 'LineWidth', 2, 'MarkerSize', 15); % Plot the path
% Plot the start point in green
plot(startPoint(2), startPoint(1), 'go', 'MarkerSize', 10, 'MarkerFaceColor', 'g');

% Plot the end point in blue
plot(endPoint(2), endPoint(1), 'bo', 'MarkerSize', 10, 'MarkerFaceColor', 'b');
```

```
title('2D Random Walk Path on the Logic Grid');
xlabel('X');
ylabel('Y');



 if region == 11
load('logicgrid.mat'); % Load the logic grid from the .mat file

elseif region == 'LCS_bistable'
        load("logicgrid_bistable_lcs.mat")

elseif region == 'LCB_bistable'
        load("logicgrid_bistable_lcb.mat")
end

    % Convert back to x, y coordinates
    x_back = (path(:,1) - 1) / (grid_size(1) - 1) * (x_max - x_min) + x_min;
    y_back = (path(:,2) - 1) / (grid_size(2) - 1) * (y_max - y_min) + y_min;






    theta_rad = deg2rad(x_back) + deg2rad(-theta_offset_deg);
phi_rad = deg2rad(y_back);
radius = 0.4; % Set the radius of the sphere


[X, Y, Z] = sph2cart(theta_rad, phi_rad, radius);

r = 0.4;
P1 = [X(1);Y(1);Z(1)];
P2 = [X(2);Y(2);Z(2)];
% Compute the angle between the two points using the dot product formula
cos_theta = dot(P1, P2) / (r^2);
theta = acos(cos_theta);  % Angle in radians

total_theta = (length(X)-1)*theta;

numPoints = floor((total_theta/k)/tstep);
% Create interpolation queries and interpolate X, Y, Z coordinates
xq = linspace(1, length(X), numPoints); % Query points for interpolation

new_X = interp1(1:length(X), X, xq, 'linear');
new_Y = interp1(1:length(Y), Y, xq, 'linear');
new_Z = interp1(1:length(Z), Z, xq, 'linear');




end
```

```
function nearestPoint= get_nearest_seizure_point(theta,phi, region)
load('logicgrid.mat'); % Load the logic grid from the .mat file
%  if region == 11
% load('logicgrid.mat'); % Load the logic grid from the .mat file
%
% elseif region == 'LCS_bistable'
%         load("logicgrid_bistable_lcs.mat")
%
% elseif region == 'LCB_bistable'
%         load("logicgrid_bistable_lcb.mat")
% end

x = [theta,phi];
[row, col] = find(logic_grid);

    x(1) = round((theta - x_min) / (x_max - x_min) * (grid_size(1) - 1)) + 1;
    x(2) = round((phi - y_min) / (y_max - y_min) * (grid_size(2) - 1)) + 1;
% Compute the Euclidean distance between x and each point in the grid
distances = sqrt((row - x(1)).^2 + (col - x(2)).^2);

% Find the index of the minimum distance
[~, minIndex] = min(distances);

% Get the coordinates of the nearest point
nearestPoint = [row(minIndex), col(minIndex)];
end

function noisy_signal = add_pink_noise(signal, rms_signal, noise_amplitude_ratio, fs)
    % Inputs:
    % signal - input signal (1D array)
    % noise_amplitude_ratio - fraction of signal amplitude for noise (e.g., 0.4 for 40%)

    % Compute the RMS amplitude of the signal


    % Generate pink noise of the same length as the signal
    % Pink noise can be generated using dsp.ColoredNoise in MATLAB
    L = length(signal);
    pink_noise = pinknoise([1,L],-1,10000)';



    %
    % % % Scale the noise so its amplitude is noise_amplitude_ratio of the signal's amplitude
    % scaling_factor = noise_amplitude_ratio * (1 / rms_noise);
    % scaled_noise = pink_noise * scaling_factor;
    min_val = min(pink_noise(:));
max_val = max(pink_noise(:));
scaled_noise = noise_amplitude_ratio*(pink_noise - min_val) / (max_val - min_val);
min_val = min(signal(:));
max_val = max(signal(:));
scaled_signal = (signal - min_val) / (max_val - min_val);
    % Add the scaled noise to the original signal
```

```
    noisy_signal = scaled_signal + scaled_noise;
end

function amp = get_amp(signal, fs)

[peaks,locs] = findpeaks(signal ,'MinPeakProminence', 0.15);
[troughs_neg,locs_troughs] = findpeaks(signal, 'MinPeakProminence', 0.15);
troughs = -1*troughs_neg;

newnew = [];
len = 0;
if length(troughs) > length(peaks)
    len = length(peaks);
else
    len = length(troughs);
end
for i = 1:len
    newnew = [newnew; abs(troughs(i) - peaks(i))];
end
amp = mean(newnew);
end

function [start_index, stop_index, signal] = bifurcation_all_class(class, tstep, sigma)
R = 1;
N = 1;
b = 0;
x0 = [0;0;0];
k =0.007;
dstar = 0.3;
tstep = 0.1;
tmax = 75000;
if ismember(class, [1 5 6 8 12 13 15])
    % Code for class 1
    % Perform specific operations for class 1
    [onset_curve,offset_curve,p3, flag] = slow_wave_circular_random_path(class);
    onset_curve_length=length(onset_curve);
    offset_curve_length=length(offset_curve);
    random_onset_index=randsample(onset_curve_length,1);
    random_offset_index=randsample(offset_curve_length,1);
    if flag == 2 || flag == 3

        p1 = onset_curve(:,random_onset_index);
        p2 = offset_curve(:,random_offset_index);
    else
        p1 = onset_curve(:,random_onset_index);
        p2 = p3(:,random_offset_index);
        p3 = offset_curve;
    end



% uncomment this code to do random path
```

```
% % One random path - select random point on onset curve and offset curve

tspan = 0:tstep:tmax;

% Create circular path based 3 defining points
[E, F, C, r] = Parametrization_3PointsCircle(p1',p2',p3');
if class == 13
    E = -E;
end


N_t = length(tspan);
X = zeros(3,N_t);
xx = x0;
sigma = 40;
Rn = [pinknoise([1,N_t],-1, sigma);pinknoise([1,N_t],-1, 00);pinknoise([1,N_t],-1, 00)];
mu2_big = zeros(1, length(N_t));
mu1_big = zeros(1, length(N_t));
nu_big = zeros(1, length(N_t));

for n = 1:N_t
    %Euler-Meruyama method
    [Fxx, mu2, mu1,nu] = SlowWave_Model(tspan(n),xx,b,k,E,F,C,r);
    xx = xx + tstep*Fxx + sqrt(tstep)*Rn(:,n);
    X(:,n) = xx;
     mu2_big(n) = mu2;
    mu1_big(n) = mu1;
    nu_big(n) = nu;


end

x = X';
t = tspan;

%%Onset and offset calculation, calculates radians to the bifurcation
%%curve, then uses tstep and k variables to compute onset location
        plot_onset_offset = 0;
        if(floor((((2*pi)/k)/tstep)) < N_t)
            plot_onset_offset = 1;
        point1 = p1' - C;
        point2 = p2' - C;
        point3 = p3'-C;
        point1 = point1 / norm(point1) * r;
        point2 = point2 / norm(point2) * r;

        point3 = point3 / norm(point3) * r;
        % Compute the quaternion for rotation
        theta1 = acos(dot(point1, point2) / (r^2));
        %%%change here
        numPoints1 = floor((theta1/k)/tstep);
        point = [mu2_big(numPoints1), -mu1_big(numPoints1), nu_big(numPoints1)];
```

```
        if round(point,2) == round(p2,2)'
        onset_index = numPoints1;
        else
        numPoints1 = floor(((2*pi - theta1)/k)/tstep);
        onset_index = numPoints1;
        theta1 = theta1-2*pi;
        end
        theta2 = acos(dot(point1, point3) / (r^2));
        numPoints2 = floor(((theta2)/k)/tstep);
        point = [mu2_big(numPoints2), -mu1_big(numPoints2), nu_big(numPoints2)];
        if round(point,2) == round(p2,2)'
        %offset_index = numPoints2;
        else
        numPoints2 = floor(((2*pi  - theta2)/k)/tstep);
        theta2 = 2*pi - theta2;
        offset_index = numPoints2;
        end
        theta3 = 2*pi;
        numPoints3 = floor(((theta3)/k)/tstep);
        point = [mu2_big(numPoints3), -mu1_big(numPoints3), nu_big(numPoints3)];
        offset_index = numPoints3;
        end
        if class == 15 || class == 12
             onset_index_temp = onset_index;
        onset_index = offset_index;
        offset_index = onset_index_temp + floor((((2*pi)/k)/tstep));
        end
        start_index = max(1,onset_index-1000);
        stop_index = min(offset_index+1000, length(x));
        signal = x(start_index:stop_index,1);


elseif ismember(class, ['2s' '2b' '4b' '14' '16'])
    k=0.01;
    tmax = 75000;
    dstar = 0.3;
   [onset_curve,offset_curve]=hysteresis_random_path(class);
   onset_curve_length=length(onset_curve);
offset_curve_length=length(offset_curve);



% uncomment this code to do random path
% % One random path - select random point on onset curve and offset curve
random_onset_index=randsample(onset_curve_length,1);
random_offset_index=randsample(offset_curve_length,1);
A = offset_curve(:,random_offset_index);
B = onset_curve(:,random_onset_index);
    tspan = 0:tstep:tmax;

% Create circular path based 3 defining points
[E, F] = Parametrization_2PointsArc(A,B,R);
```

```
N_t = length(tspan);
X = zeros(3,N_t);
xx = x0;


Rn = [pinknoise([1,N_t],-1, sigma);pinknoise([1,N_t],-1, 00);pinknoise([1,N_t],-1, 00)];

for n = 1:N_t

    % Euler-Meruyama method
    Fxx = HysteresisLoop_Model(tspan(n),xx,b,k,R,dstar,E,F,N);
    xx = xx + tstep*Fxx + sqrt(tstep)*Rn(:,n);
    X(:,n) = xx;

end

x = X';

[pks,times]=findpeaks(x(:,3),'MinPeakProminence',0.03);
onset_time = times*tstep;

% Calculate Offset Times
[pks2,times2]=findpeaks(-x(:,3),'MinPeakProminence',0.03);
offset_time = times2*tstep;


% Single seizure
if offset_time(1)>onset_time(1) % if system starts at rest
    start_index = times(1)-10000;
    stop_index = times2(1)+10000;
    start_index = max(1, start_index);
    stop_index = min(length(x), stop_index);
    signal = x(start_index:stop_index,1);
    onset = times(1);
    offset = stop_index-start_index-10000;
else % if system starts in a seizure
    start_index = times(1)-10000;
    stop_index = times2(2)+10000;
    start_index = max(1, start_index);
    stop_index = min(length(x), stop_index);
    signal = x(start_index:stop_index,1);
    onset = times(1);
    offset = stop_index-start_index-10000;
end




elseif ismember(class, [3 7 9 10 11])
    k = 0.005;
    [p0,onset_curve,p1_5,offset_curve,p3]=piecewise_random_path(class);
```

```
onset_curve_length=length(onset_curve);
offset_curve_length=length(offset_curve);
onset_curve = onset_curve';
offset_curve = offset_curve';


% uncomment this code to do random path
% % One random path - select random point on onset curve and offset curve
random_onset_index=randsample(onset_curve_length,1);
random_offset_index=randsample(offset_curve_length,1);
p1 = onset_curve(:,random_onset_index)';
p2 = offset_curve(:,random_offset_index)';


stall_val = 30000;
[mu2_straight_path0,mu1_straight_path0,nu_straight_path0,rad1] = sphereArcPath(k,tstep,p0,p1);
[mu2_straight_path0_5,mu1_straight_path0_5,nu_straight_path0_5,rad2] =
sphereArcPath(k,tstep,p1,p1_5);
points = repmat(p1_5, stall_val, 1)';
%path noise sigma
sigma_pathnoise = 100;
Rn = [pinknoise([1,length(points)],-1, sigma_pathnoise);pinknoise([1,length(points)],-1,
sigma_pathnoise);pinknoise([1,length(points)],-1, sigma_pathnoise)];
points = points + Rn;
[mu2_straight_path,mu1_straight_path,nu_straight_path,rad3] = sphereArcPath(k,tstep,p1_5,p2);
[mu2_straight_path1,mu1_straight_path1,nu_straight_path1,rad4] = sphereArcPath(k,tstep,p2,p3);
mu2_all = [mu2_straight_path0, mu2_straight_path0_5, points(1, :), mu2_straight_path,
mu2_straight_path1];
mu1_all = [mu1_straight_path0, mu1_straight_path0_5, points(2, :), mu1_straight_path,
mu1_straight_path1];
mu1_all = -mu1_all;
nu_all = [nu_straight_path0, nu_straight_path0_5, points(3,:), nu_straight_path,
nu_straight_path1];

N_t = length(mu2_all);
X = zeros(3,N_t);
xx = x0;

Rn = [pinknoise([1,N_t],-1, sigma);pinknoise([1,N_t],-1, 00);pinknoise([1,N_t],-1, 00)];
mu2_big = zeros(1, length(N_t));
mu1_big = zeros(1, length(N_t));
nu_big = zeros(1, length(N_t));

%%get onset index by finding Radians to bifurcation, and getting index
%%through k and tstep parameters
onset_index = floor((rad1/k)/tstep);
offset_index = floor(((rad1+rad2+rad3)/k)/tstep) + stall_val;
for n = 1:N_t
    %%Euler-Meruyama method
    [Fxx,mu2,mu1,nu] = SlowWave_Model_piecewise(0,xx,b,k,mu2_all(n), mu1_all(n),nu_all(n));
    xx = xx + tstep*Fxx + sqrt(tstep)*Rn(:,n);
    X(:,n) = xx;
```

```
    mu2_big(n) = mu2;
    mu1_big(n) = mu1;
    nu_big(n) = nu;
end
x = X';
signal = x(:,1);
start_index = 1;
stop_index = length(signal);
end






end
```
